# Anemia prevalence in women of reproductive age in low- and middle-income countries between 2000 and 2018

**DOI:** 10.1038/s41591-021-01498-0

**Published:** 2021-10-12

**Authors:** Damaris Kinyoki, Aaron E. Osgood-Zimmerman, Natalia V. Bhattacharjee, Local Burden of Disease Anaemia Collaborators, Lauren E. Schaeffer, Alice Lazzar-Atwood, Dan Lu, Samuel B. Ewald, Katie M. Donkers, Ian D. Letourneau, Michael Collison, Megan F. Schipp, Amanuel Abajobir, Sima Abbasi, Nooshin Abbasi, Mitra Abbasifard, Mohsen Abbasi-Kangevari, Hedayat Abbastabar, Foad Abd-Allah, Ahmed Abdelalim, Sherief M. Abd-Elsalam, Amir Abdoli, Ibrahim Abdollahpour, Aidin Abedi, Hassan Abolhassani, Biju Abraham, Lucas Guimarães Abreu, Michael R. M. Abrigo, Ahmed Abualhasan, Eman Abu-Gharbieh, Abdelrahman I. Abushouk, Manfred Mario Kokou Accrombessi, Maryam Adabi, Oladimeji M. Adebayo, Adeyinka Emmanuel Adegbosin, Victor Adekanmbi, Olatunji O. Adetokunboh, Daniel Adedayo Adeyinka, Davoud Adham, Shailesh M. Advani, Pradyumna Agasthi, Mohammad Aghaali, Sohail Ahmad, Tauseef Ahmad, Keivan Ahmadi, Sepideh Ahmadi, Muktar Beshir Ahmed, Miloud Taki Eddine Aichour, Budi Aji, Oluwaseun Oladapo Akinyemi, Addis Aklilu, Chisom Joyqueenet Akunna, Ziyad Al-Aly, Turki M. Alanzi, Jacqueline Elizabeth Alcalde-Rabanal, Biresaw Wassihun Alemu, Ayinalem Alemu, Robert Kaba Alhassan, Sheikh Mohammad Alif, Vahid Alipour, Hesam Alizade, Syed Mohamed Aljunid, Amir Almasi-Hashiani, Hesham M. Al-Mekhlafi, Rajaa M. Al-Raddadi, Nelson Alvis-Guzman, Saeed Amini, Fatemeh Amiri, Dickson A. Amugsi, Nahla Hamed Anber, Robert Ancuceanu, Tudorel Andrei, Masresha Tessema Anegago, Mina Anjomshoa, Fereshteh Ansari, Alireza Ansari-Moghaddam, Zelalem Alamrew Anteneh, Ernoiz Antriyandarti, Davood Anvari, Razique Anwer, Muhammad Aqeel, Jalal Arabloo, Morteza Arab-Zozani, Olatunde Aremu, Habtamu Abera Areri, Al Artaman, Afsaneh Arzani, Malke Asaad, Mehran Asadi-Aliabadi, Ali A. Asadi-Pooya, Mulusew A. Asemahagn, Mohammad Asghari Jafarabadi, Mengistu M. Ashebir, Zerihun Ataro, Seyyede Masoume Athari, Seyyed Shamsadin Athari, Maha Moh’d Wahbi Atout, Marcel Ausloos, Nefsu Awoke, Beatriz Paulina Ayala Quintanilla, Getinet Ayano, Martin Amogre Ayanore, Yared Asmare Aynalem, Muluken Altaye Ayza, Abbas Azadmehr, Darshan B, Tesleem Kayode Babalola, Alaa Badawi, Ashish D. Badiye, Mohammad Amin Bahrami, Mohan Bairwa, Shankar M. Bakkannavar, Palash Chandra Banik, Adhanom Gebreegziabher Baraki, Miguel A. Barboza, Huda Basaleem, Sanjay Basu, Mohsen Bayati, Bayisa Abdissa Baye, Gholamreza Bazmandegan, Neeraj Bedi, Tariku Tesfaye Tesfaye Bekuma, Michelle L. Bell, Isabela M. Bensenor, Kidanemaryam Berhe, Abadi Kidanemariam Berhe, Kidanemariam Alem Berhie, Dinesh Bhandari, Nikha Bhardwaj, Pankaj Bhardwaj, Krittika Bhattacharyya, Suraj Bhattarai, Zulfiqar A. Bhutta, Ali Bijani, Boris Bikbov, Antonio Biondi, Minyichil Birhanu, Raaj Kishore Biswas, Moses John Bockarie, Somayeh Bohlouli, Mahdi Bohluli, Archith Boloor, Shiva Borzouei, Nicola Luigi Bragazzi, Dejana Braithwaite, Andre R. Brunoni, Sharath Burugina Nagaraja, Zahid A. Butt, Florentino Luciano Caetano dos Santos, Luis Alberto Cámera, Josip Car, Rosario Cárdenas, Felix Carvalho, Joao Mauricio Castaldelli-Maia, Carlos A. Castañeda-Orjuela, Franz Castro, Muge Cevik, Wagaye Fentahun Chanie, Jaykaran Charan, Souranshu Chatterjee, Vijay Kumar Chattu, Sarika Chaturvedi, Simiao Chen, Ken Lee Chin, Mohiuddin Ahsanul Kabir Chowdhury, Aubrey J. Cook, Vera Marisa Costa, Elizabeth A. Cromwell, Berihun Assefa Dachew, Henok Dagne, Baye Dagnew, Tukur Dahiru, Saad M. A. Dahlawi, Haijiang Dai, Hancheng Dai, Lalit Dandona, Rakhi Dandona, Parnaz Daneshpajouhnejad, Farah Daoud, Jai K. Das, Rajat Das Gupta, Aditya Prasad Dash, Claudio Alberto Dávila-Cervantes, Kairat Davletov, Farah Deeba, Jan-Walter De Neve, Edgar Denova-Gutiérrez, Kebede Deribe, Assefa Desalew, Getenet Ayalew Dessie, Sagnik Dey, Meghnath Dhimal, Govinda Prasad Dhungana, Mostafa Dianatinasab, Daniel Diaz, Isaac Oluwafemi Dipeolu, Shirin Djalalinia, Hoa Thi Do, Fariba Dorostkar, Leila Doshmangir, Bereket Duko, Andre Rodrigues Duraes, Lucas Earl, Hisham Atan Edinur, Ferry Efendi, Rajesh Elayedath, Teshome Bekele Elema, Hala Rashad Elhabashy, Shaimaa I. El-Jaafary, Iman El Sayed, Maysaa El Sayed Zaki, Aisha Elsharkawy, Yasser Mohamed El-Sherbiny, Maha El Tantawi, Daniel Adane Endalew, Babak Eshrati, Khalil Eskandari, Sharareh Eskandarieh, Ibtihal Fadhil, Emerito Jose A. Faraon, Mohammad Fareed, Pawan Sirwan Faris, Medhat Farwati, Farshad Farzadfar, Abidemi Omolara Fasanmi, Nazir Fattahi, Nelsensius Klau Fauk, Valery L. Feigin, Berhanu Elfu Feleke, Seyed-Mohammad Fereshtehnejad, Eduarda Fernandes, Pietro Ferrara, Nataliya A. Foigt, Artem Alekseevich Fomenkov, Masoud Foroutan, Joel Msafiri Francis, Richard Charles Franklin, Marisa Freitas, Takeshi Fukumoto, Mohamed M. Gad, Abhay Motiramji Gaidhane, Reta Tsegaye Gayesa, Biniyam Sahiledengle Geberemariyam, Birhan Gebresillassie Gebregiorgis, Hadush Gebremariam, Tesfay B. B. Gebremariam, Leake Gebremeskel, Gebreamlak Gebremedhn Gebremeskel, Assefa Ayalew Gebreslassie, Yilma Chisha Dea Geramo, Hailay Abrha Gesesew, Bradford D. Gessner, Lemma Getacher, Keyghobad Ghadiri, Fatemeh Ghaffarifar, Mansour Ghafourifard, Mahsa Ghajarzadeh, Farhad Ghamari, Ahmad Ghashghaee, Nermin Ghith, Syed Amir Gilani, Tiffany K. Gill, Myron Anthony Godinho, Philimon N. Gona, Ayman Grada, Mohammed Ibrahim Mohialdeen Gubari, Nachiket Gudi, Davide Guido, Rashid Abdi Guled, Yuming Guo, Rachita Gupta, Rajeev Gupta, Arvin Haj-Mirzaian, Randah R. Hamadeh, Demelash Woldeyohannes Handiso, Asif Hanif, Arief Hargono, Ahmed I. Hasaballah, Md Mehedi Hasan, Syed Shahzad Hasan, Maryam Hashemian, Abdiwahab Hashi, Shoaib Hassan, Amr Hassan, Soheil Hassanipour, Hadi Hassankhani, Khezar Hayat, Mohamed I. Hegazy, Reza Heidari-Soureshjani, Nathaniel J. Henry, Claudiu Herteliu, Fatemeh Heydarpour, Sousan Heydarpour, Hagos Degefa de Hidru, Chi Linh Hoang, Ramesh Holla, Julia Hon, Sung Hwi Hong, Praveen Hoogar, Seyyed Nasrollah Hosseini, Mehdi Hosseinzadeh, Mihaela Hostiuc, Sorin Hostiuc, Peter J. Hotez, Mowafa Househ, Tanvir M. Huda, Dawit Hoyiso Huluko Huluko, Syed Ather Hussain, Bing-Fang Hwang, Olayinka Stephen Ilesanmi, Irena M. Ilic, Milena D. Ilic, Leeberk Raja Inbaraj, Usman Iqbal, M. Mofizul Islam, Sheikh Mohammed Shariful Islam, Chinwe Juliana Iwu, Chidozie C. D. Iwu, Farhad Jadidi-Niaragh, Mohammad Ali Jahani, Vardhmaan Jain, Mihajlo Jakovljevic, Amir Jalali, Farzad Jalilian, Manthan Dilipkumar Janodia, Tahereh Javaheri, Ravi Prakash Jha, Oommen John, Kimberly B. Johnson, Jost B. Jonas, Jitendra Jonnagaddala, Nitin Joseph, Ankur Joshi, Farahnaz Joukar, Jacek Jerzy Jozwiak, Ali Kabir, Zubair Kabir, Tanvir Kahlon, Leila R. Kalankesh, Rohollah Kalhor, Ashwin Kamath, Zahra Kamiab, Tanuj Kanchan, Umesh Kapil, Neeti Kapoor, Behzad Karami Matin, Salah Eddin Karimi, Ayele Semachew Kasa, Gebremicheal Gebreslassie Kasahun, Zemenu Yohannes Kassa, Gebrehiwot G. Kassa, Getinet Kassahun, Gbenga A. Kayode, Ali Kazemi Karyani, Tibebeselassie S. Keflie, Peter Njenga Keiyoro, Bayew Kelkay, Maryam Keramati, Daniel Bekele Ketema, Nauman Khalid, Mohammad Khammarnia, Md Nuruzzaman Khan, Maseer Khan, Junaid Khan, Khaled Khatab, Amir M. Khater, Mona M. Khater, Abdullah T. Khoja, Jagdish Khubchandani, Neda Kianipour, Young-Eun Kim, Yun Jin Kim, Ruth W. Kimokoti, Sezer Kisa, Adnan Kisa, Tufa Kolola, Ali Koolivand, Soewarta Kosen, Parvaiz A. Koul, Ai Koyanagi, Kewal Krishan, Vijay Krishnamoorthy, Barthelemy Kuate Defo, Nuworza Kugbey, Vaman Kulkarni, G. Anil Kumar, Nithin Kumar, Pushpendra Kumar, Manasi Kumar, Om P. Kurmi, Dian Kusuma, Ben Lacey, Deepesh P. Lad, Dharmesh Kumar Lal, Faris Hasan Lami, Iván Landires, Anders O. Larsson, Savita Lasrado, Matthew B. Laurens, Carlo La Vecchia, Avula Laxmaiah, Paul H. Lee, Shaun Wen Huey Lee, Kate E. LeGrand, Sonia Lewycka, Bingyu Li, Shanshan Li, Xuefeng Liu, Jaifred Christian F. Lopez, Daiane Borges Machado, Shilpashree Madhava Kunjathur, Hassan Magdy Abd El Razek, Muhammed Magdy Abd El Razek, D. R. Mahadeshwara Prasad, Phetole Walter Mahasha, Mina Maheri, Narayan B. Mahotra, Azeem Majeed, Venkatesh Maled, Shokofeh Maleki, Reza Malekzadeh, Deborah Carvalho Malta, Abdullah A. Mamun, Fariborz Mansour-Ghanaei, Borhan Mansouri, Mohammad Ali Mansournia, Md Dilshad Dilshad Manzar, Carlos Alberto Marrugo Arnedo, Francisco Rogerlândio Martins-Melo, Anthony Masaka, Pallab K. Maulik, Benjamin K. Mayala, Medhin Mehari, Man Mohan Mehndiratta, Entezar Mehrabi Nasab, Fereshteh Mehri, Kala M. Mehta, Wahengbam Bigyananda Meitei, Teferi Mekonnen, Gebrekiros Gebremichael Meles, Mulugeta Melku, Walter Mendoza, Ritesh G. Menezes, Meresa Berwo Mengesha, Endalkachew Worku Mengesha, Tuomo J. Meretoja, Abera M. Mersha, Workua Mekonnen Metekiya, Tomasz Miazgowski, Irmina Maria Michalek, G. K. Mini, Shabir Ahmad Mir, Andreea Mirica, Erkin M. Mirrakhimov, Hamed Mirzaei, Maryam Mirzaei, Mehdi Mirzaei-Alavijeh, Sanjeev Misra, Babak Moazen, Masoud Moghadaszadeh, Yousef Mohammad, Dara K. Mohammad, Naser Mohammad Gholi Mezerji, Seyyede Momeneh Mohammadi, Abdollah Mohammadian-Hafshejani, Reza Mohammadpourhodki, Hayat Maeruf Mohammed, Salahuddin Mohammed, Ammas Siraj Mohammed, Shafiu Mohammed, Jemal Abdu Mohammed, Mohammad A. Mohseni Bandpei, Ali H. Mokdad, Alex Molassiotis, Lorenzo Monasta, Masoud Moradi, Maziar Moradi-Lakeh, Rahmatollah Moradzadeh, Paula Moraga, Abbas Mosapour, Simin Mouodi, Seyyed Meysam Mousavi, Amin Mousavi Khaneghah, Getaneh Baye B. Mulu, Mehnaz Munir, Moses K. Muriithi, G. V. S. Murthy, Ghulam Mustafa, Ashraf F. Nabhan, Mehdi Naderi, Ahamarshan Jayaraman Nagarajan, Shankar Prasad Nagaraju, Mohsen Naghavi, Gurudatta Naik, Mukhammad David Naimzada, Vinay Nangia, Jobert Richie Nansseu, Atta Abbas Naqvi, Bruno Ramos Nascimento, Smitha Nayak, Vinod C. Nayak, Javad Nazari, Rawlance Ndejjo, Ionut Negoi, Ruxandra Irina Negoi, Henok Biresaw Netsere, Georges Nguefack-Tsague, Josephine W. Ngunjiri, Cuong Tat Nguyen, Diep Ngoc Nguyen, Huong Lan Thi Nguyen, Yeshambel T. Nigatu, Rajan Nikbakhsh, Amin Reza Nikpoor, Chukwudi A. Nnaji, Vuong Minh Nong, Jean Jacques Noubiap, Virginia Nunez-Samudio, Vincent Ebuka Nwatah, Tafadzwa Nyanhanda, Bogdan Oancea, Felix Akpojene Ogbo, Onome Bright Oghenetega, In-Hwan Oh, Daniel Micheal Okello, Morteza Oladnabi, Andrew T. Olagunju, Jacob Olusegun Olusanya, Bolajoko Olubukunola Olusanya, Ahmed Omar Bali, Muktar Omer Omer, Abidemi E. Emmanuel Omonisi, Obinna E. Onwujekwe, Alberto Ortiz, Eduardo Ortiz-Panozo, Nikita Otstavnov, Stanislav S. Otstavnov, Mayowa O. Owolabi, P. A. Mahesh, Jagadish Rao Padubidri, Abhijit P. Pakhare, Keyvan Pakshir, Adrian Pana, Songhomitra Panda-Jonas, Anamika Pandey, Seithikurippu R. Pandi-Perumal, Helena Ullyartha Pangaribuan, Deepak Kumar Pasupula, Sangram Kishor Patel, Urvish K. Patel, Ashish Pathak, George C. Patton, Hamidreza Pazoki Toroudi, Jeevan Pereira, Julia Moreira Pescarini, Hai Quang Pham, Brandon V. Pickering, Saeed Pirouzpanah, Meghdad Pirsaheb, Khem Narayan Pokhrel, Maarten J. Postma, Faheem Hyder Pottoo, Hadis Pourchamani, Hadi Pourjafar, Hossein Poustchi, Sergio I. Prada, Dimas Ria Angga Pribadi, Zahiruddin Quazi Syed, Navid Rabiee, Ata Rafiee, Fakher Rahim, Mohammad Hifz Ur Rahman, Muhammad Aziz Rahman, Amir Masoud Rahmani, Rajesh Kumar Rai, Aashish Rajesh, Pradhum Ram, Kiana Ramezanzadeh, Chhabi Lal Ranabhat, Sowmya J. Rao, Satish Rao, Prateek Rastogi, Priya Rathi, Lal Rawal, Wasiq Faraz Rawasia, Reza Rawassizadeh, Lemma Demissie Regassa, Robert C. Reiner, Bhageerathy Reshmi, Nima Rezaei, Omid Rezahosseini, Aziz Rezapour, Seyed Mohammad Riahi, Daniela Ribeiro, Ana Isabel Ribeiro, Jennifer Rickard, Hirbo Shore Roba, Leonardo Roever, Luca Ronfani, Morteza Rostamian, Susan Fred Rumisha, Godfrey M. Rwegerera, Siamak Sabour, Ehsan Sadeghi, Sahar Saeedi Moghaddam, Rajesh Sagar, Amirhossein Sahebkar, Mohammad Ali Sahraian, S. Mohammad Sajadi, Nasir Salam, Marwa Rashad Salem, Hossein Samadi Kafil, Itamar S. Santos, Milena M. Santric-Milicevic, Sivan Yegnanarayana Iyer Saraswathy, Nizal Sarrafzadegan, Benn Sartorius, Arash Sarveazad, Brijesh Sathian, Thirunavukkarasu Sathish, Deepak Saxena, Alyssa N. Sbarra, David C. Schwebel, Anbissa Muleta Senbeta, Debarka Sengupta, Subramanian Senthilkumaran, Sadaf G. Sepanlou, Allen Seylani, Feng Sha, Omid Shafaat, Saeed Shahabi, Mohammad Shahbaz, Izza Shahid, Masood Ali Shaikh, Mohammed Feyisso Shaka, Ali S. Shalash, Mahdi Shamali, Mehran Shams-Beyranvand, MohammadBagher Shamsi, Morteza Shamsizadeh, Mohammed Shannawaz, Kiomars Sharafi, Amrollah Sharifi, Aziz Sheikh, Abbas Sheikhtaheri, Ranjitha S. Shetty, B. Suresh Kumar Shetty, Adithi Shetty, Wondimeneh Shibabaw Shiferaw, Mika Shigematsu, Jae Il Shin, Rahman Shiri, Reza Shirkoohi, Velizar Shivarov, Soraya Siabani, Sudeep K. Siddappa Malleshappa, Tariq Jamal Siddiqi, Negussie Boti Sidemo, Balbir Bagicha Singh, Surya Singh, Yitagesu Sintayehu, Valentin Yurievich Skryabin, Anna Aleksandrovna Skryabina, Mohammad Reza Sobhiyeh, Amin Soheili, Shahin Soltani, Muluken Bekele Sorrie, Emma Elizabeth Spurlock, Chandrashekhar T. Sreeramareddy, Agus Sudaryanto, Mu’awiyyah Babale Sufiyan, Iyad Sultan, Rafael Tabarés-Seisdedos, Takahiro Tabuchi, Biruk Wogayehu Taddele, Eyayou Girma Tadesse, Amir Taherkhani, Zemenu Tamir, Animut Tagele Tamiru, Md Ismail Tareque, Abdelghani Tbakhi, Hirut Teame, Yonas Getaye Tefera, Arash Tehrani-Banihashemi, Yohannes Tekalegn, Merhawi Gebremedhin Tekle, Berhane Fseha Teklehaimanot, Mohamad-Hani Temsah, Getayeneh Antehunegn Tesema, Kavumpurathu Raman Thankappan, Nihal Thomas, Takele Tiki, Asres Bedaso Tilahune, Mariya Vladimirovna Titova, Marcos Roberto Tovani-Palone, Khanh Bao Tran, Bach Xuan Tran, Rajnish Tripathi, Jaya Prasad Tripathy, Phuong N. Truong, Riaz Uddin, Anayat Ullah, Chukwuma David Umeokonkwo, Chigozie Jesse Uneke, Bhaskaran Unnikrishnan, Era Upadhyay, Muhammad Shariq Usman, Marco Vacante, Alireza Vakilian, Sahel Valadan Tahbaz, Pascual R. Valdez, Yasser Vasseghian, Madhur Verma, Francesco S. Violante, Bay Vo, Yohannes Dibaba Wado, Yasir Waheed, Yafeng Wang, Yuan-Pang Wang, Kinley Wangdi, Girmay Teklay Weldesamuel, Andrea Werdecker, Taweewat Wiangkham, Nuwan Darshana Wickramasinghe, Charles Shey Wiysonge, Tewodros Eshete Wonde, Ai-Min Wu, Chenkai Wu, Yang Xie, Ali Yadollahpour, Seyed Hossein Yahyazadeh Jabbari, Tomohide Yamada, Mingyou Yang, Sanni Yaya, Vahid Yazdi-Feyzabadi, Tomas Y. Yeheyis, Alex Yeshaneh, Yigizie Yeshaw, Yordanos Gizachew Yeshitila, Mekdes Tigistu Yilma, Paul Yip, Melissa F. Young, Zabihollah Yousefi, Taraneh Yousefinezhadi, Hebat-Allah Salah A. Yousof, Abdilahi Yousuf Yousuf, Chuanhua Yu, Yong Yu, Shamsa Zafar, Syed Saoud Zaidi, Zoubida Zaidi, Josefina Zakzuk, Sojib Bin Zaman, Mohammad Zamani, Maryam Zamanian, Alireza Zandifar, Alireza Zangeneh, Mikhail Sergeevich Zastrozhin, Anasthasia Zastrozhina, Dejene Tesfaye Zewdie, Kaleab Alemayehu Zewdie, Yunquan Zhang, Cong Zhu, Arash Ziapour, Nicholas J. Kassebaum, Simon I. Hay

**Affiliations:** 1 Institute for Health Metrics and Evaluation, University of Washington, Seattle, WA USA; 2 Department of Health Metrics Sciences, School of Medicine, University of Washington, Seattle, WA USA; 3 Department of Anesthesiology & Pain Medicine, University of Washington, Seattle, WA USA; 4 Department of Global Health, University of Washington, Seattle, WA USA; 5 Global Programs, Medical Teams International, Seattle, WA USA; 6 Department of Pediatric Newborn Medicine, Brigham and Women’s Hospital, Boston, MA USA; 7 Maternal and Child Wellbeing, African Population and Health Research Center, Nairobi, Kenya; 8 Mashhad University of Medical Sciences, Mashhad, Iran; 9 Montreal Neurological Institute, McGill University, Montreal, QC Canada; 10 Department of Internal Medicine, Rafsanjan University of Medical Sciences, Rafsanjan, Iran; 11 Clinical Research Development Unit, Rafsanjan University of Medical Sciences, Rafsanjan, Iran; 12 Social Determinants of Health Research Center, Shahid Beheshti University of Medical Sciences, Tehran, Iran; 13 Advanced Diagnostic and Interventional Radiology Research Center, Tehran University of Medical Sciences, Tehran, Iran; 14 Department of Neurology, Cairo University, Cairo, Egypt; 15 Tropical Medicine Department, Tanta University, Tanta, Egypt; 16 Department of Parasitology and Mycology, Jahrom University of Medical Sciences, Jahrom, Iran; 17 Neuroscience Research Center, Isfahan University of Medical Sciences, Isfahan, Iran; 18 Department of Orthopaedic Surgery, University of Southern California, Los Angeles, CA USA; 19 Department of Laboratory Medicine, Karolinska University Hospital, Huddinge, Sweden; 20 Research Center for Immunodeficiencies, Tehran University of Medical Sciences, Tehran, Iran; 21 Department of Economics, NMSM Government College, Kalpetta, India; 22 Department of Pediatric Dentistry, Federal University of Minas Gerais, Belo Horizonte, Brazil; 23 Department of Research, Philippine Institute for Development Studies, Quezon City, Philippines; 24 Department of Clinical Sciences, University of Sharjah, Sharjah, United Arab Emirates; 25 Harvard Medical School, Harvard University, Boston, MA USA; 26 Department of Medicine, Ain Shams University, Cairo, Egypt; 27 Department of Disease Control, London School of Hygiene & Tropical Medicine, London, UK; 28 Clinical Research and Operations, Foundation for Scientific Research (FORS), Cotonou, Benin; 29 Hamadan University of Medical Sciences, Hamadan, Iran; 30 College of Medicine, University College Hospital, Ibadan, Ibadan, Nigeria; 31 School of Medicine, Griffith University, Gold Coast, QLD Australia; 32 Population Health Sciences, King’s College London, London, UK; 33 Centre of Excellence for Epidemiological Modelling and Analysis, Stellenbosch University, Stellenbosch, South Africa; 34 Department of Global Health, Stellenbosch University, Cape Town, South Africa; 35 Department of Community Health and Epidemiology, University of Saskatchewan, Saskatoon, SK Canada; 36 Department of Public Health, Federal Ministry of Health, Abuja, Nigeria; 37 School of Health, Ardabil University of Medical Science, Ardabil, Iran; 38 Social Behavioral Research Branch, National Institute of Health, Bethesda, MD USA; 39 Department of Oncology, Georgetown University, Washington DC, USA; 40 Department of Cardiovascular Medicine, Mayo Clinic, Scottsdale, AZ USA; 41 Department of Epidemiology and Biostatistics, Qom University of Medical Sciences, Qom, Iran; 42 Faculty of Pharmacy, MAHSA University, Kuala Langat, Malaysia; 43 Department of Epidemiology and Health Statistics, Southeast University, Nanjing, China; 44 Lincoln Medical School, Universities of Nottingham & Lincoln, Lincoln, UK; 45 School of Advanced Technologies in Medicine, Shahid Beheshti University of Medical Sciences, Tehran, Iran; 46 Department of Epidemiology, Jimma University, Jimma, Ethiopia; 47 Australian Center for Precision Health, University of South Australia, Adelaide, SA Australia; 48 Higher National School of Veterinary Medicine, Algiers, Algeria; 49 Faculty of Medicine and Public Health, Jenderal Soedirman University, Purwokerto, Indonesia; 50 Department of Health Policy and Management, University of Ibadan, Ibadan, Nigeria; 51 Department of Community Medicine, University College Hospital, Ibadan, Ibadan, Nigeria; 52 Department of Medical Laboratory Sciences, Arba Minch University, Arba Minch, Ethiopia; 53 Department of Public Health, The Intercountry Centre for Oral Health (ICOH) for Africa, Jos, Nigeria; 54 Department of Public Health, Federal Ministry of Health, Garki, Nigeria; 55 John T. Milliken Department of Internal Medicine, Washington University in St. Louis, St. Louis, MO USA; 56 Clinical Epidemiology Center, Department of Veterans Affairs, St. Louis, MO USA; 57 Health Information Management and Technology Department, Imam Abdulrahman Bin Faisal University, Dammam, Saudi Arabia; 58 Center for Health System Research, National Institute of Public Health, Cuernavaca, Mexico; 59 College of Medicine and Health Science, Arba Minch University, Arba Minch, Ethiopia; 60 Department of Midwifery, Arba Minch University, Injbara, Ethiopia; 61 HIV and TB Research Directorate, Ethiopian Public Health Institute, Addis Ababa, Ethiopia; 62 Institute of Health Research, University of Health and Allied Sciences, Ho, Ghana; 63 Epidemiology and Preventive Medicine, Monash University, Melbourne, VIC Australia; 64 Health Management and Economics Research Center, Iran University of Medical Sciences, Tehran, Iran; 65 Health Economics Department, Iran University of Medical Sciences, Tehran, Iran; 66 Infectious and Tropical Disease Research Center, Hormozgan University of Medical Sciences, Bandar Abbas, Iran; 67 Department of Health Policy and Management, Kuwait University, Safat, Kuwait; 68 International Centre for Casemix and Clinical Coding, National University of Malaysia, Bandar Tun Razak, Malaysia; 69 Department of Epidemiology, Arak University of Medical Sciences, Arak, Iran; 70 Medical Research Center, Jazan University, Jazan, Saudi Arabia; 71 Department of Parasitology, Sana’a University, Sana’a, Yemen; 72 Department of Community Medicine, King Abdulaziz University, Jeddah, Saudi Arabia; 73 Research Group in Health Economics, University of Cartagena, Cartagena, Colombia; 74 Research Group in Hospital Management and Health Policies, ALZAK Foundation, Cartagena, Colombia; 75 Health Services Management Department, Arak University of Medical Sciences, Arak, Iran; 76 Department of Radiology and Nuclear Medicine, Kermanshah University of Medical Sciences, Kermanshah, Iran; 77 Mansoura University, Mansoura, Egypt; 78 Faculty of Medicine, Mansoura University, Mansoura, Egypt; 79 Pharmacy Department, Carol Davila University of Medicine and Pharmacy, Bucharest, Romania; 80 Department of Statistics and Econometrics, Bucharest University of Economic Studies, Bucharest, Romania; 81 Division of Human Nutrition and Health, Wageningen University & Research, Wageningen, Netherlands; 82 Nutrition & Food Science Research Directorate, Ethiopian Public Health Institute, Addis Ababab, Ethiopia; 83 Social Determinants of Health Research Center, Rafsanjan University of Medical Sciences, Rafsanjan, Iran; 84 Research Center for Evidence Based Medicine, Tabriz University of Medical Sciences, Tabriz, Iran; 85 Razi Vaccine and Serum Research Institute, Agricultural Research, Education, and Extension Organization (AREEO), Tehran, Iran; 86 Department of Epidemiology and Biostatistics, Zahedan University of Medical Sciences, Zahedan, Iran; 87 Department of Epidemiology, Bahir Dar University, Bahir Dar, Ethiopia; 88 Agribusiness Study Program, Sebelas Maret University, Surakarta, Indonesia; 89 Department of Parasitology, Mazandaran University of Medical Sciences, Sari, Iran; 90 Department of Parasitology, Iranshahr University of Medical Sciences, Iranshahr, Iran; 91 Department of Pathology, Imam Mohammad Ibn Saud Islamic University, Riyadh, Saudi Arabia; 92 Department of Psychology, Foundation University Islamabad, Rawalpandi, Pakistan; 93 Social Determinants of Health Research Center, Birjand University of Medical Sciences, Birjand, Iran; 94 Department of Public Health, Birmingham City University, Birmingham, UK; 95 School of Nursing and Midwifery, Addis Ababa University, Addis Ababa, Ethiopia; 96 Independent Consultant, Windsor, MB Canada; 97 School of Nursing and Midwifery, Babol University of Medical Sciences, Babol, Iran; 98 Babol University of Medical Sciences, Babol, Iran; 99 Department of Plastic Surgery, The University of Texas Health Science Center at Houston, Houston, TX USA; 100 Preventive Medicine and Public Health Research Center, Iran University of Medical Sciences, Tehran, Iran; 101 Epilepsy Research Center, Shiraz University of Medical Sciences, Shiraz, Iran; 102 Department of Neurology, Thomas Jefferson University, Philadelphia, PA USA; 103 School of Public Health, Bahir Dar University, Bahir Dar, Ethiopia; 104 Department of Biostatistics and Epidemiology, Tabriz University of Medical Sciences, Tabriz, Iran; 105 Department of Biostatistics and Epidemiology, Zanjan University of Medical Sciences, Zanjan, Iran; 106 School of Public Health, Mekelle University, Mekelle, Ethiopia; 107 Department of Medical Laboratory Science, Haramaya University, Harar, Ethiopia; 108 Department of Biology, Maragheh University of Medical Sciences, Maragheh, Iran; 109 Department of Immunology, Zanjan University of Medical Sciences, Zanjan, Iran; 110 Faculty of Nursing, Philadelphia University, Amman, Jordan; 111 School of Business, University of Leicester, Leicester, UK; 112 Department of Nursing, Wolaita Sodo University, Wolaita Sodo, Ethiopia; 113 The Judith Lumley Centre, La Trobe University, Melbourne, VIC Australia; 114 School of Public Health, Curtin University, Perth, WA Australia; 115 Department of Health Policy Planning and Management, University of Health and Allied Sciences, Ho, Ghana; 116 Department of Nursing, Debre Berhan University, Debre Berhan, Ethiopia; 117 Department of Pharmacology and Toxicology, Mekelle University, Mekelle, Ethiopia; 118 Cellular and Molecular Biology Research Center, Babol University of Medical Sciences, Babol, Iran; 119 Department of Community Medicine, Manipal Academy of Higher Education, Mangalore, India; 120 Department of Public Health Medicine, University of KwaZulu-Natal, Durban, South Africa; 121 Department of Community Health and Primary Care, University of Lagos, Lagos, Nigeria; 122 Public Health Risk Sciences Division, Public Health Agency of Canada, Toronto, ON Canada; 123 Department of Nutritional Sciences, University of Toronto, Toronto, ON Canada; 124 Department of Forensic Science, Government Institute of Forensic Science, Nagpur, India; 125 Department of Healthcare Management and Education, Shiraz University of Medical Sciences, Shiraz, Iran; 126 Centre for Community Medicine, All India Institute of Medical Sciences, New Delhi, India; 127 Department of Forensic Medicine and Toxicology, Manipal Academy of Higher Education, Manipal, India; 128 Department of Non-communicable Diseases, Bangladesh University of Health Sciences, Dhaka, Bangladesh; 129 Department of Epidemiology and Biostatistics, University of Gondar, Gondar, Ethiopia; 130 Department of Neurosciences, Costa Rican Department of Social Security, San Jose, Costa Rica; 131 School of Medicine, University of Costa Rica, San Pedro, Costa Rica; 132 School of Public Health and Community Medicine, Aden College, Aden, Yemen; 133 Center for Primary Care, Harvard University, Boston, MA USA; 134 School of Public Health, Imperial College London, London, UK; 135 Health Human Resources Research Center, Shiraz University of Medical Sciences, Shiraz, Iran; 136 Department of Public Health, Ambo University, Ambo, Ethiopia; 137 Department of Community Medicine, Gandhi Medical College Bhopal, Bhopal, India; 138 Jazan University, Jazan, Saudi Arabia; 139 Department of Public Health, Wollega University, Nekemte, Ethiopia; 140 School of the Environment, Yale University, New Haven, CT USA; 141 Department of Internal Medicine, University of São Paulo, São Paulo, Brazil; 142 Department of Nutrition and Dietetics, Mekelle University, Mekelle, Ethiopia; 143 College of Medicine and Health Sciences, Adigrat University, Adigrat, Ethiopia; 144 Department of Biostatistics, Mekelle University, Mekelle, Ethiopia; 145 School of Public Health, University of Adelaide, Adelaide, SA Australia; 146 Public Health Research Laboratory, Tribhuvan University, Kathmandu, Nepal; 147 Department of Anatomy, Government Medical College Pali, Pali, India; 148 Department of Community Medicine and Family Medicine, All India Institute of Medical Sciences, Jodhpur, India; 149 School of Public Health, All India Institute of Medical Sciences, Jodhpur, India; 150 Department of Statistical and Computational Genomics, National Institute of Biomedical Genomics, Kalyani, India; 151 Department of Statistics, University of Calcutta, Kolkata, India; 152 Department of Global Health, Global Institute for Interdisciplinary Studies, Kathmandu, Nepal; 153 Centre for Global Child Health, University of Toronto, Toronto, ON Canada; 154 Centre of Excellence in Women & Child Health, Aga Khan University, Karachi, Pakistan; 155 Social Determinants of Health Research Center, Babol University of Medical Sciences, Babol, Iran; 156 Mario Negri Institute for Pharmacological Research, Ranica, Italy; 157 Department of General Surgery and Medical-Surgical Specialties, University of Catania, Catania, Italy; 158 Department of Pediatrics and Child Health Nursing, Bahir Dar University, Bahir Dar, Ethiopia; 159 Transport and Road Safety (TARS) Research Centre, University of New South Wales, Sydney, NSW Australia; 160 European & Developing Countries Clinical Trials Partnership, Cape Town, South Africa; 161 Department of Medicine, University of Cape Town, Cape Town, South Africa; 162 Department of Veterinary Medicine, Islamic Azad University, Kermanshah, Iran; 163 Department of Computer Science and Information Technology, Institute for Advanced Studies in Basic Sciences, Zanjan, Iran; 164 Department of Research and Innovation, Petanux Research GmBH, Bonn, Germany; 165 Department of Internal Medicine, Manipal Academy of Higher Education, Mangalore, India; 166 Department of Endocrinology, Hamadan University of Medical Sciences, Hamadan, Iran; 167 University of Genoa, Genoa, Italy; 168 Department of Epidemiology, University of Florida, Gainesville, FL USA; 169 Cancer Population Sciences Program, University of Florida Health Cancer Center, Gainesville, FL USA; 170 Department of Psychiatry, University of São Paulo, São Paulo, Brazil; 171 Department of Community Medicine, Employee State Insurance Post Graduate Institute of Medical Sciences and Research, Bangalore, India; 172 School of Public Health and Health Systems, University of Waterloo, Waterloo, ON Canada; 173 Al Shifa School of Public Health, Al Shifa Trust Eye Hospital, Rawalpindi, Pakistan; 174 Institute of Microengineering, Federal Polytechnic School of Lausanne, Lausanne, Switzerland; 175 Internal Medicine Department, Italian Hospital of Buenos Aires (Hospital Italiano de Buenos Aires), Buenos Aires, Argentina; 176 Board of Directors, Argentine Society of Medicine, Buenos Aires, Argentina; 177 Centre for Population Health Sciences, Nanyang Technological University, Singapore, Singapore; 178 Department of Primary Care and Public Health, Imperial College London, London, UK; 179 Department of Health Care, Metropolitan Autonomous University, Mexico City, Mexico; 180 Research Unit on Applied Molecular Biosciences (UCIBIO), University of Porto, Porto, Portugal; 181 Colombian National Health Observatory, National Institute of Health, Bogota, Colombia; 182 Epidemiology and Public Health Evaluation Group, National University of Colombia, Bogota, Colombia; 183 Gorgas Memorial Institute for Health Studies, Panama City, Panama; 184 Infection and Global Health Research, University of St. Andrews, St. Andrews, UK; 185 Regional Infectious Diseases Unit, NHS National Services Scotland, Edinburgh, UK; 186 Institute of Public Health, University of Gondar, Gondar, Ethiopia; 187 School of Public Health, Haramaya University, Harar, Ethiopia; 188 Department of Pharmacology, All India Institute of Medical Sciences, Jodhpur, India; 189 Department of Microbiology & Infection Control, Medanta Medicity, Gurugram, India; 190 Department of Medicine, University of Toronto, Toronto, ON Canada; 191 Research Department, D. Y. Patil University, Pune, India; 192 Heidelberg Institute of Global Health (HIGH), Heidelberg University, Heidelberg, Germany; 193 Department of Epidemiology and Preventive Medicine, Monash University, Melbourne, VIC Australia; 194 Melbourne Medical School, University of Melbourne, Parkville, VIC Australia; 195 Maternal and Child Health Division, International Centre for Diarrhoeal Disease Research, Bangladesh, Dhaka, Bangladesh; 196 Department of Epidemiology and Biostatistics, University of South Carolina, Columbia, SC USA; 197 Department of Epidemiology, University of Gondar, Gondar, Ethiopia; 198 Department of Environmental Health and Occupational Health and Safety, University of Gondar, Gondar, Ethiopia; 199 Department of Human Physiology, University of Gondar, Gondar, Ethiopia; 200 Department of Community Medicine, Ahmadu Bello University, Zaria, Nigeria; 201 Environmental Health Department, Imam Abdulrahman Bin Faisal University, Dammam, Saudi Arabia; 202 Department of Cardiology, Central South University, Changsha, China; 203 Department of Mathematics and Statistics, York University, Toronto, ON Canada; 204 College of Environmental Sciences and Engineering, Peking University, Beijing, China; 205 Public Health Foundation of India, Gurugram, India; 206 Indian Council of Medical Research, New Delhi, India; 207 Department of Pathology, Johns Hopkins University School of Medicine, Baltimore, MD USA; 208 Department of Pathology, Isfahan University of Medical Sciences, Isfahan, Iran; 209 Division of Women and Child Health, Aga Khan University, Karachi, Pakistan; 210 James P Grant School of Public Health, BRAC University, Dhaka, Bangladesh; 211 Asian Institute of Public Health University, Bhubaneswar, India; 212 Department of Population and Development, Latin American Faculty of Social Sciences Mexico, Mexico City, Mexico; 213 Health Research Institute, Al Farabi Kazakh National University, Almaty, Kazakhstan; 214 Centre for Interdisciplinary Research in Basic Sciences, Jamia Millia Islamia, Delhi, India; 215 Center for Nutrition and Health Research, National Institute of Public Health, Cuernavaca, Mexico; 216 Wellcome Trust Brighton and Sussex Centre for Global Health Research, Brighton and Sussex Medical School, Brighton, UK; 217 School of Public Health, Addis Ababa University, Addis Ababa, Ethiopia; 218 School of Nursing and Midwifery, Haramaya University, Harar, Ethiopia; 219 Department of Nursing, Bahir Dar University, Bahir Dar, Ethiopia; 220 Centre for Atmospheric Sciences, Indian Institute of Technology Delhi, New Delhi, India; 221 Health Research Section, Nepal Health Research Council, Kathmandu, Nepal; 222 Department of Microbiology, Far Western University, Mahendranagar, Nepal; 223 Department of Epidemiology and Biostatistics, Shahroud University of Medical Sciences, Shahroud, Iran; 224 Department of Epidemiology, Shiraz University of Medical Sciences, Shiraz, Iran; 225 Center of Complexity Sciences, National Autonomous University of Mexico, Mexico City, Mexico; 226 Faculty of Veterinary Medicine and Zootechnics, Autonomous University of Sinaloa, Culiacán Rosales, Mexico; 227 Department of Health Promotion and Education, University of Ibadan, Ibadan, Nigeria; 228 Development of Research and Technology Center, Ministry of Health and Medical Education, Tehran, Iran; 229 Institute of Health Economics and Technology, Hanoi, Vietnam; 230 Department of Medical Laboratory Sciences, Faculty of Allied Medicine, Tehran, Iran; 231 Department of Health Policy and Management, Tabriz University of Medical Sciences, Tabriz, Iran; 232 School of Public Health, Hawassa University, Hawassa, Ethiopia; 233 School of Medicine, Federal University of Bahia, Salvador, Brazil; 234 Department of Internal Medicine, Bahiana School of Medicine and Public Health (Escola Bahiana de Medicina e Saúde Pública), Salvador, Brazil; 235 School of Health Sciences, University of Science Malaysia (Universiti Sains Malaysia), Kubang Kerian, Kelantan Malaysia; 236 Department of Community Health Nursing, Airlangaa University (Universitas Airlangga), Surabaya, Indonesia; 237 School of Nursing and Midwifery, La Trobe University, Melbourne, VIC Australia; 238 School of Behavioural Sciences, Mahatma Gandhi University of Medical Sciences and Technology, Kottayam, India; 239 Department of Food Science and Nutrition, Arsi University, Asella, Ethiopia; 240 Center for Food Science and Nutrition, Addis Ababa University, Addis Ababa, Ethiopia; 241 Neurophysiology Department, Cairo University, Cairo, Egypt; 242 Biomedical Informatics and Medical Statistics Department, Alexandria University, Alexandria, Egypt; 243 Reference Laboratory of Egyptian Universities Hospitals, Ministry of Higher Education and Research, Cairo, Egypt; 244 Endemic Medicine and Hepatogastroentrology Department, Cairo University, Cairo, Egypt; 245 Department of Biosciences, Nottingham Trent University, Nottingham, UK; 246 Clinical Pathology Department, Mansoura University, Mansoura, Egypt; 247 Pediatric Dentistry and Dental Public Health Department, Alexandria University, Alexandria, Egypt; 248 Department of Midwifery, Wolkite University, Wolkite, Ethiopia; 249 Department of Medicinal Chemistry, Kerman University of Medical Sciences, Kerman, Iran; 250 Pharmaceutics Research Center, Kerman University of Medical Sciences, Kerman, Iran; 251 Multiple Sclerosis Research Center, Tehran University of Medical Sciences, Tehran, Iran; 252 Division of Non-Communicable Diseases, Ministry of Public Health and Population, Dubai, United Arab Emirates; 253 Department of Health Policy and Administration, University of the Philippines Manila, Manila, Philippines; 254 College of Medicine, Imam Mohammad Ibn Saud Islamic University, Riyadh, Saudi Arabia; 255 Department of Biology and Biotechnology ‘Lazzaro Spallanzani’, University of Pavia, Pavia, Italy; 256 Department of Biology, Cihan University-Erbil, Erbil, Iraq; 257 Internal Medicine Department, Cleveland Clinic, Cleveland, OH USA; 258 Department of Cardiovascular Medicine, Mayo Clinic, Rochester, MN USA; 259 Non-communicable Diseases Research Center, Tehran University of Medical Sciences, Tehran, Iran; 260 Satcher Health Leadership Institute, Morehouse School of Medicine, Atlanta, GA USA; 261 School of Medicine, Emory University, Atlanta, GA USA; 262 Research Center for Environmental Determinants of Health, Kermanshah University of Medical Sciences, Kermanshah, Iran; 263 College of Medicine and Public Health, Flinders University, Adelaide, SA Australia; 264 Institute of Resource Governance and Social Change, Kupang, Indonesia; 265 National Institute for Stroke and Applied Neurosciences, Auckland University of Technology, Auckland, New Zealand; 266 Research Center of Neurology, Moscow, Russia; 267 Department of Epidemiology and Biostatistics, Bahir Dar University, Bahir Dar, Ethiopia; 268 Department of Neurobiology, Karolinska Institute, Stockholm, Sweden; 269 Division of Neurology, University of Ottawa, Ottawa, ON Canada; 270 Associated Laboratory for Green Chemistry (LAQV), University of Porto, Porto, Portugal; 271 Research Center on Public Health, University of Milan Bicocca, Monza, Italy; 272 Institute of Gerontology, National Academy of Medical Sciences of Ukraine, Kyiv, Ukraine; 273 Department of Cell Biology and Biotechnology, K.A. Timiryazev Institute of Plant Physiology, Moscow, Russia; 274 Department of Medical Parasitology, Abadan Faculty of Medical Sciences, Abadan, Iran; 275 Department of Family Medicine and Primary Care, University of the Witwatersrand, Johannesburg, South Africa; 276 School of Public Health, Medical, and Veterinary Sciences, James Cook University, Douglas, QLD Australia; 277 Department of Dermatology, Kobe University, Kobe, Japan; 278 Department of Cardiovascular Medicine, Cleveland Clinic, Cleveland, OH USA; 279 Gillings School of Global Public Health, University of North Carolina Chapel Hill, Chapel Hill, NC USA; 280 Department of Community Medicine, Datta Meghe Institute of Medical Sciences, Wardha, India; 281 Department of Nursing, Wollega University, Nekemte, Ethiopia; 282 Department of Public Health, Madda Walabu University, Bale Robe, Ethiopia; 283 Department of Human Nutrition, Aksum University, Mekelle, Ethiopia; 284 School of Pharmacy, Aksum University, Aksum, Ethiopia; 285 Department of Pharmacy, Mekelle University, Mekelle, Ethiopia; 286 Department of Nursing, Aksum University, Aksum, Ethiopia; 287 Department of Nursing, Mekelle University, Mekelle, Ethiopia; 288 Department of Reproductive Health, Mekelle University, Mekelle, Ethiopia; 289 Department of Public Health, Arba Minch University, Arba Minch, Ethiopia; 290 Department of Epidemiology, Mekelle University, Mekelle, Ethiopia; 291 Pfizer Vaccines, Collegeville, PA USA; 292 Agency of Preventive Medicine, Paris, France; 293 Department of Public Health, Debre Berhan University, Debre Berhan, Ethiopia; 294 Infectious Disease Research Center, Kermanshah University of Medical Sciences, Kermanshah, Iran; 295 Pediatric Department, Kermanshah University of Medical Sciences, Kermanshah, Iran; 296 Department of Parasitology and Entomology, Tarbiat Modares University, Tehran, Iran; 297 Department of Medical Surgical Nursing, Tabriz University of Medical Sciences, Tabriz, Iran; 298 Department of Neurology, Tehran University of Medical Sciences, Tehran, Iran; 299 Occupational Health Department, Arak University of Medical Sciences, Arak, Iran; 300 Student Research Committee, Iran University of Medical Sciences, Tehran, Iran; 301 Research Group for Genomic Epidemiology, Technical University of Denmark, Copenhagen, Denmark; 302 Faculty of Allied Health Sciences, The University of Lahore, Lahore, Pakistan; 303 Afro-Asian Institute, Lahore, Pakistan; 304 Adelaide Medical School, University of Adelaide, Adelaide, SA Australia; 305 School of Public Health and Community Medicine, University of New South Wales, Kensington, NSW Australia; 306 Department of Exercise and Health Sciences, University of Massachusetts, Boston, Boston, MA USA; 307 Department of Dermatology, Boston University, Boston, MA USA; 308 Department of Family and Community Medicine, University of Sulaimani, Sulaimani, Iraq; 309 Department of Health Policy, Manipal Academy of Higher Education, Manipal, India; 310 Neurology, Public Health and Disability Unit, Carlo Besta Neurological Institute IRCCS (Fondazione IRCCS Istituto Neurologico Carlo Besta), Milan, Italy; 311 College of Medicine and Health Science, Jigjiga University, Jijiga, Ethiopia; 312 Department of Epidemiology, Binzhou Medical University, Yantai City, China; 313 Non-communicable Diseases Department, World Health Organization, New Delhi, India; 314 Department of Preventive Cardiology, Eternal Heart Care Centre & Research Institute, Jaipur, India; 315 Department of Medicine, Mahatma Gandhi University Medical Sciences, Jaipur, India; 316 Department of Pharmacology, Tehran University of Medical Sciences, Tehran, Iran; 317 Obesity Research Center, Shahid Beheshti University of Medical Sciences, Tehran, Iran; 318 Department of Family and Community Medicine, Arabian Gulf University, Manama, Bahrain; 319 Department of Public Health, Wachemo University, Hossana, Ethiopia; 320 University Institute of Public Health, The University of Lahore, Lahore, Pakistan; 321 Department of Epidemiology, Airlangga University (Universitas Airlangga), Surabaya, Indonesia; 322 Department of Zoology and Entomology, Al Azhar University, Cairo, Egypt; 323 Institute for Social Science Research, The University of Queensland, Indooroopilly, QLD Australia; 324 ARC Centre of Excellence for Children and Families over the Life Course, The University of Queensland, Indooroopilly, QLD Australia; 325 Department of Pharmacy, University of Huddersfield, Huddersfield, UK; 326 School of Biomedical Sciences and Pharmacy, University of Newcastle, Newcastle, NSW Australia; 327 Biology Department, Utica College, Utica, NY USA; 328 Digestive Diseases Research Institute, Tehran University of Medical Sciences, Tehran, Iran; 329 Department of Public Health, Jigjiga University, Jijiga, Ethiopia; 330 Center for International Health (CIH), University of Bergen, Bergen, Norway; 331 Bergen Center for Ethics and Priority Setting (BCEPS), University of Bergen, Bergen, Norway; 332 Gastrointestinal and Liver Diseases Research Center, Guilan University of Medical Sciences, Rasht, Iran; 333 Caspian Digestive Disease Research Center, Guilan University of Medical Sciences, Rasht, Iran; 334 School of Nursing and Midwifery, Tabriz University of Medical Sciences, Tabriz, Iran; 335 Independent Consultant, Tabriz, Iran; 336 Institute of Pharmaceutical Sciences, University of Veterinary and Animal Sciences, Lahore, Pakistan; 337 Department of Pharmacy Administration and Clinical Pharmacy, Xian Jiaotong University, Xian, China; 338 School of Nursing and Midwifery, Tehran University of Medical Sciences, Tehran, Iran; 339 Big Data Institute, University of Oxford, Oxford, UK; 340 School of Business, London South Bank University, London, UK; 341 School of Nursing and Midwifery, Kermanshah University of Medical Sciences, Kermanshah, Iran; 342 Department of Public Health, Adigrat University, Adigrat, Ethiopia; 343 Center of Excellence in Behavioral Medicine, Nguyen Tat Thanh University, Ho Chi Minh City, Vietnam; 344 Kasturba Medical College, Mangalore, Manipal Academy of Higher Education, Manipal, India; 345 Department of Pediatrics, Yonsei University, Seoul, South Korea; 346 Research Department, Electronic Medical Records for the Developing World, York, UK; 347 Centre for Bio Cultural Studies (CBiCS), Manipal Academy of Higher Education, Manipal, India; 348 Deputy of Education, Iranian Ministry of Health and Medical Education, Tehran, Iran; 349 Institute of Research and Development, Duy Tan University, Da Nang, Vietnam; 350 Department of Computer Science, University of Human Development, Sulaymaniyah, Iraq; 351 Department of Internal Medicine, Carol Davila University of Medicine and Pharmacy, Bucharest, Romania; 352 Department of Legal Medicine and Bioethics, Carol Davila University of Medicine and Pharmacy, Bucharest, Romania; 353 Clinical Legal Medicine Department, National Institute of Legal Medicine Mina Minovici, Bucharest, Romania; 354 National School of Tropical Medicine, Baylor College of Medicine, Houston, TX USA; 355 College of Science and Engineering, Hamad Bin Khalifa University, Doha, Qatar; 356 School of Public Health, University of Sydney, Sydney, NSW Australia; 357 School of Nursing, Hawassa University, Hawassa, Ethiopia; 358 Department of Internal Medicine, Rochester General Hospital, Rochester, NY USA; 359 Department of Occupational Safety and Health, China Medical University, Taichung, Taiwan; 360 Department of Community Medicine, University of Ibadan, Ibadan, Nigeria; 361 Faculty of Medicine, University of Belgrade, Belgrade, Serbia; 362 Department of Epidemiology, University of Kragujevac, Kragujevac, Serbia; 363 Division of Community Health and Family Medicine, Bangalore Baptist Hospital, Bangalore, India; 364 College of Public Health, Taipei Medical University, Taipei, Taiwan; 365 School of Psychology and Public Health, La Trobe University, Melbourne, VIC Australia; 366 Institute for Physical Activity and Nutrition, Deakin University, Melbourne, VIC Australia; 367 Sydney Medical School, University of Sydney, Sydney, NSW Australia; 368 South African Medical Research Council, Cape Town, South Africa; 369 School of Health Systems and Public Health, University of Pretoria, Pretoria, South Africa; 370 Department of Immunology, Tabriz University of Medical Sciences, Tabriz, Iran; 371 Department of Internal Medicine, Cleveland Clinic, Cleveland, OH USA; 372 N. A. Semashko Department of Public Health and Healthcare, I.M. Sechenov First Moscow State Medical University, Moscow, Russia; 373 Department of Global Health, Economics and Policy, University of Kragujevac, Kragujevac, Serbia; 374 Health Institute, Kermanshah University of Medical Sciences, Kermanshah, Iran; 375 Substance Abuse Prevention Research Center, Kermanshah University of Medical Sciences, Kermanshah, Iran; 376 Social Development and Health Promotion Research Center, Kermanshah University of Medical Sciences, Kermanshah, Iran; 377 Manipal College of Pharmaceutical Sciences, Manipal Academy of Higher Education, Manipal, India; 378 Health Informatic Lab, Boston University, Boston, MA USA; 379 Department of Community Medicine, Baba Saheb Ambedkar Medical College & Hospital, Delhi, India; 380 Department of Community Medicine, Banaras Hindu University, Varanasi, India; 381 Renal and Cardiovascular Division, The George Institute for Global Health, New Delhi, India; 382 Department of Medicine, University of New South Wales, Sydney, NSW Australia; 383 Department of Ophthalmology, Heidelberg University, Heidelberg, Germany; 384 Beijing Institute of Ophthalmology, Beijing Tongren Hospital, Beijing, China; 385 New South Wales Health, Sydney, NSW Australia; 386 Department of Community Medicine and Family Medicine, All India Institute of Medical Sciences, Bhopal, India; 387 Department of Family Medicine and Public Health, University of Opole, Opole, Poland; 388 Minimally Invasive Surgery Research Center, Iran University of Medical Sciences, Tehran, Iran; 389 School of Public Health, University College Cork, Cork, Ireland; 390 Division of Cardiology, University of Louisville, Louisville, KY USA; 391 Department of Medicine, Albert Einstein College of Medicine, Bronx, NY USA; 392 School of Management and Medical Informatics, Tabriz University of Medical Sciences, Tabriz, Iran; 393 Institute for Prevention of Non-communicable Diseases, Qazvin University of Medical Sciences, Qazvin, Iran; 394 Health Services Management Department, Qazvin University of Medical Sciences, Qazvin, Iran; 395 Department of Pharmacology, Manipal Academy of Higher Education, Mangalore, India; 396 Family Medicine Department, Rafsanjan University of Medical Sciences, Rafsanjan, Iran; 397 Department of Forensic Medicine and Toxicology, All India Institute of Medical Sciences, Jodhpur, India; 398 Department of Epidemiology, Biostatistics and Clinical Research, All India Institute of Medical Sciences, New Delhi, India; 399 Social Determinants of Health Research Center, Tabriz University of Medical Sciences, Tabriz, Iran; 400 Department of Adult Health Nursing, Bahir Dar University, Bahir Dar, Ethiopia; 401 School of Nursing and Midwifery, Hawassa University, Hawassa, Ethiopia; 402 Department of Biomedical Sciences, Aksum University, Aksum, Ethiopia; 403 School of Midwifery, Hawassa University, Hawassa, Ethiopia; 404 International Research Center of Excellence, Institute of Human Virology Nigeria, Abuja, Nigeria; 405 Julius Centre for Health Sciences and Primary Care, Utrecht University, Utrecht, Netherlands; 406 Institute of Biological Chemistry and Nutrition, University Hohenheim, Stuttgart, Germany; 407 Open, Distance and eLearning Campus, University of Nairobi, Nairobi, Kenya; 408 Department of Midwifery, University of Gondar, Gondar, Ethiopia; 409 Department of Public Health, Debre Markos University, Debre Markos, Ethiopia; 410 School of Food and Agricultural Sciences, University of Management and Technology, Lahore, Pakistan; 411 Health Promotion Research Center, Zahedan University of Medical Sciences, Zahedan, Iran; 412 Department of Population Science, Jatiya Kabi Kazi Nazrul Islam University, Mymensingh, Bangladesh; 413 Epidemiology Department, Jazan University, Jazan, Saudi Arabia; 414 Department of Population Studies, International Institute for Population Sciences, Mumbai, India; 415 Faculty of Health and Wellbeing, Sheffield Hallam University, Sheffield, UK; 416 College of Arts and Sciences, Ohio University, Zanesville, OH USA; 417 National Hepatology and Tropical Medicine Research Institute, Cairo University, Cairo, Egypt; 418 Department of Medical Parasitology, Cairo University, Cairo, Egypt; 419 Department of Public Health, Imam Mohammad Ibn Saud Islamic University, Riyadh, Saudi Arabia; 420 Department of Health Policy and Management, Johns Hopkins University, Baltimore, MD USA; 421 Department of Public Health, New Mexico State University, Las Cruces, NM USA; 422 Department of Public Health, Kermanshah University of Medical Sciences, Kermanshah, Iran; 423 Big Data Department, National Health Insurance Service, Wonju, South Korea; 424 School of Traditional Chinese Medicine, Xiamen University Malaysia, Sepang, Malaysia; 425 Department of Nutrition, Simmons University, Boston, MA USA; 426 Department of Nursing and Health Promotion, Oslo Metropolitan University, Oslo, Norway; 427 School of Health Sciences, Kristiania University College, Oslo, Norway; 428 Global Community Health and Behavioral Sciences, Tulane University, New Orleans, LA USA; 429 Department of Environmental Health Engineering, Arak University of Medical Sciences, Arak, Iran; 430 Independent Consultant, Jakarta, Indonesia; 431 Department of Internal and Pulmonary Medicine, Sheri Kashmir Institute of Medical Sciences, Srinagar, India; 432 CIBERSAM, San Juan de Dios Sanitary Park, Sant Boi de Llobregat, Spain; 433 Catalan Institution for Research and Advanced Studies (ICREA), Barcelona, Spain; 434 Department of Anthropology, Panjab University, Chandigarh, India; 435 Department of Anesthesiology, Duke University, Durham, NC USA; 436 Department of Demography, University of Montreal, Montreal, QC Canada; 437 Department of Social and Preventive Medicine, University of Montreal, Montreal, QC Canada; 438 Department of Family and Community Health, University of Health and Allied Sciences, Ho, Ghana; 439 International Institute for Population Sciences, Mumbai, India; 440 Department of Psychiatry, University of Nairobi, Nairobi, Kenya; 441 Division of Psychology and Language Sciences, University College London, London, UK; 442 Faculty of Health and Life Sciences, Coventry University, Coventry, UK; 443 Department of Medicine, McMaster University, Hamilton, ON Canada; 444 Imperial College Business School, Imperial College London, London, UK; 445 Faculty of Public Health, University of Indonesia, Depok, Indonesia; 446 Nuffield Department of Population Health, University of Oxford, Oxford, UK; 447 National Institute for Health Research (NIHR) Oxford Biomedical Research Centre, Oxford, UK; 448 Department of Internal Medicine, Post Graduate Institute of Medical Education and Research, Chandigarh, India; 449 Department of Community and Family Medicine, University of Baghdad, Baghdad, Iraq; 450 Unit of Genetics and Public Health, Institute of Medical Sciences, Las Tablas, Panama; 451 Ministry of Health, Herrera, Panama; 452 Department of Medical Sciences, Uppsala University, Uppsala, Sweden; 453 Department of Clinical Chemistry and Pharmacology, Uppsala University Hospital, Uppsala, Sweden; 454 Department of Otorhinolaryngology, Father Muller Medical College, Mangalore, India; 455 School of Medicine, University of Maryland, Baltimore, MD USA; 456 Department of Clinical Sciences and Community Health, University of Milan, Milan, Italy; 457 National Institute of Nutrition, Indian Council of Medical Research, Hyderabad, India; 458 School of Nursing, Hong Kong Polytechnic University, Hong Kong, China; 459 School of Pharmacy, Monash University, Bandar Sunway, Malaysia; 460 School of Pharmacy, Taylor’s University Lakeside Campus, Subang Jaya, Malaysia; 461 Centre for Tropical Medicine and Global Health, University of Oxford, Oxford, UK; 462 Oxford University Clinical Research Unit, Wellcome Trust Asia Programme, Hanoi, Vietnam; 463 Department of Sociology, Shenzhen University, Shenzhen, China; 464 School of Public Health and Preventive Medicine, Monash University, Melbourne, VIC Australia; 465 Department of Systems, Populations, and Leadership, University of Michigan, Ann Arbor, MI USA; 466 Department of Nutrition, University of the Philippines Manila, Manila, Philippines; 467 Alliance for Improving Health Outcomes, Inc., Quezon City, Philippines; 468 Center for Integration of Data and Health Knowledge, Oswald Cruz Foundation (FIOCRUZ), Salvador, Brazil; 469 Centre for Global Mental Health (CGMH), London School of Hygiene & Tropical Medicine, London, UK; 470 Department of Biochemistry, BGS Global Institute of Medical Sciences, Bengaluru, India; 471 Radiology Department, Egypt Ministry of Health and Population, Mansoura, Egypt; 472 Ophthalmology Department, Ministry of Health & Population, Aswan, Egypt; 473 Department of Forensic Medicine & Toxicology, Mysore Medical College & Research Institute, Mysooru, India; 474 Department of Health & Family Welfare, Government of Karnataka, Bangalore, India; 475 Grants, Innovation and Product Development Unit, South African Medical Research Council, Cape Town, South Africa; 476 Department of Public Health, Urmia University of Medical Science, Urmia, Iran; 477 Department of Clinical Physiology, Tribhuvan University, Kathmandu, Nepal; 478 Department of Forensic Medicine, Rajiv Gandhi University of Health Sciences, Dharwad, India; 479 Department of Forensic Medicine, Shri Dharmasthala Manjunatheshwara University, Dharwad, India; 480 Clinical Research Development Center, Kermanshah University of Medical Sciences, Kermanshah, Iran; 481 Non-communicable Disease Research Center, Shiraz University of Medical Sciences, Shiraz, Iran; 482 Department of Maternal and Child Nursing and Public Health, Federal University of Minas Gerais, Belo Horizonte, Brazil; 483 Department of Epidemiology and Biostatistics, Tehran University of Medical Sciences, Tehran, Iran; 484 Department of Nursing, Majmaah University, Majmaah, Saudi Arabia; 485 Technological Institution Colegio Mayor de Bolívar (Institución Tecnológica Colegio Mayor de Bolívar), Cartagena, Colombia; 486 Campus Caucaia, Federal Institute of Education, Science and Technology of Ceará, Caucaia, Brazil; 487 Faculty of Health and Education, Botho University, Gaborone, Botswana; 488 Research Division, The George Institute for Global Health, New Delhi, India; 489 School of Medicine, University of New South Wales, Sydney, NSW Australia; 490 ICF International, DHS Program, Rockville, MD USA; 491 Department of Epidemiology, Adigrat University, Adigrat, Ethiopia; 492 Neurology Department, Janakpuri Super Specialty Hospital Society, New Delhi, India; 493 Department of Neurology, Govind Ballabh Institute of Medical Education and Research, New Delhi, India; 494 Tehran Heart Center, Tehran University of Medical Sciences, Tehran, Iran; 495 Nutrition Health Research Center, Iran University of Medical Sciences, Hamadan, Iran; 496 Department of Epidemiology and Biostatistics, University of California, San Francisco, San Francisco, CA USA; 497 Department of Public Health and Mortality Studies, International Institute for Population Sciences, Mumbai, India; 498 Department of Nutrition, University of Oslo, Oslo, Norway; 499 Department of Hematology and Immunohematology, University of Gondar, Gondar, Ethiopia; 500 Peru Country Office, United Nations Population Fund (UNFPA), Lima, Peru; 501 Forensic Medicine Division, Imam Abdulrahman Bin Faisal University, Dammam, Saudi Arabia; 502 Department of Midwifery, Adigrat University, Adigrat, Ethiopia; 503 Department of Reproductive Health and Population Studies, Bahir Dar University, Bahir Dar, Ethiopia; 504 Breast Surgery Unit, Helsinki University Hospital, Helsinki, Finland; 505 University of Helsinki, Helsinki, Finland; 506 Department of Nursing, Arba Minch University, Arba Minch, Ethiopia; 507 Department of Psychiatry, Mekelle University, Mekelle, Ethiopia; 508 Department of Propedeutics of Internal Diseases & Arterial Hypertension, Pomeranian Medical University, Szczecin, Poland; 509 Woman-Mother-Child Department, Lausanne University Hospital, Lausanne, Switzerland; 510 Global Institute of Public Health, Ananthapuri Hospitals and Research Institute, Trivandrum, India; 511 College of Applied Medical Sciences, Majmaah University, Riyadh, Saudi Arabia; 512 Internal Medicine Programme, Kyrgyz State Medical Academy, Bishkek, Kyrgyzstan; 513 Department of Atherosclerosis and Coronary Heart Disease, National Center of Cardiology and Internal Disease, Bishkek, Kyrgyzstan; 514 Research Center for Biochemistry and Nutrition in Metabolic Diseases, Kashan University of Medical Sciences, Kashan, Iran; 515 Department of Rehabilitation and Sports Medicine, Kermanshah University of Medical Sciences, Kermanshah, Iran; 516 Department of Surgical Oncology, All India Institute of Medical Sciences, Jodhpur, India; 517 Institute of Addiction Research (ISFF), Frankfurt University of Applied Sciences, Frankfurt, Germany; 518 Biotechnology Research Center, Tabriz University of Medical Sciences, Tabriz, Iran; 519 Molecular Medicine Research Center, Tabriz University of Medical Sciences, Tabriz, Iran; 520 Internal Medicine Department, King Saud University, Riyadh, Saudi Arabia; 521 Department of Forestry, Salahaddin University-Erbil, Erbil, Iraq; 522 Department of Medicine-Huddinge, Karolinska Institute, Stockholm, Sweden; 523 Department of Biostatistics, Hamadan University of Medical Sciences, Hamadan, Iran; 524 Department of Anatomical Sciences, Zanjan University of Medical Sciences, Zanjan, Iran; 525 Department of Epidemiology and Biostatistics, Shahrekord University of Medical Sciences, Shahrekord, Iran; 526 Department of Nursing, Mashhad University of Medical Sciences, Mashhad, Iran; 527 Department of Nursing, Adigrat University, Adigrat, Ethiopia; 528 Department of Biomolecular Sciences, University of Missippi, Oxford, MS USA; 529 Department of Pharmacy, Mizan-Tepi University, Mizan, Ethiopia; 530 School of Pharmacy, Haramaya University, Harar, Ethiopia; 531 Health Systems and Policy Research Unit, Ahmadu Bello University, Zaria, Nigeria; 532 Department of Public Health, Samara University, Semera, Ethiopia; 533 Pediatric Neurorehabilitation Research Center, University of Social Welfare and Rehabilitation Sciences, Tehran, Iran; 534 Clinical Epidemiology and Public Health Research Unit, Burlo Garofolo Institute for Maternal and Child Health, Trieste, Italy; 535 Computer, Electrical, and Mathematical Sciences and Engineering Division, King Abdullah University of Science and Technology, Thuwal, Saudi Arabia; 536 Department of Clinical Biochemistry, Babol University of Medical Sciences, Babol, Iran; 537 Department of Clinical Biochemistry, Tarbiat Modares University, Tehran, Iran; 538 Department of Health Policy, Management, and Economics, Tehran University of Medical Sciences, Tehran, Iran; 539 Department of Food Science, University of Campinas (Unicamp), Campinas, Brazil; 540 Department of Pediatrics and Child Health, Debre Berhan University, Debre Berhan, Ethiopia; 541 Department of Community Health Sciences, Fatima Memorial Hospital (FMH), Lahore, Pakistan; 542 School of Economics, University of Nairobi, Nairobi, Kenya; 543 Indian Institute of Public Health, Public Health Foundation of India, Hyderabad, India; 544 Department of Pediatric Medicine, The Children’s Hospital & The Institute of Child Health, Multan, Pakistan; 545 Department of Pediatrics & Pediatric Pulmonology, Institute of Mother & Child Care, Multan, Pakistan; 546 Department of Obstetrics and Gynecology, Ain Shams University, Cairo, Egypt; 547 Knowledge Translation and Utilization, Egyptian Center for Evidence Based Medicine, Cairo, Egypt; 548 Research and Analytics Department, Initiative for Financing Health and Human Development, Chennai, India; 549 Department of Research and Analytics, Bioinsilico Technologies, Chennai, India; 550 Department of Nephrology, Manipal Academy of Higher Education, Manipal, India; 551 Comprehensive Cancer Center, University of Alabama at Birmingham, Birmingham, AL USA; 552 Laboratory of Public Health Indicators Analysis and Health Digitalization, Moscow Institute of Physics and Technology, Dolgoprudny, Russia; 553 Experimental Surgery and Oncology Laboratory, Kursk State Medical University, Kursk, Russia; 554 Suraj Eye Institute, Nagpur, India; 555 Department for the Control of Disease, Epidemics, and Pandemics, Ministry of Public Health, Yaoundé, Cameroon; 556 Department of Public Health, University of Yaoundé I, Yaoundé, Cameroon; 557 Department of Pharmacy Practice, Imam Abdulrahman Bin Faisal University, Dammam, Saudi Arabia; 558 Discipline of Social & Administrative Pharmacy, University of Science Malaysia, Penang, Malaysia; 559 Department of Clinical Medicine, Federal University of Minas Gerais, Belo Horizonte, Brazil; 560 Clinical Hospital, Federal University of Minas Gerais, Belo Horizonte, Brazil; 561 Manipal Institute of Management, Manipal Academy of Higher Education, Manipal, India; 562 Department of Pediatrics, Arak University of Medical Sciences, Arak, Iran; 563 Disease Control and Environmental Health, Makerere University, Kampala, Uganda; 564 Department of General Surgery, Carol Davila University of Medicine and Pharmacy, Bucharest, Romania; 565 Department of General Surgery, Emergency Hospital of Bucharest, Bucharest, Romania; 566 Department of Anatomy and Embryology, Carol Davila University of Medicine and Pharmacy, Bucharest, Romania; 567 Cardio-Aid, Bucharest, Romania; 568 School of Nursing, University of Gondar, Gondar, Ethiopia; 569 School of Health Sciences, Surgical Nursing, Bahir Dar University, Gondar, Ethiopia; 570 Department of Biological Sciences, University of Embu, Embu, Kenya; 571 Institute for Global Health Innovations, Duy Tan University, Hanoi, Vietnam; 572 Faculty of Pharmacy, Duy Tan University, Da Nang, Vietnam; 573 Institute for Mental Health and Policy, Centre for Addiction and Mental Health, Toronto, ON Canada; 574 Department of Clinical Epidemiology, Institute for Clinical Evaluative Sciences, Ottowa, ON Canada; 575 Hormozgan University of Medical Sciences, Bandar Abbas, Iran; 576 School of Public Health and Family Medicine, University of Cape Town, Cape Town, South Africa; 577 Centre for Heart Rhythm Disorders, University of Adelaide, Adelaide, WC Australia; 578 Unit of Microbiology and Public Health, Institute of Medical Sciences, Las Tablas, Panama; 579 Department of Public Health, Ministry of Health, Herrera, Panama; 580 Department of Pediatrics, National Hospital Abuja, Abuja, Nigeria; 581 Department of International Public Health, University of Liverpool, Liverpool, UK; 582 Department of Public Health, CQ University, Melbourne, VIC Australia; 583 Administrative and Economic Sciences Department, University of Bucharest, Bucharest, Romania; 584 Translational Health Research Institute, Western Sydney University, Sydney, NSW Australia; 585 Department of Obstetrics and Gynecology, University of Ibadan, Ibadan, Nigeria; 586 Department of Preventive Medicine, Kyung Hee University, Dongdaemun-gu, South Korea; 587 Department of Rural Development and Agribusiness, Gulu University, Gulu, Uganda; 588 Gorgan Congenital Malformations Research Center, Golestan University of Medical Sciences, Gorgan, Iran; 589 Department of Psychiatry and Behavioural Neurosciences, McMaster University, Hamilton, ON Canada; 590 Department of Psychiatry, University of Lagos, Lagos, Nigeria; 591 Centre for Healthy Start Initiative, Lagos, Nigeria; 592 Diplomacy and Public Relations Department, University of Human Development, Sulaimaniyah, Iraq; 593 Department of Anatomic Pathology, Ekiti State University Teaching Hospital, Ado Ekiti, Nigeria; 594 Department of Pharmacology and Therapeutics, University of Nigeria Nsukka, Enugu, Nigeria; 595 Department of Medicine, Autonomous University of Madrid, Madrid, Spain; 596 Department of Nephrology and Hypertension, The Institute for Health Research Foundation, Jiménez Díaz University Hospital, Madrid, Spain; 597 Center for Population Health Research, National Institute of Public Health, Cuernavaca, Mexico; 598 Department of Project Management, National Research University Higher School of Economics, Moscow, Russia; 599 Department of Medicine, University of Ibadan, Ibadan, Nigeria; 600 Department of Medicine, University College Hospital, Ibadan, Ibadan, Nigeria; 601 Department of Respiratory Medicine, Jagadguru Sri Shivarathreeswara Academy of Health Education and Research, Mysore, India; 602 Department of Forensic Medicine, Manipal Academy of Higher Education, Mangalore, India; 603 Department of Parasitology and Mycology, Shiraz University of Medical Sciences, Shiraz, Iran; 604 Department of Health Metrics, Center for Health Outcomes & Evaluation, Bucharest, Romania; 605 Department of Research, Public Health Foundation of India, Gurugram, India; 606 Somnogen Canada Inc, Toronto, ON Canada; 607 National Institute of Health Research and Development, Ministry of Health, Jakarta, Indonesia; 608 Division of General Internal Medicine, University of Pittsburgh Medical Center, Pittsburgh, PA USA; 609 Department of Poverty, Gender and Youth, Population Council, New Delhi, India; 610 Department of Neurology and Public Health, Icahn School of Medicine at Mount Sinai, New York, NY USA; 611 Department of Pediatrics, RD Gardi Medical College, Ujjain, India; 612 Global Public Health-Health Systems and Policy (HSP): Medicines Focusing Antibiotics, Karolinska Institute, Stockholm, Sweden; 613 Department of Pediatrics, University of Melbourne, Melbourne, VIC Australia; 614 Population Health Theme, Murdoch Children’s Research Institute, Melbourne, VIC Australia; 615 Department of Physiology, Iran University of Medical Sciences, Tehran, Iran; 616 Physiology Research Center, Iran University of Medical Sciences, Tehran, Iran; 617 Department of Orthopedics, Yenepoya Medical College, Mangalore, India; 618 Department of Biochemistry and Dietetics, Tabriz University of Medical Sciences, Tabriz, Iran; 619 HIV and Mental Health Department, Integrated Development Foundation Nepal, Kathmandu, Nepal; 620 University Medical Center Groningen, University of Groningen, Groningen, Netherlands; 621 School of Economics and Business, University of Groningen, Groningen, Netherlands; 622 Department of Pharmacology, Imam Abdulrahman Bin Faisal University, Dammam, Saudi Arabia; 623 Clinical Research Development Centre, Taleghani and Imam Ali Hospital, Kermanshah University of Medical Sciences, Kermanshah, Iran; 624 Department of Nutrition and Food Sciences, Maragheh University of Medical Sciences, Maragheh, Iran; 625 Dietary Supplements and Probiotic Research Center, Alborz University of Medical Sciences, Karaj, Iran; 626 Clinical Research Center, Valle del Lili Foundation (Centro de Investigaciones Clinicas, Fundación Valle del Lili), Cali, Colombia; 627 PROESA, ICESI University (Centro PROESA, Universidad ICESI), Cali, Colombia; 628 Health Sciences Department, Muhammadiyah University of Surakarta, Sukoharjo, Indonesia; 629 Department of Chemistry, Sharif University of Technology, Tehran, Iran; 630 Department of Medicine, University of Alberta, Edmonton, AB Canada; 631 Thalassemia and Hemoglobinopathy Research Center, Ahvaz Jundishapur University of Medical Sciences, Ahvaz, Iran; 632 Metabolomics and Genomics Research Center, Tehran University of Medical Sciences, Tehran, Iran; 633 Department of Community Medicine, Maharishi Markandeshwar Medical College & Hospital, Solan, India; 634 School of Nursing and Healthcare Professions, Federation University Australia, Berwick, VIC Australia; 635 Department of Computer Science, Khazar University, Baku, Azerbaijan; 636 Society for Health and Demographic Surveillance, Suri, India; 637 Department of Economics, University of Göttingen, Göttingen, Germany; 638 Department of Surgery, The University of Texas Health Science Center at San Antonio, San Antonio, TX USA; 639 Department of Cardiology, Emory University, Atlanta, GA USA; 640 Department of Pharmacology, Shahid Beheshti University of Medical Sciences, Tehran, Iran; 641 Research Department, Policy Research Institute, Kathmandu, Nepal; 642 Health and Public Policy Department, Global Center for Research and Development, Kathmandu, Nepal; 643 Department of Oral Pathology, Srinivas Institute of Dental Sciences, Mangalore, India; 644 Department of Infectious Disease, Manipal Academy of Higher Education, Mangalore, India; 645 Department of Forensic Medicine and Toxicology, Manipal Academy of Higher Education, Mangalore, India; 646 School of Health, Medical and Applied Sciences, CQ University, Sydney, NSW Australia; 647 River Region Cardiology Associates, Montgomery, WV USA; 648 Department of Computer Science, Boston University, Boston, MA USA; 649 Department of Health Information Management, Manipal Academy of Higher Education, Manipal, India; 650 Manipal Academy of Higher Education, Manipal, India; 651 Network of Immunity in Infection, Malignancy and Autoimmunity (NIIMA), Universal Scientific Education and Research Network (USERN), Tehran, Iran; 652 Department of Infectious Diseases, University of Copenhagen, Copenhagen, Denmark; 653 Cardiovascular Diseases Research Center, Birjand University of Medical Sciences, Birjand, Iran; 654 Department of Chemical Sciences, University of Porto, Oporto, Portugal; 655 Epidemiology Research Unit Institute of Public Health (EPIUnit-ISPUP), University of Porto, Porto, Portugal; 656 Department of Surgery, University of Minnesota, Minneapolis, MN USA; 657 Department of Surgery, University Teaching Hospital of Kigali, Kigali, Rwanda; 658 Department of Clinical Research, Federal University of Uberlândia, Uberlândia, Brazil; 659 School of Medicine, Gonabad University of Medical Sciences, Gonabad, Iran; 660 Malaria Atlas Project, University of Oxford, Oxford, UK; 661 Department of Health Statistics, National Institute for Medical Research, Dar es Salaam, Tanzania; 662 Department of Internal Medicine, University of Botswana, Gaborone, Botswana; 663 Department of Epidemiology, Shahid Beheshti University of Medical Sciences, Tehran, Iran; 664 Department of Psychiatry, All India Institute of Medical Sciences, New Delhi, India; 665 Halal Research Center of IRI, Food and Drug Administration of the Islamic Republic of Iran, Tehran, Iran; 666 Neurogenic Inflammation Research Center, Mashhad University of Medical Sciences, Mashhad, Iran; 667 Department of Phytochemistry, Soran University, Soran, Iraq; 668 Department of Nutrition, Cihan University-Erbil, Erbil, Iraq; 669 Department of Microbiology, Central University of Punjab, Bathinda, India; 670 Public Health and Community Medicine Department, Cairo University, Giza, Egypt; 671 Drug Applied Research Center, Tabriz University of Medical Sciences, Tabriz, Iran; 672 Center for Clinical and Epidemiological Research, Hospital Universitário, University of São Paulo, São Paulo, Brazil; 673 School of Public Health and Health Management, University of Belgrade, Belgrade, Serbia; 674 Department of Community Medicine, PSG Institute of Medical Sciences and Research, Coimbatore, India; 675 PSG-FAIMER South Asia Regional Institute, Coimbatore, India; 676 Isfahan Cardiovascular Research Institute, Isfahan University of Medical Sciences, Isfahan, Iran; 677 School of Population and Public Health, University of British Columbia, Vancouver, BC Canada; 678 Faculty of Infectious and Tropical Diseases, London School of Hygiene & Tropical Medicine, London, UK; 679 Colorectal Research Center, Iran University of Medical Sciences, Tehran, Iran; 680 Department of Geriatrics and Long Term Care, Hamad Medical Corporation, Doha, Qatar; 681 Faculty of Health & Social Sciences, Bournemouth University, Bournemouth, UK; 682 Population Health Research Institute, McMaster University, Hamilton, ON Canada; 683 Department of Epidemiology, Indian Institute of Public Health, Gandhinagar, India; 684 Department of Psychology, University of Alabama at Birmingham, Birmingham, AL USA; 685 Department of Food Science and Nutrition, Jigjiga University, Jigjiga, Ethiopia; 686 Department of Computational Biology, Indraprastha Institute of Information Technology, Delhi, India; 687 Emergency Department, Manian Medical Centre, Erode, India; 688 National Heart, Lung, and Blood Institute, National Institutes of Health, Rockville, MD USA; 689 Center for Biomedical Information Technology, Shenzhen Institutes of Advanced Technology, Shenzhen, China; 690 Department of Radiology and Radiological Science, Johns Hopkins University, Baltimore, MD USA; 691 Department of Radiology and Interventional Neuroradiology, Isfahan University of Medical Sciences, Isfahan, Iran; 692 Health Policy Research Center, Shiraz University of Medical Sciences, Shiraz, Iran; 693 Department of Internal Medicine, Ziauddin University, Karachi, Pakistan; 694 Independent Consultant, Karachi, Pakistan; 695 School of Public Health, Dilla University, Dilla, Ethiopia; 696 Neurology Department, Ain Shams University, Cairo, Egypt; 697 Department of Clinical Research, University of Southern Denmark, Odense, Denmark; 698 School of Medicine, Alborz University of Medical Sciences, Karaj, Iran; 699 Faculty of Caring Science, Work Life, and Social Welfare, University of Borås, Borås, Sweden; 700 Department of Community Medicine, BLDE University, Vijayapur, India; 701 Golestan Research Center of Gastroenterology and Hepatology (GRCGH), Golestan University of Medical Sciences, Gorgan, Iran; 702 Centre for Medical Informatics, University of Edinburgh, Edinburgh, UK; 703 Division of General Internal Medicine, Harvard University, Boston, MA USA; 704 Health Information Management, Iran University of Medical Sciences, Tehran, Iran; 705 Department of Community Medicine, Manipal Academy of Higher Education, Manipal, India; 706 Department of Obstetrics and Gynaecology, Manipal Academy of Higher Education, Mangalore, India; 707 National Institute of Infectious Diseases, Tokyo, Japan; 708 College of Medicine, Yonsei University, Seoul, South Korea; 709 Finnish Institute of Occupational Health, Helsinki, Finland; 710 Cancer Research Institute, Tehran University of Medical Sciences, Tehran, Iran; 711 Cancer Biology Research Center, Tehran University of Medical Sciences, Tehran, Iran; 712 Clinical Immunology and Hematology, Sofiamed University Hospital, Sofia, Bulgaria; 713 Department of Genetics, Sofia University ‘St. Kliment Ohridiski’, Sofia, Bulgaria; 714 Department of Health Education and Health Promotion, Kermanshah University of Medical Sciences, Kermanshah, Iran; 715 School of Health, University of Technology Sydney, Sydney, NSW Australia; 716 Department of Hematology-Oncology, Baystate Medical Center, Springfield, MA USA; 717 Department of Medicine, Dow University of Health Sciences, Karachi, Pakistan; 718 School of Public Health & Zoonoses, Guru Angad Dev Veterinary & Animal Sciences University, Ludhiana, India; 719 School of Veterinary Science, University of Sydney, Sydney, NSW Australia; 720 Division of Environmental Monitoring & Exposure Assessment (Water & Soil), National Institute for Research in Environmental Health, Bhopal, India; 721 Department of Midwifery, Haramaya University, Harar, Ethiopia; 722 Department No. 16, Moscow Research and Practical Centre on Addictions, Moscow, Russia; 723 Therapeutic Department, Balashiha Central Hospital, Balashikha, Russia; 724 Department of Vascular and Endovascular Surgery, Kermanshah University of Medical Sciences, Kermanshah, Iran; 725 Nursing Care Research Center, Semnan University of Medical Sciences, Semnan, Iran; 726 Division of Community Medicine, International Medical University, Kuala Lumpur, Malaysia; 727 Nursing Department, Muhammadiyah University of Surakarta, Surakarta, Indonesia; 728 Pediatric Services, King Hussein Cancer Center, Amman, Jordan; 729 Pediatrics, University of Jordan, Amman, Jordan; 730 Department of Medicine, University of Valencia, Valencia, Spain; 731 Carlos III Health Institute, Biomedical Research Networking Center for Mental Health Network (CiberSAM), Madrid, Spain; 732 Cancer Control Center, Osaka International Cancer Institute, Osaka, Japan; 733 Department of Pharmacy, Arbaminch College of Health Sciences, Arba Minch, Ethiopia; 734 Department of Biomedical Sciences, Arba Minch University, Arba Minch, Ethiopia; 735 Research Center for Molecular Medicine, Hamadan University of Medical Sciences, Hamadan, Iran; 736 Department of Medical Laboratory Science, Addis Ababa University, Addis Ababa, Ethiopia; 737 Department of Population Science and Human Resource Development, University of Rajshahi, Rajshahi, Bangladesh; 738 Department of Cell Therapy and Applied Genomics, King Hussein Cancer Center, Amman, Jordan; 739 Department of Clinical Pharmacy, University of Gondar, Gondar, Ethiopia; 740 Department of Community and Family Medicine, Iran University of Medical Sciences, Tehran, Iran; 741 Pediatric Intensive Care Unit, King Saud University, Riyadh, Saudi Arabia; 742 Department of Public Health and Community Medicine, Central University of Kerala, Kasaragod, India; 743 Department of Endocrinology, Diabetes and Metabolism, Christian Medical College and Hospital (CMC), Vellore, India; 744 Department of Psychiatry Nursing, Ambo University, Ambo, Ethiopia; 745 K.A. Timiryazev Institute of Plant Physiology, Russian Academy of Sciences, Moscow, Russia; 746 Department of Pathology and Legal Medicine, University of São Paulo, Ribeirão Preto, Brazil; 747 Modestum LTD, London, UK; 748 Molecular Medicine and Pathology, University of Auckland, Auckland, New Zealand; 749 Clinical Hematology and Toxicology, Maurice Wilkins Centre, Auckland, New Zealand; 750 Department of Health Economics, Hanoi Medical University, Hanoi, Vietnam; 751 Department of Zoology, Arabian Gulf University, Churu, India; 752 Department of Community Medicine, All India Institute of Medical Sciences, Nagpur, India; 753 Faculty of Geo-Information Science and Earth Observation, University of Twente, Enschede, Netherlands; 754 School of Health and Rehabilitation Sciences, The University of Queensland, Brisbane, QLD Australia; 755 Multidisciplinary Department, National University of Medical Sciences (NUMS), Rawalpindi, Pakistan; 756 Department of Community Medicine, Alex Ekwueme Federal University Teaching Hospital Abakaliki, Abakaliki, Nigeria; 757 Department of Medical Microbiology/Parasitology, Ebonyi State University, Abakaliki, Nigeria; 758 Kasturba Medical College, Manipal Academy of Higher Education, Mangalore, India; 759 Amity Institute of Biotechnology, Amity University Rajasthan, Jaipur, India; 760 Department of Internal Medicine, Dow University of Health Sciences, Karachi, Pakistan; 761 Department of Neurology, Rafsanjan University of Medical Sciences, Rafsanjan, Iran; 762 Non-communicable Diseases Research Center, Rafsanjan University of Medical Sciences, Rafsanjan, Iran; 763 Clinical Cancer Research Center, Milad General Hospital, Tehran, Iran; 764 Department of Microbiology, Islamic Azad University, Tehran, Iran; 765 Argentine Society of Medicine, Buenos Aires, Argentina; 766 Velez Sarsfield Hospital, Buenos Aires, Argentina; 767 Department of Community Medicine and Family Medicine, All India Institute of Medical Sciences, Bathinda, India; 768 Department of Medical and Surgical Sciences, University of Bologna, Bologna, Italy; 769 Occupational Health Unit, Sant’Orsola Malpighi Hospital, Bologna, Italy; 770 Faculty of Information Technology, Ho Chi Minh City University of Technology (HUTECH), Ho Chi Minh City, Vietnam; 771 Population Dynamics and Sexual and Reproductive Health, African Population and Health Research Center, Nairobi, Kenya; 772 Foundation University Medical College, Foundation University Islamabad, Islamabad, Pakistan; 773 Department of Epidemiology and Biostatistics, Wuhan University, Wuhan, China; 774 Research School of Population Health, Australian National University, Canberra, ACT Australia; 775 Demographic Change and Aging Research Area, Federal Institute for Population Research, Wiesbaden, Germany; 776 Department of Physical Therapy, Naresuan University, Phitsanulok, Thailand; 777 Department of Community Medicine, Rajarata University of Sri Lanka, Anuradhapura, Sri Lanka; 778 Department of Orthopaedics, Wenzhou Medical University, Wenzhou, China; 779 Global Health Research Center, Duke Kunshan University, Kunshan, China; 780 Duke Global Health Institute, Duke University, Durham, NC USA; 781 Department of Behavior and Operation Management, Beijing Advanced Innovation Center for Big Data-based Precision Medicine, Beijing, China; 782 Psychology Department, University of Sheffield, Sheffield, UK; 783 Department of Diabetes and Metabolic Diseases, University of Tokyo, Tokyo, Japan; 784 School of International Development and Global Studies, University of Ottawa, Ottawa, ON Canada; 785 The George Institute for Global Health, University of Oxford, Oxford, UK; 786 Health Services Management Research Center, Kerman University of Medical Sciences, Kerman, Iran; 787 Department of Health Management, Policy, and Economics, Kerman University of Medical Sciences, Kerman, Iran; 788 Centre for Suicide Research and Prevention, University of Hong Kong, Hong Kong, China; 789 Department of Social Work and Social Administration, University of Hong Kong, Hong Kong, China; 790 Hubert Department of Global Health, Emory University, Atlanta, GA USA; 791 Department of Environmental Health, Mazandaran University of Medical Sciences, Sari, Iran; 792 Injury Prevention and Safety Promotion Research Center, Shahid Beheshti University of Medical Sciences, Tehran, Iran; 793 School of Public Health and Management, Hubei University of Medicine, Shiyan, China; 794 Department of Obstetrics and Gynaecology, Fazaia Medical College, Islamabad, Pakistan; 795 Department of Obstetrics and Gynaecology, Air University, Islamabad, Pakistan; 796 Department of Pharmaceutics, Dow University of Health Sciences, Karachi, Pakistan; 797 Department of Medicine, University Ferhat Abbas of Setif, Sétif, Algeria; 798 Institute for Immunological Research, University of Cartagena, Cartagena, Colombia; 799 The School of Clinical Sciences at Monash Health, Monash University, Melbourne, VIC Australia; 800 Student Research Committee, Babol University of Medical Sciences, Babol, Iran; 801 Department of Radiology, Children’s Hospital of Philadelphia, Philadelphia, PA USA; 802 Laboratory of Genetics and Genomics, Moscow Research and Practical Centre on Addictions, Moscow, Russia; 803 Addictology Department, Russian Medical Academy of Continuous Professional Education, Moscow, Russia; 804 Pediatrics Department, Russian Medical Academy of Continuous Professional Education, Moscow, Russia; 805 Department of Psychiatric Nursing, Haramaya University, Harar, Ethiopia; 806 School of Pharmacy, Mekelle University, Mekelle, Ethiopia; 807 School of Public Health, Wuhan University of Science and Technology, Wuhan, China; 808 Hubei Province Key Laboratory of Occupational Hazard Identification and Control, Wuhan University of Science and Technology, Wuhan, China; 809 Department of Epidemiology, Human Genetics, and Environmental Sciences, The University of Texas Health Science Center at Houston, Houston, TX USA

**Keywords:** Risk factors, Diseases

## Abstract

Anemia is a globally widespread condition in women and is associated with reduced economic productivity and increased mortality worldwide. Here we map annual 2000–2018 geospatial estimates of anemia prevalence in women of reproductive age (15–49 years) across 82 low- and middle-income countries (LMICs), stratify anemia by severity and aggregate results to policy-relevant administrative and national levels. Additionally, we provide subnational disparity analyses to provide a comprehensive overview of anemia prevalence inequalities within these countries and predict progress toward the World Health Organization’s Global Nutrition Target (WHO GNT) to reduce anemia by half by 2030. Our results demonstrate widespread moderate improvements in overall anemia prevalence but identify only three LMICs with a high probability of achieving the WHO GNT by 2030 at a national scale, and no LMIC is expected to achieve the target in all their subnational administrative units. Our maps show where large within-country disparities occur, as well as areas likely to fall short of the WHO GNT, offering precision public health tools so that adequate resource allocation and subsequent interventions can be targeted to the most vulnerable populations.

## Abstract (web summary)

Geospatial estimates of the prevalence of anemia in women of reproductive age across 82 low-income and middle-income countries reveals considerable heterogeneity and inequality at national and subnational levels, with few countries on track to meet the WHO Global Nutrition Targets by 2030.

## Main

Anemia occurs when the number of healthy red blood cells is insufficient to meet the body’s physiological needs for oxygen delivery to the brain, heart, muscles and other vital tissues. Hemoglobin is the primary oxygen-carrying molecule within red blood cells, so anemia is most typically measured in terms of hemoglobin content of the blood rather than red blood cell volume^1,2^. Anemia can reduce cognitive and physical capacities and is associated with reduced economic productivity^3,4^ and increased morbidity and all-cause mortality^5^. Maternal iron deficiency can lead to adverse pregnancy and newborn outcomes, including stillbirth, low birth weight and infant mortality^6^, and anemia in pregnancy has been suggested as a potential marker of increased risk of major hemorrhage^7^ and a risk factor for maternal death^8^.

Causes of anemia can be divided into three non-mutually exclusive pathways: blood loss, increased red blood cell destruction and inadequate red blood cell production. Blood loss can be acute due to events such as injuries, maternal hemorrhage or surgery, or it can be chronic, due to conditions such as gastrointestinal disorders, helminthic infections, bleeding disorders or abnormal uterine bleeding^9,10^. Increased red blood cell destruction happens either as a consequence of abnormal red blood cell structure, such as in thalassaemia or sickle cell disease, or because of external mechanical, immune or infectious factors^11^. Inadequate production of red blood cells can happen when the bone marrow itself is depressed, such as in HIV^12^ or some malignancies; because there are hormonal imbalances, such as with chronic inflammation;^13^ or due to increased demand (such as during pregnancy), nutrient malabsorption or inadequate supply of red blood cell building blocks, such as protein, iron, vitamin A^14^, folate or vitamin B-12 (ref. ^15^) Iron deficiency is often thought of as the most common cause of anemia, which is true but also misleading, because absolute and/or functional iron deficiency can arise as a consequence of any of the three pathways and, therefore, as a consequence of multiple different causes. Women of reproductive age (WRA; ages 15–49 years) are at particularly increased risk of iron deficiency and, therefore, anemia, compared to men, due to physiological changes such as menstruation (blood loss pathway), pregnancy (inadequate production pathway due to increased demand) and bleeding in childbirth^16,17^. Additionally, unequal household food allocation can make WRA vulnerable to anemia as they might not have access to iron-rich foods^17^.

Anemia continues to affect millions of women worldwide and remains concentrated in LMICs as defined by the Global Burden of Disease (GBD) Socio-Demographic Index (SDI)^18^. In 2019, 30.1% of WRA were estimated to have anemia globally, with wide geographical variation^18^, and dietary iron deficiency was among the highest-ranking conditions in both prevalence and years lived with disability (YLDs) among WRA in LMICs^19^. The WHO has set a GNT to reduce anemia in WRA by 50% by 2025 (refs. ^2,20^); this target and other related WHO GNTs have since been extended to 2030 (ref. ^21^). In October 2019, the percentage of WRA with anemia was officially added as an indicator to track progress toward the Sustainable Development Goal (SDG) 2.2 to end all forms of malnutrition by 2030 (refs. ^22,23^). Although the WHO provides national-level anemia estimates and tracking tools, available reports do not show the subnational heterogeneity needed to inform within-country planning, annual changes to track progress or anemia severity stratifications^20,24^. Maps of comparable estimates across space and time at policy-relevant administrative levels are vital to identify the most vulnerable populations, track progress toward international anemia goals and provide decision-makers and policy-makers with tools to aid targeted interventions.

This study is part of a series using high-spatial-resolution estimates to map progress toward the WHO GNTs^25–27^. To perform this study, we compiled an extensive geo-positioned dataset from 218 surveys representing over 3 million women. Using Bayesian model-based geostatistics and the assumption that locations with similar socioeconomic and environmental patterns and proximity in time and space would have similar anemia levels, we produced estimates for all areas across 82 LMICs, even where data were sparse. The geospatial nature of our estimates also allows for the flexibility to aggregate to different (and sometimes changing) boundaries and catchment areas over the observation period.

Here we present annual geospatial estimates from 2000 to 2018 of prevalence and absolute counts of anemia of WRA (non-pregnant and pregnant combined), stratified by severity and aggregated to first-level (for example, provinces) and second-level (for example, districts) administrative units and national levels across 82 LMICs. Overall anemia was defined as <12 g dl^−1^ for non-pregnant WRA and <11 g dl^−1^ for pregnant WRA^28^. Anemia severity categories are defined by the WHO: mild anemia (11.0–11.9 g dl^−1^ for non-pregnant WRA; 10.0–10.9 g dl^−1^ for pregnant WRA), moderate anemia (8.0–10.9 g dl^−1^ for non-pregnant WRA; 7.0–9.9 g dl^−1^for pregnant WRA) and severe anemia (<8.0 g dl^−1^ for non-pregnant WRA; <7.0 g dl^−1^ for pregnant WRA). We also discuss our results in light of public health problem thresholds: no public health problem (<5% overall anemia prevalence), low public health problem (5–19.9% overall anemia prevalence), medium public health problem (20–39.9% overall anemia prevalence) and high public health problem (≥40% overall anemia prevalence)^28^. We show annualized rates of change (AROCs) between 2000 and 2018 and estimate the probability of achieving the WHO GNT by 2025 and 2030 based on recent trends. Additionally, we provide subnational disparity analyses. These estimates can aid in focusing attention on exemplars of progress, highlighting subnational inequalities and identifying locations requiring further investments. The full suite of outputs from the analysis are publicly available on the Global Health Data Exchange (http://ghdx.healthdata.org/record/ihme-data/global-anemia-prevalence-geospatial-estimates-2000-2019) and via our interactive data visualization tool (https://vizhub.healthdata.org/lbd/aneamia).

## Results

### Prevalence and trends of overall anemia

**Fig. 1:**
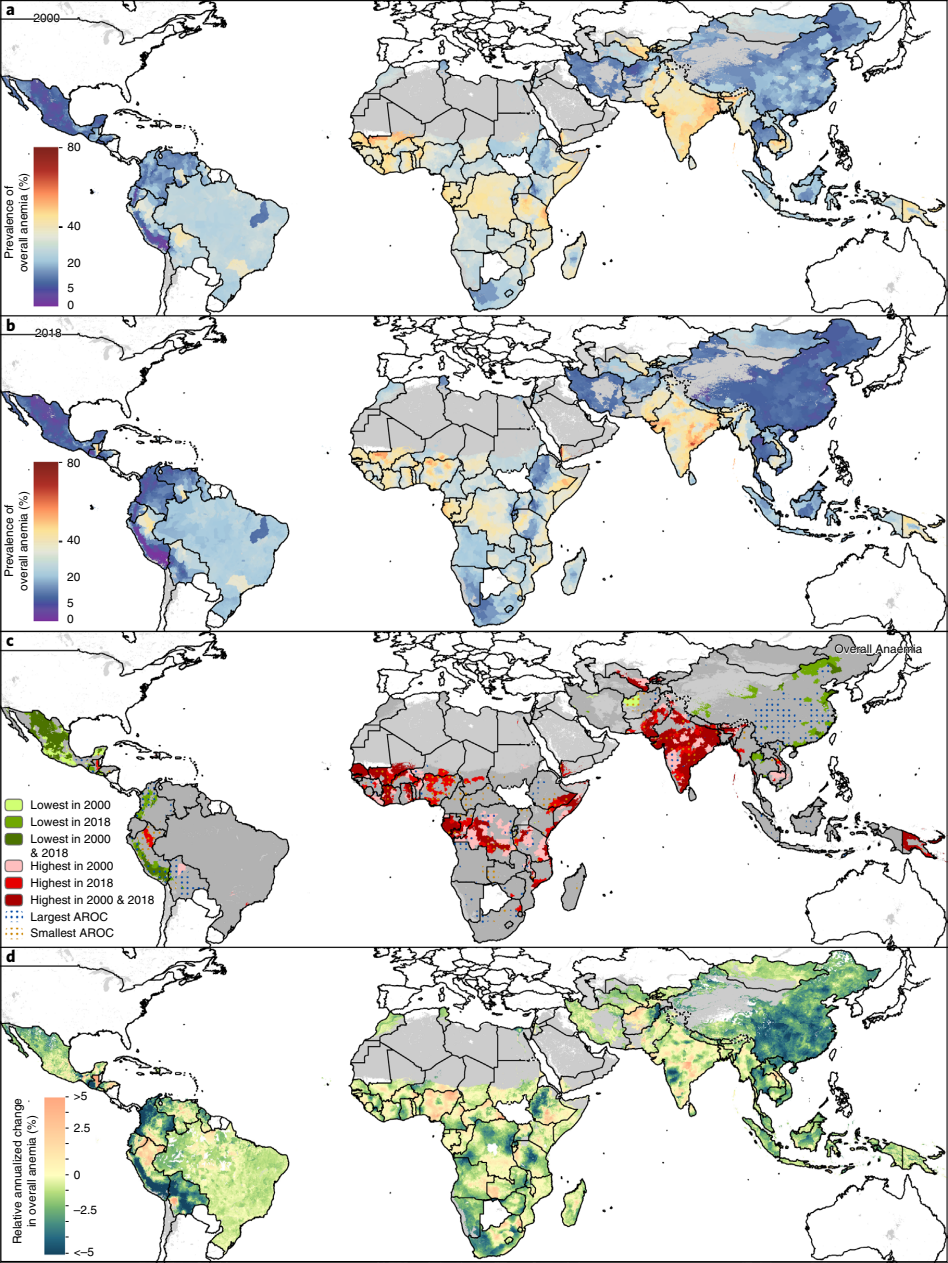
Prevalence and AROCs of overall anemia in WRA (2000–2018). **a**, **b**, Prevalence of overall anemia among WRA (ages 15–49) at the second administrative unit (for example, district) level in 2000 (**a**) and 2018 (**b**). **c**, Overlapping population-weighted highest and lowest (10th and 90th deciles) prevalence and AROCs between 2000 and 2018. Largest AROC indicates where largest decreases in overall anemia prevalence from 2000 to 2018 occurred, whereas smallest AROC indicates where the largest increases (or smallest decreases or stagnation) in overall anemia prevalence from 2000 to 2018 occurred. **d**, Weighted annualized percentage of change of overall anemia prevalence in WRA from 2000 to 2018. Maps reflect administrative boundaries, land cover, lakes and population; gray-colored grid cells had fewer than ten people per 1 × 1-km grid cell and were classified as ‘barren or sparsely vegetated’, whereas white-colored grid cells were not included in this analysis^42–47^.

The prevalence of overall anemia among WRA varied broadly across LMICs (Fig. 1a,b). In 2018, anemia prevalence was highest in West African, Middle Eastern and South Asian countries, including Gambia (50.3% (95% uncertainty interval: 43.3–57.5)), Senegal (47.3% (43.4–50.1)), Mali (47.6% (45.8–49.4)), Yemen (57.4% (50.9–63.8)) and India (49.9% (47.2–52.4)). The lowest national-level anemia prevalence in 2018 was found in Central America and the Caribbean, Andean South America and East Asia, including El Salvador (8.2% (3.6–16.1)), Colombia (9.2% (4.5–17.0)), Mexico (10.4% (7.3–15.3)) and China (11.1% (9.1–13.1)).

Gradual declines on a global scale indicate that little progress was seen in reducing anemia on a more local scale. Across the 82 LMICs, overall anemia among WRA decreased from 35.6% (25.7–46.9) in 2000 to 31.6% (25.2–39.1) in 2018. High levels of anemia remained widespread in 2018, with just over half (56.1%; 46 of 82) of LMICs with 20–39.9% prevalence of mean national-level overall anemia in 2018. On a subnational scale, 80 (97.6%) LMICs had at least one second administrative-level unit (hereafter ‘district’), and 38 (46.3%) LMICs had a majority of districts with 20–39.9% mean overall anemia prevalence. Over a quarter of LMICs (25.6%; 21 LMICs) had >40% mean national-level anemia in 2018, whereas 76 (92.7%) had at least one district, and 22 (26.8%) LMICs had most of their districts, with >40% mean overall anemia prevalence.

Anemia was at unacceptable levels (>5% prevalence)^28^ in 99.7% (21,868 of 21,917) of districts across LMICs in 2000 and 98.9% (21,686 of 21,917) in 2018. (Fig. 1a,b). In 2000, 30.7% (6,725 of 21,917), 48.9% (10,726 of 21,917) and 20.1% (4,417 of 21,917) of subnational districts had low (5–19.9%), medium (20–39.9%) and high (≥40%) public health threat levels of anemia among WRA^28^, respectively (Extended Data Table 1). Global shifts led to 37.7% (8,273 of 21,917), 43.5% (9,523 of 21,917) and 17.7% (3,881 of 21,917) of districts having low, medium and high public health problem levels in anemia prevalence among WRA, respectively, in 2018. Only two countries (Peru and Ecuador) had districts that maintained levels below 5% prevalence of overall anemia in both 2000 and 2018. In Peru, 36 of 195 (19.5%) districts had overall anemia prevalence levels <5% in both 2000 and 2018, such as in San Román (Puno) in the south (2.3% (1.1–4.3) in 2000; 0.4% (0.2–0.7) in 2018); in Ecuador, only one of 223 (0.4%) districts mean estimates achieved <5% prevalence in both years: the centrally located Cevallos (Tungurahua) (4.4% (1.0–12.5) in 2000; 4.6% (1.2–11.4) in 2018). Although Mexico had 16 districts and Iran had two districts below public health problem levels (<5%) in 2000, these districts exceeded 5% overall anemia prevalence in 2018. In 2018, only nine LMICs had at least one district with no public health problem in anemia (<5%), including Bolivia (two of 114 districts), Colombia (13 of 1,065 districts), Ecuador (one of 223 districts), El Salvador (six of 266 districts), Guatemala (87 of 354 districts), Mexico (25 of 2,454 districts), Thailand (one of 928 districts) and Uganda (two of 203 districts). Peru has seen great success reducing childhood stunting^29^, in part due to its targeted focus on those most in need—the poor, the more disadvantaged and rural populations—and some of this progress is mirrored in its low rates of anemia as demonstrated by half of its districts (57.9%; 113 of 195) having less than 5% mean overall anemia prevalence in 2018.

With few exceptions, we see that countries with the subnational units with the best anemia prevalence rates in 2000 continue to have administrative units that perform well in 2018, and likewise for countries with the worst-performing subnational units. To illustrate where these high and low pockets continue to be most pervasive and how their rates of change contribute to maintaining this relative status, we overlaid the highest and lowest deciles for prevalence (Fig. 1a,b) and AROCs (Fig. 1d) for overall anemia among WRA across LMICs to simultaneously show the best- and worst-performing districts as defined by both of these measures over the study period (Fig. 1c). Much of Central and South America had districts with the lowest levels of prevalence of overall anemia in 2000 and 2018, with some areas experiencing the largest decreases over time (largest AROCs), including in western Colombia and central and southern Peru. Much of Mexico and El Salvador, as well as districts in western Honduras, central Ecuador and select districts in eastern Brazil, also had among the lowest prevalence levels in both years, whereas western Bolivia and western Guatemala experienced some of the largest declines in the period that led to their place among the lowest decile of anemia prevalence in 2018. Within these same countries, however, there were also districts with the highest prevalence levels and/or largest increases or stagnating trends in anemia (smallest AROC) between 2000 and 2018. Districts in southern Mexico, eastern Honduras, eastern Venezuela and eastern Colombia had among the lowest prevalence levels in 2000, but increases pushed these districts out of the lowest prevalence decile by 2018. Eastern Guatemala, eastern Ecuador and northern Bolivia had among the highest prevalence levels in both 2000 and 2018. In Asia, northern Vietnam and large stretches of China experienced some of the largest declines and had the lowest levels of anemia prevalence. Districts throughout Uzbekistan, Pakistan, India and Papua New Guinea and in northern Myanmar, however, saw the highest consistent prevalence, and the centers of Laos and India and parts of Afghanistan experienced among the largest increases or stagnating trends (smallest AROC). Several African countries had among the highest levels of anemia in both years, including Senegal, Mali, Côte d’Ivoire, eastern Ghana, southern Benin, central Niger, Nigeria, Gabon, Democratic Republic of the Congo, Tanzania, Kenya, Ethiopia, Somalia, Malawi, Mozambique, Zimbabwe and Egypt, and the belt across the Sahel witnessed some of the worst stagnation. No African districts ranked among the lowest decile of anemia prevalence, but there were areas in Ethiopia, Tanzania, Democratic Republic of the Congo, South Africa and a few other select districts that experienced some of the fastest decreases.

Overall, 71 (86.6%) LMICs experienced decreases in mean anemia prevalence in most of their districts over the 2000–2018 period, and seven (8.5%) LMICs (Cape Verde, China, Kyrgyzstan, Malaysia, Namibia, Tunisia and Turkmenistan) had annualized improvements (declines) in all districts. Increases in overall anemia prevalence were experienced in the majority of districts in nine LMICs (Burundi, Central African Republic, Côte d’Ivoire, Gabon, Gambia, Nigeria, Republic of the Congo, Tajikistan and Yemen), and no countries experienced increases in all their districts. Many countries experienced extreme differences in their rates of change across their subnational units: 57 (69.5%) LMICs had at least 2.5% annualized decreases and increases across their districts, whereas 18 (22.0%) LMICs had districts with at least 5% AROC in both directions.

### Prevalence and trends of anemia by severity

**Fig. 2:**
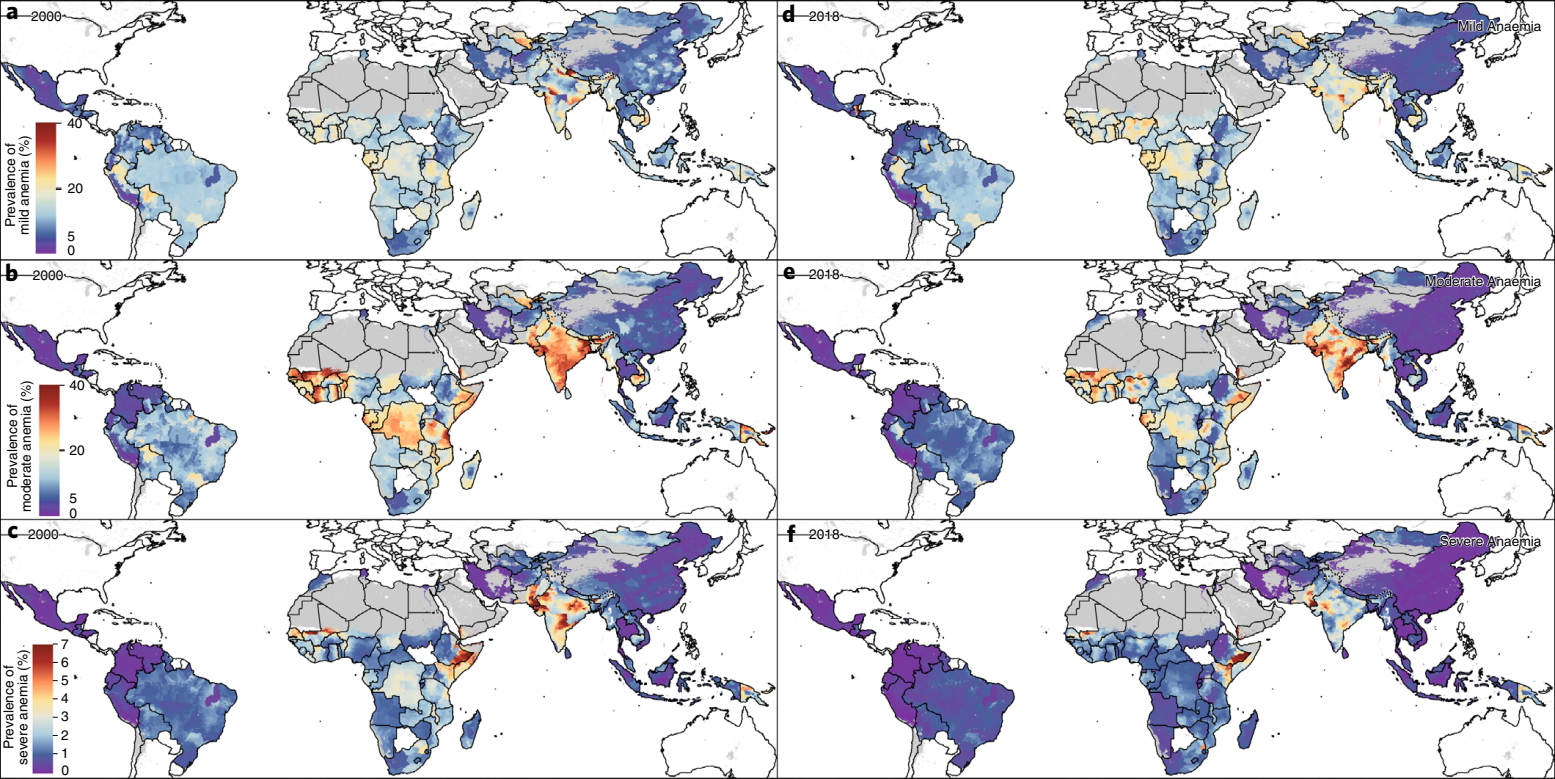
Prevalence of anemia in WRA by severity in LMICs (2000 and 2018). **a**–**f**, Prevalence of anemia stratified by severity among WRA (ages 15–49) at the second administrative unit (for example, district) level. Prevalence of mild anemia among WRA in 2000 (**a**) and 2018 (**d**). Prevalence of moderate anemia among WRA in 2000 (**b**) and 2018 (**e**). Prevalence of severe anemia among WRA in 2000 (**c**) and 2018 (**f**). See Supplementary Table 7 for the cutoffs defining mild, moderate and severe anemia. Maps reflect administrative boundaries, land cover, lakes and population; gray-colored grid cells had fewer than ten people per 1 × 1-km grid cell and were classified as ‘barren or sparsely vegetated’, whereas white-colored grid cells were not included in this analysis^42–47^.

**Extended Data Fig. 1:**
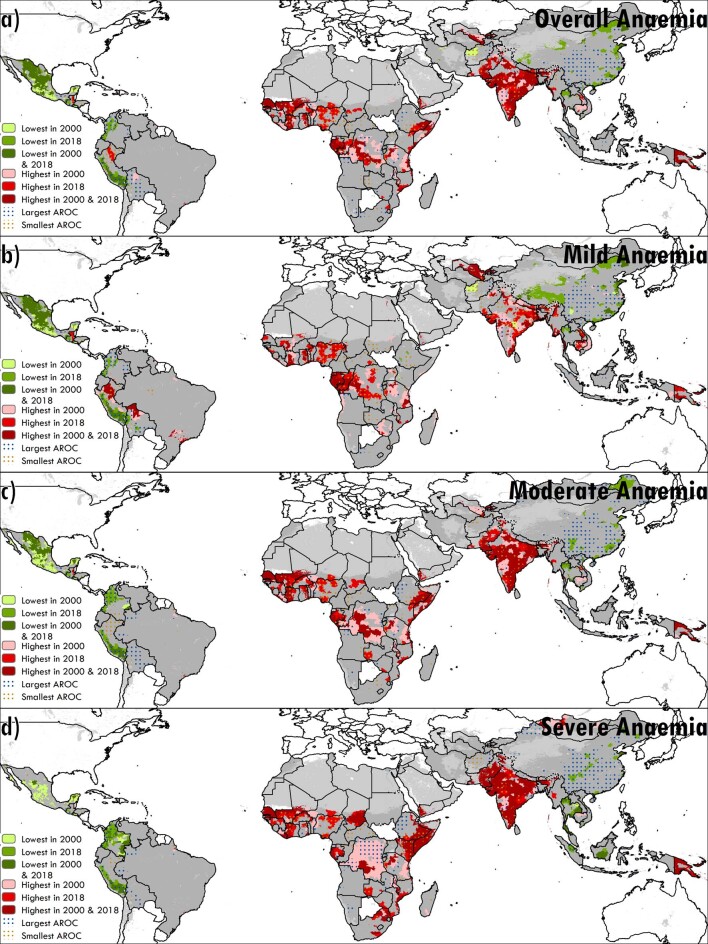
Highest- and lowest-performing second administrative-level units stratified by anaemia severity in women of reproductive age across LMICs (2000–2018). **a**–**d**, Overlapping population-weighted highest and lowest deciles of prevalence and weighted annualised rates of change (AROC) by severity at the second administrative level between 2000 and 2018 for overall (**a**), mild (**b**), moderate (**c**), and severe anaemia (**d**) among WRA. Maps reflect administrative boundaries, land cover, lakes, and population; grey-coloured grid cells had fewer than ten people per 1 × 1-km grid cell and were classified as “barren or sparsely vegetated”, while white-coloured grid cells were not included in this analysis^42–47^.

Mean prevalence of moderate and severe anemia had reduced in the majority (84.1%; 18,441 of 21,917) of districts across LMICs between 2000 and 2018 (Fig. 2). In almost a quarter of the districts in which moderate and severe anemia had declined (24.5%; 4,526 of 18,441 across 79 LMICs), mild anemia had increased, indicating a downward shift in severity levels over the populations. Among these, three-quarters (76.0%; 3,476 of 4,562) saw decreases in overall anemia, suggesting an overall shift toward normal levels of hemoglobin regardless of the historical baseline and in spite of the observed increased prevalence of mild anemia. This is further corroborated by the remaining 13,915 districts, which experienced decreases in moderate, severe and mild anemia. Among the districts that saw increases in prevalence of moderate and severe anemia (15.9%; 3,476 of 21,917), 91.3% (3,175 of 3,476 in 57 LMICs) experienced increases in overall anaemia, indicating a population-wide shift toward reduced hemoglobin levels. This was seen particularly in Yemen and Nigeria, where 81.7% (272 of 333) and 68.8% (533 of 775) of their districts, respectively, saw increases in overall, moderate and severe anemia. In contrast, only 276 districts saw increases in moderate and severe anemia but decreases in overall anemia, possibly indicating a subpopulation that has been left behind while the majority trend is toward non-anemic hemoglobin levels. Of note, Papua New Guinea and Burkina Faso experienced this divergent trend where 11.5% (10 of 87) and 11.1% (5 of 45) of their districts, respectively, saw increases in the prevalence of moderate and severe anemia while overall anemia decreased. Our stratified maps of the highest- and lowest-decile districts for prevalence and AROC for mild, moderate and severe anemia offer a detailed view of these shifts in severity across and within LMICs over time (Extended Data Fig. 1).

### Absolute and relative geographic inequalities of anemia

**Fig. 3:**
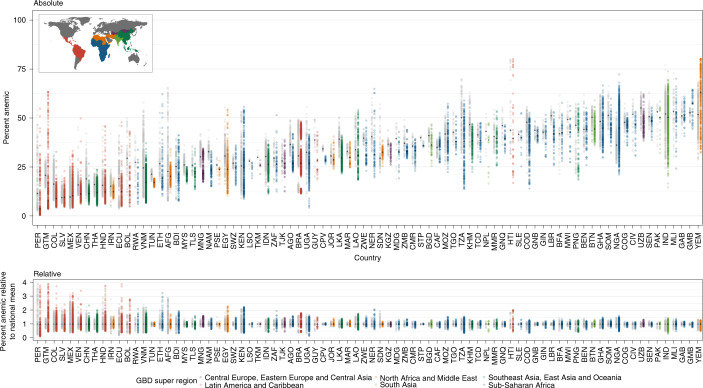
Geographical inequality in overall anemia among WRA across 82 countries for 2000 and 2018. **a**, Absolute inequalities: range of overall anemia estimates in WRA in second administrative-level units within 82 LMICs. **b**, Relative inequalities: range of ratios of overall anemia estimates in WRA in second administrative-level units relative to country means (administrative level/country level). Each dot represents a second administrative-level unit. The lower bound of each bar represents the second administrative-level unit with the lowest overall anemia in WRA in each country. The upper end of each bar represents the second administrative-level unit with the highest overall anemia in WRA in each country. Thus, each bar represents the extent of geographic inequality in overall anemia in WRA estimated for each country. Bars indicating the range in 2018 are colored according to their GBD super-region^48^ (Extended Data Fig. 3). Gray bars indicate the range in overall anemia in WRA in 2000. The black diamond in each bar represents the median and mean overall anemia in WRA estimated across second administrative-level units in each country and year for the absolute (median) and relative (mean) inequalities plots. A colored bar that is shorter than its gray counterpart indicates that geographic inequality has narrowed.

In addition to the overall trend toward lower levels of anemia prevalence, the heterogeneity of district-level anemia prevalence and, thus, subnational inequality has decreased over the last two decades. By plotting the absolute geographic inequalities (Fig. 3a), we show the range of overall anemia prevalence among each country’s districts in 2000 and 2018. Subnational inequalities between districts with the highest and lowest anemia prevalence in each country have increased in most (65.9%; 54 of 82) LMICs over the study period. Absolute inequalities among districts as well as national median anemia prevalence increased in six countries during the period from 2000 to 2018: Yemen (2.4-fold to 2.6-fold difference; 51.7% (34.0–68.6%) to 63.0% (50.9–74.3%) national median prevalence); Gambia (1.2-fold to 1.5-fold difference; 52.7% (30.4–74.7%) to 637.4% (47.4–66.2%)); Nigeria (2.0-fold to 3.4-fold difference; 36.1% (17.4–58.4%) to 44.8% (37.2–66.2%)); Central African Republic (1.3-fold to 1.6-fold difference; 35.0% (19.5–53.5%) to 36.2% (20.4–54.2%)); El Salvador (3.3-fold to 5.0-fold difference; 9.2% (4.6–16.8%) to 9.5% (3.1–21.4%)); and Gabon (1.4-fold to 1.5-fold difference; 51.0% (32.2–70.5%) to 51.2% (33.8–67.9%)). Although absolute inequalities had also increased in the other 48 LMICs, national median anemia prevalence decreased in these countries, indicating select exemplar districts that made progress and/or districts that were left behind in national progress. Overall, 28 LMICs reduced absolute inequalities as well as their national median anemia prevalence; most notably, China had reduced absolute inequalities from 5.6-fold to 4.7-fold across its districts, reducing its national median from 18.8% (10.2–30.9%) to 11.4% (4.4–22.7%) between 2000 and 2018. In 2000, 19 LMICs experienced ≥3-fold difference in overall anemia, and six LMICs experienced ≥6-fold difference in overall anemia (Afghanistan, Ecuador, Iran, Mexico, Peru and Vietnam); in 2018, 30 LMICs had ≥3-fold difference, and 11 LMICs had ≥6-fold difference, across districts (Bolivia, Colombia, Ecuador, Ethiopia, Guatemala, Honduras, Kenya, Mexico, Peru, Uganda and Venezuela) (Supplementary Table 10).

Our relative inequality plot shows the relative deviation of each country’s districts from their national mean anemia prevalence (Fig. 3b). To elucidate these within-country differences, consider that, in 2000, overall anemia prevalence varied across the national level by as much as 5.8-fold (9.5% (6.4%–13.7%) in El Salvador; 55.5% (41.4%–69.4%) in Gabon), and, in 2018, overall anemia varied by as much as 7.0-fold at the national level (8.2% (3.5%–16.3%) in El Salvador; 57.4% (51.4%–63.5%) in Yemen). Within-country relative inequalities in overall anemia increased in 63 LMICs between 2000 and 2018, with some of the most apparent deviations in Guatemala, Venezuela, Colombia, Ecuador, Bolivia, Thailand, Ethiopia, Egypt and Tajikistan; 19 LMICs experienced decreases in relative inequalities, including Iran, Vietnam, Palestine and Sudan. Although many of the countries with large subnational disparities in anemia prevalence could use the results from this study to efficiently target precision public health interventions where they are most needed, there is a second set of countries that had low subnational inequalities and high national prevalence, indicating a pervasive problem where ubiquitous intervention coverage is warranted. In 2018, among the 21 countries that qualified as high public health problems with a national mean overall anemia prevalence above 40%, four of these countries had low relative inequalities ranging from 75% to 125% of the national median: Gabon, Guinea-Bissau, Republic of Congo and Senegal.

### Population size, severity and disability burden of anemia

**Fig. 4:**
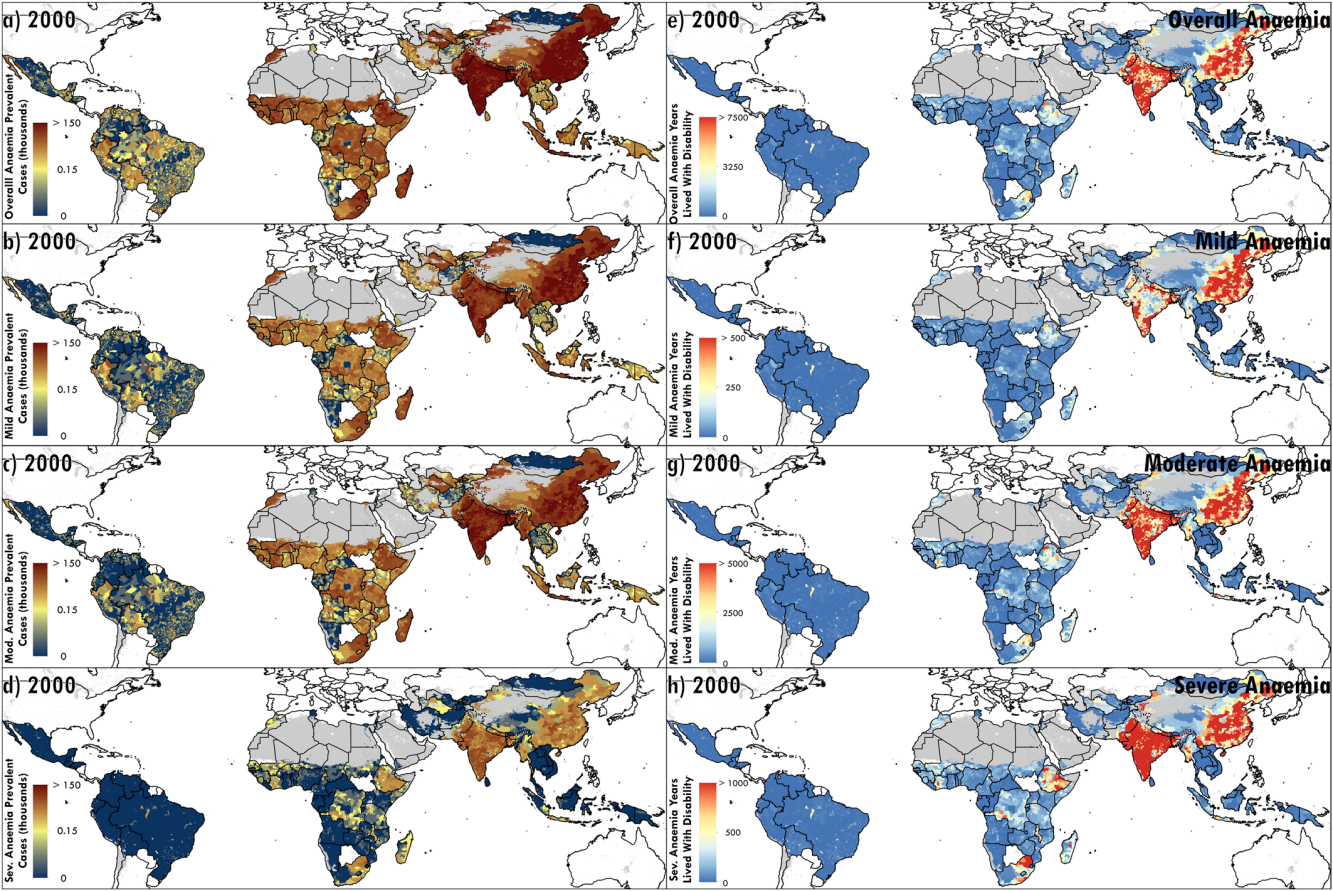
Counts and YLDs by anemia severity among WRA across LMICs in 2018. **a**–**d**, Number of WRA across 82 LMICs with overall (**a**), mild (**b**), moderate (**c**) and severe (**d**) anemia in 2018 by second administrative-level units. **e**–**h**, Number of YLDs among WRA attributable to overall (**e**), mild (**f**), moderate (**g**) and severe (**h**) anemia in 2018 by second administrative-level units. Maps reflect administrative boundaries, land cover, lakes and population; gray-colored grid cells had fewer than ten people per 1 × 1-km grid cell and were classified as ‘barren or sparsely vegetated’, whereas white-colored grid cells were not included in this analysis^42–47^.

**Extended Data Fig. 2:**
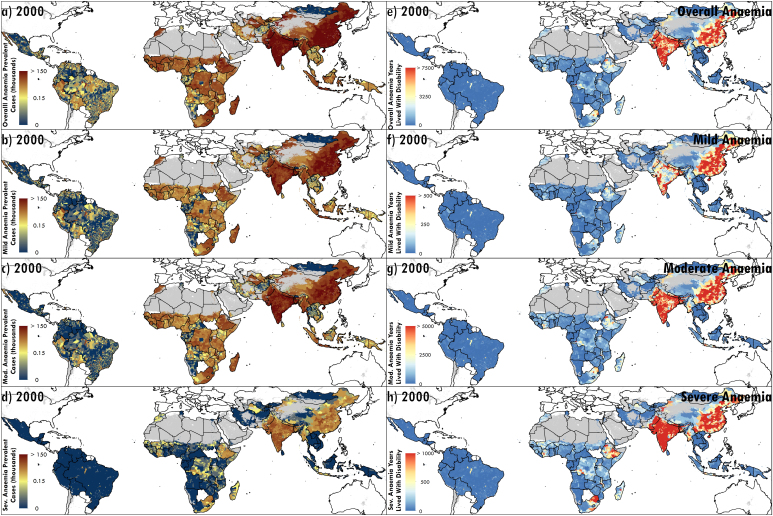
Counts and years lived with disability (YLDs) by anaemia severity among WRA in 2000. **a**–**d**, Number of WRA across 82 LMICs with overall (**a**), mild (**b**), moderate (**c**), and severe anaemia (**d**) in 2000 by second administrative-level units. **e**–**h**, Number of years lived with disability (YLDs) among women of reproductive age (WRA) attributable to overall (**e**), mild (**f**), moderate (**g**), and severe anaemia (**h**) in 2000 by second administrative-level units. Maps reflect administrative boundaries, land cover, lakes, and population; grey-coloured grid cells had fewer than ten people per 1 × 1-km grid cell and were classified as “barren or sparsely vegetated”, while white-coloured grid cells were not included in this analysis^42–47^.

Of the estimated 1.2 billion WRA across the 82 LMICs represented by our analysis in 2000, we estimate that 378.3 million (95% uncertainty interval: 308.0–456.0) (32.8% (26.7–39.5)) of WRA were anemic (Extended Data Fig. 2a). Of these, 178.4 million (134.6–231.8) or 47.2% (43.7–50.8) were categorized as having mild anemia, whereas 182.4 million (138.1–234.5) or 48.2% (44.9–51.4) were moderate anemia cases, and 17.4 million (11.2–26.3) or 4.6% (3.6–5.8) were severe anemia cases (Extended Data Fig. 2b–d). In 2018, of the 1.5 billion WRA represented by our analysis, 449.1 million (382.4–526.9) (30.4% (25.9–35.6)) were estimated to be anemic—224.8 million (180.9–275.7) (50.1% (47.3–52.3%)) with mild cases of anemia, 208.1 million (173.1–247.3) (46.3% (45.3–46.9)) with moderate cases of anemia, and 16.1 million (12.2–21.3) (3.6% (3.2-4.0)) with severe cases of anemia (Fig. 4a–d).

A large proportion of anemic WRA were concentrated in a few countries in 2018; 83.0% (81.0–85.3) of overall anemia occurred in the Asian (61.7% (60.9–62.9)) and sub-Saharan African (21.3% (20.1–22.4)) regions (Fig. 4a). An estimated 59.6% (55.6–63.2) of anemic WRA, amounting to an estimated 267.5 million (241.5–293.2) cases across LMICs, lived in just four countries in 2018: India (181.3 million (171.4–190.2) cases; 40.4% (36.1–44.8) of anemia burden), China (39.5 million (32.3–46.9); 8.8% (8.9–8.5)), Pakistan (23.8 million (15.3–32.6); 5.3% (4.0–6.2)) and Nigeria (23.0 million (22.5–23.5); 5.1% (5.9–4.5)). In 2018, we estimated that 65.2% (14,292 of 21,917) of districts contained fewer than 5,000 anemic WRA, 14.6% (3,199 of 21,917) with 5,000–14,999, 12.4% (2,716 of 21,917) with 15,000–49,999, 4.5% (991 of 21,917) with 50,000–150,000 and 1.7% (377 of 21,917) with 150,000–250,000, and 1.7% (374 of 21,917) had more than 250,000 WRA with any severity of anemia (Supplementary Table 12). The 374 districts that had more than 250,000 anemic WRA each were in 22 LMICs: Angola, Bangladesh, Brazil, Burkina Faso, Cameroon, China, Côte d’Ivoire, Democratic Republic of the Congo, Ethiopia, Ghana, Haiti, India, Indonesia, Madagascar, Morocco, Myanmar, Nepal, Pakistan, Peru, South Africa, Tanzania and Togo. Across the 1,545 first administrative-level units (hereafter ‘provinces’) in the 82 LMICs, 66 provinces located in 12 LMICs (Angola, Bangladesh, Brazil, China, Ethiopia, India, Indonesia, Myanmar, Nepal, Nigeria, Pakistan and South Africa) each had 1 million or more anemic WRA in 2018. All five of the provinces with the highest estimated number of WRA with anemia in 2018 were in India and Pakistan: Uttar Pradesh in India (29.0 million (26.3–31.5)), Bihar in India (15.8 million (14.4–17.1)), West Bengal in India (15.5 million (14.4–16.6)), Maharashtra in India (15.4 million (13.4–17.2)) and Punjab in Pakistan (12.6 million (6.8–18.8)).

Stratifying by severity, an estimated 57.9% (55.1–60.9) of moderately or severely anemic WRA lived in only three countries in 2018: India (103.4 million (94.2–112.7) cases; 46.1% (41.9–50.8%) of moderate or severe WRA anemia cases), Pakistan (13.4 million (8.5–19.0) cases; 6.0% (4.6–7.1%)) and China (13.0 million (10.2–16.5) cases; 5.8% (5.5–6.1%)) (Fig. 4c,d). We found that 133 districts had more than 250,000 WRA with moderate or severe anemia in 2018, located in nine LMICs: Bangladesh (two districts), Brazil (one district), China (one district), Côte d’Ivoire (one district), Democratic Republic of the Congo (one district), India (118 districts), Nepal (one district), Pakistan (seven districts) and Peru (one district). The five provinces with the highest estimated numbers of moderate or severe WRA in 2018 were also all in India and Pakistan: Uttar Pradesh in India (16.7 million (14.7–19.0)), Bihar in India (9.4 million (8.4–10.5)), Maharashtra in India (8.2 million (6.7–9.8)), West Bengal in India (8.1 million (7.2–9.2)) and Punjab in Pakistan (6.9 million (3.7–10.6)).

Multiplying counts in each anemia severity category with the appropriate disability weights from the GBD study^30^ allowed us to visualize where the majority of YLDs (attributable burden) due to anemia among WRA have been most concentrated in LMICs and how it has reduced over time (Extended Data Fig. 2e–h and Fig. 4e–h). Overall anemia contributed 12.7 million (5.9–22.2) YLDs in 2000, with 0.7 million (0.3–1.1), 9.4 million (4.7–15.4) and 2.6 million (0.9–5.7) YLDs from mild, moderate and severe anaemia, respectively (Extended Data Fig. 2e–h). By 2018, YLDs had increased to 14.0 million (9.0–20.4) overall; mild, moderate and severe anemia increased to 0.8 million (0.5–1.3), 10.7 million (7.2–15.1) and reduced to 2.4 million (1.3–4.1) YLDs, respectively (Fig. 4e–h). In 2018, 0.7% (145 of 21,917) of districts each contributed more than 15,000 YLDs due to overall anemia among WRA; these districts were in just nine LMICs: Bangladesh, Brazil, China, Côte d’Ivoire, Democratic Republic of the Congo, India, Nepal, Pakistan and Peru. Districts with over 5,000 YLDs attributed to overall anemia among WRA (3.1% (677 of 21,917)) were in 33 LMICs. The three countries with the most YLDs from overall anemia among WRA in 2018 were India (6.43 million (5.80–7.11) YLDs), Pakistan (0.85 million (0.53–1.21) YLDs) and China (0.83 million (0.65–1.06) YLDs). In 2018, 532 of 21,917 districts across 30 LMICs contributed more than half of YLDs (7.0 million (4.8–9.6)) attributed to overall anemia across the 82 LMICs in this analysis.

Between 2000 and 2018, the majority of districts across LMICs experienced reductions in estimates of YLDs attributable to moderate anemia (54.1%; 11,859 of 21,917 districts) and severe anemia (67.9%; 14,876 of 21,917 districts) among WRA (Extended Data Fig. 2g,h and Fig. 4g,h). This progress in reducing YLDs due to moderate and severe anemia was especially evident in China (1.74 million (1.40–2.17) YLDs in 2000 and 0.74 million (0.57–0.95) in 2018; declines in 359 of 364 districts). In 10,078 districts located across all 82 LMICs, however, YLDs from moderate anemia increased, including in 12 countries where all districts experienced increases: Burkina Faso, Chad, Côte d’Ivoire, Guinea-Bissau, Jordan, Mali, Pakistan, São Tomé and Príncipe, Senegal, Sierra Leone, Somalia and Yemen. The YLDs from severe anemia increased in 7,061 districts across 79 LMICs, including in Yemen (332 of 333 districts), Burkina Faso (44 of 45 districts), Chad (53 of 55 districts) and Jordan (48 of 52 districts). The district with the largest increase in YLDs from moderate anemia was Bangalore (Karnataka) in India, with 23,003 (7,665–44,041) YLDs in 2000 and 43,497 (30,063–57,816) YLDs in 2018. The largest increase in YLDs from severe anemia was in Bijnor (Uttar Pradesh) in India, with 791 (282–1,627) YLDs in 2000 and 6,884 (4,767–9,476) YLDs in 2018.

### Prospects of meeting 2030 WHO GNT

**Fig. 5:**
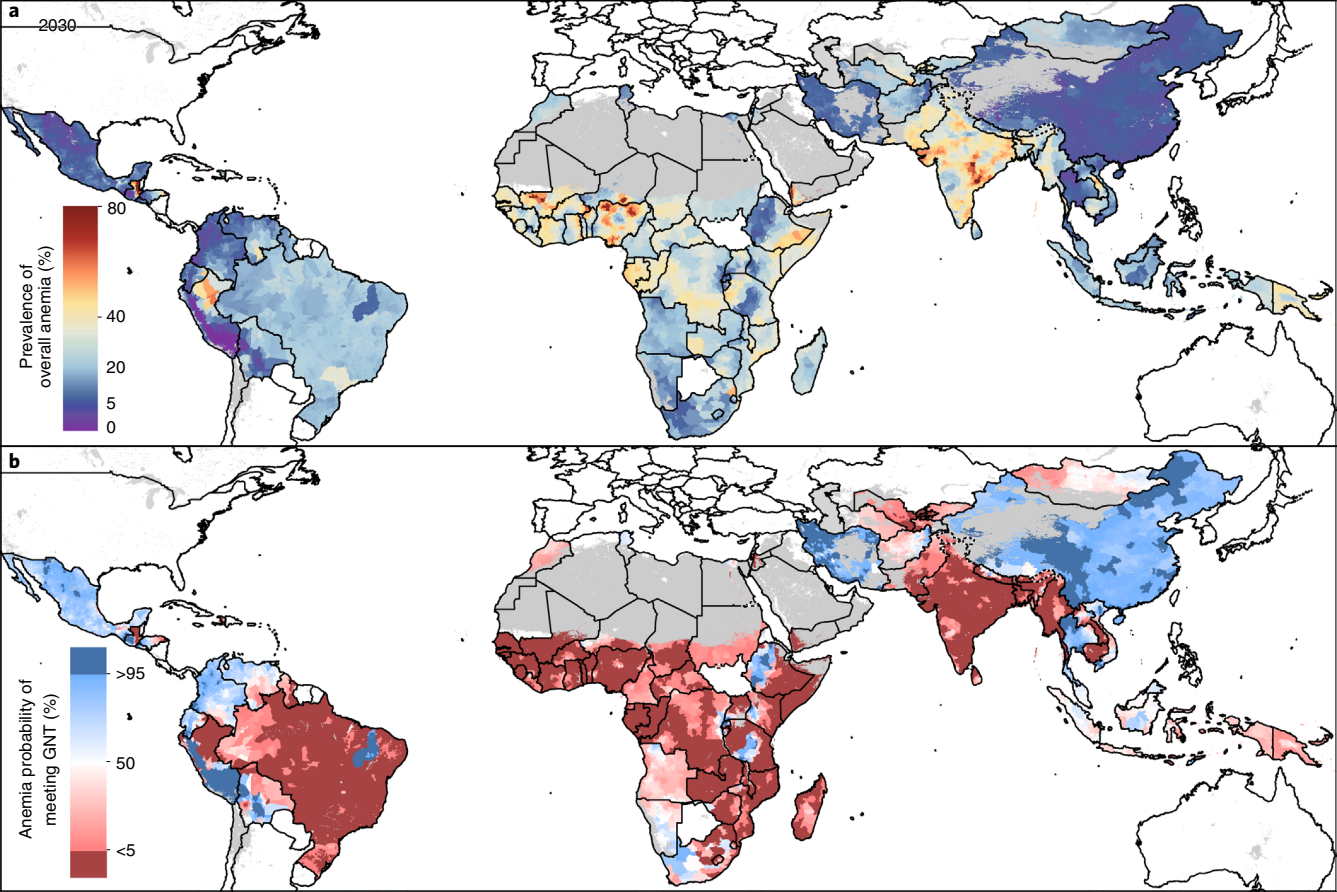
Prevalence for overall anemia among WRA in 2030 and probability of achieving the WHO GNT for overall anemia by 2030. **a**, Predicted prevalence of overall anemia among WRA in 2030 by second administrative-level units. **b**, Probability of achievement of the WHO GNT to reduce overall anemia in WRA by 50% by the year 2030, with the year 2012 as a baseline, by second administrative-level units. Maps reflect administrative boundaries, land cover, lakes and population; gray-colored grid cells had fewer than ten people per 1 × 1-km grid cell and were classified as ‘barren or sparsely vegetated’ or were not included in this analysis^42–47^.

We applied the estimated AROCs to the final year of our estimates to predicted anemia prevalence estimates for the year 2030 (Fig. 5a). In 2018, 29 of 21,917 districts had >80% mean prevalence of overall anemia; if current trends continue, 100 districts across Guatemala (11 districts), Haiti (seven districts), India (two districts), Nigeria (four districts) and Yemen (76 districts) are estimated to reach >80% mean prevalence for overall anemia among WRA by 2030. Subnational inequalities in Guatemala are expected to continue, and, although 17 northeastern districts are projected to reach >75% prevalence by 2030, 179 southwestern districts are expected to reduce to below 5% prevalence, considered acceptable levels of anemia. Including Guatemala, we estimate that districts in 15 LMICs will have less than 5% prevalence in overall anemia by 2030: Afghanistan (1 of 399 districts), Bolivia (16 of 117), China (2 of 364), Colombia (123 of 1,065), Ecuador (1 of 223), El Salvador (32 of 266), Guatemala (179 of 354), Honduras (13 of 298), Mexico (31 of 2,454), Peru (120 of 195), Rwanda (3 of 30), Thailand (28 of 928), Uganda (4 of 203), Venezuela (1 of 338) and Vietnam (1 of 710). Based on current projections, we expect that 21 LMICs will maintain high national levels of overall anemia (≥40%) in 2030; on a subnational scale, 16.4% (3,594 of 21,917) of districts located in 61 LMICs are estimated to have ≥40% anemia prevalence in 2030 if existing trajectories continue.

Assuming that recent trends persist, and using the year 2012 (the year that WHO GNTs were established) as a baseline, we estimated the probability of subnational units across LMICs achieving the WHO GNT to relatively reduce anemia by 50% by the year 2030 (Fig. 5b). By 2030, only three of the 82 (3.7%) LMICs in this analysis are expected to achieve the target of 16.2% at a national scale with a high probability (>95% posterior probability): China, Iran and Thailand. Subnationally, however, no countries have a high probability of meeting the WHO GNT for anemia in all provinces, nor in all their districts, by the target year. About a third (31.7%; 26 of 82) of LMICs have a high probability (>95%) of meeting the target in at least one district, whereas only three LMICs (Guatemala, Iran and Peru) have a high probability of meeting the goal in most districts. We expect far more LMICs to have a low probability (<5% posterior probability) of achieving the target nationally and subnationally. By 2030, 64.6% (53 of 82) of LMICs have a low probability (<5%) of meeting the WHO GNT nationally, whereas 21.2% (18) have a low probability in all provinces, and four LMICs (Gabon, Gambia, Senegal and Togo) have a low probability of meeting the target in all their districts. Although 15 (18.3%) LMICs have a >50% probability of achieving the WHO GNT by 2030 nationally, five (6.1%) LMICs have >50% probability of achieving the target in all their province-level units, and only Tunisia has >50% probability of meeting the goal in all its district-level units by 2030.

Large inequalities in achieving the WHO GNT are expected to continue, and 56.1% (46 of 82) of LMICs are predicted to have districts with both >50% and <50% probability of meeting the goal by 2030. We estimate that 20 LMICs have districts with both high probability (>95%) and low probability (<5%) of achieving the WHO GNT by 2030.

## Discussion

Marginal declines in anemia prevalence among WRA in LMICs have left individuals, populations and nations at risk of reduced economic productivity^3,4^, increased all-cause mortality^5^ and increased potential for adverse outcomes for mothers and newborns^31^. Although most district-level units (80.5%; 17,651 of 21,917 districts) decreased their prevalence between 2000 and 2018, the overall prevalence among the 82 LMICs in our analysis has only declined, from 35.6% (95% uncertainty interval: 25.9–46.6) to 31.6% (25.7–38.2) in the nearly 20-year period. Even for the many countries with overall improvements in reducing anemia prevalence, our results highlight enduring disparities across global geographic regions and within select countries and subnational locations that have stagnated or fallen behind the general improvements of their neighbors. Although three LMICs (China, Iran and Thailand) have a high probability of meeting the WHO GNT of reducing anemia among WRA by 50% by the year 2030, no LMIC is predicted to meet the target in all provinces or all districts. Most LMICs (64.6%; 53 LMICs) have a low probability (<5%) of meeting the target even on a national scale. Broad inequalities are expected to continue into 2030; we estimate that 20 LMICs have districts with a high probability of meeting the target as well as districts with a low probability of meeting the target. Furthermore, population growth during this period has led to substantial increases in the number of WRA affected by anemia in various locations. Although the overall number of prevalence of anemia in WRA has decreased, growing populations have caused the number of anemic WRA to increase from 378.3 million to 449.1 million, with the largest increases in Central Asia and western, central and eastern sub-Saharan Africa (54.7% increase: 20.2–31.3 million; 88.0% increase: 24.6–46.2 million; 53.1% increase: 7.6–11.6 million; and 51.8% increase: 20.9–31.8 million, respectively), offsetting the large decreases seen in East Asia and Andean South America (44.1% decrease: 70.9–39.6 million and 13.7% decrease: 5.6–4.8 million, respectively).

The multitude of different diseases and injuries, nutritional and behavioral risk factors and sociodemographic factors that can lead to anemia mandate inter- and multi-sectorial approaches involving stakeholders and actors in the public and private sectors and coordination across food systems and health-related sectors if large-scale reductions in anemia prevalence are to be achieved^2,16^. GBD 2019 estimated the top-ranked global causes of anemia in WRA to be, in order, dietary iron deficiency; thalassaemia trait; sickle cell trait; menstrual disorders; endocrine, metabolic, blood and immune disorders; and malaria^19^, although the specific cause composition varied by country and age group. Regardless of anemia prevalence levels, the WHO recommends a diet with adequate bioavailable iron and iron folate and micronutrient fortification of rice and flours where they are major staples^16^. Intermittent or daily iron and folic acid supplementation is recommended for WRA depending on pregnancy and postpartum status, menstruation, tuberculosis diagnosis and population-level prevalence, with key prevalence thresholds of 20% and 40%^16^. Research suggests that multiple micronutrient supplementation for pregnant women in LMICs might provide additional benefits of reducing low-birth-weight outcomes, small-for-gestational-age outcomes and preterm birth outcomes^32^. Universal antenatal hemoglobin testing can help identify anemic women early, providing time to investigate causality and eliminate anemia before delivery^33^. In endemic areas, malaria control has demonstrated over 25% and 60% reduction in overall anemia and severe anaemia, respectively^16^. Countries with high levels of anemia and malaria^34^, such as Mali, Democratic Republic of the Congo, Papua New Guinea, Pakistan and India, might benefit from increased malaria control efforts. Proper water and sanitation, including safe water and education on hand-washing and hygienic disposal of fecal matter, can reduce infection risks and related nutritional losses^2^. Additionally, the association between intestinal helminths and anemia, due to nutritional theft and direct blood loss, has led the WHO^35^ to recommend de-worming pregnant women in helminth-endemic areas. LMICs with co-distribution of helminths^36^ and high prevalence of anemia include Nigeria, Madagascar, Bangladesh and Papua New Guinea. A variety of intervention delivery platforms could be used, including regular routine antenatal care visits, community health workers and community-based social marketing^16^. Strategies and delivery platforms should be context-specific and tailored for populations based on the local culture and disease burden; these estimates provide policy-makers the opportunity to ‘aim to ensure the most vulnerable members of the populations are reached’^16^. For those with chronic conditions, such as sickle cell disease, thalassaemia, inflammatory bowel disease, endocrine disorders or chronic kidney disease, more nuanced and potentially more intensive treatments are likely to be required to manage the underlying disease and reduce anemia burden.

Future research could cross-reference our estimates with implemented policies by location to determine effective strategies and exemplars of progress to further aid policy-makers and decision-makers. Although the models used in this study are not inherently inferential, the complex, yet still relatively predictable, pathways that lead to anemia suggest that those populations with a high burden of anemia are also highly likely to have a high burden of the diseases that cause anemia and are likely to be suffering from multiple simultaneous deprivations of nutrition, economics, health systems and overall resilience. We have seen success, as evidenced in Peru, in using targeted programs to reach those most in need, and understanding where they might be is a prerequisite toward analogous future campaigns against anemia and many other inequitable global health crises. These maps thus provide a roadmap to identifying the most vulnerable populations in the world and can be viewed concurrently with our previous work tracking progress and/or predictions of meeting other WHO GNTs—including geospatial annual estimates of exclusive breastfeeding^25^, childhood overweight and wasting^37^ and childhood stunting, wasting and underweight^26,27^—as well as estimates of child diarrhea^38^, child mortality^39^, malaria^34^, inherited blood disorders (for example, sickle cell diseases^40^), helminths^36^ and food system sustainability^41^ to gain a more complete view of the needs of specific countries and communities.

Although this study sheds light on the varied levels of anemia across countries, the unequal levels within them and the varied rates of progress that have led them to their status, it is not without limitations. Most notably, the accuracy of these estimates is predicated on the quality and the quantity of the underlying data. We have invested substantial effort in building a geo-located database of over 3 million women for the purpose of this analysis, but large gaps in both the spatial and temporal data coverage remain. Supplementary Figs. 6 and 7 show the number of years of data underpinning each administrative level-one and level-two unit in the analysis, and Supplementary Figs. 1–5 illustrate the spatial resolution and temporal location of this data. The uncertainty of these estimates, shown in Supplementary Figs. 10–13, is largely driven by the consistency and volume of the data and, at times, can be quite high. Our validation analysis shows that our model is well-calibrated with minimal bias and good coverage of the 95% prediction intervals, demonstrating that the uncertainty of the estimates is appropriate given the data. To improve the precision of these estimates, increases in data collection and reporting will be needed, and the uncertainty maps provide a starting point for adaptive sampling techniques that can target areas that we uncertainly estimate to have high risk.

Combined with the lack of necessary data that would be needed to perform high-resolution mapping of the conditions that cause anemia, our analysis and some of its limitations underscore the challenges in large-scale global reduction of anemia. Venous sampling of whole blood followed by assessment via automated hematology analyzers is considered the gold standard measurement, but most population-based surveys use capillary samples and the HemoCue colorimetric point-of-care tool to measure hemoglobin concentration and assess population prevalence of anemia. There are documented differences in the concentration of hemoglobin in venous blood samples compared to capillary blood samples, but the direction and consistency of the error introduced by capillary measurement has not been definitively established. We did not have sufficient data to stratify by the mode of assessment in each country at the local level. In addition, we did not estimate anemia by underlying cause, which limits the precision with which we can make specific statements about likely appropriateness of specific interventions for specific locations, although we do expect the epidemiology of anemia to track with the underlying causes of anemia. Similarly, prevalence and count maps of all-anemia burden can be used to target hotspots but are not sufficient to determine the best course of treatment for those communities. Neither the uncertainty from resampling polygonal data to point data, nor the uncertainty from modeled covariates, were accounted for in our models. Uncertainty plots of the outputs in our models can be found in Supplementary Figs. 10–13 and 16. We expect that propagating the uncertainty from the resampling and the modeled covariates would increase the overall uncertainty in our estimates. In contrast, if we were able to incorporate the assessment technique (venous versus capillary) or the processing technique, we expect that accounting for these possible confounders would decrease the uncertainty of these estimates.

The large global burden of anemia continues to underline the need for high-resolution estimates to track progress toward international targets and to aid policy-makers in targeting interventions and scarce resources. The recent addition of anemia reduction as a target for the Sustainable Development Goal 2 further highlights the global importance of the issue^22,23^. This study details the subnational trends in anemia prevalence in WRA across 82 LMICs, broken down by severity, and highlights the local differences in burden and progress within and between countries. The results and the interactive visualizations presented in this study provide an unprecedented opportunity for policy-makers and health institutes to examine the variation in anemia prevalence and its historical progress within their communities and can aid targeting of further data collection, limited resources and interventions to populations most in need.

## Methods

### Overview

This study implemented continuous geostatistics modeling of mild, moderate and severe anemia prevalence over time, from which local-, administrative- and national-level estimates of prevalence, counts and all other estimated quantities were derived. An ensemble approach using a Bayesian generalized linear mixed effects model was used to embed non-linear algorithmically predicted mean functions within a Gaussian process framework, assumed to have a correlated space–time covariance structure. We sampled 1,000 draws from an approximate posterior distribution of this model and generated annual prevalence estimates for mild, moderate, and severe anemia prevalence of WRA (ages 15–49 years) on an approximate 5 × 5-km grid over 82 LMICs from 2000 to 2018 and performed population-weighted aggregation of these gridded estimates to administrative and national levels. Countries were selected for inclusion in this study using the SDI, a summary measure of development that combines education, fertility and poverty^18^. Selected countries were in the low, lower-middle and middle SDI quintiles, with several exceptions (Supplementary Table 3). China, Malaysia and Turkmenistan were included despite high-middle SDIs for geographic continuity with other included countries. Albania, Bosnia-Herzegovina, North Korea and Moldova were excluded due to geographic discontinuity and lack of available survey data. Of this set of countries, we did not generate estimates for 26 countries, as no survey data could be sourced (Supplementary Table 4).

### Data

#### Surveys and hemoglobin data

We extracted each individual woman’s hemoglobin concentrations (g L^−1^), age, pregnancy status, smoking status and elevation from household series, including the Demographic and Health Surveys, the Multiple Indicator Cluster Surveys, the Living Standards Measurement Study and the Core Welfare Indicators Questionnaire, among other country-specific child health and nutrition surveys. Included across our models were 218 geo-referenced household surveys from 2000 to 2018 representing over 3 million WRA. Each individual woman’s record was associated with a cluster, a group of neighboring households or a ‘community’ that acted as a primary sampling unit in the survey design. The 218 surveys with hemoglobin, pregnancy, smoking and elevation data included geographic coordinates or precise place names for each cluster within that survey. In the absence of geographic coordinates for each cluster, we assigned data to the smallest available administrative areal unit in the survey (polygon) while accounting for the survey sample design^49^. Boundary information for these administrative units was obtained as shapefiles either directly from the surveys or by matching to shapefiles in the Global Administrative Unit Layers database^42^ or the Database of Global Administrative Areas (GADM)^50^. In select cases, shapefiles provided by the survey administrator were used, or custom shapefiles were created based on survey documentation. Using methods from our previous works^38^, these areal data were resampled to point locations using a population-weighted sampling approach over the relevant areal unit with the number of locations set proportionally to the number of grid cells in the area and the total weights of all the resampled points summing to 1. In addition, some data sources did not contain hemoglobin concentrations and, instead, reported only the anemia severity category. These severity categories were used directly, whereas hemoglobin concentrations were adjusted and thresholded as described in the following section.

Select data sources were excluded for the following reasons: missing survey weights for areal data, missing sex or age variable, incomplete sampling (for example, only women aged 20–24 years measured) or untrustworthy data (as determined by the survey administrator or by inspection). Data availability plots for anemia by country, data type and year can be found in Supplementary Figs. 1–5.

#### Hemoglobin adjustments and anemia severity

For the purpose of defining anemia severity status, hemoglobin concentrations are often first adjusted for individual smoking status and residential elevation^28^. Many data sources provide some combination of raw hemoglobin, smoking-adjusted hemoglobin, elevation-adjusted hemoglobin and smoking- and elevation-adjusted hemoglobin concentrations. Wherever possible, this study started with the raw hemoglobin concentrations and performed both smoking and elevation adjustments, as suggested by the WHO. If only partially adjusted (either only smoking-adjusted or elevation-adjusted), we performed the second adjustment, and, if only completely adjusted hemoglobin concentrations were available, we used those. The elevation adjustments are shown in Supplementary Table 5, and the smoking adjustments are shown in Supplementary Table 6.

Once the hemoglobin concentrations had been doubly adjusted for smoking and elevation, they were then thresholded into non-anemic, mild anaemia, moderate anaemia or severe anaemia categories using the WHO definitions shown in Supplementary Table 7. Some data sources reported only the anemia severity categories, which were then used directly in the modeling stage. After classification into anemia severity categories, individual-level data observations were then collapsed to cluster-level totals for the number of WRA sampled and total number of WRA who were determined to be mildly, moderately or severely anemic.

#### Temporal resolution

We estimated the prevalence of mild, moderate and severe anemia annually from 2000 to 2018 using a model that allowed us to account for data points measured across survey years and, as such, allows us to predict at monthly or finer temporal resolutions. We were limited, however, both computationally and by the temporal resolution of covariates and, thus, have produced annual estimates (Supplementary Table 8 and Supplementary Fig. 8).

#### Spatial covariates

A variety of socioeconomic and environmental variables were used to predict anemia. Where available, the finest spatio-temporal resolution of gridded datasets was used. These covariates were selected based on their potential to be predictive for anemia and the pathways to anemia, including certain nutritional deficiencies, according to literature review and plausible hypothesis as to their influence. Acquisition of temporally dynamic datasets, where possible, was prioritized to closely align with our observations and to predict the changing dynamics of the anemia severity indicators.

We used covariate-driven predictive models to leverage strength from locations with observations to the entire spatial-temporal domain. Several 5 × 5-km raster layers of putative socioeconomic and environmental correlates of anemia were compiled and used as covariates across the 82 LMICs in the modeling domain (Supplementary Table 8 and Supplementary Fig. 8). These covariates were selected based on their potential to be predictive for anemia and the pathways to anemia, including certain nutritional deficiencies, according to literature review and plausible hypothesis as to their influence. Acquisition of temporally dynamic datasets, where possible, was prioritized to closely align with our observations and to predict the changing dynamics of the anemia severity indicators. Of the 19 covariates included, 12 were temporally dynamic and were re-formatted as a mid-year estimate or synoptic mean for each year in the estimation period. These included average diurnal temperature range, average potential evapotranspiration, average daily mean rainfall (precipitation), outdoor air pollution (PM_2.5_), educational attainment in WRA (ages 15–49 years), enhanced vegetation index, tasselled cap brightness, prevalence of underweight (ages 0–5 years), Healthcare Access and Quality Index, fertility, urbanicity and population. The remaining seven covariate layers were static throughout the study period and were applied uniformly across all modeling years; these covariates included growing season length, irrigation, nutritional yield for vitamin A, nutritional yield for zinc, nutritional yield for iron, distance to rivers and lakes and travel time to nearest settlement with more than 50,000 inhabitants.

Travel time to nearest settlement, nutritional yield for vitamin A, nutritional yield for iron and nutritional yield for zinc were selected because of their potential to be predictive for anemia and the pathways to anemia. Fertility, malaria incidence, population, outdoor air pollution and prevalence of underweight were selected for inclusion in modeling owing to their correlation with a wide variety of health-related outcomes. Average daily mean temperature, average daily mean rainfall, irrigation, land cover, multi-source weighted-ensemble precipitation and tassled cap brightness were selected for their correlation with a variety of crop yields. In addition, the stacking methodology used in this study boosts the predictive performance of individual covariates by leveraging non-linear and high-order interactions among the covariates and generally performs better when given a variety of covariates.

### Analysis

#### Geostatistical model

To model the full distribution of possible indicators of anemia status—that is, all, mild, moderate and severe anemia—we used an ordinal modeling approach^51^ to estimate the relative proportion of each indicator.

We implemented a continuation ratio model to estimate the prevalence of three categories: mild, moderate and severe. We first modeled the proportion of all anemia within a Bayesian hierarchical framework using logistic regression with a spatially and temporally explicit generalized linear mixed effects model. Second, we modeled the probability of being mildly anemic conditional on being anemic (that is, being mildly, moderately or severely anemic) using the same Bayesian modeling framework. Finally, we modeled the probability of being severely anemic conditional on being either moderately or severely anemic. The estimates from the two conditional models were combined with the all-anemia estimates to compute the marginal prevalence of mild, moderate and severe anemia.

For each modeling region, at each cluster, *d*, where $d = 1,2, \ldots n$, and time *t*, where $t = 2000,2001, \ldots ,2018$, the prevalence of all anemia was modeled using the observed number of WRA in cluster *d* who were found to be anemic as a binomial count, *C*_*d*_, among an observed sample of *N*_*d*_:

$$C_d|p_{i\left( d \right),t\left( d \right)},N_d \sim {{{\mathrm{Binomial}}}}\left( {p_{i\left( d \right),t\left( d \right)},N_d} \right)\forall \;observed\;clusters\;d$$

$$\begin{array}{l}{{{\mathrm{logit}}}}\left( {p_{i,t}} \right) = \beta _0 + {{{\boldsymbol{X}}}}_{i,t}\beta + Z_{i,t} + {\it{\epsilon }}_{ctr(i)}\\ + {\it{\epsilon }}_{i,t} + Z_{i,t}\forall \;i \in spatial\;domain\;\forall \;t \in time\;domain\end{array}$$

$$\mathop {\sum }\limits_{h = 1}^3 \beta _h = 1$$

$${\it{\epsilon }}_{ctr} \sim {{{\mathrm{iid}}}}\;{{{\mathrm{Normal}}}}\left( {0,\gamma ^2} \right)$$

$${\it{\epsilon }}_{i,t} \sim {{{\mathrm{iid}}}}\;{{{\mathrm{Normal}}}}\left( {0,\sigma ^2} \right)$$

$${{{\boldsymbol{Z}}}} \sim {{{\mathrm{GP}}}}\left( {0,{{{\mathrm{{\Sigma}}}}}^{{{{\mathrm{space}}}}} \otimes {{{\mathrm{{\Sigma}}}}}^{{{{\mathrm{time}}}}}} \right)$$

$${{{\mathrm{{\Sigma}}}}}^{{{{\mathrm{space}}}}} = \frac{{\omega ^2}}{{{{{\mathrm{{\Gamma}}}}}\left( \nu \right)2^{v - 1}}} \times \left( {\kappa D} \right)^\nu \times {\rm K}_\nu \left( {\kappa D} \right)$$

$${{{\mathrm{{\Sigma}}}}}_{j,k}^{{{{\mathrm{time}}}}} = \rho ^{\left| {k - j} \right|}$$

**Extended Data Fig. 3:**
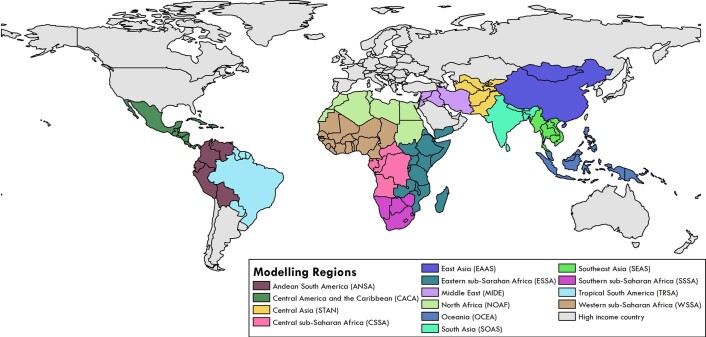
Modelling regions. Modelling regions^48^ were based on geographic and Socio-demographic Index (SDI) regions from the Global Burden of Disease^18^, defined as Andean South America, Central America and the Caribbean, Central sub-Saharan Africa (SSA), East Asia, Eastern SSA, Middle East, North Africa, Oceania, Southeast Asia, South Asia, South SSA, Central Asia, Tropical South America, and Western SSA. Regions in grey (Stage 3) were not included in our models due to high-middle and high SDI. Only 82 low- and middle-income countries (LMICs) were included in this study.

For indices *d,i* and *t*, *(index) is the value of * at the index. The annual prevalence of all anemia, *p*_*i,t*_, in spatial location *i*, in time *t*, was modeled as a linear combination of the three submodels (generalized additive model, boosted regression trees and lasso regression), rasterised covariate values, *X*_*i,t*_, a correlated spatio-temporal random effect term *Z*_*i,t*_, country random effects ${\it{\epsilon }}_{ctr(i)}$, with one unstructured country random effect fit for each country in the modeling region (Extended Data Fig. 3) and all ${\it{\epsilon }}_{ctr}$ sharing a common variance parameter, *γ*^2^, and an independent nugget random effect, ${\it{\epsilon }}_{i,t}$, with variance parameter *σ*^2^. Coefficients *β*_*h*_ in the three submodels *h*∈1,2,3 represent their respective predictive weighting in the logit-link, whereas the joint structured process, *Z*_*i,t*_, accounts for residual spatio-temporal autocorrelation among individual data points that remain after accounting for the predictive effect of the submodel covariates, the country-level random effect, ${\it{\epsilon }}_{ctr(i)}$, and the nugget, ${\it{\epsilon }}_{i,t}$. The spatio-temporal residual process, *Z*_*i,t*_, was modeled as a three-dimensional Gaussian process in space–time centerd at 0 and with a covariance matrix constructed from a Kronecker product of spatial and temporal covariance kernels. The spatial covariance, ${{{\mathrm{{\Sigma}}}}}^{{{{\mathrm{space}}}}}$, was modeled using an isotropic and stationary Matérn function^52^ and the temporal covariance, ${{{\mathrm{{\Sigma}}}}}^{{{{\mathrm{time}}}}}$, as an annual autoregressive (AR1) function over the 19 years represented in the model. In the stationary Matérn function, the covariance between two spatial locations that are Euclidean distance *D* apart is a function of Γ, the gamma function, K_*v*_, the modified Bessel function of the second kind of order $v > 0,\kappa > 0$, a scaling parameter and *ω*^2^, the marginal variance. The scaling parameter, *κ*, is defined to be $\kappa = \sqrt {8v} /\delta$, where *δ* is the range parameter (interpreted to be the approximate distance at which the correlation between two locations drops to 0.1), and *v* is a scaling constant, which is set to 2 rather than fit from the data. The number of rows and the number of columns of the spatial Matérn covariance matrix are both equal to the number of spatial mesh points for a given modeling region. The Matérn kernel is a practical and common choice that can flexibly model a wide variety of spatial surfaces and allows for fitting or selection of the smoothness of the surface, helping to avoid unrealistic over-smoothing^52^. For the temporal kernel, we chose to use an AR1 process owing to its stability, which aligns well with the observed relatively slow and smooth changes in anemia prevalence across time, and for its interpretability. In the AR1 function, *ρ* is the temporal correlation between adjacent time steps, taken to be single years in this study, and *k* and *j* are time steps. The number of rows and the number of columns of the AR1 covariance matrix are both equal to the number of temporal mesh points (19). The number of rows and the number of columns of the space–time covariance matrix, ${{{\mathrm{{\Sigma}}}}}^{{{{\mathrm{space}}}}} \otimes {{{\mathrm{{\Sigma}}}}}^{{{{\mathrm{time}}}}}$, for a given modeling region are equal (the number of spatial mesh points × the number of temporal mesh points). Previous sensitivity analyses on these models showed these modeling choices to be generally quite robust^37,53^.

This approach leverages the residual correlation structure to more accurately predict prevalence estimates for locations with no data while also propagating the dependence in the data through to uncertainty estimates^54^. The posterior distributions were fit using computationally efficient and accurate approximations in R-INLA^55^ (integrated nested Laplace approximation) with the stochastic partial differential equations (SPDE)^56^ approximation to the spatio-temporal Gaussian process using R version 3.5.1. The SPDE approach using INLA was demonstrated elsewhere, including the estimation of health indicators, particulate air matter and population age structure^56^. Uncertainty intervals were generated from 1,000 draws (that is, statistically plausible candidate maps)^57^ created from the posterior-estimated distributions of modeled parameters.

#### Mesh construction

We constructed the finite elements mesh for the SPDE approximation to the Gaussian process regression using a simplified polygon boundary (in which coastlines and complex boundaries were smoothed) for each of the regions within our model. We set the inner mesh triangle maximum edge length (the mesh size for areas over land) to be 0.75 degrees and the buffer maximum edge length (the mesh size for areas over the ocean) to be 5.0 degrees^58^. An example finite elements mesh constructed for eastern sub-Saharan Africa mesh can be found in Supplementary Fig. 7.

#### Post-estimation

To transform grid cell-level estimates into a range of information useful to a wide constituency of potential users, these estimates were aggregated at first and second administrative units specific to each country and at national levels^40^. Although the models can predict all locations covered by available raster covariates, all final model outputs for which land cover was classified as ‘barren or sparsely vegetated’ on the basis of Moderate Resolution Imaging Spectroradiometer satellite data (2013) were masked^59^. Areas where the total population density was fewer than ten individuals per 1 × 1-km grid cell in 2015 were also masked in the final outputs. To compute the YLDs, we applied the corresponding disability weights from the GBD study^30^ on prevalence estimates of the severity bins (mild, moderate and severe anemia) and summed to get the total YLDs for all anemia.

#### Model validation

Models were validated using spatially stratified five-fold out-of-sample cross-validation. To replicate real-world missingness in the datasets and to fairly assess model performance in areas far from observed data, acknowledging the spatial correlation inherent in the observation, holdout folds were created by combining sets of all data falling within first administrative-level units. Validation was performed by calculating bias (mean error), variance (root-mean-square error), 95% data coverage within prediction intervals and correlation between observed data and predictions. All validation metrics were calculated on the out-of-sample predictions from the five-fold cross-validation. All validation procedures and corresponding results are provided in Supplementary Tables 17–19 and Supplementary Figs. 20–22.

#### In-sample metrics

To assess the in-sample performance of our models and compare to national-level estimates produced by the GBD study, we generated a suite of diagnostic plots for anemia estimates in each of the regions and countries modeled. To explore residual error over space and time, absolute error (data minus predicted posterior mean estimates at the corresponding grid cells) was produced.

#### Metrics of predictive validity

To assess the predictive validity of our estimates, we validated our models using spatially stratified five-fold out-of-sample cross-validation^60^. To construct each spatial fold, we used a modified bi-tree algorithm to spatially aggregate data points. This algorithm recursively partitions two-dimensional space, alternating between horizontal and vertical splits on the weighted data sample size medians, until the data contained within each spatial partition are of a similar sample size. The depth of recursive partitioning is constrained by the target sample size within a partition and the minimum number of clusters or pseudo-clusters allowed within each spatial partition (in this case, a minimum sample size of 500 was used). These spatial partitions are then allocated to one of five folds for cross-validation. For validation, each geostatistical model was run five times, each time holding out data from one of the folds, generating a set of out-of-sample predictions for the held-out data. A full suite of out-of-sample predictions over the entire dataset was generated by combining the out-of-sample predictions from the five cross-validation runs.

Using these out-of-sample predictions, we then calculated mean error (or bias), root-mean-squared error (RMSE, which summarizes total variance), coefficient of variation (defined to be the standard deviation divided by the mean and multiplied by 100, which is a measure of relative variability) and 95% coverage of our predictive intervals (the proportion of observed out-of-sample data that fall within our predicted 95% credible intervals) aggregated up to different administrative levels (levels 0, 1 and 2) as defined by the GADM^50^. Administrative level 0 (admin 0) borders correspond to national boundaries; administrative level 1 (admin 1) borders generally correspond to regions, provinces or state-level boundaries within a country; and administrative level 2 (admin 2) borders correspond to the next finer subdivision, often districts, within regions. These metrics are summarized in Supplementary Tables 13–15 and Supplementary Figs. 15–17 and are calculated across all regions. Included in the sample tables for comparison are the same metrics calculated on in-sample predictions.

### Sensitity analysis

We ran four five-fold cross-validation holdout in-sample experiments, using different combinations of covariates and random effects:

1. Raw covariates + Gaussian process: ${{{\mathrm{logit}}}}\left( {{{{\mathrm{p}}}}_{{{\mathrm{i}}}}} \right) = {\upbeta}_0 + {{{\mathrm{X}}}}_{{{\mathrm{i}}}}{\upbeta}_{{{{\mathrm{raw}}}}} + \epsilon _{{{{\mathrm{GP}}}}_{{{\mathrm{i}}}}} + \epsilon _{{{\mathrm{i}}}}$
2. Raw covariates: $${{{\mathrm{logit}}}}\left( {{{{\mathrm{p}}}}_{{{\mathrm{i}}}}} \right) = {\upbeta}_0 + {{{\mathrm{X}}}}_{{{\mathrm{i}}}}{\upbeta}_{{{{\mathrm{raw}}}}} + \epsilon _{{{\mathrm{i}}}}$$
3. Stacking predictions as covariates: ${{{\mathrm{logit}}}}\left( {{{{\mathrm{p}}}}_{{{\mathrm{i}}}}} \right) = {\upbeta}_0 + {{{\mathrm{X}}}}_{{{\mathrm{i}}}}{\upbeta}_{{{{\mathrm{stack}}}}} + \epsilon _{{{\mathrm{i}}}}$
4. Stacking covariates + Gaussian process (standard model): ${{{\mathrm{logit}}}}\left( {{{{\mathrm{p}}}}_{{{\mathrm{i}}}}} \right) = {\upbeta}_0 + {{{\mathrm{X}}}}_{{{\mathrm{i}}}}{\upbeta}_{{{{\mathrm{stack}}}}} + \epsilon _{{{{\mathrm{GP}}}}_{{{\mathrm{i}}}}} + \epsilon _{{{\mathrm{i}}}}$

The summary error measures for all models are shown in Supplementary Figs. 20 and 21 to demonstrate how adding stackers or the Gaussian process individually change predictive capacity on administrative level 1 and 2, respectively. Across the two levels of aggregation and all four validation metrics, the models with a Gaussian process outperformed those without, as they had smaller RMSE and greater correlation. For the standard model, which used both the stacking covariates and the Gaussian process, the in-sample RMSE and correlation were 0.053 and 0.078 and 0.87 and 0.77 at administrative levels 1 and 2, respectively. For the raw covariates model with the Gaussian process, RMSE = 0.069 and 0.084, and the correlation = 0.71 and 0.63; for the model that used raw covariates only, RMSE = 0.066 and 0.091, and the correlation = 0.55 and 0.43; and for the stacked covariates model, RMSE = 0.056 and 0.079, and the correlation = 0.85 and 0.75, at administrative levels 1 and 2, respectively.

#### Projections

To compare our estimated rates of improvement in all-anemia prevalence over the 19-year period across different locations, and to assess if locations are on track to meet the WHO GNT for anemia given historical rates of improvement, we performed a simple projection using estimated AROCs applied to the final year of our estimates. Both AROCs and projections were calculated at the draw level to construct uncertainty estimates for both.

For all-anemia prevalence, we calculated AROCs at each administrative-level unit (*a*) by calculating the AROC between each pair of adjacent years, *t*:

$$AROC_{a,t} = {{{\mathrm{logit}}}}\left( {\frac{{p_{a,t}}}{{p_{a,t - 1}}}} \right)$$

We then calculated a weighted AROC for all-anemia by taking a weighted average across the years, where more recent AROCs were given more weight in the average. We defined the weights to be:

$$W_t = \frac{{\left( {t - 2000} \right)^\gamma }}{{{\Sigma}_{2001}^{2018}\left( {t - 2000} \right)^\gamma }},$$

where *γ* may be chosen to give varying amounts of weight across the years. Using the weights and the AROCs between consecutive years, the average AROC across the duration of the study was calculated:

$$AROC_a = \left( {\mathop {\sum }\limits_{2001}^{2018} W_t \ast AROC_{a,t}} \right)$$

Finally, we calculated the projections (*Proj*) by applying the 7 years of the AROC (from 2018 to 2025) to our mean 2018 prevalence estimates. The projection was performed in logit-space (consistent with the AROC calculation) to ensure that the projected estimates range between 0 and 1:

$$Proj_{a,2025} = {{{\mathrm{logit}}}}^{ - 1}\left( {{{{\mathrm{logit}}}}\left( {p_{a,2018}} \right) + AROC_a \times 7} \right).$$

This projection scheme is analogous to the methods used in the GBD 2017 measurement of progress and projected attainment of health-related SDGs^18^. The exponential power in the weighting scheme was chosen to match that used by the GBD study, which selects this parameter using an out-of-sample predictive validation framework. Our projections assume that areas will sustain the current AROC, and the precision of the AROC estimates is dependent on this assumption and the uncertainty from the all-anemia annual prevalence estimates.

### Post-estimation calibration to national and subnational estimates

To leverage national-level data that were included in GBD 2017 (ref. ^18^) but were outside the scope of our current geospatial modeling framework, and to ensure alignment between this study’s estimates and GBD 2017 estimates, we performed a post hoc calibration to each of our 1,000 candidate maps. For each posterior draw, we calculated population-weighted grid cell aggregations at the level of GBD estimates (at national or subnational level) and compared these estimates in each year to the analogous and available GBD 2017 estimates from 2000 to 2017. We defined the raking factor to be the ratio between the GBD 2017 estimates and our current estimates and linearly interpolated raking factors in each country between the available years. Finally, we multiplied each of our grid cells in a country-year by its associated raking factor. This ensures alignment between our geospatial estimates and GBD 2017 estimates while preserving our estimated within-country geospatial and temporal variation.

### Reporting Summary

Further information on research design is available in the Nature Research Reporting Summary linked to this article.

## Online content

Any methods, additional references, Nature Research reporting summaries, source data, extended data, supplementary information, acknowledgements, peer review information; details of author contributions and competing interests; and statements of data and code availability are available at 10.1038/s41591-021-01498-0.

## Supplementary information

Supplementary Information Supplementary Figs. 1–21, Supplementary Tables 1–18, GATHER compliance, Supplementary Data, Supplementary Covariates, Supplementary Methods, Model Results, Model Validation and Supplementary References

Reporting Summary

## Figures and Tables

**Extended Data Fig. 4:**
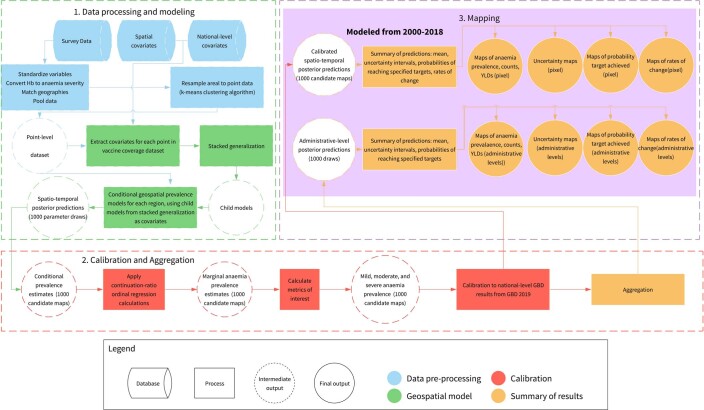
Modeling process flow diagram. The geospatial modelling process consists of three main sections. First, all available survey data that can be referenced to a coordinate/point (for example, survey cluster) or small polygon (administrative) unit are compiled, Hb measurements are converted to anaemia severity, data matched to polygons are resampled into pseudo-points, covariates are merged onto the point-level dataset, a series of conditional geospatial model is fit using stacked generalization child models as main effects is fit for each geographic region, and 1000 posterior predictions are sampled from the fitted model. Second, the 1000 parameter draws are projected into 1000 5x5km pixel conditional prevalence candidate maps, converted into marginal anaemia severity prevalence maps, calibrated to GBD 2019 estimates, and aggregated to administrative levels. Lastly, the 1000 calibrated pixel and aggregated draws are summarized into estimates of anaemia prevalence, counts, YLDs, and the associated uncertainty, probabilities of meeting targets, and rates of change. See the Methods for more details.

## Data Availability

The findings of this study are supported by data available in public online repositories, data publicly available upon reasonable request of the data provider and data not publicly available owing to restrictions by the data provider. Non-publicly available data were used under licence for the current study but might be available from the authors upon reasonable request and with permission of the data provider. A detailed table of data sources and availability can be found in Supplementary Section 2. The full list of input data sources and output of the analyses is publicly available in the Global Health Data Exchange (http://ghdx.healthdata.org/record/ihme-data/global-anemia-prevalence-geospatial-estimates-2000-2019) and can further be explored via customized data visualization tools (https://vizhub.healthdata.org/lbd/anemia). Administrative boundaries were retrieved from the Database of Global Administrative Areas^50^. Land cover was retrieved from the online Data Pool, courtesy of the NASA EOSDIS Land Processes Distributed Active Archive Center, USGS/Earth Resources Observation and Science Center^43^. Lakes were retrieved from the Global Lakes and Wetlands Database^45^, courtesy of the World Wildlife Fund and the Center for Environmental Systems Research at the University of Kassel^44^. Populations were retrieved from WorldPop^46,47^.

## Code availability

This study follows the Guidelines for Accurate and Transparent Health Estimates Reporting (GATHER; Supplementary Table 1). All code used for these analyses is publicly available at http://ghdx.healthdata.org/record/ihme-data/global-anemia-prevalence-geospatial-estimates-2000-2019 and https://github.com/ihmeuw/lbd/tree/anemia-lmic-2021.
