# Supplementary material for: Anemia prevalence in women of reproductive age in low- and middle-income countries between 2000 and 2018
Source: Nat Med. 2021 Oct 12;27(10):1761–82. doi: 10.1038/s41591-021-01498-0 (PMC8516651; doi:10.1038/s41591-021-01498-0)
Supplement: Supplementary file 1 — Supplementary Figs. 1–21, Supplementary Tables 1–18, GATHER compliance, Supplementary Data, Supplementary Covariates, Supplementary Methods, Model Results, Model Validation and Supplementary References [file 41591_2021_1498_MOESM1_ESM.pdf]

nature portfolio

https://doi.org/10.1038/s41591-021-01498-0

Supplementary information

Anemia prevalence in women of reproductive age in low- and middle-income countries between 2000 and 2018

In the format provided by the authors and unedited

Supplementary Information

Contents

Supplementary Figures 2
Supplementary Tables 3
1.0 GATHER compliance 4
2.0 Supplementary data 6
3.0 Supplementary covariates 20
4.0 Supplementary methods 24
4.1 Geostatistical model 24
5.0 Model result 26
6.0 Model validation 174
6.1 Anaemia validation metrics 174
6.2 Anaemia sensitivity analysis 180
7.0 Supplementary references 182

Supplementary Figures

Supplementary Figure 1: Anaemia data availability by type and country, 1998–2018, in Africa 13
Supplementary Figure 2: Anaemia data availability by type and country, 1998–2018, in Central America and the Caribbean and South America 14
Supplementary Figure 3: Anaemia data availability by type and country, 1998–2018, in Southeast Asia. 15
Supplementary Figure 4: Anaemia data availability by type and country, 1998–2018, in South Asia ... 16
Supplementary Figure 5: Anaemia data availability by type and country, 1998–2018, in Middle East and Central Asia 17
Supplementary Figure 6: Years of data informing administrative level one units 18
Supplementary Figure 7: Years of data informing administrative level two units 19
Supplementary Figure 8: Covariates. 23
Supplementary Figure 9: Finite elements mesh 25
Supplementary Figure 10: Anaemia posterior means and upper and lower 95% uncertainty intervals 167
Supplementary Figure 11: Mild anaemia posterior means and upper and lower 95% uncertainty intervals 168
Supplementary Figure 12: Moderate anaemia posterior means and upper and lower 95% uncertainty intervals 169
Supplementary Figure 13: Severe anaemia posterior means and upper and lower 95% uncertainty intervals 170
Supplementary Figure 14: Prevalence for overall anaemia among WRA in 2018, and probability of achieving the WHO GNT for overall anaemia by 2018 171
Supplementary Figure 15: Prevalence for overall anaemia among WRA in 2025, and probability of achieving the WHO GNT for overall anaemia by 2025 172
Supplementary Figure 16: Overlapping population-weighted quartiles of overall anaemia and relative 95% uncertainty in 2018 173
Supplementary Figure 17: Anaemia admin 0 aggregation in-sample 177
Supplementary Figure 18: Anaemia admin 1 aggregation in-sample 178
Supplementary Figure 19: Anaemia admin 2 aggregation in-sample 179
Supplementary Figure 20: Sensitivity analysis results admin 1 level 180
Supplementary Figure 21: Sensitivity analysis results admin 2 level 181

Supplementary Tables

Supplementary Table 1: Guidelines for Accurate and Transparent Health Estimates Reporting (GATHER) checklist 4
Supplementary Table 2: Summary of data source geographical resolution, and haemoglobin assessment methods 6
Supplementary Table 3: The low- and middle-income countries included in model of prevalence of anaemia from 2000 to 2018 7
Supplementary Table 4: The low- and middle-income countries excluded in model of prevalence of anaemia from 2000 to 2018 10
Supplementary Table 5: Altitude adjustment to measured haemoglobin concentrations[37] 11
Supplementary Table 6[24]: Smoking status adjustments to measured haemoglobin concentrations[37]. 11
Supplementary Table 7: Smoking and elevation adjusted haemoglobin level definitions to diagnose anaemia (g/L)[37] 12
Supplementary Table 8: Covariates used in mapping 20
Supplementary Table 9: Spatial hyperparameter priors by region 24
Supplementary Table 10: Widest absolute inequalities between subnational districts in 2000 and 2018 26
Supplementary Table 11: Widest relative inequalities: LMICs with districts deviating ≥50% from the national mean in 2000 and 2018 27
Supplementary Table 12: Shifts in the number of WRA with anaemia by districts in 2000 and 2018 ... 28
Supplementary Table 13: LMICs with extreme differences in their rates of change across subnational districts 29
Supplementary Table 14: Countries and administrative units achieving the WHO GNT of reducing anaemia prevalence among WRA by 50% prevalence by 2018 and 2030 (with a baseline year of 2012) with high probability (>95%). 30
Supplementary Table 15: Anaemia fitted parameters 165
Supplementary Table 16: Predictive metrics for anaemia aggregated to admin 0 174
Supplementary Table 17: Predictive metrics for anaemia aggregated to admin 1 175
Supplementary Table 18: Predictive metrics for anaemia aggregated to admin 2 176

1.0 GATHER compliance

Supplementary Table 1: Guidelines for Accurate and Transparent Health Estimates Reporting (GATHER) checklist

Item #	Checklist item	Reported on page #
**Objectives and funding**		
**1**	Define the indicator(s), populations (including age, sex, and geographic entities), and time period(s) for which estimates were made.	Main text: Introduction, Methods (Data)
**2**	List the funding sources for the work.	Main text: Acknowledgments
**Data Inputs**		
For all data inputs from multiple sources that are synthesized as part of the study:		
**3**	Describe how the data were identified and how the data were accessed.	Main text: Methods (Data, Data availability), Supplementary section 2.0
**4**	Specify the inclusion and exclusion criteria. Identify all ad-hoc exclusions.	Main text: Methods (Data exclusion criteria), Supplementary Information: 2.0
**5**	Provide information on all included data sources and their main characteristics. For each data source used, report reference information or contact name/institution, population represented, data collection method, year(s) of data collection, sex and age range, diagnostic criteria or measurement method, and sample size, as relevant.	Supplementary Information: 2.0
**6**	Identify and describe any categories of input data that have potentially important biases (e.g., based on characteristics listed in item 5).	Main text: Methods (Limitations)
For data inputs that contribute to the analysis but were not synthesized as part of the study:		
**7**	Describe and give sources for any other data inputs.	Main text: Methods (Data: Spatial covariates), Supplementary Information: 2.0 Supplementary covariates
For all data inputs:		
**8**	Provide all data inputs in a file format from which data can be efficiently extracted (e.g., a spreadsheet rather than a PDF), including all relevant meta-data listed in item 5. For any data inputs that cannot be shared because of ethical or legal reasons, such as third-party ownership, provide a contact name or	(GHDx link available upon publication) Supplementary Information: 2.0

	the name of the institution that retains the right to the data.	
**Data analysis**		
**9**	Provide a conceptual overview of the data analysis method. A diagram may be helpful.	Main text: Methods (Data)
**10**	Provide a detailed description of all steps of the analysis, including mathematical formulae. This description should cover, as relevant, data cleaning, data pre-processing, data adjustments and weighting of data sources, and mathematical or statistical model(s).	Main text: Methods (Analysis), Supplementary Information: 4.1 Geostatistical model.
**11**	Describe how candidate models were evaluated and how the final model(s) were selected.	Main text: Methods (Analysis: Model validation), Supplementary Information: 5.0 Model Result and 6.0 Model validation.
**12**	Provide the results of an evaluation of model performance, if done, as well as the results of any relevant sensitivity analysis.	Main text: Methods (Analysis), Supplementary Information: 5.0 Model Result and 6.0 Model validation.
**13**	Describe methods for calculating uncertainty of the estimates. State which sources of uncertainty were, and were not, accounted for in the uncertainty analysis.	Main text: Methods (Analysis: Geostatistical model), Supplementary Information: 5.0 Model Result and 6.0 Model validation.
**14**	State how analytic or statistical source code used to generate estimates can be accessed.	(GHDx link available upon publication)
**Results and Discussion**		
**15**	Provide published estimates in a file format from which data can be efficiently extracted.	Raster files for spatial data and CSVs of estimates available at (GHDx link available upon publication)
**16**	Report a quantitative measure of the uncertainty of the estimates (e.g., uncertainty intervals).	Supplementary Information: 5.0 Model Result and 6.0 Model validation.
**17**	Interpret results in light of existing evidence. If updating a previous set of estimates, describe the reasons for changes in estimates.	Main text: Results and Discussion sections.
**18**	Discuss limitations of the estimates. Include a discussion of any modelling assumptions or data limitations that affect interpretation of the estimates.	Main text: Methods (Limitations)

2.0 Supplementary data

We modelled the prevalence of anaemia among women of reproductive age (WRA; ages 15–49) in 82 low- and middle-income countries (LMICs) from 2000 to 2018, and stratified by anaemia severity. These countries were determined by their Socio-demographic Index (SDI), a summary measure of development which combines education, fertility, and poverty, developed and computed by the Global Burden of Disease (GBD) study[1]. Malaysia, China, and Iran were included despite high-middle SDI status to create better geographic continuity. Albania, Bosnia-Herzegovina, North Korea, and Moldova were excluded despite their middle SDI status due to geographic discontinuity with other included countries and lack of available survey data. We did not estimate for the island nations of American Samoa, Federated States of Micronesia, Fiji, Kiribati, Marshall Islands, Samoa, Solomon Islands, or Tonga, where no available survey data could be sourced.

The list of data sources used to model anaemia can be found in the Global Health Data Exchange by Number Identification (ID): http://ghdx.healthdata.org/lbd-publication-data-input-sources?field_rec_ihme_publication_tid=29140.

Supplementary Table 2: Summary of data source geographical resolution, and haemoglobin assessment methods

	Microdata (Point) Sources			Administrative (Polygonal) Sources			
	Venous Draw	Capillary Draw	Unknown	Venous Draw	Capillary Draw	Unknown	Total
**Hemocue**	115	5	4	11	20	2	157
**Centrifuge/Hematocrit**	1	0	0	2	1	0	4
**Dried blood spot**	1	0	0	0	1	0	2
**Liquid blood & Spectral**	0	1	0	2	0	2	5
**Unknown**	2	0	5	10	3	22	42
**Total**	119	6	9	25	25	26	210
**Spatial Resolution Total**	134			76			

136 sources gave raw haemoglobin values (135 microdata surveys and 1 administrative data survey) and 74 sources reported categorically binned data (74 administrative data surveys). Country-specific surveys made up 12 of the 135 microdata sources used in

the models, consisting of National Nutrition surveys and Demographic and Family Health surveys from Latin America and Pakistan, and the Indonesia Family Life Survey.

18 of 75 administrative data reports were sourced from the GHDx, and 57 reports were gathered from a systematic literature review of 31,821 search results on PubMed. Testing increasingly targeted search strings against broader search results' accepted sources allowed us to hone the number of papers reviewed down. China's 15,675 search results were narrowed to 2982 using our optimised search string, resulting in more accepted administrative data sources than from any other country.

Supplementary Table 3: The low- and middle-income countries included in model of prevalence of anaemia from 2000 to 2018

Country	SDI	SDI Quintile
Afghanistan	0.2903	Low SDI
Angola	0.4605	Low-middle SDI
Bangladesh	0.4580	Low SDI
Benin	0.3734	Low SDI
Bhutan	0.5699	Low-middle SDI
Bolivia	0.5874	Low-middle SDI
Brazil	0.6633	Middle SDI
Burkina Faso	0.2839	Low SDI
Burundi	0.3097	Low SDI
Cambodia	0.4816	Low-middle SDI
Cameroon	0.4820	Low-middle SDI
Cape Verde	0.5491	Low-middle SDI
Central African Republic	0.3344	Low SDI
Chad	0.2529	Low SDI
China	0.7073	High-middle SDI
Colombia	0.6337	Middle SDI
Côte d'Ivoire	0.4121	Low SDI
Democratic Republic of the Congo	0.3645	Low SDI
Ecuador	0.6356	Middle SDI
Egypt	0.6043	Low-middle SDI

Country	SDI	SDI Quintile
El Salvador	0.5931	Low-middle SDI
Equatorial Guinea	0.6252	Middle SDI
Ethiopia	0.3342	Low SDI
Gabon	0.6506	Middle SDI
Ghana	0.5370	Low-middle SDI
Guatemala	0.5242	Low-middle SDI
Guinea	0.3247	Low SDI
Guinea-Bissau	0.3490	Low SDI
Guyana	0.5837	Low-middle SDI
Haiti	0.4417	Low SDI
Honduras	0.5123	Low-middle SDI
India	0.5502	Low-middle SDI
Indonesia	0.6476	Middle SDI
Iran	0.7001	High-middle SDI
Jordan	0.6968	Middle SDI
Kenya	0.4995	Low-middle SDI
Kyrgyzstan	0.6066	Low-middle SDI
Laos	0.5188	Low-middle SDI
Lesotho	0.4934	Low-middle SDI
Liberia	0.3284	Low SDI
Madagascar	0.3308	Low SDI
Malawi	0.3493	Low SDI
Malaysia	0.7592	High-middle SDI
Mali	0.2669	Low SDI
Mexico	0.6284	Middle SDI
Mongolia	0.6619	Middle SDI
Morocco	0.5792	Low-middle SDI
Mozambique	0.3405	Low SDI
Myanmar	0.5558	Low-middle SDI

Country	SDI	SDI Quintile
Namibia	0.6158	Middle SDI
Nepal	0.4285	Low SDI
Niger	0.1906	Low SDI
Nigeria	0.4934	Low-middle SDI
Pakistan	0.4922	Low-middle SDI
Palestine	0.5414	Low-middle SDI
Papua New Guinea	0.4190	Low SDI
Peru	0.6358	Middle SDI
Republic of the Congo	0.5741	Low-middle SDI
Rwanda	0.4074	Low SDI
Sao Tome and Principe	0.4883	Low-middle SDI
Senegal	0.3730	Low SDI
Sierra Leone	0.3572	Low SDI
Somalia	0.2348	Low SDI
South Africa	0.6765	Middle SDI
Sri Lanka	0.6797	Middle SDI
Sudan	0.4779	Low-middle SDI
Swaziland	0.5777	Low-middle SDI
Tajikistan	0.5226	Low-middle SDI
Tanzania	0.4122	Low SDI
Thailand	0.6843	Middle SDI
The Gambia	0.4048	Low SDI
Timor-Leste	0.5048	Low-middle SDI
Togo	0.4133	Low SDI
Tunisia	0.6754	Middle SDI
Turkmenistan	0.6964	Middle SDI
Uganda	0.3877	Low SDI
Uzbekistan	0.6295	Middle SDI
Venezuela	0.6554	Middle SDI

Country	SDI	SDI Quintile
Vietnam	0.6068	Middle SDI
Yemen	0.4295	Low SDI
Zambia	0.4722	Low-middle SDI
Zimbabwe	0.4632	Low-middle SDI

Supplementary Table 4: The low- and middle-income countries excluded in model of prevalence of anaemia from 2000 to 2018

Country	SDI	SDI Quintile
Algeria	0.6958	Middle SDI
Belize	0.6022	Low-middle SDI
Botswana	0.6632	Middle SDI
Comoros	0.4343	Low SDI
Costa Rica	0.6621	Middle SDI
Cuba	0.6877	Middle SDI
Djibouti	0.4848	Low-middle SDI
Dominican Republic	0.5926	Low-middle SDI
Eritrea	0.4088	Low SDI
French Guiana	NA	NA
Iraq	0.5848	Low-middle SDI
Jamaica	0.6785	Middle SDI
Mauritania	0.4706	Low-middle SDI
Nicaragua	0.5296	Low-middle SDI
Panama	0.6770	Middle SDI
Paraguay	0.6188	Middle SDI
Philippines	0.6172	Middle SDI
South Sudan	0.2747	Low SDI
Suriname	0.6410	Middle SDI
Syria	0.6111	Middle SDI
Trinidad and Tobago	0.6984	Middle SDI
Western Sahara	NA	NA

Country	SDI	SDI Quintile
Albania	NA	NA
Bosnia-Herzegovina	NA	NA
North Korea	NA	NA
Moldova	NA	NA

Supplementary Table 5: Altitude adjustment to measured haemoglobin concentrations[37]

Altitude (metres above sea level)	Measured haemoglobin adjustments (g/L)
< 1000	0
1000	-2
1500	-5
2000	-8
2500	-13
3000	-19
3500	-27
4000	-35
4500	-45

Supplementary Table 6[24]: Smoking status adjustments to measured haemoglobin concentrations[37].

Smoking status	Measured haemoglobin adjustments (g/L)
Non-smoker	0
Smoker (alL)	-0.3
½–1 Packet/day	-0.3

1–2 Packet/day	-0.5
≥2 Packet/day	-0.7

Supplementary Table 7: Smoking and elevation adjusted haemoglobin level definitions to diagnose anaemia (g/L)[37]

Population	Non-anaemic	Mild anaemia	Moderate anaemia	Severe anaemia
Non-pregnant women	≥120	110–119	80–109	<80
Pregnant women	≥110	100–109	70–99	<70

Supplementary Figures 1–5 show the data availability for anaemia in the 82 countries in our analysis. We incorporated data from several survey series, including the Demographic and Health Survey by Macro International (Macro DHS), the Malaria Indicator Survey (MACRO-MIS), United Nations International Children's Emergency Fund (UNICEF MICS), the Pan Arab Programme on Family Health survey by League of Arab States (PAPFAM), the Living Standards Measurement Study by World Bank (LSMS), the Family Life Survey by RAND Corporation (RAND FLS), the Reproductive Health Survey by Center for Disease Control (CDC RHS), the Demographic and Health Survey for Pacific countries funded by the Asian Development Bank (ADB DHS), and country-specific surveys that were not clearly associated with an international survey series. We regularly monitored larger survey series such as Macro DHS and UNICEF MICS for new data. We also included data from scientific literature by conducting a systematic literature review. We searched PubMed for articles published after January 1, 1998 with the following key terms: "haemoglobin", "anaemia", and "haematocrit". First, we did a title/abstract screen of the PubMed search results to remove publications that did not report anaemia prevalence in women of reproductive age. We then did a full text review of the remaining articles to ensure the data met the inclusion criteria. We manually extracted data from the accepted publications into Excel spreadsheets. The database for anaemia consists of 218 geo-referenced household surveys representing over 3 million WRA in LMICs.

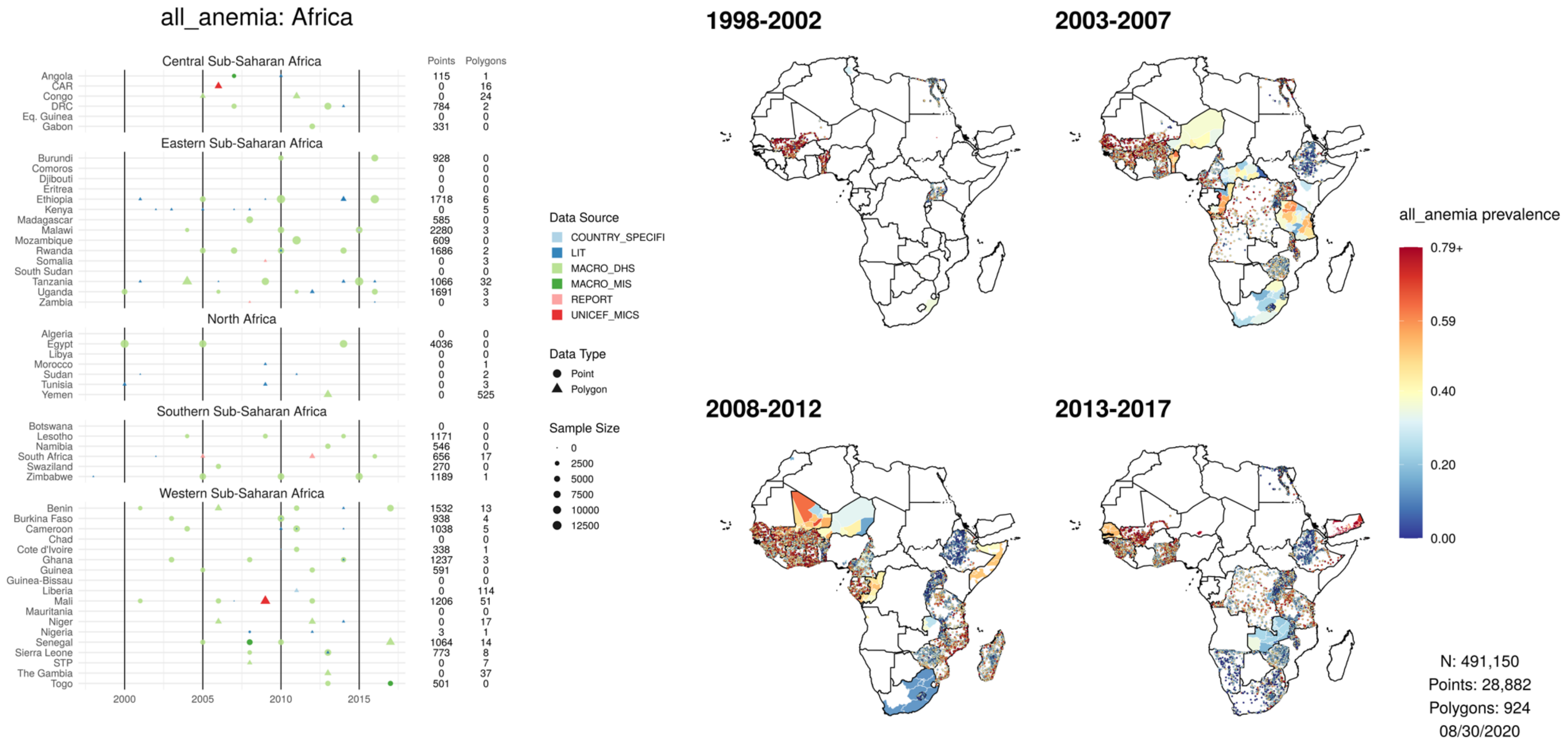

Supplementary Figure 1: Anaemia data availability by type and country, 1998–2018, in Africa

All data are shown by country and year of survey and mapped at their corresponding geo-positioned coordinate or area. The total number of points and polygons (areal) for each country are plotted by data source, type, and sample size. Sample size represents the number of individual microdata records for each survey. Mean anaemia prevalence of the input coordinate or area is mapped.

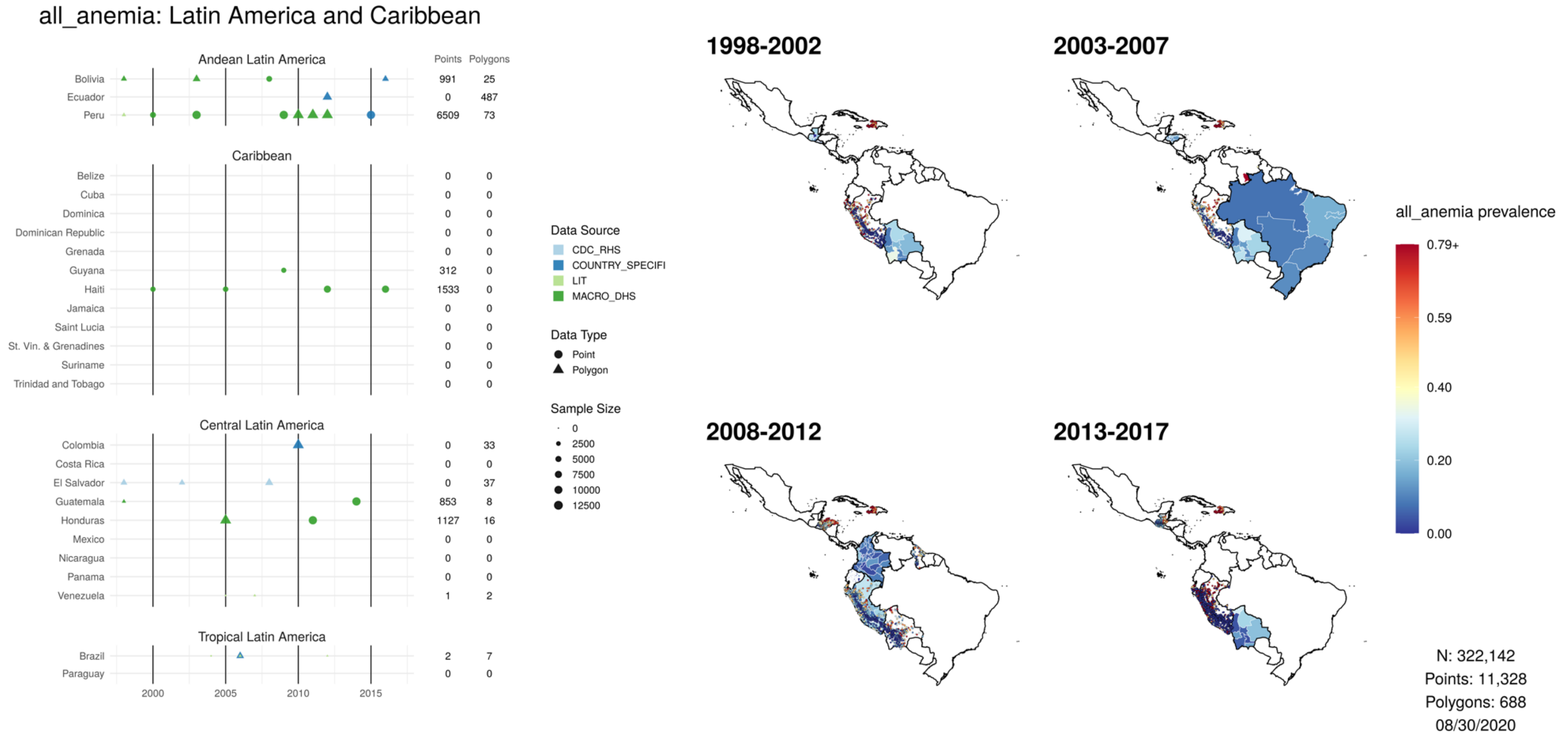

Supplementary Figure 2: Anaemia data availability by type and country, 1998–2018, in Central America and the Caribbean and South America

All data are shown by country and year of survey and mapped at their corresponding geo-positioned coordinate or area. The total number of points and polygons (areal) for each country are plotted by data source, type, and sample size. Sample size represents the number of individual microdata records for each survey. Mean anaemia prevalence of the input coordinate or area is mapped.

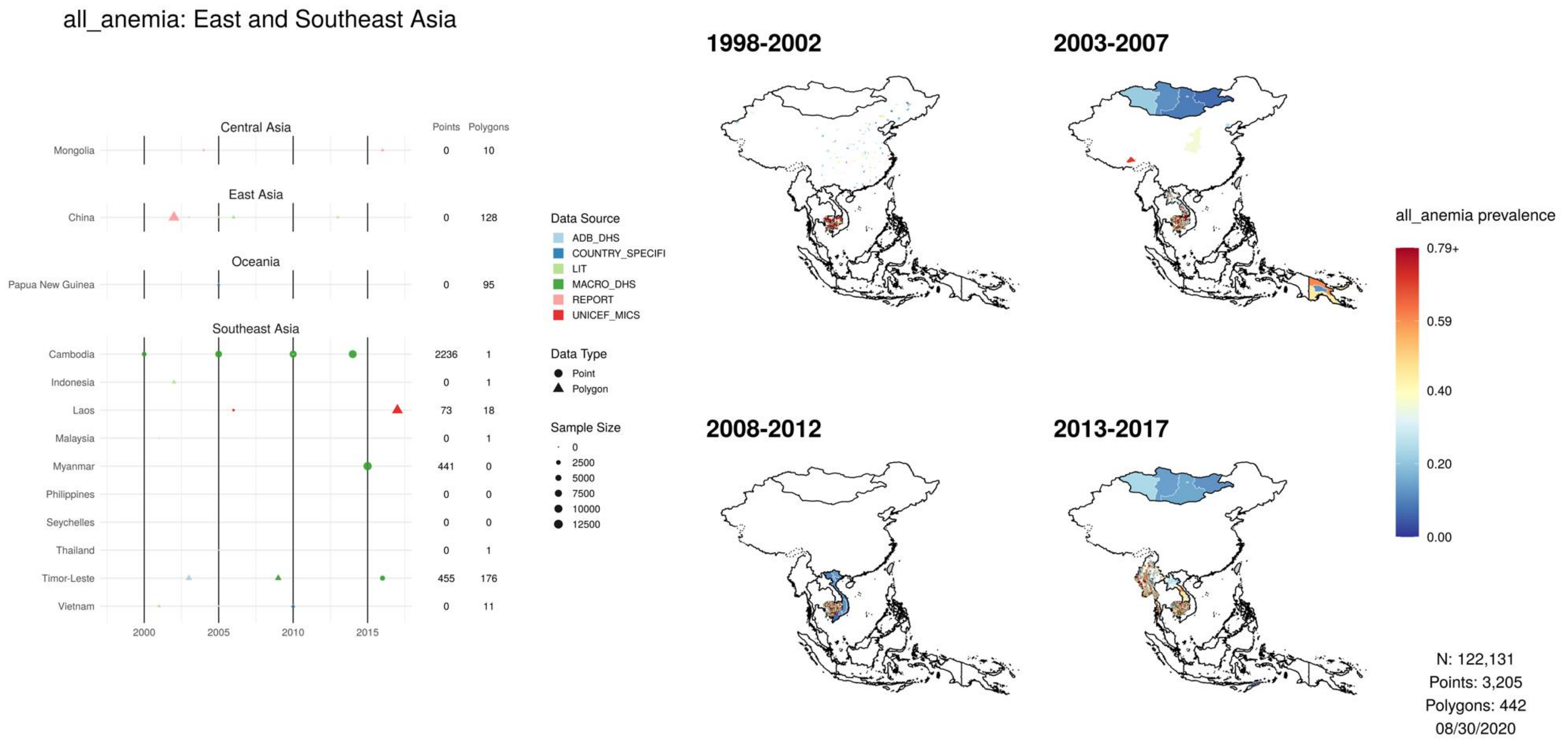

Supplementary Figure 3: Anaemia data availability by type and country, 1998–2018, in Southeast Asia.
All data are shown by country and year of survey and mapped at their corresponding geo-positioned coordinate or area. The total number of points and polygons (areal) for each country are plotted by data source, type, and sample size. Sample size represents the number of individual microdata records for each survey. Mean anaemia prevalence of the input coordinate or area is mapped.

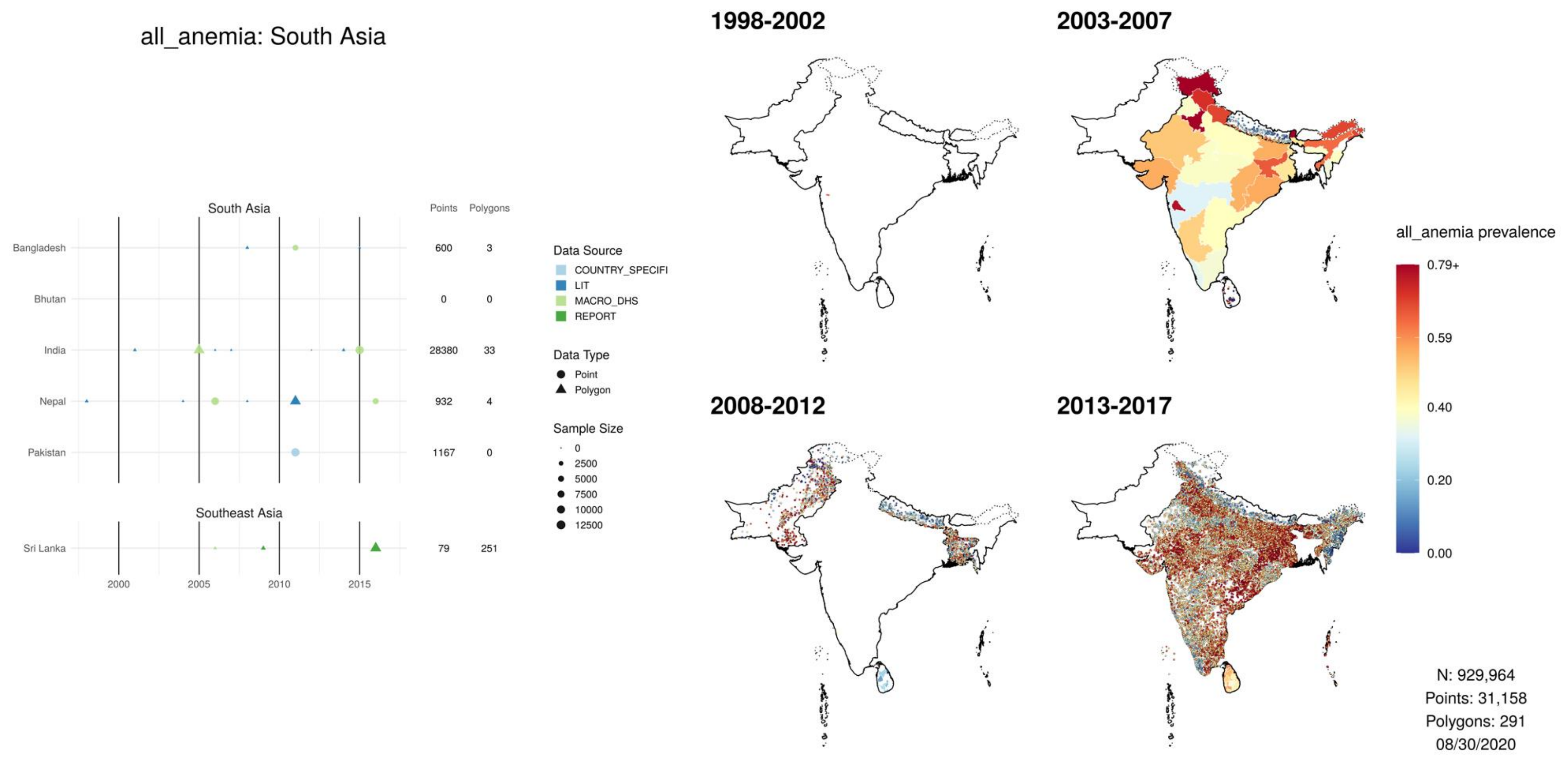

Supplementary Figure 4: Anaemia data availability by type and country, 1998–2018, in South Asia

All data are shown by country and year of survey and mapped at their corresponding geo-positioned coordinate or area. The total number of points and polygons (areal) for each country are plotted by data source, type, and sample size. Sample size represents the number of individual microdata records for each survey. Mean anaemia prevalence of the input coordinate or area is mapped.

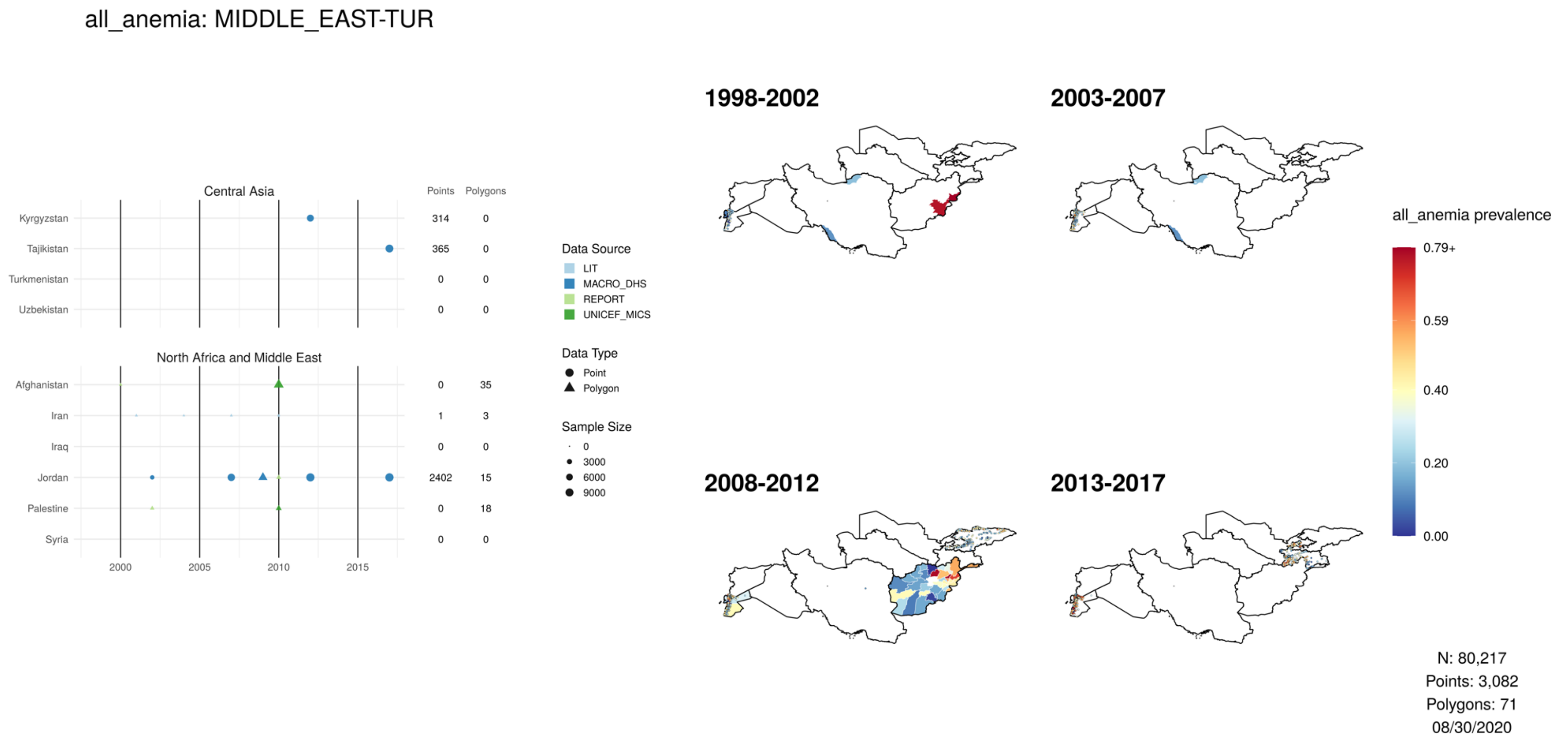

Supplementary Figure 5: Anaemia data availability by type and country, 1998–2018, in Middle East and Central Asia
All data are shown by country and year of survey and mapped at their corresponding geo-positioned coordinate or area. The total number of points and polygons (areal) for each country are plotted by data source, type, and sample size. Sample size represents the number of individual microdata records for each survey. Mean anaemia prevalence of the input coordinate or area is mapped.

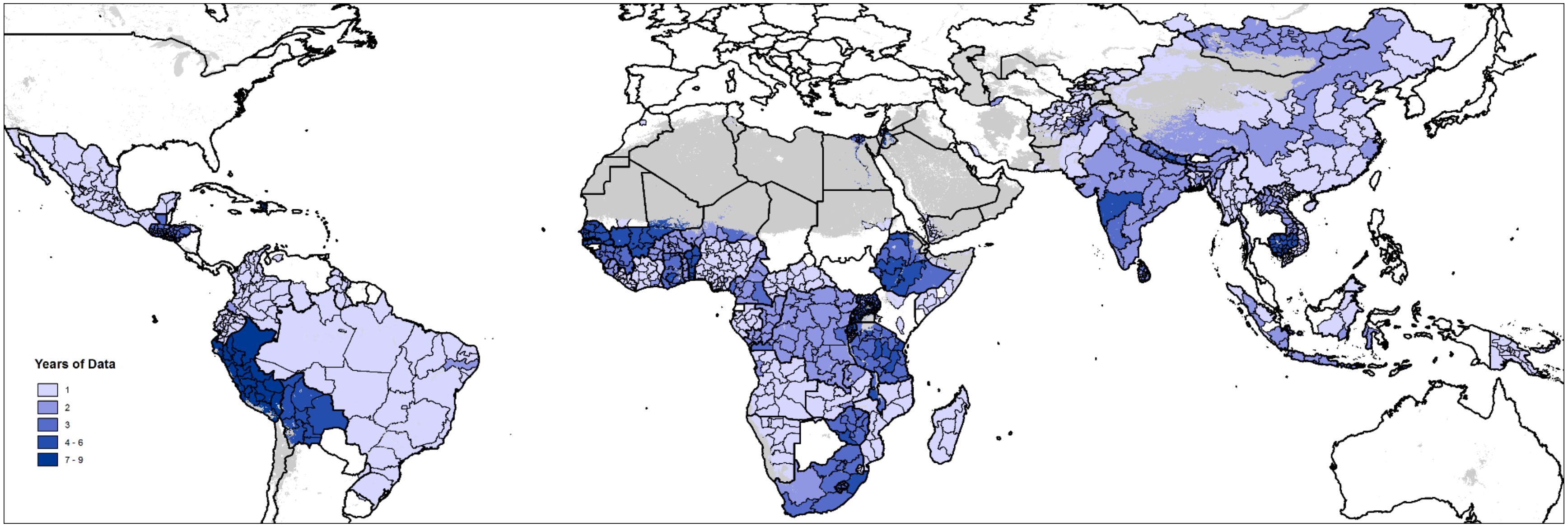

Supplementary Figure 6: Years of data informing administrative level one units

The number of years of extracted data (during 2000–2018) within each administrative level one unit. This figure only illustrates the number of years of observed data and not the sample size or volume of the data. The majority of the data sources contained point-referenced data, and those that did not primarily contained data that was representative for administrative level one polygons.

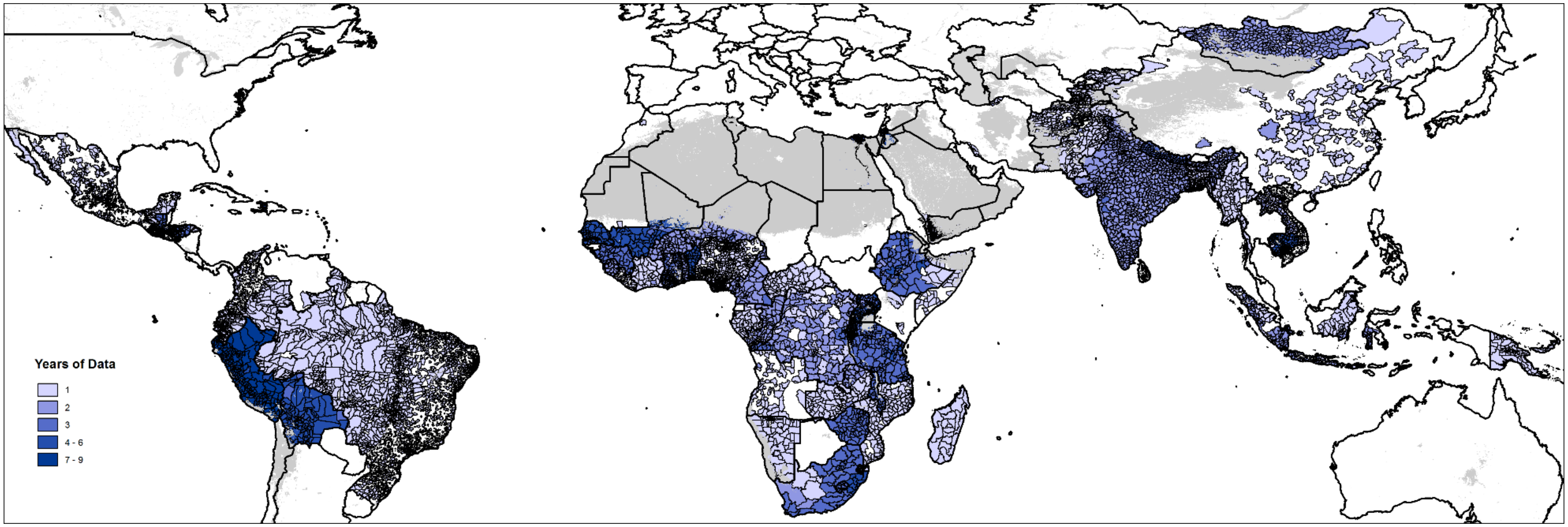

Supplementary Figure 7: Years of data informing administrative level two units

The number of years of extracted data (during 2000–2018) within each administrative level one unit. This figure only illustrates the number of years of observed data and not the sample size or volume of the data. The majority of the data sources contained point-referenced data and those that did not primarily contained data that was representative for administrative level one polygons. Polygonal data was resampled to points, and, as such, this second-level data volume map may suggest more data than was available at each of these administrative units. For a more representative illustration of the data volume used in this study, please see Supplementary Figure 6.

3.0 Supplementary covariates

Supplementary Table 8: Covariates used in mapping

No.	Covariate	Temporal resolution	Source	Reference
**1**	Travel time to nearest settlement >50,000 inhabitants	Static	Big Data Institute, Nuffield Department of Medicine, University of Oxford	Weiss, D. J. et al. A global map of travel time to cities to assess inequalities in accessibility in 2015. Nature 533, 333-336 (2018). Available at: https://forobs.jrc.ec.europa.eu/products/gam/download.php
**2**	Average daily mean temperature	Annual	CRUTS	Harris, I., Jones, P. d., Osborn, T. j. & Lister, D. h. Updated high-resolution grids of monthly climatic observations – the CRU TS3.10 dataset. Int. J. Climatol. 34, 623–642 (2014).<br><br>University of East Anglia. Climatic Research Unit TS v. 3.24 dataset. Available at: https://crudata.uea.ac.uk/cru/data/hrg/cru_ts_3.24.01/. (Accessed: 24th July 2017).
**3**	Average daily mean rainfall (Precipitation)	Annual	CRUTS	Harris, I., Jones, P. d., Osborn, T. j. & Lister, D. h. Updated high-resolution grids of monthly climatic observations – the CRU TS3.10 dataset. Int. J. Climatol. 34, 623–642 (2014).<br><br>University of East Anglia. Climatic Research Unit TS v. 3.24 dataset. Available at: https://crudata.uea.ac.uk/cru/data/hrg/cru_ts_3.24.01/. (Accessed: 24th July 2017).
**4**	Fertility	Annual	WorldPop (derived)	Lloyd, C. T., Sorichetta, A. & Tatem, A. J. High resolution global gridded data for use in population studies. Sci. Data 4, sdata20171 (2017).<br><br>World Pop. Get data. Available at: http://www.worldpop.org.uk/data/get_data/. (Accessed: 25th July 2017)

No.	Covariate	Temporal resolution	Source	Reference
**5**	Nutritional yield for vitamin A	Static	Herrero et al (modelled)	Herrero, M. et al. Farming and the geography of nutrient production for human use: a transdisciplinary analysis. Lancet Planet. Health 1, e33–e42 (2017). Contact M. Herrero for availability.
**6**	Irrigation	Static	University of Frankfurt	Goethe-Universität. Generation of a digital global map of irrigation areas. Available at: https://www.uni-frankfurt.de/45218039/Global_Irrigation_Map. (Accessed: 25th July 2017)
**7**	Malaria incidence	Annual	Malaria Atlas Project	Bhatt, S. et al. The effect of malaria control on Plasmodium falciparum in Africa between 2000 and 2015. Nature 526, 207–211 (2015). Available at: https://malariaatlas.org/malaria-burden-data-download/
**8**	Population	Annual	WorldPop	Lloyd, C. T., Sorichetta, A. & Tatem, A. J. High resolution global gridded data for use in population studies. Sci. Data 4, sdata20171 (2017).<br><br>World Pop. Get data. Available at: http://www.worldpop.org.uk/data/get_data/. (Accessed: 25th July 2017)
**9**	Nutritional yield for iron	Static	Herrero et al 2017	Herrero, M. et al. Farming and the geography of nutrient production for human use: a transdisciplinary analysis. Lancet Planet. Health 1, e33–e42 (2017). Contact M. Herrero for availability.
**10**	Nutritional yield for zinc	Static	Herrero et al 2017	Herrero, M. et al. Farming and the geography of nutrient production for human use: a transdisciplinary analysis. Lancet Planet. Health 1, e33–e42 (2017). Contact M. Herrero for availability.
**11**	Land cover	Annual	MODIS	https://lpdaac.usgs.gov/products/mcd12q1v006/

No.	Covariate	Temporal resolution	Source	Reference
**12**	Particulate matter in cubic micrograms (outdoor air pollution)	Annual	Ambient air pollution data (pm 2.5) as modelled for GBD	Shaddick, Gavin, et al. "Data integration model for air quality: a hierarchical approach to the global estimation of exposures to ambient air pollution." Journal of the Royal Statistical Society: Series C (Applied Statistics) 67.1 (2018): 231-253. Please contact the corresponding author for availability.
**13**	Multi-source Weighted-Ensemble Precipitation	Annual	https://data.princetonclimate.com/opendap	Beck, H.E., A.I.J.M. van Dijk, V. Levizzani, J. Schellekens, D.G. Miralles, B. Martens, A. de Roo: MSWEP: 3-hourly 0.25 global gridded precipitation (1979-2015) by merging gauge, satellite, and reanalysis data, Hydrology and Earth System Sciences, 21(1), 589-615, 2017. Available at: http://www.gloh2o.org/
**14**	Tassled Cap Brightness	Annual	MODIS	https://modis.gsfc.nasa.gov/data/dataprod/mod43.php
**15**	Prevalence of underweight	Annual	Kinyoki et al 2020	Kinyoki, D.K., Osgood-Zimmerman, A.E., Pickering, B.V. et al. Mapping child growth failure across low- and middle-income countries. Nature 577, 231–234 (2020). Available at: http://ghdx.healthdata.org/record/ihme-data/lmic-child-growth-failure-geospatial-estimates-2000-2017

211

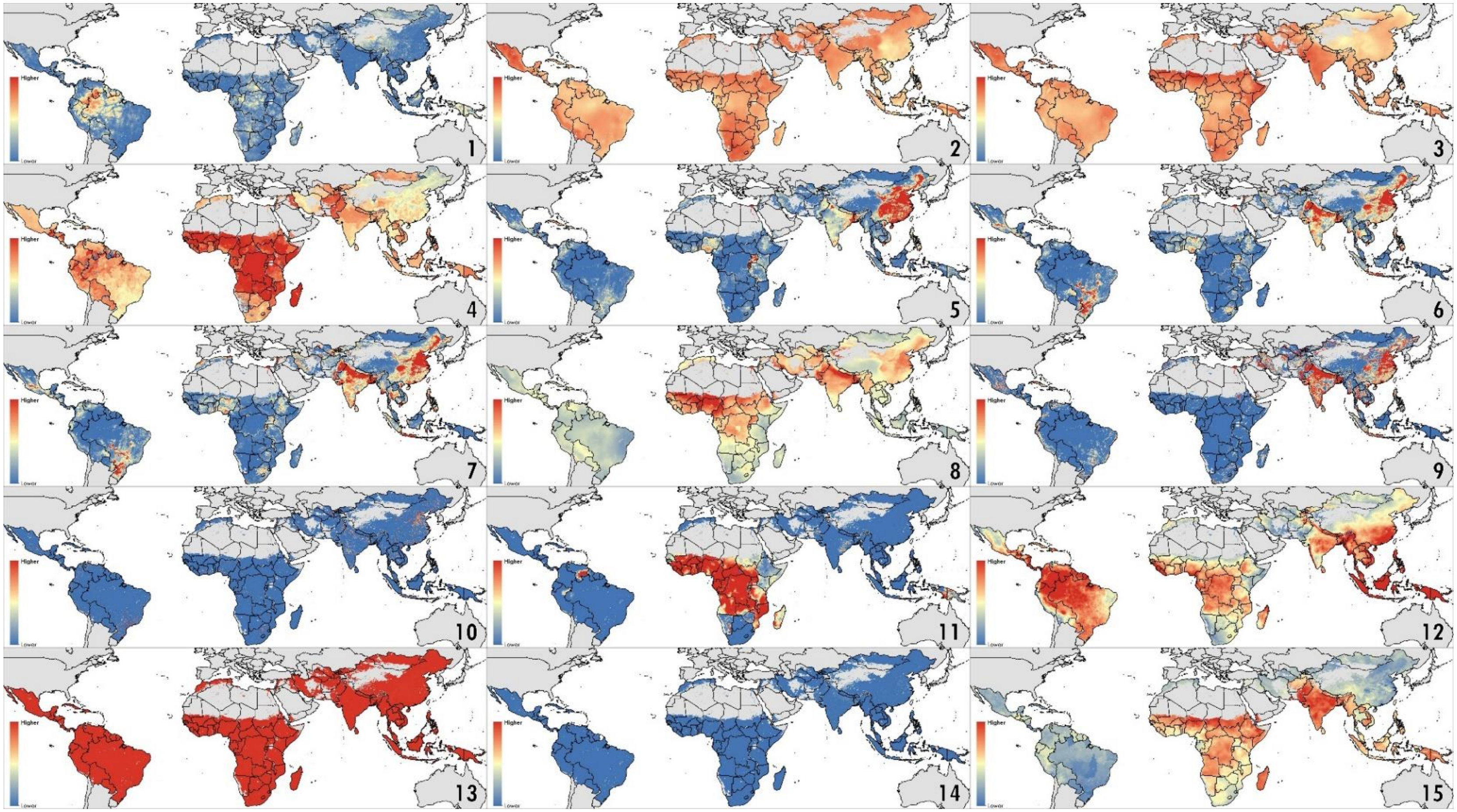

Supplementary Figure 8: Covariates.

Fifteen covariate raster layers of possible socioeconomic and environmental correlates of anaemia in LMICs were used as inputs for the stacking modelling process. Time-varying covariates are presented for the year 2018. For the year of production of non-time-varying covariates and additional details, please refer to the individual covariate citation in Supplementary Table 4: **1.** Travel time to nearest settlement >50,000 inhabitants, **2.** Average daily mean temperature, **3.** Average daily mean rainfall (Precipitation), **4**. Fertility, **5.** Nutritional yield for vitamin A, **6.** Irrigation, **7.** Malaria incidence, **8.** Population, **9.** Nutritional yield for iron, **10.** Nutritional yield for zinc, **11.** Land cover, **12.** Particulate matter in cubic micrograms (outdoor air pollution), **13.** Multi-source Weighted-Ensemble Precipitation, **14.** Tassled Cap Brightness, **15.** Prevalence of underweight.

4.0 Supplementary methods

4.1 Geostatistical model

Supplementary Table 9: Spatial hyperparameter priors by region

Region	μ_{θ_1}	$\sigma^2_{\theta_1}$	μ_2	$\sigma^2_{\theta_2}$
Andean South America	-4.08543	10	2.819916	10
Central America and the Caribbean	-4.02711	10	2.761601	10
Central sub-Saharan Africa	-4.29914	10	3.033632	10
East Asia	-4.02392	10	2.758405	10
Eastern sub-Saharan Africa	-3.96253	10	2.697021	10
Middle East	-4.65437	10	3.388858	10
North Africa	-3.9573	10	2.691784	10
Southeast Asia and Oceania	-3.9555	10	2.689985	10
South and Central Asia	-4.50051	10	3.234994	10
Southern sub-Saharan Africa	-4.34465	10	3.079134	10
Tropical South America	-4.61222	10	3.34671	10
Western sub-Saharan Africa	-4.57494	10	3.309432	10

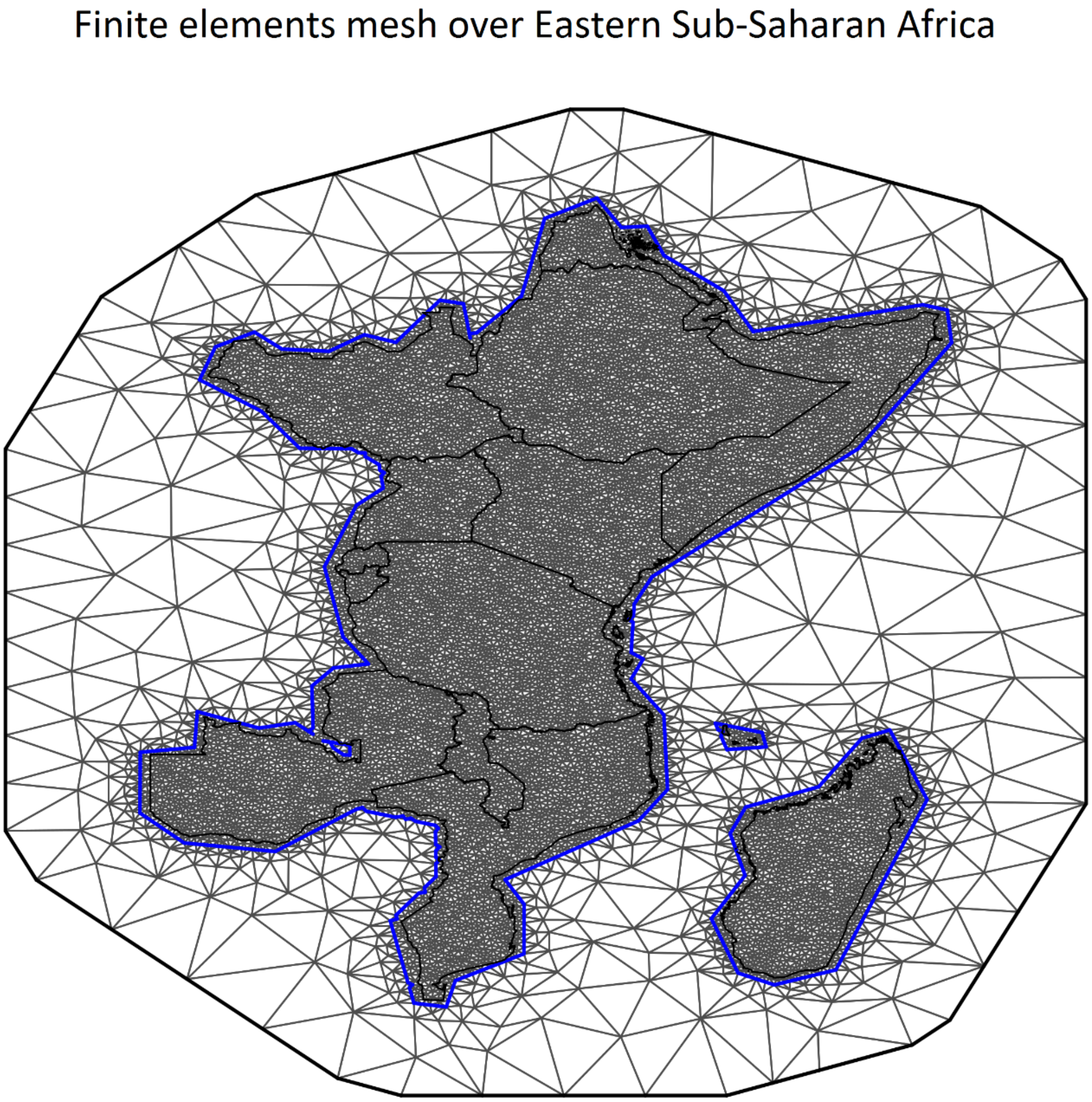

Supplementary Figure 9: Finite elements mesh

The finite elements mesh used to fit the space-time correlated error for the Eastern sub-Saharan Africa (ESSA) region overlaid on the countries in ESSA. Both the fine-scale mesh over land in the modelling region and the coarser buffer region mesh are shown. The simplified region polygon used to determine the boundary for the modelling region is shown in blue.

5.0 Model result

Supplementary Table 10: Widest absolute inequalities between subnational districts in 2000 and 2018

2000		2018	
≥3-fold difference (17)	≥6-fold difference (4)	≥3-fold difference (26)	≥6-fold difference (7)
Afghanistan	Afghanistan	Afghanistan	Bolivia
Bolivia	Iran	Bolivia	Colombia
Brazil	Mexico	Brazil	Ethiopia
Burundi	Vietnam	Burundi	Honduras
China		China	Kenya
Colombia		Colombia	Mexico
Egypt		Democratic Republic of the Congo	Venezuela
El Salvador		Egypt	
Ethiopia		El Salvador	
Honduras		Ethiopia	
India		Haiti	
Iran		Honduras	
Kenya		India	
Mexico		Indonesia	
Uganda		Kenya	
Venezuela		Mexico	
Vietnam		Niger	
		Nigeria	
		Papua New Guinea	
		Rwanda	
		South Africa	
		Tajikistan	
		Tanzania	
		Thailand	
		Venezuela	
		Vietnam	

Supplementary Table 11: Widest relative inequalities: LMICs with districts deviating ≥50% from the national mean in 2000 and 2018

2000 (27 LMICs)	2018 (36 LMICs)
Afghanistan	Afghanistan
Burundi	Burundi
Bolivia	Bolivia
Brazil	Brazil
China	China
Colombia	Colombia
Democratic Republic of the Congo	Democratic Republic of the Congo
Ecuador	Ecuador
Egypt	Egypt
El Salvador	El Salvador
Ethiopia	Ethiopia
Guyana	Guatemala
Honduras	Haiti
Haiti	Honduras
Indonesia	Indonesia
India	India
Iran	Iran
Kenya	Kenya
Mexico	Laos
Peru	Mexico
Rwanda	Mongolia
Sudan	Nepal
Thailand	Niger
Tanzania	Nigeria
Uganda	Papua New Guinea
Venezuela	Peru
Vietnam	Rwanda
	South Africa
	Tajikistan

	Tanzania<br>Thailand<br>Timor-Leste<br>Uganda<br>Venezuela<br>Vietnam<br>Zimbabwe

Supplementary Table 12: Shifts in the number of WRA with anaemia by districts in 2000 and 2018

WRA with anaemia	Number of districts in 2000	Number of districts in 2018
<5,000	66.8% (14643 of 21917)	65.2% (14292 of 21917)
5,000–14,999	17.7% (3879 of 21917)	14.6% (3199 of 21917
15,000–49,999	8.8% (1926 of 21917	12.4% (2716 of 21917)
50,000–150,000	3.8% (823 of 21917)	4.5% (991 of 21917)
>150,000	3.1% (678 of 21917)	3.4% (751 of 21917)

Supplementary Table 13: LMICs with extreme differences in their rates of change across subnational districts

LMICs with at least 2.5% annualized decreases and increases across their districts (21)	LMICs with at least 5% annualized decreases and increases across their districts (21)
Afghanistan	Guatemala
Bolivia	
Burkina Faso	
Central African Republic	
Ecuador	
Guatemala	
Honduras	
India	
Indonesia	
Iran	
Laos	
Mexico	
Niger	
Nigeria	
Peru	
South Africa	
Sri Lanka	
Tajikistan	
Thailand	
Timor-Leste	
Venezuela	

Supplementary Table 14: Countries and administrative units achieving the WHO GNT of reducing anaemia prevalence among WRA by 50% prevalence by 2018 and 2030 (with a baseline year of 2012) with high probability (>95%).
This table is sorted separately for 2018 and 2030 by region, within region by country, within country by province, and within province by district. If District is NA, then the row refers to a Province, and if both District and Province is NA, then the row refers to a Country.

	2018			2030		
**Region**	**Country**	**Province**	**District**	**County**	**Province**	**District**
**Andean South America**	Bolivia	Chuquisaca	NA	Bolivia	Chuquisaca	NA
**Andean South America**	Bolivia	Chuquisaca	Oropeza	Bolivia	Chuquisaca	Azurduy
**Andean South America**	Bolivia	Chuquisaca	Yamparaez	Bolivia	Chuquisaca	Belisario Boeto
**Andean South America**	Bolivia	Chuquisaca	Zudanez	Bolivia	Chuquisaca	Hernando Siles
**Andean South America**	Bolivia	Cochabamba	Arani	Bolivia	Chuquisaca	Nor Cinti
**Andean South America**	Bolivia	Cochabamba	Quillacollo	Bolivia	Chuquisaca	Oropeza
**Andean South America**	Bolivia	La Paz	NA	Bolivia	Chuquisaca	Tomina
**Andean South America**	Bolivia	La Paz	Bautista Saavedra	Bolivia	Chuquisaca	Yamparaez
**Andean South America**	Bolivia	La Paz	Camacho	Bolivia	Chuquisaca	Zudanez
**Andean South America**	Bolivia	La Paz	Franz Tamayo	Bolivia	Cochabamba	Arani
**Andean South America**	Bolivia	La Paz	General Jose Manuel Pando	Bolivia	Cochabamba	Campero

**Andean South America**	Bolivia	La Paz	Ingavi	Bolivia	Cochabamba	Chapare
**Andean South America**	Bolivia	La Paz	Larecaja	Bolivia	Cochabamba	Mizque
**Andean South America**	Bolivia	La Paz	Los Andes	Bolivia	Cochabamba	Tiraque
**Andean South America**	Bolivia	La Paz	Manco Kapac	Bolivia	La Paz	NA
**Andean South America**	Bolivia	La Paz	Munecas	Bolivia	La Paz	Bautista Saavedra
**Andean South America**	Bolivia	La Paz	Murillo	Bolivia	La Paz	Camacho
**Andean South America**	Bolivia	La Paz	Nor Yungas	Bolivia	La Paz	Caranavi
**Andean South America**	Bolivia	La Paz	Omasuyos	Bolivia	La Paz	Franz Tamayo
**Andean South America**	Bolivia	La Paz	Pacajes	Bolivia	La Paz	General Jose Manuel Pando
**Andean South America**	Bolivia	Lake Titicaca	NA	Bolivia	La Paz	Ingavi
**Andean South America**	Bolivia	Lake Titicaca	Lake Titicaca	Bolivia	La Paz	Larecaja
**Andean South America**	Bolivia	Potosi	Charcas	Bolivia	La Paz	Los Andes
**Andean South America**	Bolivia	Potosi	Cornelio Saavedra	Bolivia	La Paz	Manco Kapac
**Andean South America**	Bolivia	Potosi	Jose Maria Linares	Bolivia	La Paz	Munecas
**Andean South America**	Colombia	NA	NA	Bolivia	La Paz	Murillo

**Andean South America**	Colombia	Antioquia	NA	Bolivia	La Paz	Nor Yungas
**Andean South America**	Colombia	Antioquia	Abejorral	Bolivia	La Paz	Omasuyos
**Andean South America**	Colombia	Antioquia	Abriaquí	Bolivia	La Paz	Sud Yungas
**Andean South America**	Colombia	Antioquia	Alejandría	Bolivia	Lake Titicaca	NA
**Andean South America**	Colombia	Antioquia	Amagá	Bolivia	Lake Titicaca	Lake Titicaca
**Andean South America**	Colombia	Antioquia	Amalfi	Bolivia	Potosi	NA
**Andean South America**	Colombia	Antioquia	Andes	Bolivia	Potosi	Charcas
**Andean South America**	Colombia	Antioquia	Angelópolis	Bolivia	Potosi	Chayanta
**Andean South America**	Colombia	Antioquia	Angostura	Bolivia	Potosi	Cornelio Saavedra
**Andean South America**	Colombia	Antioquia	Anzá	Bolivia	Potosi	Jose Maria Linares
**Andean South America**	Colombia	Antioquia	Armenia	Bolivia	Potosi	Tomas Frias
**Andean South America**	Colombia	Antioquia	Bello	Colombia	Antioquia	NA
**Andean South America**	Colombia	Antioquia	Belmira	Colombia	Antioquia	Abejorral
**Andean South America**	Colombia	Antioquia	Betania	Colombia	Antioquia	Andes
**Andean South America**	Colombia	Antioquia	Betulia	Colombia	Antioquia	Angelópolis

**Andean South America**	Colombia	Antioquia	Bolívar	Colombia	Antioquia	Anzá
**Andean South America**	Colombia	Antioquia	Buriticá	Colombia	Antioquia	Betania
**Andean South America**	Colombia	Antioquia	Caicedo	Colombia	Antioquia	Caicedo
**Andean South America**	Colombia	Antioquia	Caldas	Colombia	Antioquia	Concordia
**Andean South America**	Colombia	Antioquia	Caracolí	Colombia	Antioquia	Ebéjico
**Andean South America**	Colombia	Antioquia	Caramanta	Colombia	Antioquia	Envigado
**Andean South America**	Colombia	Antioquia	Carolina del Principe	Colombia	Antioquia	Heliconia
**Andean South America**	Colombia	Antioquia	Caucasia	Colombia	Antioquia	Nechí
**Andean South America**	Colombia	Antioquia	Cañasgordas	Colombia	Antioquia	Salgar
**Andean South America**	Colombia	Antioquia	Cisneros	Colombia	Antioquia	Titiribí
**Andean South America**	Colombia	Antioquia	Cocorná	Colombia	Antioquia	Urrao
**Andean South America**	Colombia	Antioquia	Concepción	Colombia	Antioquia	Valparaíso
**Andean South America**	Colombia	Antioquia	Concordia	Colombia	Antioquia	Venecia
**Andean South America**	Colombia	Antioquia	Cáceres	Colombia	Bolívar	Achí
**Andean South America**	Colombia	Antioquia	Dabeiba	Colombia	Bolívar	Montecristo

**Andean South America**	Colombia	Antioquia	Don Matías	Colombia	Nariño	NA
**Andean South America**	Colombia	Antioquia	Ebéjico	Colombia	Nariño	Aldana
**Andean South America**	Colombia	Antioquia	El Bagre	Colombia	Nariño	Barbacoas
**Andean South America**	Colombia	Antioquia	El Carmen de Viboral	Colombia	Nariño	Contadero
**Andean South America**	Colombia	Antioquia	El Santuario	Colombia	Nariño	Cuaspud
**Andean South America**	Colombia	Antioquia	Entrerríos	Colombia	Nariño	Cumbal
**Andean South America**	Colombia	Antioquia	Envigado	Colombia	Nariño	El Charco
**Andean South America**	Colombia	Antioquia	Frontino	Colombia	Nariño	El Rosario
**Andean South America**	Colombia	Antioquia	Giraldo	Colombia	Nariño	Francisco Pizarro
**Andean South America**	Colombia	Antioquia	Granada	Colombia	Nariño	Guachucal
**Andean South America**	Colombia	Antioquia	Guadalupe	Colombia	Nariño	Guaitarilla
**Andean South America**	Colombia	Antioquia	Guarne	Colombia	Nariño	Gualmatán
**Andean South America**	Colombia	Antioquia	Gómez Plata	Colombia	Nariño	Iles
**Andean South America**	Colombia	Antioquia	Heliconia	Colombia	Nariño	Imués
**Andean South America**	Colombia	Antioquia	Hispania	Colombia	Nariño	Ipiales

**Andean South America**	Colombia	Antioquia	Itagüí	Colombia	Nariño	La Tola
**Andean South America**	Colombia	Antioquia	Jardín	Colombia	Nariño	Leiva
**Andean South America**	Colombia	Antioquia	Jericó	Colombia	Nariño	Magüí
**Andean South America**	Colombia	Antioquia	La Ceja	Colombia	Nariño	Mosquera
**Andean South America**	Colombia	Antioquia	La Estrella	Colombia	Nariño	Olaya Herrera
**Andean South America**	Colombia	Antioquia	La Unión de Sucre	Colombia	Nariño	Ospina
**Andean South America**	Colombia	Antioquia	Liborina	Colombia	Nariño	Potosí
**Andean South America**	Colombia	Antioquia	Marinilla	Colombia	Nariño	Pupiales
**Andean South America**	Colombia	Antioquia	Medellín	Colombia	Nariño	Ricaurte
**Andean South America**	Colombia	Antioquia	Montebello	Colombia	Nariño	Roberto Payán
**Andean South America**	Colombia	Antioquia	Murindó	Colombia	Nariño	San Bernardo
**Andean South America**	Colombia	Antioquia	Mutatá	Colombia	Nariño	San Juan de Pasto
**Andean South America**	Colombia	Antioquia	Nechí	Colombia	Nariño	San Pedro de Cartago
**Andean South America**	Colombia	Antioquia	Pequé	Colombia	Nariño	Santa Bárbara
**Andean South America**	Colombia	Antioquia	Pueblorrico	Colombia	Nariño	Sapuyes

**Andean South America**	Colombia	Antioquia	Retiro	Colombia	Nariño	Tangua
**Andean South America**	Colombia	Antioquia	Rionegro	Colombia	Nariño	Tumaco
**Andean South America**	Colombia	Antioquia	Sabanalarga	Colombia	Nariño	Túquerres
**Andean South America**	Colombia	Antioquia	Sabaneta	Colombia	Nariño	Yacuanquer
**Andean South America**	Colombia	Antioquia	Salgar	Colombia	Risaralda	Guática
**Andean South America**	Colombia	Antioquia	San Carlos	Colombia	Santander	Aratoca
**Andean South America**	Colombia	Antioquia	San Jerónimo	Colombia	Santander	Barichara
**Andean South America**	Colombia	Antioquia	San José de la Montaña	Colombia	Santander	Cabrera
**Andean South America**	Colombia	Antioquia	San Pedro de los Milagros	Colombia	Santander	Cerrito
**Andean South America**	Colombia	Antioquia	San Rafael	Colombia	Santander	Charalá
**Andean South America**	Colombia	Antioquia	San Roque	Colombia	Santander	Chimá
**Andean South America**	Colombia	Antioquia	San Vicente	Colombia	Santander	Confines
**Andean South America**	Colombia	Antioquia	Santa Rosa de Osos	Colombia	Santander	Contratación
**Andean South America**	Colombia	Antioquia	Santo Domingo	Colombia	Santander	Galán
**Andean South America**	Colombia	Antioquia	Sopetrán	Colombia	Santander	Guadalupe

**Andean South America**	Colombia	Antioquia	Tarazá	Colombia	Santander	Guapotá
**Andean South America**	Colombia	Antioquia	Tarso	Colombia	Santander	Hato
**Andean South America**	Colombia	Antioquia	Titiribí	Colombia	Santander	Jordán
**Andean South America**	Colombia	Antioquia	Toledo	Colombia	Santander	Los Santos
**Andean South America**	Colombia	Antioquia	Támesis	Colombia	Santander	Macaravita
**Andean South America**	Colombia	Antioquia	Uramita	Colombia	Santander	Ocamonte
**Andean South America**	Colombia	Antioquia	Urrao	Colombia	Santander	Oiba
**Andean South America**	Colombia	Antioquia	Valparaíso	Colombia	Santander	Palmar
**Andean South America**	Colombia	Antioquia	Venecia	Colombia	Santander	Palmas del Socorro
**Andean South America**	Colombia	Antioquia	Yarumal	Colombia	Santander	Pinchote
**Andean South America**	Colombia	Bolívar	Montecristo	Colombia	Santander	Páramo
**Andean South America**	Colombia	Boyacá	Duitama	Colombia	Santander	Socorro
**Andean South America**	Colombia	Caldas	NA	Colombia	Santander	Villanueva
**Andean South America**	Colombia	Caldas	Aguadas	Colombia	Santander	Zapatoca
**Andean South America**	Colombia	Caldas	Anserma	Colombia	Valle del Cauca	Andalucía

**Andean South America**	Colombia	Caldas	Aranzazú	Colombia	Valle del Cauca	Bolívar
**Andean South America**	Colombia	Caldas	Belalcázar	Ecuador	Azuay	Nabón
**Andean South America**	Colombia	Caldas	Chinchiná	Ecuador	El Oro	Balsas
**Andean South America**	Colombia	Caldas	Filadelfia	Ecuador	El Oro	Chilla
**Andean South America**	Colombia	Caldas	La Merced	Ecuador	El Oro	El Guabo
**Andean South America**	Colombia	Caldas	Manizales	Ecuador	El Oro	Las Lajas
**Andean South America**	Colombia	Caldas	Manzanares	Ecuador	El Oro	Marcabelí
**Andean South America**	Colombia	Caldas	Marquetalia	Ecuador	El Oro	Pasaje
**Andean South America**	Colombia	Caldas	Marulanda	Ecuador	El Oro	Piñas
**Andean South America**	Colombia	Caldas	Neira	Ecuador	El Oro	Portovelo
**Andean South America**	Colombia	Caldas	Palestina	Ecuador	El Oro	Santa Rosa
**Andean South America**	Colombia	Caldas	Pácora	Ecuador	El Oro	Zaruma
**Andean South America**	Colombia	Caldas	Riosucio	Ecuador	Loja	NA
**Andean South America**	Colombia	Caldas	Risaralda	Ecuador	Loja	Calvas
**Andean South America**	Colombia	Caldas	Salamina	Ecuador	Loja	Catamayo

**Andean South America**	Colombia	Caldas	Villamaría	Ecuador	Loja	Chaguarpamba
**Andean South America**	Colombia	Caldas	Viterbo	Ecuador	Loja	Espíndola
**Andean South America**	Colombia	Cauca	Argelia	Ecuador	Loja	Gonzanamá
**Andean South America**	Colombia	Cauca	Balboa	Ecuador	Loja	Olmedo
**Andean South America**	Colombia	Cauca	La Sierra	Ecuador	Loja	Paltas
**Andean South America**	Colombia	Cauca	Rosas	Ecuador	Loja	Quilanga
**Andean South America**	Colombia	Cauca	Sotará	Ecuador	Loja	Saraguro
**Andean South America**	Colombia	Cundinamarca	Chipaque	Ecuador	Loja	Sozoranga
**Andean South America**	Colombia	Cundinamarca	Choachí	Ecuador	Zamora Chinchipe	NA
**Andean South America**	Colombia	Cundinamarca	Manta	Ecuador	Zamora Chinchipe	Centinela del Cóndor
**Andean South America**	Colombia	Cundinamarca	Ubaque	Ecuador	Zamora Chinchipe	Chinchipe
**Andean South America**	Colombia	Nariño	NA	Ecuador	Zamora Chinchipe	Yacuambi
**Andean South America**	Colombia	Nariño	Albán	Ecuador	Zamora Chinchipe	Yantzaza
**Andean South America**	Colombia	Nariño	Aldana	Peru	Amazonas	NA
**Andean South America**	Colombia	Nariño	Ancuyá	Peru	Amazonas	Bongará

**Andean South America**	Colombia	Nariño	Arboleda	Peru	Amazonas	Chachapoyas
**Andean South America**	Colombia	Nariño	Barbacoas	Peru	Amazonas	Luya
**Andean South America**	Colombia	Nariño	Belén	Peru	Amazonas	Rodríguez de Mendoza
**Andean South America**	Colombia	Nariño	Buesaco	Peru	Amazonas	Utcubamba
**Andean South America**	Colombia	Nariño	Consacá	Peru	Ancash	NA
**Andean South America**	Colombia	Nariño	Contadero	Peru	Ancash	Aija
**Andean South America**	Colombia	Nariño	Cuaspud	Peru	Ancash	Antonio Raymondi
**Andean South America**	Colombia	Nariño	Cumbal	Peru	Ancash	Asunción
**Andean South America**	Colombia	Nariño	Cumbitara	Peru	Ancash	Bolognesi
**Andean South America**	Colombia	Nariño	Córdoba	Peru	Ancash	Carhuaz
**Andean South America**	Colombia	Nariño	El Charco	Peru	Ancash	Carlos Fermin Fitzcarrald
**Andean South America**	Colombia	Nariño	El Rosario	Peru	Ancash	Corongo
**Andean South America**	Colombia	Nariño	El Tablón de Gomez	Peru	Ancash	Huaraz
**Andean South America**	Colombia	Nariño	El Tambo	Peru	Ancash	Huari
**Andean South America**	Colombia	Nariño	Francisco Pizarro	Peru	Ancash	Huaylas

**Andean South America**	Colombia	Nariño	Funes	Peru	Ancash	Mariscal Luzuriaga
**Andean South America**	Colombia	Nariño	Guachucal	Peru	Ancash	Ocros
**Andean South America**	Colombia	Nariño	Guaitarilla	Peru	Ancash	Pallasca
**Andean South America**	Colombia	Nariño	Gualmatán	Peru	Ancash	Pomabamba
**Andean South America**	Colombia	Nariño	Iles	Peru	Ancash	Recuay
**Andean South America**	Colombia	Nariño	Imués	Peru	Ancash	Sihuas
**Andean South America**	Colombia	Nariño	Ipiales	Peru	Ancash	Yungay
**Andean South America**	Colombia	Nariño	La Cruz	Peru	Apurímac	NA
**Andean South America**	Colombia	Nariño	La Florida	Peru	Apurímac	Abancay
**Andean South America**	Colombia	Nariño	La Llanada	Peru	Apurímac	Andahuaylas
**Andean South America**	Colombia	Nariño	La Tola	Peru	Apurímac	Antabamba
**Andean South America**	Colombia	Nariño	Leiva	Peru	Apurímac	Aymaraes
**Andean South America**	Colombia	Nariño	Los Andes	Peru	Apurímac	Chincheros
**Andean South America**	Colombia	Nariño	Magüí	Peru	Apurímac	Cotabambas
**Andean South America**	Colombia	Nariño	Mallama	Peru	Apurímac	Grau

**Andean South America**	Colombia	Nariño	Mosquera	Peru	Arequipa	NA
**Andean South America**	Colombia	Nariño	Olaya Herrera	Peru	Arequipa	Arequipa
**Andean South America**	Colombia	Nariño	Ospina	Peru	Arequipa	Camaná
**Andean South America**	Colombia	Nariño	Policarpa	Peru	Arequipa	Caravelí
**Andean South America**	Colombia	Nariño	Potosí	Peru	Arequipa	Castilla
**Andean South America**	Colombia	Nariño	Providencia	Peru	Arequipa	Caylloma
**Andean South America**	Colombia	Nariño	Puerres	Peru	Arequipa	Condesuyos
**Andean South America**	Colombia	Nariño	Pupiales	Peru	Arequipa	Islay
**Andean South America**	Colombia	Nariño	Ricaurte	Peru	Arequipa	La Unión
**Andean South America**	Colombia	Nariño	Roberto Payán	Peru	Ayacucho	NA
**Andean South America**	Colombia	Nariño	San Bernardo	Peru	Ayacucho	Cangallo
**Andean South America**	Colombia	Nariño	San Juan de Pasto	Peru	Ayacucho	Huamanga
**Andean South America**	Colombia	Nariño	San Lorenzo	Peru	Ayacucho	Huanca Sancos
**Andean South America**	Colombia	Nariño	San Pablo	Peru	Ayacucho	Huanta
**Andean South America**	Colombia	Nariño	San Pedro de Cartago	Peru	Ayacucho	La Mar

**Andean South America**	Colombia	Nariño	Santa Bárbara	Peru	Ayacucho	Lucanas
**Andean South America**	Colombia	Nariño	Santa Cruz	Peru	Ayacucho	Parinacochas
**Andean South America**	Colombia	Nariño	Sapuyes	Peru	Ayacucho	Paucar del Sara Sara
**Andean South America**	Colombia	Nariño	Tangua	Peru	Ayacucho	Sucre
**Andean South America**	Colombia	Nariño	Tumaco	Peru	Ayacucho	Victor Fajardo
**Andean South America**	Colombia	Nariño	Túquerres	Peru	Ayacucho	Vilcas Huamán
**Andean South America**	Colombia	Nariño	Yacuanquer	Peru	Cajamarca	NA
**Andean South America**	Colombia	Norte de Santander	Santo Domingo de Silos	Peru	Cajamarca	Cajabamba
**Andean South America**	Colombia	Putumayo	Colón	Peru	Cajamarca	Cajamarca
**Andean South America**	Colombia	Quindío	NA	Peru	Cajamarca	Celendín
**Andean South America**	Colombia	Quindío	Armenia	Peru	Cajamarca	Chota
**Andean South America**	Colombia	Quindío	Calarcá	Peru	Cajamarca	Contumazá
**Andean South America**	Colombia	Quindío	Circasia	Peru	Cajamarca	Cutervo
**Andean South America**	Colombia	Quindío	Córdoba	Peru	Cajamarca	Hualgayoc
**Andean South America**	Colombia	Quindío	Salento	Peru	Cajamarca	Jaén

**Andean South America**	Colombia	Risaralda	NA	Peru	Cajamarca	San Ignacio
**Andean South America**	Colombia	Risaralda	Apía	Peru	Cajamarca	San Marcos
**Andean South America**	Colombia	Risaralda	Belén de Umbría	Peru	Cajamarca	San Miguel
**Andean South America**	Colombia	Risaralda	Dosquebradas	Peru	Cajamarca	San Pablo
**Andean South America**	Colombia	Risaralda	Guática	Peru	Cajamarca	Santa Cruz
**Andean South America**	Colombia	Risaralda	La Celia	Peru	Cusco	NA
**Andean South America**	Colombia	Risaralda	La Virginia	Peru	Cusco	Acomayo
**Andean South America**	Colombia	Risaralda	Marsella	Peru	Cusco	Anta
**Andean South America**	Colombia	Risaralda	Mistrato	Peru	Cusco	Calca
**Andean South America**	Colombia	Risaralda	Pereira	Peru	Cusco	Canas
**Andean South America**	Colombia	Risaralda	Pueblo Rico	Peru	Cusco	Canchis
**Andean South America**	Colombia	Risaralda	Quinchía	Peru	Cusco	Chumbivilcas
**Andean South America**	Colombia	Risaralda	Santa Rosa de Cabal	Peru	Cusco	Cusco
**Andean South America**	Colombia	Risaralda	Santuario	Peru	Cusco	Espinar
**Andean South America**	Colombia	Santander	Aguada	Peru	Cusco	La Convención

**Andean South America**	Colombia	Santander	Aratoca	Peru	Cusco	Paruro
**Andean South America**	Colombia	Santander	Barbosa	Peru	Cusco	Paucartambo
**Andean South America**	Colombia	Santander	Barichara	Peru	Cusco	Quispicanchi
**Andean South America**	Colombia	Santander	Cabrera	Peru	Cusco	Urubamba
**Andean South America**	Colombia	Santander	California	Peru	Huancavelica	NA
**Andean South America**	Colombia	Santander	Carcasí	Peru	Huancavelica	Acobamba
**Andean South America**	Colombia	Santander	Cepitá	Peru	Huancavelica	Angaraes
**Andean South America**	Colombia	Santander	Cerrito	Peru	Huancavelica	Castrovirreyna
**Andean South America**	Colombia	Santander	Charalá	Peru	Huancavelica	Churcampa
**Andean South America**	Colombia	Santander	Chimá	Peru	Huancavelica	Huancavelica
**Andean South America**	Colombia	Santander	Chipatá	Peru	Huancavelica	Huaytara
**Andean South America**	Colombia	Santander	Concepción	Peru	Huancavelica	Tayacaja
**Andean South America**	Colombia	Santander	Confines	Peru	Huánuco	NA
**Andean South America**	Colombia	Santander	Contratación	Peru	Huánuco	Ambo
**Andean South America**	Colombia	Santander	Coromoro	Peru	Huánuco	Dos de Mayo

**Andean South America**	Colombia	Santander	Curití	Peru	Huánuco	Huacaybamba
**Andean South America**	Colombia	Santander	El Guacamayo	Peru	Huánuco	Huamalíes
**Andean South America**	Colombia	Santander	El Peñon	Peru	Huánuco	Huenuco
**Andean South America**	Colombia	Santander	Galán	Peru	Huánuco	Lauricocha
**Andean South America**	Colombia	Santander	Guaca	Peru	Huánuco	Marañón
**Andean South America**	Colombia	Santander	Guadalupe	Peru	Huánuco	Pachitea
**Andean South America**	Colombia	Santander	Guapotá	Peru	Huánuco	Yarowilca
**Andean South America**	Colombia	Santander	Güepsa	Peru	Ica	Ica
**Andean South America**	Colombia	Santander	Hato	Peru	Junín	NA
**Andean South America**	Colombia	Santander	Jordán	Peru	Junín	Chupaca
**Andean South America**	Colombia	Santander	Los Santos	Peru	Junín	Concepción
**Andean South America**	Colombia	Santander	Macaravita	Peru	Junín	Huancayo
**Andean South America**	Colombia	Santander	Mogotes	Peru	Junín	Jauja
**Andean South America**	Colombia	Santander	Molagavita	Peru	Junín	Junín
**Andean South America**	Colombia	Santander	Ocamonte	Peru	Junín	Satipo

**Andean South America**	Colombia	Santander	Oiba	Peru	Junín	Tarma
**Andean South America**	Colombia	Santander	Onzaga	Peru	Junín	Yauli
**Andean South America**	Colombia	Santander	Palmar	Peru	La Libertad	NA
**Andean South America**	Colombia	Santander	Palmas del Socorro	Peru	La Libertad	Bolívar
**Andean South America**	Colombia	Santander	Pinchote	Peru	La Libertad	Gran Chimú
**Andean South America**	Colombia	Santander	Páramo	Peru	La Libertad	Julcan
**Andean South America**	Colombia	Santander	San Benito	Peru	La Libertad	Otuzco
**Andean South America**	Colombia	Santander	San Joaquín	Peru	La Libertad	Pataz
**Andean South America**	Colombia	Santander	San José de Miranda	Peru	La Libertad	Santiago de Chuco
**Andean South America**	Colombia	Santander	San Miguel	Peru	La Libertad	Sánchez Carrión
**Andean South America**	Colombia	Santander	Socorro	Peru	Lima	Cajatambo
**Andean South America**	Colombia	Santander	Suaita	Peru	Lima	Canta
**Andean South America**	Colombia	Santander	Sucre	Peru	Lima	Huarochiri
**Andean South America**	Colombia	Santander	Valle de San José	Peru	Lima	Oyon
**Andean South America**	Colombia	Santander	Vetas	Peru	Lima	Yauyos

**Andean South America**	Colombia	Santander	Villanueva	Peru	Madre de Dios	NA
**Andean South America**	Colombia	Santander	Zapatoca	Peru	Madre de Dios	Manu
**Andean South America**	Colombia	Valle del Cauca	Andalucía	Peru	Madre de Dios	Tahuamanu
**Andean South America**	Colombia	Valle del Cauca	Bolívar	Peru	Madre de Dios	Tambopata
**Andean South America**	Colombia	Valle del Cauca	Bugalagrande	Peru	Moquegua	NA
**Andean South America**	Colombia	Valle del Cauca	El Águila	Peru	Moquegua	General Sánchez Cerro
**Andean South America**	Colombia	Valle del Cauca	Guacarí	Peru	Moquegua	Mariscal Nieto
**Andean South America**	Colombia	Valle del Cauca	Guadalajara de Buga	Peru	Pasco	NA
**Andean South America**	Colombia	Valle del Cauca	La Cumbre	Peru	Pasco	Daniel Alcides Carrión
**Andean South America**	Colombia	Valle del Cauca	Pradera	Peru	Pasco	Pasco
**Andean South America**	Colombia	Valle del Cauca	Riofrío	Peru	Piura	Ayabaca
**Andean South America**	Colombia	Valle del Cauca	Roldanillo	Peru	Piura	Huancabamba
**Andean South America**	Colombia	Valle del Cauca	San Pedro	Peru	Puno	NA
**Andean South America**	Colombia	Valle del Cauca	Trujillo	Peru	Puno	Azángaro
**Andean South America**	Colombia	Valle del Cauca	Tuluá	Peru	Puno	Carabaya

**Andean South America**	Colombia	Valle del Cauca	Yotoco	Peru	Puno	Chucuíto
**Andean South America**	Colombia	Valle del Cauca	Zarzal	Peru	Puno	El Collao
**Andean South America**	Ecuador	Azuay	NA	Peru	Puno	Huancane
**Andean South America**	Ecuador	Azuay	Chordeleg	Peru	Puno	Lago Titicaca
**Andean South America**	Ecuador	Azuay	Cuenca	Peru	Puno	Lampa
**Andean South America**	Ecuador	Azuay	El Pan	Peru	Puno	Melgar
**Andean South America**	Ecuador	Azuay	Girón	Peru	Puno	Moho
**Andean South America**	Ecuador	Azuay	Guachapala	Peru	Puno	Puno
**Andean South America**	Ecuador	Azuay	Gualaceo	Peru	Puno	San Antonio de Putina
**Andean South America**	Ecuador	Azuay	Nabón	Peru	Puno	San Román
**Andean South America**	Ecuador	Azuay	Oña	Peru	Puno	Sandia
**Andean South America**	Ecuador	Azuay	Paute	Peru	Puno	Yunguyo
**Andean South America**	Ecuador	Azuay	Pucará	Peru	Tacna	NA
**Andean South America**	Ecuador	Azuay	San Fernando	Peru	Tacna	Candarave
**Andean South America**	Ecuador	Azuay	Santa Isabel	Peru	Tacna	Jorge Basadre

**Andean South America**	Ecuador	Azuay	Sevilla de Oro	Peru	Tacna	Tacna
**Andean South America**	Ecuador	Azuay	Sigsig	Peru	Tacna	Tarata
**Andean South America**	Ecuador	Bolivar	NA	Venezuela	Carabobo	Juan José Mora
**Andean South America**	Ecuador	Bolivar	Chillanes	NA	NA	NA
**Andean South America**	Ecuador	Bolivar	Chimbo	NA	NA	NA
**Andean South America**	Ecuador	Bolivar	Guaranda	NA	NA	NA
**Andean South America**	Ecuador	Bolivar	San Miguel	NA	NA	NA
**Andean South America**	Ecuador	Carchi	NA	NA	NA	NA
**Andean South America**	Ecuador	Carchi	Espejo	NA	NA	NA
**Andean South America**	Ecuador	Carchi	Montúfar	NA	NA	NA
**Andean South America**	Ecuador	Carchi	San Pedro de Huaca	NA	NA	NA
**Andean South America**	Ecuador	Carchi	Tulcán	NA	NA	NA
**Andean South America**	Ecuador	Cañar	NA	NA	NA	NA
**Andean South America**	Ecuador	Cañar	Azogues	NA	NA	NA
**Andean South America**	Ecuador	Cañar	Biblián	NA	NA	NA

**Andean South America**	Ecuador	Cañar	Cañar	NA	NA	NA
**Andean South America**	Ecuador	Cañar	Déleg	NA	NA	NA
**Andean South America**	Ecuador	Cañar	El Tambo	NA	NA	NA
**Andean South America**	Ecuador	Cañar	Suscal	NA	NA	NA
**Andean South America**	Ecuador	Chimborazo	NA	NA	NA	NA
**Andean South America**	Ecuador	Chimborazo	Alausí	NA	NA	NA
**Andean South America**	Ecuador	Chimborazo	Chambo	NA	NA	NA
**Andean South America**	Ecuador	Chimborazo	Chunchi	NA	NA	NA
**Andean South America**	Ecuador	Chimborazo	Colta	NA	NA	NA
**Andean South America**	Ecuador	Chimborazo	Guamote	NA	NA	NA
**Andean South America**	Ecuador	Chimborazo	Guano	NA	NA	NA
**Andean South America**	Ecuador	Chimborazo	Pallatanga	NA	NA	NA
**Andean South America**	Ecuador	Chimborazo	Penipe	NA	NA	NA
**Andean South America**	Ecuador	Chimborazo	Riobamba	NA	NA	NA
**Andean South America**	Ecuador	Cotopaxi	NA	NA	NA	NA

**Andean South America**	Ecuador	Cotopaxi	Latacunga	NA	NA	NA
**Andean South America**	Ecuador	Cotopaxi	Pujilí	NA	NA	NA
**Andean South America**	Ecuador	Cotopaxi	Salcedo	NA	NA	NA
**Andean South America**	Ecuador	Cotopaxi	Saquisili	NA	NA	NA
**Andean South America**	Ecuador	Cotopaxi	Saquisilí	NA	NA	NA
**Andean South America**	Ecuador	Cotopaxi	Sigchos	NA	NA	NA
**Andean South America**	Ecuador	El Oro	Atahualpa	NA	NA	NA
**Andean South America**	Ecuador	El Oro	Balsas	NA	NA	NA
**Andean South America**	Ecuador	El Oro	Chilla	NA	NA	NA
**Andean South America**	Ecuador	El Oro	Marcabelí	NA	NA	NA
**Andean South America**	Ecuador	El Oro	Pasaje	NA	NA	NA
**Andean South America**	Ecuador	El Oro	Piñas	NA	NA	NA
**Andean South America**	Ecuador	El Oro	Portovelo	NA	NA	NA
**Andean South America**	Ecuador	El Oro	Zaruma	NA	NA	NA
**Andean South America**	Ecuador	Imbabura	NA	NA	NA	NA

**Andean South America**	Ecuador	Imbabura	Antonio Ante	NA	NA	NA
**Andean South America**	Ecuador	Imbabura	Ibarra	NA	NA	NA
**Andean South America**	Ecuador	Imbabura	Otavalo	NA	NA	NA
**Andean South America**	Ecuador	Loja	NA	NA	NA	NA
**Andean South America**	Ecuador	Loja	Calvas	NA	NA	NA
**Andean South America**	Ecuador	Loja	Catamayo	NA	NA	NA
**Andean South America**	Ecuador	Loja	Chaguarpamba	NA	NA	NA
**Andean South America**	Ecuador	Loja	Espíndola	NA	NA	NA
**Andean South America**	Ecuador	Loja	Gonzanamá	NA	NA	NA
**Andean South America**	Ecuador	Loja	Loja	NA	NA	NA
**Andean South America**	Ecuador	Loja	Olmedo	NA	NA	NA
**Andean South America**	Ecuador	Loja	Paltas	NA	NA	NA
**Andean South America**	Ecuador	Loja	Quilanga	NA	NA	NA
**Andean South America**	Ecuador	Loja	Saraguro	NA	NA	NA
**Andean South America**	Ecuador	Loja	Sozoranga	NA	NA	NA

**Andean South America**	Ecuador	Pichincha	NA	NA	NA	NA
**Andean South America**	Ecuador	Pichincha	Mejía	NA	NA	NA
**Andean South America**	Ecuador	Pichincha	Quito	NA	NA	NA
**Andean South America**	Ecuador	Pichincha	Rumiñahui	NA	NA	NA
**Andean South America**	Ecuador	Tungurahua	NA	NA	NA	NA
**Andean South America**	Ecuador	Tungurahua	Ambato	NA	NA	NA
**Andean South America**	Ecuador	Tungurahua	Baños de Agua Santa	NA	NA	NA
**Andean South America**	Ecuador	Tungurahua	Cevallos	NA	NA	NA
**Andean South America**	Ecuador	Tungurahua	Mocha	NA	NA	NA
**Andean South America**	Ecuador	Tungurahua	Patate	NA	NA	NA
**Andean South America**	Ecuador	Tungurahua	Quero	NA	NA	NA
**Andean South America**	Ecuador	Tungurahua	San Pedro de Pelileo	NA	NA	NA
**Andean South America**	Ecuador	Tungurahua	Santiago de Pillaro	NA	NA	NA
**Andean South America**	Ecuador	Tungurahua	Tisaleo	NA	NA	NA
**Andean South America**	Ecuador	Zamora Chinchipe	NA	NA	NA	NA

**Andean South America**	Ecuador	Zamora Chinchipe	Centinela del Cóndor	NA	NA	NA
**Andean South America**	Ecuador	Zamora Chinchipe	Chinchipe	NA	NA	NA
**Andean South America**	Ecuador	Zamora Chinchipe	Yacuambi	NA	NA	NA
**Andean South America**	Ecuador	Zamora Chinchipe	Yantzaza	NA	NA	NA
**Andean South America**	Ecuador	Zamora Chinchipe	Zamora	NA	NA	NA
**Andean South America**	Peru	Amazonas	NA	NA	NA	NA
**Andean South America**	Peru	Amazonas	Bongará	NA	NA	NA
**Andean South America**	Peru	Amazonas	Chachapoyas	NA	NA	NA
**Andean South America**	Peru	Amazonas	Luya	NA	NA	NA
**Andean South America**	Peru	Amazonas	Rodríguez de Mendoza	NA	NA	NA
**Andean South America**	Peru	Amazonas	Utcubamba	NA	NA	NA
**Andean South America**	Peru	Ancash	NA	NA	NA	NA
**Andean South America**	Peru	Ancash	Aija	NA	NA	NA
**Andean South America**	Peru	Ancash	Antonio Raymondi	NA	NA	NA
**Andean South America**	Peru	Ancash	Asunción	NA	NA	NA

**Andean South America**	Peru	Ancash	Bolognesi	NA	NA	NA
**Andean South America**	Peru	Ancash	Carhuaz	NA	NA	NA
**Andean South America**	Peru	Ancash	Carlos Fermin Fitzcarrald	NA	NA	NA
**Andean South America**	Peru	Ancash	Corongo	NA	NA	NA
**Andean South America**	Peru	Ancash	Huaraz	NA	NA	NA
**Andean South America**	Peru	Ancash	Huari	NA	NA	NA
**Andean South America**	Peru	Ancash	Huaylas	NA	NA	NA
**Andean South America**	Peru	Ancash	Mariscal Luzuriaga	NA	NA	NA
**Andean South America**	Peru	Ancash	Ocros	NA	NA	NA
**Andean South America**	Peru	Ancash	Pallasca	NA	NA	NA
**Andean South America**	Peru	Ancash	Pomabamba	NA	NA	NA
**Andean South America**	Peru	Ancash	Recuay	NA	NA	NA
**Andean South America**	Peru	Ancash	Sihuas	NA	NA	NA
**Andean South America**	Peru	Ancash	Yungay	NA	NA	NA
**Andean South America**	Peru	Apurímac	NA	NA	NA	NA

**Andean South America**	Peru	Apurímac	Abancay	NA	NA	NA
**Andean South America**	Peru	Apurímac	Andahuaylas	NA	NA	NA
**Andean South America**	Peru	Apurímac	Antabamba	NA	NA	NA
**Andean South America**	Peru	Apurímac	Aymaraes	NA	NA	NA
**Andean South America**	Peru	Apurímac	Chincheros	NA	NA	NA
**Andean South America**	Peru	Apurímac	Cotabambas	NA	NA	NA
**Andean South America**	Peru	Apurímac	Grau	NA	NA	NA
**Andean South America**	Peru	Arequipa	NA	NA	NA	NA
**Andean South America**	Peru	Arequipa	Arequipa	NA	NA	NA
**Andean South America**	Peru	Arequipa	Camaná	NA	NA	NA
**Andean South America**	Peru	Arequipa	Caravelí	NA	NA	NA
**Andean South America**	Peru	Arequipa	Castilla	NA	NA	NA
**Andean South America**	Peru	Arequipa	Caylloma	NA	NA	NA
**Andean South America**	Peru	Arequipa	Condesuyos	NA	NA	NA
**Andean South America**	Peru	Arequipa	Islay	NA	NA	NA

**Andean South America**	Peru	Arequipa	La Unión	NA	NA	NA
**Andean South America**	Peru	Ayacucho	NA	NA	NA	NA
**Andean South America**	Peru	Ayacucho	Cangallo	NA	NA	NA
**Andean South America**	Peru	Ayacucho	Huamanga	NA	NA	NA
**Andean South America**	Peru	Ayacucho	Huanca Sancos	NA	NA	NA
**Andean South America**	Peru	Ayacucho	Huanta	NA	NA	NA
**Andean South America**	Peru	Ayacucho	La Mar	NA	NA	NA
**Andean South America**	Peru	Ayacucho	Lucanas	NA	NA	NA
**Andean South America**	Peru	Ayacucho	Parinacochas	NA	NA	NA
**Andean South America**	Peru	Ayacucho	Paucar del Sara Sara	NA	NA	NA
**Andean South America**	Peru	Ayacucho	Sucre	NA	NA	NA
**Andean South America**	Peru	Ayacucho	Victor Fajardo	NA	NA	NA
**Andean South America**	Peru	Ayacucho	Vilcas Huamán	NA	NA	NA
**Andean South America**	Peru	Cajamarca	NA	NA	NA	NA
**Andean South America**	Peru	Cajamarca	Cajabamba	NA	NA	NA

**Andean South America**	Peru	Cajamarca	Cajamarca	NA	NA	NA
**Andean South America**	Peru	Cajamarca	Celendín	NA	NA	NA
**Andean South America**	Peru	Cajamarca	Chota	NA	NA	NA
**Andean South America**	Peru	Cajamarca	Contumazá	NA	NA	NA
**Andean South America**	Peru	Cajamarca	Cutervo	NA	NA	NA
**Andean South America**	Peru	Cajamarca	Hualgayoc	NA	NA	NA
**Andean South America**	Peru	Cajamarca	Jaén	NA	NA	NA
**Andean South America**	Peru	Cajamarca	San Ignacio	NA	NA	NA
**Andean South America**	Peru	Cajamarca	San Marcos	NA	NA	NA
**Andean South America**	Peru	Cajamarca	San Miguel	NA	NA	NA
**Andean South America**	Peru	Cajamarca	San Pablo	NA	NA	NA
**Andean South America**	Peru	Cajamarca	Santa Cruz	NA	NA	NA
**Andean South America**	Peru	Cusco	NA	NA	NA	NA
**Andean South America**	Peru	Cusco	Acomayo	NA	NA	NA
**Andean South America**	Peru	Cusco	Anta	NA	NA	NA

**Andean South America**	Peru	Cusco	Calca	NA	NA	NA
**Andean South America**	Peru	Cusco	Canas	NA	NA	NA
**Andean South America**	Peru	Cusco	Canchis	NA	NA	NA
**Andean South America**	Peru	Cusco	Chumbivilcas	NA	NA	NA
**Andean South America**	Peru	Cusco	Cusco	NA	NA	NA
**Andean South America**	Peru	Cusco	Espinar	NA	NA	NA
**Andean South America**	Peru	Cusco	La Convención	NA	NA	NA
**Andean South America**	Peru	Cusco	Paruro	NA	NA	NA
**Andean South America**	Peru	Cusco	Paucartambo	NA	NA	NA
**Andean South America**	Peru	Cusco	Quispicanchi	NA	NA	NA
**Andean South America**	Peru	Cusco	Urubamba	NA	NA	NA
**Andean South America**	Peru	Huancavelica	NA	NA	NA	NA
**Andean South America**	Peru	Huancavelica	Acobamba	NA	NA	NA
**Andean South America**	Peru	Huancavelica	Angaraes	NA	NA	NA
**Andean South America**	Peru	Huancavelica	Castrovirreyna	NA	NA	NA

**Andean South America**	Peru	Huancavelica	Churcampa	NA	NA	NA
**Andean South America**	Peru	Huancavelica	Huancavelica	NA	NA	NA
**Andean South America**	Peru	Huancavelica	Huaytara	NA	NA	NA
**Andean South America**	Peru	Huancavelica	Tayacaja	NA	NA	NA
**Andean South America**	Peru	Huánuco	NA	NA	NA	NA
**Andean South America**	Peru	Huánuco	Ambo	NA	NA	NA
**Andean South America**	Peru	Huánuco	Dos de Mayo	NA	NA	NA
**Andean South America**	Peru	Huánuco	Huacaybamba	NA	NA	NA
**Andean South America**	Peru	Huánuco	Huamalíes	NA	NA	NA
**Andean South America**	Peru	Huánuco	Huenuco	NA	NA	NA
**Andean South America**	Peru	Huánuco	Lauricocha	NA	NA	NA
**Andean South America**	Peru	Huánuco	Marañón	NA	NA	NA
**Andean South America**	Peru	Huánuco	Pachitea	NA	NA	NA
**Andean South America**	Peru	Huánuco	Yarowilca	NA	NA	NA
**Andean South America**	Peru	Ica	Ica	NA	NA	NA

**Andean South America**	Peru	Ica	Nazca	NA	NA	NA
**Andean South America**	Peru	Junín	NA	NA	NA	NA
**Andean South America**	Peru	Junín	Chupaca	NA	NA	NA
**Andean South America**	Peru	Junín	Concepción	NA	NA	NA
**Andean South America**	Peru	Junín	Huancayo	NA	NA	NA
**Andean South America**	Peru	Junín	Jauja	NA	NA	NA
**Andean South America**	Peru	Junín	Junín	NA	NA	NA
**Andean South America**	Peru	Junín	Satipo	NA	NA	NA
**Andean South America**	Peru	Junín	Tarma	NA	NA	NA
**Andean South America**	Peru	Junín	Yauli	NA	NA	NA
**Andean South America**	Peru	La Libertad	Bolívar	NA	NA	NA
**Andean South America**	Peru	La Libertad	Gran Chimú	NA	NA	NA
**Andean South America**	Peru	La Libertad	Julcan	NA	NA	NA
**Andean South America**	Peru	La Libertad	Otuzco	NA	NA	NA
**Andean South America**	Peru	La Libertad	Pataz	NA	NA	NA

**Andean South America**	Peru	La Libertad	Santiago de Chuco	NA	NA	NA
**Andean South America**	Peru	La Libertad	Sánchez Carrión	NA	NA	NA
**Andean South America**	Peru	Lima	Cajatambo	NA	NA	NA
**Andean South America**	Peru	Lima	Canta	NA	NA	NA
**Andean South America**	Peru	Lima	Huarochiri	NA	NA	NA
**Andean South America**	Peru	Lima	Oyon	NA	NA	NA
**Andean South America**	Peru	Lima	Yauyos	NA	NA	NA
**Andean South America**	Peru	Madre de Dios	NA	NA	NA	NA
**Andean South America**	Peru	Madre de Dios	Manu	NA	NA	NA
**Andean South America**	Peru	Madre de Dios	Tahuamanu	NA	NA	NA
**Andean South America**	Peru	Madre de Dios	Tambopata	NA	NA	NA
**Andean South America**	Peru	Moquegua	NA	NA	NA	NA
**Andean South America**	Peru	Moquegua	General Sánchez Cerro	NA	NA	NA
**Andean South America**	Peru	Moquegua	Mariscal Nieto	NA	NA	NA
**Andean South America**	Peru	Pasco	NA	NA	NA	NA

**Andean South America**	Peru	Pasco	Daniel Alcides Carrión	NA	NA	NA
**Andean South America**	Peru	Pasco	Pasco	NA	NA	NA
**Andean South America**	Peru	Piura	Ayabaca	NA	NA	NA
**Andean South America**	Peru	Piura	Huancabamba	NA	NA	NA
**Andean South America**	Peru	Puno	NA	NA	NA	NA
**Andean South America**	Peru	Puno	Azángaro	NA	NA	NA
**Andean South America**	Peru	Puno	Carabaya	NA	NA	NA
**Andean South America**	Peru	Puno	Chucuíto	NA	NA	NA
**Andean South America**	Peru	Puno	El Collao	NA	NA	NA
**Andean South America**	Peru	Puno	Huancane	NA	NA	NA
**Andean South America**	Peru	Puno	Lago Titicaca	NA	NA	NA
**Andean South America**	Peru	Puno	Lampa	NA	NA	NA
**Andean South America**	Peru	Puno	Melgar	NA	NA	NA
**Andean South America**	Peru	Puno	Moho	NA	NA	NA
**Andean South America**	Peru	Puno	Puno	NA	NA	NA

**Andean South America**	Peru	Puno	San Antonio de Putina	NA	NA	NA
**Andean South America**	Peru	Puno	San Román	NA	NA	NA
**Andean South America**	Peru	Puno	Sandia	NA	NA	NA
**Andean South America**	Peru	Puno	Yunguyo	NA	NA	NA
**Andean South America**	Peru	Tacna	NA	NA	NA	NA
**Andean South America**	Peru	Tacna	Candarave	NA	NA	NA
**Andean South America**	Peru	Tacna	Jorge Basadre	NA	NA	NA
**Andean South America**	Peru	Tacna	Tacna	NA	NA	NA
**Andean South America**	Peru	Tacna	Tarata	NA	NA	NA
**Andean South America**	Venezuela	Carabobo	Juan José Mora	NA	NA	NA
**Andean South America**	Venezuela	Carabobo	Libertador	NA	NA	NA
**Andean South America**	Venezuela	Cojedes	NA	NA	NA	NA
**Andean South America**	Venezuela	Cojedes	Ezequiel Zomora	NA	NA	NA
**Andean South America**	Venezuela	Cojedes	Falcón	NA	NA	NA
**Andean South America**	Venezuela	Cojedes	Romulo Gallegos	NA	NA	NA

**Andean South America**	Venezuela	Cojedes	Tinaco	NA	NA	NA
**Andean South America**	Venezuela	Falcón	Palma Sola	NA	NA	NA
**Andean South America**	Venezuela	Lara	Crespo	NA	NA	NA
**Andean South America**	Venezuela	Yaracuy	NA	NA	NA	NA
**Andean South America**	Venezuela	Yaracuy	Bruzual	NA	NA	NA
**Andean South America**	Venezuela	Yaracuy	Independencia	NA	NA	NA
**Andean South America**	Venezuela	Yaracuy	Manuel Monge	NA	NA	NA
**Andean South America**	Venezuela	Yaracuy	Nirgua	NA	NA	NA
**Andean South America**	Venezuela	Yaracuy	Peña	NA	NA	NA
**Andean South America**	Venezuela	Yaracuy	San Felipe	NA	NA	NA
**Andean South America**	Venezuela	Yaracuy	Veroes	NA	NA	NA
**Central America and Caribbean**	El Salvador	NA	NA	El Salvador	Ahuachapán	NA
**Central America and Caribbean**	El Salvador	Ahuachapán	NA	El Salvador	Ahuachapán	Ahuachapán
**Central America and Caribbean**	El Salvador	Ahuachapán	Ahuachapán	El Salvador	Ahuachapán	Apaneca
**Central America and Caribbean**	El Salvador	Ahuachapán	Apaneca	El Salvador	Ahuachapán	Atiquizaya

**Central America and Caribbean**	El Salvador	Ahuachapán	Atiquizaya	El Salvador	Ahuachapán	Concepción de Ataco
**Central America and Caribbean**	El Salvador	Ahuachapán	Concepción de Ataco	El Salvador	Ahuachapán	El Refugio
**Central America and Caribbean**	El Salvador	Ahuachapán	El Refugio	El Salvador	Ahuachapán	Guaymango
**Central America and Caribbean**	El Salvador	Ahuachapán	Guaymango	El Salvador	Ahuachapán	Jujutla
**Central America and Caribbean**	El Salvador	Ahuachapán	Jujutla	El Salvador	Ahuachapán	San Francisco Menéndez
**Central America and Caribbean**	El Salvador	Ahuachapán	San Francisco Menéndez	El Salvador	Ahuachapán	San Lorenzo
**Central America and Caribbean**	El Salvador	Ahuachapán	San Lorenzo	El Salvador	Ahuachapán	San Pedro Puxtla
**Central America and Caribbean**	El Salvador	Ahuachapán	San Pedro Puxtla	El Salvador	Ahuachapán	Tacuba
**Central America and Caribbean**	El Salvador	Ahuachapán	Tacuba	El Salvador	Ahuachapán	Turín
**Central America and Caribbean**	El Salvador	Ahuachapán	Turín	El Salvador	La Libertad	NA
**Central America and Caribbean**	El Salvador	Cabañas	Cinquera	El Salvador	La Libertad	Ciudad Arce
**Central America and Caribbean**	El Salvador	Cabañas	Tejutepeque	El Salvador	La Libertad	Colón
**Central America and Caribbean**	El Salvador	Chalatenango	NA	El Salvador	La Libertad	Jayaque
**Central America and Caribbean**	El Salvador	Chalatenango	Agua Caliente	El Salvador	La Libertad	Jicalapa
**Central America and Caribbean**	El Salvador	Chalatenango	Dulce Nombre de María	El Salvador	La Libertad	Sacacoyo

**Central America and Caribbean**	El Salvador	Chalatenango	Embalse Cerron Grande	El Salvador	La Libertad	San Pablo Tacachico
**Central America and Caribbean**	El Salvador	Chalatenango	La Palma	El Salvador	La Libertad	Teotepeque
**Central America and Caribbean**	El Salvador	Chalatenango	La Reina	El Salvador	La Libertad	Tepecoyo
**Central America and Caribbean**	El Salvador	Chalatenango	Nueva Concepción	El Salvador	Santa Ana	NA
**Central America and Caribbean**	El Salvador	Chalatenango	San Francisco Morazán	El Salvador	Santa Ana	Candelaria de la Frontera
**Central America and Caribbean**	El Salvador	Chalatenango	San Luis del Carmen	El Salvador	Santa Ana	Chalchuapa
**Central America and Caribbean**	El Salvador	Chalatenango	San Rafael	El Salvador	Santa Ana	Coatepeque
**Central America and Caribbean**	El Salvador	Chalatenango	Tejutla	El Salvador	Santa Ana	El Congo
**Central America and Caribbean**	El Salvador	Cuscatlán	NA	El Salvador	Santa Ana	El Porvenir
**Central America and Caribbean**	El Salvador	Cuscatlán	Oratorio de Concepción	El Salvador	Santa Ana	Lago de Coatepeque
**Central America and Caribbean**	El Salvador	Cuscatlán	San Bartolomé Perulapía	El Salvador	Santa Ana	Lago de Guija
**Central America and Caribbean**	El Salvador	Cuscatlán	San José Guayabal	El Salvador	Santa Ana	Masahuat
**Central America and Caribbean**	El Salvador	Cuscatlán	Suchitoto	El Salvador	Santa Ana	Metapán
**Central America and Caribbean**	El Salvador	Cuscatlán	Tenancingo	El Salvador	Santa Ana	San Antonio Pajonal
**Central America and Caribbean**	El Salvador	La Libertad	NA	El Salvador	Santa Ana	San Sebastián Salitrillo

**Central America and Caribbean**	El Salvador	La Libertad	Antiguo Cuscatlán	El Salvador	Santa Ana	Santa Ana
**Central America and Caribbean**	El Salvador	La Libertad	Chiltiupán	El Salvador	Santa Ana	Santiago de la Frontera
**Central America and Caribbean**	El Salvador	La Libertad	Ciudad Arce	El Salvador	Santa Ana	Texistepeque
**Central America and Caribbean**	El Salvador	La Libertad	Colón	El Salvador	Sonsonate	NA
**Central America and Caribbean**	El Salvador	La Libertad	Comasagua	El Salvador	Sonsonate	Acajutla
**Central America and Caribbean**	El Salvador	La Libertad	Huizúcar	El Salvador	Sonsonate	Armenia
**Central America and Caribbean**	El Salvador	La Libertad	Jayaque	El Salvador	Sonsonate	Caluco
**Central America and Caribbean**	El Salvador	La Libertad	Jicalapa	El Salvador	Sonsonate	Cuisnahuat
**Central America and Caribbean**	El Salvador	La Libertad	Nueva San Salvador	El Salvador	Sonsonate	Izalco
**Central America and Caribbean**	El Salvador	La Libertad	Opico	El Salvador	Sonsonate	Juayúa
**Central America and Caribbean**	El Salvador	La Libertad	Quezaltepeque	El Salvador	Sonsonate	Nahuizalco
**Central America and Caribbean**	El Salvador	La Libertad	Sacacoyo	El Salvador	Sonsonate	Nahulingo
**Central America and Caribbean**	El Salvador	La Libertad	San José Villanueva	El Salvador	Sonsonate	Salcoatitán
**Central America and Caribbean**	El Salvador	La Libertad	San Matías	El Salvador	Sonsonate	San Antonio del Monte
**Central America and Caribbean**	El Salvador	La Libertad	San Pablo Tacachico	El Salvador	Sonsonate	San Julián

**Central America and Caribbean**	El Salvador	La Libertad	Talnique	El Salvador	Sonsonate	Santa Catarina Masahuat
**Central America and Caribbean**	El Salvador	La Libertad	Tamanique	El Salvador	Sonsonate	Santa Isabel Ishuatán
**Central America and Caribbean**	El Salvador	La Libertad	Teotepeque	El Salvador	Sonsonate	Santo Domingo
**Central America and Caribbean**	El Salvador	La Libertad	Tepecoyo	El Salvador	Sonsonate	Sonsonate
**Central America and Caribbean**	El Salvador	La Libertad	Zaragoza	El Salvador	Sonsonate	Sonzacate
**Central America and Caribbean**	El Salvador	La Paz	Olocuilta	Guatemala	Alta Verapaz	Cobán
**Central America and Caribbean**	El Salvador	San Salvador	NA	Guatemala	Alta Verapaz	San Cristóbal Verapaz
**Central America and Caribbean**	El Salvador	San Salvador	Aguilares	Guatemala	Alta Verapaz	San Juan Chamelco
**Central America and Caribbean**	El Salvador	San Salvador	Apopa	Guatemala	Alta Verapaz	Santa Cruz Verapaz
**Central America and Caribbean**	El Salvador	San Salvador	Ayutuxtepeque	Guatemala	Alta Verapaz	Tactic
**Central America and Caribbean**	El Salvador	San Salvador	Cuscatancingo	Guatemala	Baja Verapaz	NA
**Central America and Caribbean**	El Salvador	San Salvador	Delgado	Guatemala	Baja Verapaz	Cubulco
**Central America and Caribbean**	El Salvador	San Salvador	El Paisnal	Guatemala	Baja Verapaz	Granados
**Central America and Caribbean**	El Salvador	San Salvador	Guazapa	Guatemala	Baja Verapaz	Rabinal
**Central America and Caribbean**	El Salvador	San Salvador	Ilopango	Guatemala	Baja Verapaz	San Miguel Chicaj

**Central America and Caribbean**	El Salvador	San Salvador	Mejicanos	Guatemala	Baja Verapaz	Santa Cruz El Chol
**Central America and Caribbean**	El Salvador	San Salvador	Nejapa	Guatemala	Chimaltenango	NA
**Central America and Caribbean**	El Salvador	San Salvador	Panchimalco	Guatemala	Chimaltenango	Acatenango
**Central America and Caribbean**	El Salvador	San Salvador	Rosario de Mora	Guatemala	Chimaltenango	Chimaltenango
**Central America and Caribbean**	El Salvador	San Salvador	San Marcos	Guatemala	Chimaltenango	Comalapa
**Central America and Caribbean**	El Salvador	San Salvador	San Martín	Guatemala	Chimaltenango	El Tejar
**Central America and Caribbean**	El Salvador	San Salvador	San Salvador	Guatemala	Chimaltenango	Parramos
**Central America and Caribbean**	El Salvador	San Salvador	Soyapango	Guatemala	Chimaltenango	Patzicía
**Central America and Caribbean**	El Salvador	San Salvador	Tonacatepeque	Guatemala	Chimaltenango	Patzún
**Central America and Caribbean**	El Salvador	Santa Ana	NA	Guatemala	Chimaltenango	Pochuta
**Central America and Caribbean**	El Salvador	Santa Ana	Candelaria de la Frontera	Guatemala	Chimaltenango	San Andrés Itzapa
**Central America and Caribbean**	El Salvador	Santa Ana	Chalchuapa	Guatemala	Chimaltenango	San José Poaquil
**Central America and Caribbean**	El Salvador	Santa Ana	Coatepeque	Guatemala	Chimaltenango	San Martín Jilotepeque
**Central America and Caribbean**	El Salvador	Santa Ana	El Congo	Guatemala	Chimaltenango	Santa Apolonia
**Central America and Caribbean**	El Salvador	Santa Ana	El Porvenir	Guatemala	Chimaltenango	Santa Cruz Balanyá

**Central America and Caribbean**	El Salvador	Santa Ana	Lago de Coatepeque	Guatemala	Chimaltenango	Tecpán Guatemala
**Central America and Caribbean**	El Salvador	Santa Ana	Lago de Guija	Guatemala	Chimaltenango	Yepocapa
**Central America and Caribbean**	El Salvador	Santa Ana	Masahuat	Guatemala	Chimaltenango	Zaragoza
**Central America and Caribbean**	El Salvador	Santa Ana	Metapán	Guatemala	Escuintla	Escuintla
**Central America and Caribbean**	El Salvador	Santa Ana	San Antonio Pajonal	Guatemala	Escuintla	Palín
**Central America and Caribbean**	El Salvador	Santa Ana	San Sebastián Salitrillo	Guatemala	Escuintla	San Vicente Pacaya
**Central America and Caribbean**	El Salvador	Santa Ana	Santa Ana	Guatemala	Guatemala	NA
**Central America and Caribbean**	El Salvador	Santa Ana	Santa Rosa Guachipilín	Guatemala	Guatemala	Amatitlán
**Central America and Caribbean**	El Salvador	Santa Ana	Santiago de la Frontera	Guatemala	Guatemala	Chinautla
**Central America and Caribbean**	El Salvador	Santa Ana	Texistepeque	Guatemala	Guatemala	Chuarrancho
**Central America and Caribbean**	El Salvador	Sonsonate	NA	Guatemala	Guatemala	Fraijanes
**Central America and Caribbean**	El Salvador	Sonsonate	Acajutla	Guatemala	Guatemala	Mixco
**Central America and Caribbean**	El Salvador	Sonsonate	Armenia	Guatemala	Guatemala	Palencia
**Central America and Caribbean**	El Salvador	Sonsonate	Caluco	Guatemala	Guatemala	Petapa
**Central America and Caribbean**	El Salvador	Sonsonate	Cuisnahuat	Guatemala	Guatemala	San José Pinula

**Central America and Caribbean**	El Salvador	Sonsonate	Izalco	Guatemala	Guatemala	San José del Golfo
**Central America and Caribbean**	El Salvador	Sonsonate	Juayúa	Guatemala	Guatemala	San Juan Sacatepéquez
**Central America and Caribbean**	El Salvador	Sonsonate	Nahuizalco	Guatemala	Guatemala	San Pedro Ayampuc
**Central America and Caribbean**	El Salvador	Sonsonate	Nahulingo	Guatemala	Guatemala	San Pedro Sacatepéquez
**Central America and Caribbean**	El Salvador	Sonsonate	Salcoatitán	Guatemala	Guatemala	San Raymundo
**Central America and Caribbean**	El Salvador	Sonsonate	San Antonio del Monte	Guatemala	Guatemala	Santa Catarina Pinula
**Central America and Caribbean**	El Salvador	Sonsonate	San Julián	Guatemala	Guatemala	Villa Canales
**Central America and Caribbean**	El Salvador	Sonsonate	Santa Catarina Masahuat	Guatemala	Guatemala	Villa Nueva
**Central America and Caribbean**	El Salvador	Sonsonate	Santa Isabel Ishuatán	Guatemala	Guatemala	ZONA 1
**Central America and Caribbean**	El Salvador	Sonsonate	Santo Domingo	Guatemala	Guatemala	ZONA 10
**Central America and Caribbean**	El Salvador	Sonsonate	Sonsonate	Guatemala	Guatemala	ZONA 11
**Central America and Caribbean**	El Salvador	Sonsonate	Sonzacate	Guatemala	Guatemala	ZONA 12
**Central America and Caribbean**	Guatemala	Alta Verapaz	Cobán	Guatemala	Guatemala	ZONA 13
**Central America and Caribbean**	Guatemala	Alta Verapaz	San Cristóbal Verapaz	Guatemala	Guatemala	ZONA 14
**Central America and Caribbean**	Guatemala	Alta Verapaz	San Juan Chamelco	Guatemala	Guatemala	ZONA 15

**Central America and Caribbean**	Guatemala	Alta Verapaz	Santa Cruz Verapaz	Guatemala	Guatemala	ZONA 16
**Central America and Caribbean**	Guatemala	Alta Verapaz	Tactic	Guatemala	Guatemala	ZONA 17
**Central America and Caribbean**	Guatemala	Baja Verapaz	NA	Guatemala	Guatemala	ZONA 18
**Central America and Caribbean**	Guatemala	Baja Verapaz	Cubulco	Guatemala	Guatemala	ZONA 19
**Central America and Caribbean**	Guatemala	Baja Verapaz	Granados	Guatemala	Guatemala	ZONA 2
**Central America and Caribbean**	Guatemala	Baja Verapaz	Rabinal	Guatemala	Guatemala	ZONA 22
**Central America and Caribbean**	Guatemala	Baja Verapaz	San Miguel Chicaj	Guatemala	Guatemala	ZONA 24
**Central America and Caribbean**	Guatemala	Baja Verapaz	Santa Cruz El Chol	Guatemala	Guatemala	ZONA 25
**Central America and Caribbean**	Guatemala	Chimaltenango	NA	Guatemala	Guatemala	ZONA 3
**Central America and Caribbean**	Guatemala	Chimaltenango	Acatenango	Guatemala	Guatemala	ZONA 4
**Central America and Caribbean**	Guatemala	Chimaltenango	Chimaltenango	Guatemala	Guatemala	ZONA 5
**Central America and Caribbean**	Guatemala	Chimaltenango	Comalapa	Guatemala	Guatemala	ZONA 6
**Central America and Caribbean**	Guatemala	Chimaltenango	El Tejar	Guatemala	Guatemala	ZONA 7
**Central America and Caribbean**	Guatemala	Chimaltenango	Parramos	Guatemala	Guatemala	ZONA 8
**Central America and Caribbean**	Guatemala	Chimaltenango	Patzicía	Guatemala	Guatemala	ZONA 9

**Central America and Caribbean**	Guatemala	Chimaltenango	Patzún	Guatemala	Huehuetenango	NA
**Central America and Caribbean**	Guatemala	Chimaltenango	San Andrés Itzapa	Guatemala	Huehuetenango	Aguacatán
**Central America and Caribbean**	Guatemala	Chimaltenango	San José Poaquil	Guatemala	Huehuetenango	Chiantla
**Central America and Caribbean**	Guatemala	Chimaltenango	San Martín Jilotepeque	Guatemala	Huehuetenango	Colotenango
**Central America and Caribbean**	Guatemala	Chimaltenango	Santa Apolonia	Guatemala	Huehuetenango	Concepción Huista
**Central America and Caribbean**	Guatemala	Chimaltenango	Santa Cruz Balanyá	Guatemala	Huehuetenango	Cuilco
**Central America and Caribbean**	Guatemala	Chimaltenango	Tecpán Guatemala	Guatemala	Huehuetenango	Huehuetenango
**Central America and Caribbean**	Guatemala	Chimaltenango	Zaragoza	Guatemala	Huehuetenango	Jacaltenango
**Central America and Caribbean**	Guatemala	El Progreso	San Antonio La Paz	Guatemala	Huehuetenango	La Democracia
**Central America and Caribbean**	Guatemala	Guatemala	NA	Guatemala	Huehuetenango	La Libertad
**Central America and Caribbean**	Guatemala	Guatemala	Amatitlán	Guatemala	Huehuetenango	Malacatancito
**Central America and Caribbean**	Guatemala	Guatemala	Chinautla	Guatemala	Huehuetenango	San Antonio Huista
**Central America and Caribbean**	Guatemala	Guatemala	Chuarrancho	Guatemala	Huehuetenango	San Gaspar Ixchil
**Central America and Caribbean**	Guatemala	Guatemala	Fraijanes	Guatemala	Huehuetenango	San Ildefonso Ixtahuacán
**Central America and Caribbean**	Guatemala	Guatemala	Mixco	Guatemala	Huehuetenango	San Juan Atitán

**Central America and Caribbean**	Guatemala	Guatemala	Palencia	Guatemala	Huehuetenango	San Juan Ixcoy
**Central America and Caribbean**	Guatemala	Guatemala	Petapa	Guatemala	Huehuetenango	San Miguel Acatán
**Central America and Caribbean**	Guatemala	Guatemala	San José Pinula	Guatemala	Huehuetenango	San Pedro Necta
**Central America and Caribbean**	Guatemala	Guatemala	San José del Golfo	Guatemala	Huehuetenango	San Rafael La Independencia
**Central America and Caribbean**	Guatemala	Guatemala	San Juan Sacatepéquez	Guatemala	Huehuetenango	San Rafael Petzal
**Central America and Caribbean**	Guatemala	Guatemala	San Pedro Ayampuc	Guatemala	Huehuetenango	San Sebastián Coatán
**Central America and Caribbean**	Guatemala	Guatemala	San Pedro Sacatepéquez	Guatemala	Huehuetenango	San Sebastián Huehuetenango
**Central America and Caribbean**	Guatemala	Guatemala	San Raymundo	Guatemala	Huehuetenango	Santa Ana Huista
**Central America and Caribbean**	Guatemala	Guatemala	Santa Catarina Pinula	Guatemala	Huehuetenango	Santa Bárbara
**Central America and Caribbean**	Guatemala	Guatemala	Villa Canales	Guatemala	Huehuetenango	Santa Eulalia
**Central America and Caribbean**	Guatemala	Guatemala	Villa Nueva	Guatemala	Huehuetenango	Santiago Chimaltenango
**Central America and Caribbean**	Guatemala	Guatemala	ZONA 1	Guatemala	Huehuetenango	Soloma
**Central America and Caribbean**	Guatemala	Guatemala	ZONA 10	Guatemala	Huehuetenango	Tectitán
**Central America and Caribbean**	Guatemala	Guatemala	ZONA 11	Guatemala	Huehuetenango	Todos Santos Cuchumatán
**Central America and Caribbean**	Guatemala	Guatemala	ZONA 12	Guatemala	Quezaltenango	NA

**Central America and Caribbean**	Guatemala	Guatemala	ZONA 13	Guatemala	Quezaltenango	Almolonga
**Central America and Caribbean**	Guatemala	Guatemala	ZONA 14	Guatemala	Quezaltenango	Cabricán
**Central America and Caribbean**	Guatemala	Guatemala	ZONA 15	Guatemala	Quezaltenango	Cajolá
**Central America and Caribbean**	Guatemala	Guatemala	ZONA 16	Guatemala	Quezaltenango	Cantel
**Central America and Caribbean**	Guatemala	Guatemala	ZONA 17	Guatemala	Quezaltenango	Concepción Chiquirichapa
**Central America and Caribbean**	Guatemala	Guatemala	ZONA 18	Guatemala	Quezaltenango	El Palmar
**Central America and Caribbean**	Guatemala	Guatemala	ZONA 19	Guatemala	Quezaltenango	Huitán
**Central America and Caribbean**	Guatemala	Guatemala	ZONA 2	Guatemala	Quezaltenango	La Esperanza
**Central America and Caribbean**	Guatemala	Guatemala	ZONA 22	Guatemala	Quezaltenango	Olintepeque
**Central America and Caribbean**	Guatemala	Guatemala	ZONA 24	Guatemala	Quezaltenango	Ostuncalco
**Central America and Caribbean**	Guatemala	Guatemala	ZONA 25	Guatemala	Quezaltenango	Palestina de Los Altos
**Central America and Caribbean**	Guatemala	Guatemala	ZONA 3	Guatemala	Quezaltenango	Quetzaltenango
**Central America and Caribbean**	Guatemala	Guatemala	ZONA 4	Guatemala	Quezaltenango	Salcajá
**Central America and Caribbean**	Guatemala	Guatemala	ZONA 5	Guatemala	Quezaltenango	San Carlos Sija
**Central America and Caribbean**	Guatemala	Guatemala	ZONA 6	Guatemala	Quezaltenango	San Francisco La Unión

**Central America and Caribbean**	Guatemala	Guatemala	ZONA 7	Guatemala	Quezaltenango	San Martín Sacatepéquez
**Central America and Caribbean**	Guatemala	Guatemala	ZONA 8	Guatemala	Quezaltenango	San Mateo
**Central America and Caribbean**	Guatemala	Guatemala	ZONA 9	Guatemala	Quezaltenango	San Miguel Sigüilá
**Central America and Caribbean**	Guatemala	Huehuetenango	NA	Guatemala	Quezaltenango	Sibilia
**Central America and Caribbean**	Guatemala	Huehuetenango	Aguacatán	Guatemala	Quezaltenango	Zunil
**Central America and Caribbean**	Guatemala	Huehuetenango	Chiantla	Guatemala	Quiché	NA
**Central America and Caribbean**	Guatemala	Huehuetenango	Colotenango	Guatemala	Quiché	Canillá
**Central America and Caribbean**	Guatemala	Huehuetenango	Concepción Huista	Guatemala	Quiché	Chajul
**Central America and Caribbean**	Guatemala	Huehuetenango	Cuilco	Guatemala	Quiché	Chicaman
**Central America and Caribbean**	Guatemala	Huehuetenango	Huehuetenango	Guatemala	Quiché	Chichicastenango
**Central America and Caribbean**	Guatemala	Huehuetenango	Jacaltenango	Guatemala	Quiché	Chiché
**Central America and Caribbean**	Guatemala	Huehuetenango	La Democracia	Guatemala	Quiché	Chinique
**Central America and Caribbean**	Guatemala	Huehuetenango	La Libertad	Guatemala	Quiché	Cunén
**Central America and Caribbean**	Guatemala	Huehuetenango	Malacatancito	Guatemala	Quiché	Joyabaj
**Central America and Caribbean**	Guatemala	Huehuetenango	Nentón	Guatemala	Quiché	Nebaj

**Central America and Caribbean**	Guatemala	Huehuetenango	San Antonio Huista	Guatemala	Quiché	Pachalúm
**Central America and Caribbean**	Guatemala	Huehuetenango	San Gaspar Ixchil	Guatemala	Quiché	Patzité
**Central America and Caribbean**	Guatemala	Huehuetenango	San Ildefonso Ixtahuacán	Guatemala	Quiché	Sacapulas
**Central America and Caribbean**	Guatemala	Huehuetenango	San Juan Atitán	Guatemala	Quiché	San Andrés Sajcabajá
**Central America and Caribbean**	Guatemala	Huehuetenango	San Juan Ixcoy	Guatemala	Quiché	San Antonio Ilotenango
**Central America and Caribbean**	Guatemala	Huehuetenango	San Mateo Ixtatán	Guatemala	Quiché	San Bartolomé Jocotenango
**Central America and Caribbean**	Guatemala	Huehuetenango	San Miguel Acatán	Guatemala	Quiché	San Juan Cotzal
**Central America and Caribbean**	Guatemala	Huehuetenango	San Pedro Necta	Guatemala	Quiché	San Pedro Jocopilas
**Central America and Caribbean**	Guatemala	Huehuetenango	San Rafael La Independencia	Guatemala	Quiché	Santa Cruz del Quiché
**Central America and Caribbean**	Guatemala	Huehuetenango	San Rafael Petzal	Guatemala	Quiché	Uspantán
**Central America and Caribbean**	Guatemala	Huehuetenango	San Sebastián Coatán	Guatemala	Quiché	Zacualpa
**Central America and Caribbean**	Guatemala	Huehuetenango	San Sebastián Huehuetenango	Guatemala	Sacatepéquez	NA
**Central America and Caribbean**	Guatemala	Huehuetenango	Santa Ana Huista	Guatemala	Sacatepéquez	Antigua Guatemala
**Central America and Caribbean**	Guatemala	Huehuetenango	Santa Bárbara	Guatemala	Sacatepéquez	Ciudad Vieja
**Central America and Caribbean**	Guatemala	Huehuetenango	Santa Cruz Barillas	Guatemala	Sacatepéquez	Jocotenango

**Central America and Caribbean**	Guatemala	Huehuetenango	Santa Eulalia	Guatemala	Sacatepéquez	Magdalena Milpas Altas
**Central America and Caribbean**	Guatemala	Huehuetenango	Santiago Chimaltenango	Guatemala	Sacatepéquez	Pastores
**Central America and Caribbean**	Guatemala	Huehuetenango	Soloma	Guatemala	Sacatepéquez	San Antonio Aguas Calientes
**Central America and Caribbean**	Guatemala	Huehuetenango	Tectitán	Guatemala	Sacatepéquez	San Bartolomé Milpas Altas
**Central America and Caribbean**	Guatemala	Huehuetenango	Todos Santos Cuchumatán	Guatemala	Sacatepéquez	San Lucas Sacatepéquez
**Central America and Caribbean**	Guatemala	Quezaltenango	NA	Guatemala	Sacatepéquez	San Miguel Dueñas
**Central America and Caribbean**	Guatemala	Quezaltenango	Almolonga	Guatemala	Sacatepéquez	Santa Lucía Milpas Altas
**Central America and Caribbean**	Guatemala	Quezaltenango	Cabricán	Guatemala	Sacatepéquez	Santa María de Jesús
**Central America and Caribbean**	Guatemala	Quezaltenango	Cajolá	Guatemala	Sacatepéquez	Santiago Sacatepéquez
**Central America and Caribbean**	Guatemala	Quezaltenango	Cantel	Guatemala	Sacatepéquez	Santo Domingo Xenacoj
**Central America and Caribbean**	Guatemala	Quezaltenango	Concepción Chiquirichapa	Guatemala	Sacatepéquez	Sumpango
**Central America and Caribbean**	Guatemala	Quezaltenango	Huitán	Guatemala	San Marcos	NA
**Central America and Caribbean**	Guatemala	Quezaltenango	La Esperanza	Guatemala	San Marcos	Comitancillo
**Central America and Caribbean**	Guatemala	Quezaltenango	Olintepeque	Guatemala	San Marcos	Concepción Tutuapa
**Central America and Caribbean**	Guatemala	Quezaltenango	Ostuncalco	Guatemala	San Marcos	El Tumbador

**Central America and Caribbean**	Guatemala	Quezaltenango	Palestina de Los Altos	Guatemala	San Marcos	Esquipulas Palo Gordo
**Central America and Caribbean**	Guatemala	Quezaltenango	Quetzaltenango	Guatemala	San Marcos	Ixchiguan
**Central America and Caribbean**	Guatemala	Quezaltenango	Salcajá	Guatemala	San Marcos	La Reforma
**Central America and Caribbean**	Guatemala	Quezaltenango	San Carlos Sija	Guatemala	San Marcos	Río Blanco
**Central America and Caribbean**	Guatemala	Quezaltenango	San Francisco La Unión	Guatemala	San Marcos	San Antonio Sacatepéquez
**Central America and Caribbean**	Guatemala	Quezaltenango	San Martín Sacatepéquez	Guatemala	San Marcos	San Cristobal Cucho
**Central America and Caribbean**	Guatemala	Quezaltenango	San Mateo	Guatemala	San Marcos	San José Ojetenam
**Central America and Caribbean**	Guatemala	Quezaltenango	San Miguel Sigüilá	Guatemala	San Marcos	San Lorenzo
**Central America and Caribbean**	Guatemala	Quezaltenango	Sibilia	Guatemala	San Marcos	San Marcos
**Central America and Caribbean**	Guatemala	Quezaltenango	Zunil	Guatemala	San Marcos	San Miguel Ixtahuacán
**Central America and Caribbean**	Guatemala	Quiché	NA	Guatemala	San Marcos	San Pablo
**Central America and Caribbean**	Guatemala	Quiché	Canillá	Guatemala	San Marcos	San Pedro Sacatepéquez
**Central America and Caribbean**	Guatemala	Quiché	Chajul	Guatemala	San Marcos	San Rafaél Pie de la Cuesta
**Central America and Caribbean**	Guatemala	Quiché	Chicaman	Guatemala	San Marcos	San Sibinal
**Central America and Caribbean**	Guatemala	Quiché	Chichicastenango	Guatemala	San Marcos	Sipacapa

**Central America and Caribbean**	Guatemala	Quiché	Chiché	Guatemala	San Marcos	Tacaná
**Central America and Caribbean**	Guatemala	Quiché	Chinique	Guatemala	San Marcos	Tajumulco
**Central America and Caribbean**	Guatemala	Quiché	Cunén	Guatemala	San Marcos	Tejutla
**Central America and Caribbean**	Guatemala	Quiché	Joyabaj	Guatemala	Santa Rosa	Nueva Santa Rosa
**Central America and Caribbean**	Guatemala	Quiché	Nebaj	Guatemala	Santa Rosa	Santa Cruz Naranjo
**Central America and Caribbean**	Guatemala	Quiché	Pachalúm	Guatemala	Santa Rosa	Santa Rosa de Lima
**Central America and Caribbean**	Guatemala	Quiché	Patzité	Guatemala	Sololá	NA
**Central America and Caribbean**	Guatemala	Quiché	Sacapulas	Guatemala	Sololá	NA
**Central America and Caribbean**	Guatemala	Quiché	San Andrés Sajcabajá	Guatemala	Sololá	Concepción
**Central America and Caribbean**	Guatemala	Quiché	San Antonio Ilotenango	Guatemala	Sololá	Nahualá
**Central America and Caribbean**	Guatemala	Quiché	San Bartolomé Jocotenango	Guatemala	Sololá	Panajachel
**Central America and Caribbean**	Guatemala	Quiché	San Juan Cotzal	Guatemala	Sololá	San Andrés Semetabaj
**Central America and Caribbean**	Guatemala	Quiché	San Pedro Jocopilas	Guatemala	Sololá	San Antonio Palopó
**Central America and Caribbean**	Guatemala	Quiché	Santa Cruz del Quiché	Guatemala	Sololá	San José Chacayá
**Central America and Caribbean**	Guatemala	Quiché	Uspantán	Guatemala	Sololá	San Juan La Laguna

**Central America and Caribbean**	Guatemala	Quiché	Zacualpa	Guatemala	Sololá	San Lucas Tolimán
**Central America and Caribbean**	Guatemala	Sacatepéquez	NA	Guatemala	Sololá	San Marcos La Laguna
**Central America and Caribbean**	Guatemala	Sacatepéquez	Antigua Guatemala	Guatemala	Sololá	San Pablo La Laguna
**Central America and Caribbean**	Guatemala	Sacatepéquez	Ciudad Vieja	Guatemala	Sololá	San Pedro La Laguna
**Central America and Caribbean**	Guatemala	Sacatepéquez	Jocotenango	Guatemala	Sololá	Santa Catarina Ixtahuacan
**Central America and Caribbean**	Guatemala	Sacatepéquez	Magdalena Milpas Altas	Guatemala	Sololá	Santa Catarina Palopó
**Central America and Caribbean**	Guatemala	Sacatepéquez	Pastores	Guatemala	Sololá	Santa Clara La Laguna
**Central America and Caribbean**	Guatemala	Sacatepéquez	San Antonio Aguas Calientes	Guatemala	Sololá	Santa Cruz La Laguna
**Central America and Caribbean**	Guatemala	Sacatepéquez	San Bartolomé Milpas Altas	Guatemala	Sololá	Santa Lucía Utatlán
**Central America and Caribbean**	Guatemala	Sacatepéquez	San Lucas Sacatepéquez	Guatemala	Sololá	Santa María Visitación
**Central America and Caribbean**	Guatemala	Sacatepéquez	San Miguel Dueñas	Guatemala	Sololá	Santiago Atitlán
**Central America and Caribbean**	Guatemala	Sacatepéquez	Santa Catarina Barahona	Guatemala	Sololá	Sololá
**Central America and Caribbean**	Guatemala	Sacatepéquez	Santa Lucía Milpas Altas	Guatemala	Suchitepéquez	NA
**Central America and Caribbean**	Guatemala	Sacatepéquez	Santa María de Jesús	Guatemala	Suchitepéquez	Chicacao
**Central America and Caribbean**	Guatemala	Sacatepéquez	Santiago Sacatepéquez	Guatemala	Suchitepéquez	Samayac

**Central America and Caribbean**	Guatemala	Sacatepéquez	Santo Domingo Xenacoj	Guatemala	Totonicapán	NA
**Central America and Caribbean**	Guatemala	Sacatepéquez	Sumpango	Guatemala	Totonicapán	Momostenango
**Central America and Caribbean**	Guatemala	San Marcos	NA	Guatemala	Totonicapán	San Andrés Xecul
**Central America and Caribbean**	Guatemala	San Marcos	Comitancillo	Guatemala	Totonicapán	San Bartolo
**Central America and Caribbean**	Guatemala	San Marcos	Concepción Tutuapa	Guatemala	Totonicapán	San Cristóbal Totonicapán
**Central America and Caribbean**	Guatemala	San Marcos	El Tumbador	Guatemala	Totonicapán	San Francisco El Alto
**Central America and Caribbean**	Guatemala	San Marcos	Esquipulas Palo Gordo	Guatemala	Totonicapán	Santa Lucía La Reforma
**Central America and Caribbean**	Guatemala	San Marcos	Ixchiguan	Guatemala	Totonicapán	Santa María Chiquimula
**Central America and Caribbean**	Guatemala	San Marcos	La Reforma	Guatemala	Totonicapán	Totonicapán
**Central America and Caribbean**	Guatemala	San Marcos	Nuevo Progreso	Haiti	Nord-Est	Vallières
**Central America and Caribbean**	Guatemala	San Marcos	Río Blanco	Honduras	Copán	NA
**Central America and Caribbean**	Guatemala	San Marcos	San Antonio Sacatepéquez	Honduras	Copán	Cabañas
**Central America and Caribbean**	Guatemala	San Marcos	San Cristobal Cucho	Honduras	Copán	Concepción
**Central America and Caribbean**	Guatemala	San Marcos	San José Ojetenam	Honduras	Copán	Copán Ruinas
**Central America and Caribbean**	Guatemala	San Marcos	San Lorenzo	Honduras	Copán	La Unión

**Central America and Caribbean**	Guatemala	San Marcos	San Marcos	Honduras	Copán	Santa Rita
**Central America and Caribbean**	Guatemala	San Marcos	San Miguel Ixtahuacán	Honduras	Lempira	Cololaca
**Central America and Caribbean**	Guatemala	San Marcos	San Pablo	Honduras	Lempira	Guarita
**Central America and Caribbean**	Guatemala	San Marcos	San Pedro Sacatepéquez	Honduras	Lempira	La Virtud
**Central America and Caribbean**	Guatemala	San Marcos	San Rafaél Pie de la Cuesta	Honduras	Lempira	Mapulaca
**Central America and Caribbean**	Guatemala	San Marcos	San Sibinal	Honduras	Lempira	San Sebastian
**Central America and Caribbean**	Guatemala	San Marcos	Sipacapa	Honduras	Lempira	Talgua
**Central America and Caribbean**	Guatemala	San Marcos	Tacaná	Honduras	Lempira	Virginia
**Central America and Caribbean**	Guatemala	San Marcos	Tajumulco	Honduras	Ocotepeque	NA
**Central America and Caribbean**	Guatemala	San Marcos	Tejutla	Honduras	Ocotepeque	Belén Gualcho
**Central America and Caribbean**	Guatemala	Santa Rosa	Santa Cruz Naranjo	Honduras	Ocotepeque	Concepción
**Central America and Caribbean**	Guatemala	Santa Rosa	Santa Rosa de Lima	Honduras	Ocotepeque	Dolores Merendon
**Central America and Caribbean**	Guatemala	Sololá	NA	Honduras	Ocotepeque	Fraternidad
**Central America and Caribbean**	Guatemala	Sololá	NA	Honduras	Ocotepeque	La Encarnación
**Central America and Caribbean**	Guatemala	Sololá	Concepción	Honduras	Ocotepeque	La Labor

**Central America and Caribbean**	Guatemala	Sololá	Nahualá	Honduras	Ocotepeque	Lucerna
**Central America and Caribbean**	Guatemala	Sololá	Panajachel	Honduras	Ocotepeque	Mercedes
**Central America and Caribbean**	Guatemala	Sololá	San Andrés Semetabaj	Honduras	Ocotepeque	Ocotepeque
**Central America and Caribbean**	Guatemala	Sololá	San Antonio Palopó	Honduras	Ocotepeque	San Fernando
**Central America and Caribbean**	Guatemala	Sololá	San José Chacayá	Honduras	Ocotepeque	San Francisco del Valle
**Central America and Caribbean**	Guatemala	Sololá	San Juan La Laguna	Honduras	Ocotepeque	San Jorge
**Central America and Caribbean**	Guatemala	Sololá	San Lucas Tolimán	Honduras	Ocotepeque	San Marcos
**Central America and Caribbean**	Guatemala	Sololá	San Marcos La Laguna	Honduras	Ocotepeque	Santa Fé
**Central America and Caribbean**	Guatemala	Sololá	San Pablo La Laguna	Honduras	Ocotepeque	Sensenti
**Central America and Caribbean**	Guatemala	Sololá	San Pedro La Laguna	Honduras	Ocotepeque	Sinuapa
**Central America and Caribbean**	Guatemala	Sololá	Santa Catarina Ixtahuacan	Mexico	Chiapas	Amatenango De La Frontera
**Central America and Caribbean**	Guatemala	Sololá	Santa Catarina Palopó	Mexico	Chiapas	Bejucal De Ocampo
**Central America and Caribbean**	Guatemala	Sololá	Santa Clara La Laguna	Mexico	Chiapas	Bella Vista
**Central America and Caribbean**	Guatemala	Sololá	Santa Cruz La Laguna	Mexico	Chiapas	Cacahoatan
**Central America and Caribbean**	Guatemala	Sololá	Santa Lucía Utatlán	Mexico	Chiapas	Chicomuselo

**Central America and Caribbean**	Guatemala	Sololá	Santa María Visitación	Mexico	Chiapas	Comitan De Dominguez
**Central America and Caribbean**	Guatemala	Sololá	Santiago Atitlán	Mexico	Chiapas	El Porvenir
**Central America and Caribbean**	Guatemala	Sololá	Sololá	Mexico	Chiapas	Escuintla
**Central America and Caribbean**	Guatemala	Totonicapán	NA	Mexico	Chiapas	Frontera Comalapa
**Central America and Caribbean**	Guatemala	Totonicapán	Momostenango	Mexico	Chiapas	Frontera Hidalgo
**Central America and Caribbean**	Guatemala	Totonicapán	San Andrés Xecul	Mexico	Chiapas	Huehuetan
**Central America and Caribbean**	Guatemala	Totonicapán	San Bartolo	Mexico	Chiapas	Huixtla
**Central America and Caribbean**	Guatemala	Totonicapán	San Cristóbal Totonicapán	Mexico	Chiapas	La Grandeza
**Central America and Caribbean**	Guatemala	Totonicapán	San Francisco El Alto	Mexico	Chiapas	La Independencia
**Central America and Caribbean**	Guatemala	Totonicapán	Santa Lucía La Reforma	Mexico	Chiapas	La Trinitaria
**Central America and Caribbean**	Guatemala	Totonicapán	Santa María Chiquimula	Mexico	Chiapas	Las Margaritas
**Central America and Caribbean**	Guatemala	Totonicapán	Totonicapán	Mexico	Chiapas	Maravilla Tenejapa
**Central America and Caribbean**	Honduras	Copán	NA	Mexico	Chiapas	Mazapa De Madero
**Central America and Caribbean**	Honduras	Copán	Cabañas	Mexico	Chiapas	Mazatan
**Central America and Caribbean**	Honduras	Copán	Concepción	Mexico	Chiapas	Metapa

**Central America and Caribbean**	Honduras	Copán	Copán Ruinas	Mexico	Chiapas	Motozintla
**Central America and Caribbean**	Honduras	Copán	Corquín	Mexico	Chiapas	Siltepec
**Central America and Caribbean**	Honduras	Copán	Cucuyagua	Mexico	Chiapas	Tapachula
**Central America and Caribbean**	Honduras	Copán	Dolores	Mexico	Chiapas	Tuxtla Chico
**Central America and Caribbean**	Honduras	Copán	Dulce Nombre	Mexico	Chiapas	Tuzantan
**Central America and Caribbean**	Honduras	Copán	La Unión	Mexico	Chiapas	Tzimol
**Central America and Caribbean**	Honduras	Copán	San Agustín	Mexico	Chiapas	Union Juarez
**Central America and Caribbean**	Honduras	Copán	San Jerónimo	Mexico	Chiapas	Villa Comaltitlan
**Central America and Caribbean**	Honduras	Copán	San José	Mexico	Chihuahua	Ignacio Zaragoza
**Central America and Caribbean**	Honduras	Copán	San Juan de Opoa	Mexico	Chihuahua	Janos
**Central America and Caribbean**	Honduras	Copán	San Nicolás	Mexico	Durango	Coneto De Comonfort
**Central America and Caribbean**	Honduras	Copán	San Pedro	Mexico	Durango	Hidalgo
**Central America and Caribbean**	Honduras	Copán	Santa Rita	Mexico	Durango	Rodeo
**Central America and Caribbean**	Honduras	Copán	Santa Rosa de Copán	Mexico	Sinaloa	Choix
**Central America and Caribbean**	Honduras	Copán	Veracruz	Mexico	Zacatecas	Mazapil

**Central America and Caribbean**	Honduras	Intibucá	Colomoncagua	Mexico	Zacatecas	Melchor Ocampo
**Central America and Caribbean**	Honduras	Intibucá	Dolores	NA	NA	NA
**Central America and Caribbean**	Honduras	Intibucá	San Antonio	NA	NA	NA
**Central America and Caribbean**	Honduras	Intibucá	San Juan	NA	NA	NA
**Central America and Caribbean**	Honduras	Intibucá	San Miguelito	NA	NA	NA
**Central America and Caribbean**	Honduras	Intibucá	Santa Lucía	NA	NA	NA
**Central America and Caribbean**	Honduras	Intibucá	Yamaranguila	NA	NA	NA
**Central America and Caribbean**	Honduras	Lempira	NA	NA	NA	NA
**Central America and Caribbean**	Honduras	Lempira	Candelaria	NA	NA	NA
**Central America and Caribbean**	Honduras	Lempira	Cololaca	NA	NA	NA
**Central America and Caribbean**	Honduras	Lempira	Erandique	NA	NA	NA
**Central America and Caribbean**	Honduras	Lempira	Guarita	NA	NA	NA
**Central America and Caribbean**	Honduras	Lempira	La Campa	NA	NA	NA
**Central America and Caribbean**	Honduras	Lempira	La Virtud	NA	NA	NA
**Central America and Caribbean**	Honduras	Lempira	Mapulaca	NA	NA	NA

**Central America and Caribbean**	Honduras	Lempira	San Andrés	NA	NA	NA
**Central America and Caribbean**	Honduras	Lempira	San Juan Guarita	NA	NA	NA
**Central America and Caribbean**	Honduras	Lempira	San Manuel Colohete	NA	NA	NA
**Central America and Caribbean**	Honduras	Lempira	San Marcos de Caiquín	NA	NA	NA
**Central America and Caribbean**	Honduras	Lempira	San Sebastian	NA	NA	NA
**Central America and Caribbean**	Honduras	Lempira	Santa Cruz	NA	NA	NA
**Central America and Caribbean**	Honduras	Lempira	Talgua	NA	NA	NA
**Central America and Caribbean**	Honduras	Lempira	Tambla	NA	NA	NA
**Central America and Caribbean**	Honduras	Lempira	Tomalá	NA	NA	NA
**Central America and Caribbean**	Honduras	Lempira	Valladolid	NA	NA	NA
**Central America and Caribbean**	Honduras	Lempira	Virginia	NA	NA	NA
**Central America and Caribbean**	Honduras	Ocotepeque	NA	NA	NA	NA
**Central America and Caribbean**	Honduras	Ocotepeque	Belén Gualcho	NA	NA	NA
**Central America and Caribbean**	Honduras	Ocotepeque	Concepción	NA	NA	NA
**Central America and Caribbean**	Honduras	Ocotepeque	Dolores Merendon	NA	NA	NA

**Central America and Caribbean**	Honduras	Ocotepeque	Fraternidad	NA	NA	NA
**Central America and Caribbean**	Honduras	Ocotepeque	La Encarnación	NA	NA	NA
**Central America and Caribbean**	Honduras	Ocotepeque	La Labor	NA	NA	NA
**Central America and Caribbean**	Honduras	Ocotepeque	Lucerna	NA	NA	NA
**Central America and Caribbean**	Honduras	Ocotepeque	Mercedes	NA	NA	NA
**Central America and Caribbean**	Honduras	Ocotepeque	Ocotepeque	NA	NA	NA
**Central America and Caribbean**	Honduras	Ocotepeque	San Fernando	NA	NA	NA
**Central America and Caribbean**	Honduras	Ocotepeque	San Francisco del Valle	NA	NA	NA
**Central America and Caribbean**	Honduras	Ocotepeque	San Jorge	NA	NA	NA
**Central America and Caribbean**	Honduras	Ocotepeque	San Marcos	NA	NA	NA
**Central America and Caribbean**	Honduras	Ocotepeque	Santa Fé	NA	NA	NA
**Central America and Caribbean**	Honduras	Ocotepeque	Sensenti	NA	NA	NA
**Central America and Caribbean**	Honduras	Ocotepeque	Sinuapa	NA	NA	NA
**Central America and Caribbean**	Mexico	NA	NA	NA	NA	NA
**Central America and Caribbean**	Mexico	Aguascalientes	NA	NA	NA	NA

**Central America and Caribbean**	Mexico	Aguascalientes	Aguascalientes	NA	NA	NA
**Central America and Caribbean**	Mexico	Aguascalientes	Asientos	NA	NA	NA
**Central America and Caribbean**	Mexico	Aguascalientes	Cosio	NA	NA	NA
**Central America and Caribbean**	Mexico	Aguascalientes	El Llano	NA	NA	NA
**Central America and Caribbean**	Mexico	Aguascalientes	Jesus Maria	NA	NA	NA
**Central America and Caribbean**	Mexico	Aguascalientes	Pabellon De Arteaga	NA	NA	NA
**Central America and Caribbean**	Mexico	Aguascalientes	San Francisco De Los Romo	NA	NA	NA
**Central America and Caribbean**	Mexico	Aguascalientes	San Jose De Gracia	NA	NA	NA
**Central America and Caribbean**	Mexico	Baja California	Mexicali	NA	NA	NA
**Central America and Caribbean**	Mexico	Chiapas	Acacoyagua	NA	NA	NA
**Central America and Caribbean**	Mexico	Chiapas	Acapetahua	NA	NA	NA
**Central America and Caribbean**	Mexico	Chiapas	Amatenango De La Frontera	NA	NA	NA
**Central America and Caribbean**	Mexico	Chiapas	Bejucal De Ocampo	NA	NA	NA
**Central America and Caribbean**	Mexico	Chiapas	Bella Vista	NA	NA	NA
**Central America and Caribbean**	Mexico	Chiapas	Cacahoatan	NA	NA	NA

**Central America and Caribbean**	Mexico	Chiapas	Chicomuselo	NA	NA	NA
**Central America and Caribbean**	Mexico	Chiapas	Comitan De Dominguez	NA	NA	NA
**Central America and Caribbean**	Mexico	Chiapas	El Porvenir	NA	NA	NA
**Central America and Caribbean**	Mexico	Chiapas	Escuintla	NA	NA	NA
**Central America and Caribbean**	Mexico	Chiapas	Frontera Comalapa	NA	NA	NA
**Central America and Caribbean**	Mexico	Chiapas	Frontera Hidalgo	NA	NA	NA
**Central America and Caribbean**	Mexico	Chiapas	Huehuetan	NA	NA	NA
**Central America and Caribbean**	Mexico	Chiapas	Huixtla	NA	NA	NA
**Central America and Caribbean**	Mexico	Chiapas	La Grandeza	NA	NA	NA
**Central America and Caribbean**	Mexico	Chiapas	La Independencia	NA	NA	NA
**Central America and Caribbean**	Mexico	Chiapas	La Trinitaria	NA	NA	NA
**Central America and Caribbean**	Mexico	Chiapas	Las Margaritas	NA	NA	NA
**Central America and Caribbean**	Mexico	Chiapas	Maravilla Tenejapa	NA	NA	NA
**Central America and Caribbean**	Mexico	Chiapas	Mazapa De Madero	NA	NA	NA
**Central America and Caribbean**	Mexico	Chiapas	Mazatan	NA	NA	NA

**Central America and Caribbean**	Mexico	Chiapas	Metapa	NA	NA	NA
**Central America and Caribbean**	Mexico	Chiapas	Motozintla	NA	NA	NA
**Central America and Caribbean**	Mexico	Chiapas	Siltepec	NA	NA	NA
**Central America and Caribbean**	Mexico	Chiapas	Tapachula	NA	NA	NA
**Central America and Caribbean**	Mexico	Chiapas	Tuxtla Chico	NA	NA	NA
**Central America and Caribbean**	Mexico	Chiapas	Tuzantan	NA	NA	NA
**Central America and Caribbean**	Mexico	Chiapas	Tzimol	NA	NA	NA
**Central America and Caribbean**	Mexico	Chiapas	Union Juarez	NA	NA	NA
**Central America and Caribbean**	Mexico	Chiapas	Villa Comaltitlan	NA	NA	NA
**Central America and Caribbean**	Mexico	Chihuahua	NA	NA	NA	NA
**Central America and Caribbean**	Mexico	Chihuahua	Ahumada	NA	NA	NA
**Central America and Caribbean**	Mexico	Chihuahua	Aldama	NA	NA	NA
**Central America and Caribbean**	Mexico	Chihuahua	Allende	NA	NA	NA
**Central America and Caribbean**	Mexico	Chihuahua	Aquiles Serdan	NA	NA	NA
**Central America and Caribbean**	Mexico	Chihuahua	Ascension	NA	NA	NA

**Central America and Caribbean**	Mexico	Chihuahua	Bachiniva	NA	NA	NA
**Central America and Caribbean**	Mexico	Chihuahua	Bocoyna	NA	NA	NA
**Central America and Caribbean**	Mexico	Chihuahua	Buenaventura	NA	NA	NA
**Central America and Caribbean**	Mexico	Chihuahua	Camargo	NA	NA	NA
**Central America and Caribbean**	Mexico	Chihuahua	Carichi	NA	NA	NA
**Central America and Caribbean**	Mexico	Chihuahua	Casas Grandes	NA	NA	NA
**Central America and Caribbean**	Mexico	Chihuahua	Chihuahua	NA	NA	NA
**Central America and Caribbean**	Mexico	Chihuahua	Coronado	NA	NA	NA
**Central America and Caribbean**	Mexico	Chihuahua	Coyame Del Sotol	NA	NA	NA
**Central America and Caribbean**	Mexico	Chihuahua	Cuauhtemoc	NA	NA	NA
**Central America and Caribbean**	Mexico	Chihuahua	Cusihuiriachi	NA	NA	NA
**Central America and Caribbean**	Mexico	Chihuahua	Delicias	NA	NA	NA
**Central America and Caribbean**	Mexico	Chihuahua	Dr. Belisario Dominguez	NA	NA	NA
**Central America and Caribbean**	Mexico	Chihuahua	El Tule	NA	NA	NA
**Central America and Caribbean**	Mexico	Chihuahua	Galeana	NA	NA	NA

**Central America and Caribbean**	Mexico	Chihuahua	Gomez Farias	NA	NA	NA
**Central America and Caribbean**	Mexico	Chihuahua	Gran Morelos	NA	NA	NA
**Central America and Caribbean**	Mexico	Chihuahua	Guerrero	NA	NA	NA
**Central America and Caribbean**	Mexico	Chihuahua	Huejotitan	NA	NA	NA
**Central America and Caribbean**	Mexico	Chihuahua	Ignacio Zaragoza	NA	NA	NA
**Central America and Caribbean**	Mexico	Chihuahua	Janos	NA	NA	NA
**Central America and Caribbean**	Mexico	Chihuahua	Jimenez	NA	NA	NA
**Central America and Caribbean**	Mexico	Chihuahua	Julimes	NA	NA	NA
**Central America and Caribbean**	Mexico	Chihuahua	La Cruz	NA	NA	NA
**Central America and Caribbean**	Mexico	Chihuahua	Lopez	NA	NA	NA
**Central America and Caribbean**	Mexico	Chihuahua	Madera	NA	NA	NA
**Central America and Caribbean**	Mexico	Chihuahua	Maguarichi	NA	NA	NA
**Central America and Caribbean**	Mexico	Chihuahua	Manuel Benavides	NA	NA	NA
**Central America and Caribbean**	Mexico	Chihuahua	Matachi	NA	NA	NA
**Central America and Caribbean**	Mexico	Chihuahua	Matamoros	NA	NA	NA

**Central America and Caribbean**	Mexico	Chihuahua	Meoqui	NA	NA	NA
**Central America and Caribbean**	Mexico	Chihuahua	Namiquipa	NA	NA	NA
**Central America and Caribbean**	Mexico	Chihuahua	Nuevo Casas Grandes	NA	NA	NA
**Central America and Caribbean**	Mexico	Chihuahua	Ocampo	NA	NA	NA
**Central America and Caribbean**	Mexico	Chihuahua	Ojinaga	NA	NA	NA
**Central America and Caribbean**	Mexico	Chihuahua	Praxedis G. Guerrero	NA	NA	NA
**Central America and Caribbean**	Mexico	Chihuahua	Riva Palacio	NA	NA	NA
**Central America and Caribbean**	Mexico	Chihuahua	Rosales	NA	NA	NA
**Central America and Caribbean**	Mexico	Chihuahua	Rosario	NA	NA	NA
**Central America and Caribbean**	Mexico	Chihuahua	San Francisco De Borja	NA	NA	NA
**Central America and Caribbean**	Mexico	Chihuahua	San Francisco De Conchos	NA	NA	NA
**Central America and Caribbean**	Mexico	Chihuahua	Santa Isabel	NA	NA	NA
**Central America and Caribbean**	Mexico	Chihuahua	Satevo	NA	NA	NA
**Central America and Caribbean**	Mexico	Chihuahua	Saucillo	NA	NA	NA
**Central America and Caribbean**	Mexico	Chihuahua	Temosachi	NA	NA	NA

**Central America and Caribbean**	Mexico	Chihuahua	Valle De Zaragoza	NA	NA	NA
**Central America and Caribbean**	Mexico	Coahuila De Zaragoza	NA	NA	NA	NA
**Central America and Caribbean**	Mexico	Coahuila De Zaragoza	Francisco I. Madero	NA	NA	NA
**Central America and Caribbean**	Mexico	Coahuila De Zaragoza	General Cepeda	NA	NA	NA
**Central America and Caribbean**	Mexico	Coahuila De Zaragoza	Matamoros	NA	NA	NA
**Central America and Caribbean**	Mexico	Coahuila De Zaragoza	Ocampo	NA	NA	NA
**Central America and Caribbean**	Mexico	Coahuila De Zaragoza	Parras	NA	NA	NA
**Central America and Caribbean**	Mexico	Coahuila De Zaragoza	San Pedro	NA	NA	NA
**Central America and Caribbean**	Mexico	Coahuila De Zaragoza	Sierra Mojada	NA	NA	NA
**Central America and Caribbean**	Mexico	Coahuila De Zaragoza	Torreon	NA	NA	NA
**Central America and Caribbean**	Mexico	Coahuila De Zaragoza	Viesca	NA	NA	NA
**Central America and Caribbean**	Mexico	Durango	NA	NA	NA	NA
**Central America and Caribbean**	Mexico	Durango	Coneto De Comonfort	NA	NA	NA
**Central America and Caribbean**	Mexico	Durango	Cuencame	NA	NA	NA
**Central America and Caribbean**	Mexico	Durango	El Oro	NA	NA	NA

**Central America and Caribbean**	Mexico	Durango	Gomez Palacio	NA	NA	NA
**Central America and Caribbean**	Mexico	Durango	Gral. Simon Bolivar	NA	NA	NA
**Central America and Caribbean**	Mexico	Durango	Hidalgo	NA	NA	NA
**Central America and Caribbean**	Mexico	Durango	Inde	NA	NA	NA
**Central America and Caribbean**	Mexico	Durango	Lerdo	NA	NA	NA
**Central America and Caribbean**	Mexico	Durango	Mapimi	NA	NA	NA
**Central America and Caribbean**	Mexico	Durango	Nazas	NA	NA	NA
**Central America and Caribbean**	Mexico	Durango	Nuevo Ideal	NA	NA	NA
**Central America and Caribbean**	Mexico	Durango	Ocampo	NA	NA	NA
**Central America and Caribbean**	Mexico	Durango	Penon Blanco	NA	NA	NA
**Central America and Caribbean**	Mexico	Durango	Rodeo	NA	NA	NA
**Central America and Caribbean**	Mexico	Durango	San Bernardo	NA	NA	NA
**Central America and Caribbean**	Mexico	Durango	San Juan De Guadalupe	NA	NA	NA
**Central America and Caribbean**	Mexico	Durango	San Juan Del Rio	NA	NA	NA
**Central America and Caribbean**	Mexico	Durango	San Luis Del Cordero	NA	NA	NA

**Central America and Caribbean**	Mexico	Durango	San Pedro Del Gallo	NA	NA	NA
**Central America and Caribbean**	Mexico	Durango	Santa Clara	NA	NA	NA
**Central America and Caribbean**	Mexico	Durango	Santiago Papasquiaro	NA	NA	NA
**Central America and Caribbean**	Mexico	Durango	Tepehuanes	NA	NA	NA
**Central America and Caribbean**	Mexico	Durango	Tlahualilo	NA	NA	NA
**Central America and Caribbean**	Mexico	Guanajuato	NA	NA	NA	NA
**Central America and Caribbean**	Mexico	Guanajuato	Abasolo	NA	NA	NA
**Central America and Caribbean**	Mexico	Guanajuato	Cueramaro	NA	NA	NA
**Central America and Caribbean**	Mexico	Guanajuato	Huanimaro	NA	NA	NA
**Central America and Caribbean**	Mexico	Guanajuato	Irapuato	NA	NA	NA
**Central America and Caribbean**	Mexico	Guanajuato	Jaral Del Progreso	NA	NA	NA
**Central America and Caribbean**	Mexico	Guanajuato	Manuel Doblado	NA	NA	NA
**Central America and Caribbean**	Mexico	Guanajuato	Penjamo	NA	NA	NA
**Central America and Caribbean**	Mexico	Guanajuato	Pueblo Nuevo	NA	NA	NA
**Central America and Caribbean**	Mexico	Guanajuato	Romita	NA	NA	NA

**Central America and Caribbean**	Mexico	Guanajuato	Salamanca	NA	NA	NA
**Central America and Caribbean**	Mexico	Guanajuato	Silao	NA	NA	NA
**Central America and Caribbean**	Mexico	Guanajuato	Valle De Santiago	NA	NA	NA
**Central America and Caribbean**	Mexico	Guanajuato	Villagran	NA	NA	NA
**Central America and Caribbean**	Mexico	Jalisco	Chimaltitan	NA	NA	NA
**Central America and Caribbean**	Mexico	Jalisco	Encarnacion De Diaz	NA	NA	NA
**Central America and Caribbean**	Mexico	Jalisco	Jalostotitlan	NA	NA	NA
**Central America and Caribbean**	Mexico	Jalisco	Mexticacan	NA	NA	NA
**Central America and Caribbean**	Mexico	Jalisco	San Juan De Los Lagos	NA	NA	NA
**Central America and Caribbean**	Mexico	Jalisco	San Martin De Bolanos	NA	NA	NA
**Central America and Caribbean**	Mexico	Jalisco	Santa Maria De Los Angeles	NA	NA	NA
**Central America and Caribbean**	Mexico	Michoacán De Ocampo	NA	NA	NA	NA
**Central America and Caribbean**	Mexico	Michoacán De Ocampo	Angamacutiro	NA	NA	NA
**Central America and Caribbean**	Mexico	Michoacán De Ocampo	Caracuaro	NA	NA	NA
**Central America and Caribbean**	Mexico	Michoacán De Ocampo	Churintzio	NA	NA	NA

**Central America and Caribbean**	Mexico	Michoacán De Ocampo	Churumuco	NA	NA	NA
**Central America and Caribbean**	Mexico	Michoacán De Ocampo	Huetamo	NA	NA	NA
**Central America and Caribbean**	Mexico	Michoacán De Ocampo	Jimenez	NA	NA	NA
**Central America and Caribbean**	Mexico	Michoacán De Ocampo	Jose Sixto Verduzco	NA	NA	NA
**Central America and Caribbean**	Mexico	Michoacán De Ocampo	La Huacana	NA	NA	NA
**Central America and Caribbean**	Mexico	Michoacán De Ocampo	La Piedad	NA	NA	NA
**Central America and Caribbean**	Mexico	Michoacán De Ocampo	Nocupetaro	NA	NA	NA
**Central America and Caribbean**	Mexico	Michoacán De Ocampo	Numaran	NA	NA	NA
**Central America and Caribbean**	Mexico	Michoacán De Ocampo	Panindicuaro	NA	NA	NA
**Central America and Caribbean**	Mexico	Michoacán De Ocampo	Penjamillo	NA	NA	NA
**Central America and Caribbean**	Mexico	Michoacán De Ocampo	Puruandiro	NA	NA	NA
**Central America and Caribbean**	Mexico	Michoacán De Ocampo	San Lucas	NA	NA	NA
**Central America and Caribbean**	Mexico	Michoacán De Ocampo	Tiquicheo De Nicolas Romero	NA	NA	NA
**Central America and Caribbean**	Mexico	Michoacán De Ocampo	Tlazazalca	NA	NA	NA
**Central America and Caribbean**	Mexico	Michoacán De Ocampo	Zacapu	NA	NA	NA

**Central America and Caribbean**	Mexico	Michoacán De Ocampo	Zinaparo	NA	NA	NA
**Central America and Caribbean**	Mexico	México	Aculco	NA	NA	NA
**Central America and Caribbean**	Mexico	México	Atlacomulco	NA	NA	NA
**Central America and Caribbean**	Mexico	México	Chapa De Mota	NA	NA	NA
**Central America and Caribbean**	Mexico	México	Coyotepec	NA	NA	NA
**Central America and Caribbean**	Mexico	México	Huehuetoca	NA	NA	NA
**Central America and Caribbean**	Mexico	México	Jilotepec	NA	NA	NA
**Central America and Caribbean**	Mexico	México	Jocotitlan	NA	NA	NA
**Central America and Caribbean**	Mexico	México	Morelos	NA	NA	NA
**Central America and Caribbean**	Mexico	México	Polotitlan	NA	NA	NA
**Central America and Caribbean**	Mexico	México	Soyaniquilpan De Juarez	NA	NA	NA
**Central America and Caribbean**	Mexico	México	Timilpan	NA	NA	NA
**Central America and Caribbean**	Mexico	México	Villa Del Carbon	NA	NA	NA
**Central America and Caribbean**	Mexico	Nayarit	Ahuacatlan	NA	NA	NA
**Central America and Caribbean**	Mexico	Nayarit	Amatlan De Canas	NA	NA	NA

**Central America and Caribbean**	Mexico	Nayarit	Ixtlan Del Rio	NA	NA	NA
**Central America and Caribbean**	Mexico	Nayarit	Jala	NA	NA	NA
**Central America and Caribbean**	Mexico	Nayarit	La Yesca	NA	NA	NA
**Central America and Caribbean**	Mexico	Nayarit	San Pedro Lagunillas	NA	NA	NA
**Central America and Caribbean**	Mexico	Nayarit	Santa Maria Del Oro	NA	NA	NA
**Central America and Caribbean**	Mexico	Nuevo León	Aramberri	NA	NA	NA
**Central America and Caribbean**	Mexico	Nuevo León	Dr. Arroyo	NA	NA	NA
**Central America and Caribbean**	Mexico	Nuevo León	Galeana	NA	NA	NA
**Central America and Caribbean**	Mexico	Nuevo León	Gral. Zaragoza	NA	NA	NA
**Central America and Caribbean**	Mexico	Nuevo León	Hualahuises	NA	NA	NA
**Central America and Caribbean**	Mexico	Nuevo León	Iturbide	NA	NA	NA
**Central America and Caribbean**	Mexico	Nuevo León	Linares	NA	NA	NA
**Central America and Caribbean**	Mexico	Nuevo León	Mier Y Noriega	NA	NA	NA
**Central America and Caribbean**	Mexico	Nuevo León	Rayones	NA	NA	NA
**Central America and Caribbean**	Mexico	Oaxaca	Asuncion Tlacolulita	NA	NA	NA

**Central America and Caribbean**	Mexico	Puebla	Acatzingo	NA	NA	NA
**Central America and Caribbean**	Mexico	Puebla	Ahuatlan	NA	NA	NA
**Central America and Caribbean**	Mexico	Puebla	Ahuehuetitla	NA	NA	NA
**Central America and Caribbean**	Mexico	Puebla	Ajalpan	NA	NA	NA
**Central America and Caribbean**	Mexico	Puebla	Aljojuca	NA	NA	NA
**Central America and Caribbean**	Mexico	Puebla	Altepexi	NA	NA	NA
**Central America and Caribbean**	Mexico	Puebla	Atexcal	NA	NA	NA
**Central America and Caribbean**	Mexico	Puebla	Atzitzintla	NA	NA	NA
**Central America and Caribbean**	Mexico	Puebla	Axutla	NA	NA	NA
**Central America and Caribbean**	Mexico	Puebla	Canada Morelos	NA	NA	NA
**Central America and Caribbean**	Mexico	Puebla	Chalchicomula De Sesma	NA	NA	NA
**Central America and Caribbean**	Mexico	Puebla	Chapulco	NA	NA	NA
**Central America and Caribbean**	Mexico	Puebla	Chigmecatitlan	NA	NA	NA
**Central America and Caribbean**	Mexico	Puebla	Chila De La Sal	NA	NA	NA
**Central America and Caribbean**	Mexico	Puebla	Chinantla	NA	NA	NA

**Central America and Caribbean**	Mexico	Puebla	Cohetzala	NA	NA	NA
**Central America and Caribbean**	Mexico	Puebla	Coxcatlan	NA	NA	NA
**Central America and Caribbean**	Mexico	Puebla	Coyomeapan	NA	NA	NA
**Central America and Caribbean**	Mexico	Puebla	Esperanza	NA	NA	NA
**Central America and Caribbean**	Mexico	Puebla	General Felipe Angeles	NA	NA	NA
**Central America and Caribbean**	Mexico	Puebla	Guadalupe Victoria	NA	NA	NA
**Central America and Caribbean**	Mexico	Puebla	Huatlatlauca	NA	NA	NA
**Central America and Caribbean**	Mexico	Puebla	Huehuetlan El Grande	NA	NA	NA
**Central America and Caribbean**	Mexico	Puebla	Huitziltepec	NA	NA	NA
**Central America and Caribbean**	Mexico	Puebla	Juan N. Mendez	NA	NA	NA
**Central America and Caribbean**	Mexico	Puebla	La Magdalena Tlatlauquitepec	NA	NA	NA
**Central America and Caribbean**	Mexico	Puebla	Libres	NA	NA	NA
**Central America and Caribbean**	Mexico	Puebla	Mazapiltepec De Juarez	NA	NA	NA
**Central America and Caribbean**	Mexico	Puebla	Molcaxac	NA	NA	NA
**Central America and Caribbean**	Mexico	Puebla	Nicolas Bravo	NA	NA	NA

**Central America and Caribbean**	Mexico	Puebla	Oriental	NA	NA	NA
**Central America and Caribbean**	Mexico	Puebla	Palmar De Bravo	NA	NA	NA
**Central America and Caribbean**	Mexico	Puebla	Piaxtla	NA	NA	NA
**Central America and Caribbean**	Mexico	Puebla	Quecholac	NA	NA	NA
**Central America and Caribbean**	Mexico	Puebla	Rafael Lara Grajales	NA	NA	NA
**Central America and Caribbean**	Mexico	Puebla	San Jose Chiapa	NA	NA	NA
**Central America and Caribbean**	Mexico	Puebla	San Jose Miahuatlan	NA	NA	NA
**Central America and Caribbean**	Mexico	Puebla	San Juan Atenco	NA	NA	NA
**Central America and Caribbean**	Mexico	Puebla	San Juan Atzompa	NA	NA	NA
**Central America and Caribbean**	Mexico	Puebla	San Miguel Ixitlan	NA	NA	NA
**Central America and Caribbean**	Mexico	Puebla	San Nicolas Buenos Aires	NA	NA	NA
**Central America and Caribbean**	Mexico	Puebla	San Salvador El Seco	NA	NA	NA
**Central America and Caribbean**	Mexico	Puebla	San Sebastian Tlacotepec	NA	NA	NA
**Central America and Caribbean**	Mexico	Puebla	Santa Catarina Tlaltempan	NA	NA	NA
**Central America and Caribbean**	Mexico	Puebla	Santiago Miahuatlan	NA	NA	NA

**Central America and Caribbean**	Mexico	Puebla	Soltepec	NA	NA	NA
**Central America and Caribbean**	Mexico	Puebla	Tecali De Herrera	NA	NA	NA
**Central America and Caribbean**	Mexico	Puebla	Tecamachalco	NA	NA	NA
**Central America and Caribbean**	Mexico	Puebla	Tehuacan	NA	NA	NA
**Central America and Caribbean**	Mexico	Puebla	Tehuitzingo	NA	NA	NA
**Central America and Caribbean**	Mexico	Puebla	Teopantlan	NA	NA	NA
**Central America and Caribbean**	Mexico	Puebla	Tepanco De Lopez	NA	NA	NA
**Central America and Caribbean**	Mexico	Puebla	Tlachichuca	NA	NA	NA
**Central America and Caribbean**	Mexico	Puebla	Tlacotepec De Benito Juarez	NA	NA	NA
**Central America and Caribbean**	Mexico	Puebla	Tochtepec	NA	NA	NA
**Central America and Caribbean**	Mexico	Puebla	Vicente Guerrero	NA	NA	NA
**Central America and Caribbean**	Mexico	Puebla	Xochitlan Todos Santos	NA	NA	NA
**Central America and Caribbean**	Mexico	Puebla	Yehualtepec	NA	NA	NA
**Central America and Caribbean**	Mexico	Puebla	Zacapala	NA	NA	NA
**Central America and Caribbean**	Mexico	Puebla	Zapotitlan	NA	NA	NA

**Central America and Caribbean**	Mexico	Puebla	Zoquitlan	NA	NA	NA
**Central America and Caribbean**	Mexico	Querétaro Arteaga	NA	NA	NA	NA
**Central America and Caribbean**	Mexico	Querétaro Arteaga	Amealco De Bonfil	NA	NA	NA
**Central America and Caribbean**	Mexico	Querétaro Arteaga	Arroyo Seco	NA	NA	NA
**Central America and Caribbean**	Mexico	Querétaro Arteaga	Cadereyta De Montes	NA	NA	NA
**Central America and Caribbean**	Mexico	Querétaro Arteaga	Colon	NA	NA	NA
**Central America and Caribbean**	Mexico	Querétaro Arteaga	Corregidora	NA	NA	NA
**Central America and Caribbean**	Mexico	Querétaro Arteaga	El Marques	NA	NA	NA
**Central America and Caribbean**	Mexico	Querétaro Arteaga	Ezequiel Montes	NA	NA	NA
**Central America and Caribbean**	Mexico	Querétaro Arteaga	Huimilpan	NA	NA	NA
**Central America and Caribbean**	Mexico	Querétaro Arteaga	Jalpan De Serra	NA	NA	NA
**Central America and Caribbean**	Mexico	Querétaro Arteaga	Landa De Matamoros	NA	NA	NA
**Central America and Caribbean**	Mexico	Querétaro Arteaga	Pedro Escobedo	NA	NA	NA
**Central America and Caribbean**	Mexico	Querétaro Arteaga	Penamiller	NA	NA	NA
**Central America and Caribbean**	Mexico	Querétaro Arteaga	Pinal De Amoles	NA	NA	NA

**Central America and Caribbean**	Mexico	Querétaro Arteaga	Queretaro	NA	NA	NA
**Central America and Caribbean**	Mexico	Querétaro Arteaga	San Joaquin	NA	NA	NA
**Central America and Caribbean**	Mexico	Querétaro Arteaga	San Juan Del Rio	NA	NA	NA
**Central America and Caribbean**	Mexico	Querétaro Arteaga	Tequisquiapan	NA	NA	NA
**Central America and Caribbean**	Mexico	Querétaro Arteaga	Toliman	NA	NA	NA
**Central America and Caribbean**	Mexico	San Luis Potosí	NA	NA	NA	NA
**Central America and Caribbean**	Mexico	San Luis Potosí	Catorce	NA	NA	NA
**Central America and Caribbean**	Mexico	San Luis Potosí	Cedral	NA	NA	NA
**Central America and Caribbean**	Mexico	San Luis Potosí	Cerritos	NA	NA	NA
**Central America and Caribbean**	Mexico	San Luis Potosí	Charcas	NA	NA	NA
**Central America and Caribbean**	Mexico	San Luis Potosí	Guadalcazar	NA	NA	NA
**Central America and Caribbean**	Mexico	San Luis Potosí	Matehuala	NA	NA	NA
**Central America and Caribbean**	Mexico	San Luis Potosí	Moctezuma	NA	NA	NA
**Central America and Caribbean**	Mexico	San Luis Potosí	San Nicolas Tolentino	NA	NA	NA
**Central America and Caribbean**	Mexico	San Luis Potosí	Santa Maria Del Rio	NA	NA	NA

**Central America and Caribbean**	Mexico	San Luis Potosí	Santo Domingo	NA	NA	NA
**Central America and Caribbean**	Mexico	San Luis Potosí	Vanegas	NA	NA	NA
**Central America and Caribbean**	Mexico	San Luis Potosí	Villa De Arriaga	NA	NA	NA
**Central America and Caribbean**	Mexico	San Luis Potosí	Villa De Guadalupe	NA	NA	NA
**Central America and Caribbean**	Mexico	San Luis Potosí	Villa De La Paz	NA	NA	NA
**Central America and Caribbean**	Mexico	San Luis Potosí	Villa De Reyes	NA	NA	NA
**Central America and Caribbean**	Mexico	San Luis Potosí	Villa Hidalgo	NA	NA	NA
**Central America and Caribbean**	Mexico	San Luis Potosí	Villa Juarez	NA	NA	NA
**Central America and Caribbean**	Mexico	Sinaloa	Ahome	NA	NA	NA
**Central America and Caribbean**	Mexico	Sinaloa	Angostura	NA	NA	NA
**Central America and Caribbean**	Mexico	Sinaloa	Badiraguato	NA	NA	NA
**Central America and Caribbean**	Mexico	Sinaloa	Choix	NA	NA	NA
**Central America and Caribbean**	Mexico	Sinaloa	Culiacan	NA	NA	NA
**Central America and Caribbean**	Mexico	Sinaloa	El Fuerte	NA	NA	NA
**Central America and Caribbean**	Mexico	Sinaloa	Elota	NA	NA	NA

**Central America and Caribbean**	Mexico	Sinaloa	Guasave	NA	NA	NA
**Central America and Caribbean**	Mexico	Sinaloa	Mocorito	NA	NA	NA
**Central America and Caribbean**	Mexico	Sinaloa	Salvador Alvarado	NA	NA	NA
**Central America and Caribbean**	Mexico	Sinaloa	Sinaloa	NA	NA	NA
**Central America and Caribbean**	Mexico	Sonora	Agua Prieta	NA	NA	NA
**Central America and Caribbean**	Mexico	Tamaulipas	Abasolo	NA	NA	NA
**Central America and Caribbean**	Mexico	Tamaulipas	Burgos	NA	NA	NA
**Central America and Caribbean**	Mexico	Tamaulipas	Bustamante	NA	NA	NA
**Central America and Caribbean**	Mexico	Tamaulipas	Casas	NA	NA	NA
**Central America and Caribbean**	Mexico	Tamaulipas	Cruillas	NA	NA	NA
**Central America and Caribbean**	Mexico	Tamaulipas	Guemez	NA	NA	NA
**Central America and Caribbean**	Mexico	Tamaulipas	Hidalgo	NA	NA	NA
**Central America and Caribbean**	Mexico	Tamaulipas	Jaumave	NA	NA	NA
**Central America and Caribbean**	Mexico	Tamaulipas	Mainero	NA	NA	NA
**Central America and Caribbean**	Mexico	Tamaulipas	Miquihuana	NA	NA	NA

**Central America and Caribbean**	Mexico	Tamaulipas	Padilla	NA	NA	NA
**Central America and Caribbean**	Mexico	Tamaulipas	San Carlos	NA	NA	NA
**Central America and Caribbean**	Mexico	Tamaulipas	San Fernando	NA	NA	NA
**Central America and Caribbean**	Mexico	Tamaulipas	San Nicolas	NA	NA	NA
**Central America and Caribbean**	Mexico	Tamaulipas	Soto La Marina	NA	NA	NA
**Central America and Caribbean**	Mexico	Tamaulipas	Victoria	NA	NA	NA
**Central America and Caribbean**	Mexico	Tamaulipas	Villagran	NA	NA	NA
**Central America and Caribbean**	Mexico	Veracruz De Ignacio De La Llave	Acultzingo	NA	NA	NA
**Central America and Caribbean**	Mexico	Veracruz De Ignacio De La Llave	Cordoba	NA	NA	NA
**Central America and Caribbean**	Mexico	Veracruz De Ignacio De La Llave	Rio Blanco	NA	NA	NA
**Central America and Caribbean**	Mexico	Veracruz De Ignacio De La Llave	Tequila	NA	NA	NA
**Central America and Caribbean**	Mexico	Yucatán	Cantamayec	NA	NA	NA
**Central America and Caribbean**	Mexico	Yucatán	Chankom	NA	NA	NA

**Central America and Caribbean**	Mexico	Yucatán	Kaua	NA	NA	NA
**Central America and Caribbean**	Mexico	Yucatán	Tekax	NA	NA	NA
**Central America and Caribbean**	Mexico	Yucatán	Yaxcaba	NA	NA	NA
**Central America and Caribbean**	Mexico	Zacatecas	NA	NA	NA	NA
**Central America and Caribbean**	Mexico	Zacatecas	Apozol	NA	NA	NA
**Central America and Caribbean**	Mexico	Zacatecas	Apulco	NA	NA	NA
**Central America and Caribbean**	Mexico	Zacatecas	Atolinga	NA	NA	NA
**Central America and Caribbean**	Mexico	Zacatecas	Benito Juarez	NA	NA	NA
**Central America and Caribbean**	Mexico	Zacatecas	Calera	NA	NA	NA
**Central America and Caribbean**	Mexico	Zacatecas	Canitas De Felipe Pescador	NA	NA	NA
**Central America and Caribbean**	Mexico	Zacatecas	Concepcion Del Oro	NA	NA	NA
**Central America and Caribbean**	Mexico	Zacatecas	Cuauhtemoc	NA	NA	NA
**Central America and Caribbean**	Mexico	Zacatecas	El Plateado De Joaquin Amaro	NA	NA	NA
**Central America and Caribbean**	Mexico	Zacatecas	El Salvador	NA	NA	NA
**Central America and Caribbean**	Mexico	Zacatecas	Genaro Codina	NA	NA	NA

**Central America and Caribbean**	Mexico	Zacatecas	General Enrique Estrada	NA	NA	NA
**Central America and Caribbean**	Mexico	Zacatecas	General Francisco R. Murguia	NA	NA	NA
**Central America and Caribbean**	Mexico	Zacatecas	Huanusco	NA	NA	NA
**Central America and Caribbean**	Mexico	Zacatecas	Jalpa	NA	NA	NA
**Central America and Caribbean**	Mexico	Zacatecas	Juan Aldama	NA	NA	NA
**Central America and Caribbean**	Mexico	Zacatecas	Luis Moya	NA	NA	NA
**Central America and Caribbean**	Mexico	Zacatecas	Mazapil	NA	NA	NA
**Central America and Caribbean**	Mexico	Zacatecas	Melchor Ocampo	NA	NA	NA
**Central America and Caribbean**	Mexico	Zacatecas	Mezquital Del Oro	NA	NA	NA
**Central America and Caribbean**	Mexico	Zacatecas	Miguel Auza	NA	NA	NA
**Central America and Caribbean**	Mexico	Zacatecas	Momax	NA	NA	NA
**Central America and Caribbean**	Mexico	Zacatecas	Monte Escobedo	NA	NA	NA
**Central America and Caribbean**	Mexico	Zacatecas	Moyahua De Estrada	NA	NA	NA
**Central America and Caribbean**	Mexico	Zacatecas	Nochistlan De Mejia	NA	NA	NA
**Central America and Caribbean**	Mexico	Zacatecas	Pinos	NA	NA	NA

**Central America and Caribbean**	Mexico	Zacatecas	Rio Grande	NA	NA	NA
**Central America and Caribbean**	Mexico	Zacatecas	Sain Alto	NA	NA	NA
**Central America and Caribbean**	Mexico	Zacatecas	Tabasco	NA	NA	NA
**Central America and Caribbean**	Mexico	Zacatecas	Tepechitlan	NA	NA	NA
**Central America and Caribbean**	Mexico	Zacatecas	Teul De Gonzalez Ortega	NA	NA	NA
**Central America and Caribbean**	Mexico	Zacatecas	Villa De Cos	NA	NA	NA
**Central America and Caribbean**	Mexico	Zacatecas	Villa Garcia	NA	NA	NA
**Central Asia**	Afghanistan	Badakhshan	Wakhan	Afghanistan	Badakhshan	Eshkmesh
**NA**	NA	NA	NA	Afghanistan	Badakhshan	Shighnan
**NA**	NA	NA	NA	Afghanistan	Badakhshan	Shuhada
**NA**	NA	NA	NA	Afghanistan	Badakhshan	Wakhan
**NA**	NA	NA	NA	Afghanistan	Nuristan	Barg-e- Matal
**NA**	NA	NA	NA	Afghanistan	Nuristan	Kamdesh
**NA**	NA	NA	NA	Tajikistan	Gorno-Badakhshan	Ishkoshim
**East Asia**	China	NA	NA	China	NA	NA
**East Asia**	China	Anhui	Chuzhou	China	Anhui	NA
**East Asia**	China	Fujian	NA	China	Chongqing	NA
**East Asia**	China	Fujian	Longyan	China	Chongqing	Chongqing
**East Asia**	China	Fujian	Quanzhou	China	Fujian	NA
**East Asia**	China	Fujian	Xiamen	China	Gansu	NA
**East Asia**	China	Gansu	Jiuquan	China	Gansu	Jiuquan
**East Asia**	China	Guangdong	NA	China	Guangdong	NA

**East Asia**	China	Guangdong	Maoming	China	Guangdong	Zhanjiang
**East Asia**	China	Guangdong	Yangjiang	China	Guangxi	NA
**East Asia**	China	Guangdong	Zhanjiang	China	Guangxi	Beihai
**East Asia**	China	Guangxi	Beihai	China	Guangxi	Fangchenggang
**East Asia**	China	Henan	NA	China	Guizhou	NA
**East Asia**	China	Jiangsu	Huai'an	China	Henan	NA
**East Asia**	China	Jiangsu	Lianyungang	China	Hunan	NA
**East Asia**	China	Jiangsu	Nanjing	China	Jiangsu	Huai'an
**East Asia**	China	Jiangsu	Suqian	China	Jiangsu	Lianyungang
**East Asia**	China	Jiangsu	Yangzhou	China	Jiangsu	Suqian
**East Asia**	China	Jiangxi	NA	China	Jiangsu	Xuzhou
**East Asia**	China	Jiangxi	Ganzhou	China	Jiangxi	NA
**East Asia**	China	Jiangxi	Yingtan	China	Jiangxi	Ganzhou
**East Asia**	China	Nei Mongol	NA	China	Jiangxi	Shangrao
**East Asia**	China	Nei Mongol	Alxa	China	Nei Mongol	NA
**East Asia**	China	Nei Mongol	Baotou	China	Nei Mongol	Alxa
**East Asia**	China	Nei Mongol	Hulunbuir	China	Nei Mongol	Baotou
**East Asia**	China	Nei Mongol	Tongliao	China	Nei Mongol	Hohhot
**East Asia**	China	Nei Mongol	Wuhai	China	Nei Mongol	Hulunbuir
**East Asia**	China	Nei Mongol	Xilin Gol	China	Nei Mongol	Tongliao
**East Asia**	China	Nei Mongol	Xing'an	China	Nei Mongol	Wuhai
**East Asia**	China	Ningxia Hui	NA	China	Nei Mongol	Xilin Gol
**East Asia**	China	Ningxia Hui	Shizuishan	China	Nei Mongol	Xing'an
**East Asia**	China	Ningxia Hui	Yinchuan	China	Ningxia Hui	NA
**East Asia**	China	Qinghai	Gyêgu Tibetan	China	Qinghai	Golog Tibetan
**East Asia**	China	Qinghai	Haixi Mongol and Tibetan	China	Qinghai	Gyêgu Tibetan
**East Asia**	China	Shandong	NA	China	Qinghai	Haixi Mongol and Tibetan

**East Asia**	China	Shandong	Linyi	China	Shandong	NA
**East Asia**	China	Shandong	Qingdao	China	Shanxi	NA
**East Asia**	China	Shandong	Rizhao	China	Sichuan	NA
**East Asia**	China	Shandong	Weihai	China	Sichuan	Garzê Tibetan
**East Asia**	China	Shandong	Yantai	China	Sichuan	Liangshan Yi
**East Asia**	China	Sichuan	Garzê Tibetan	China	Sichuan	Ngawa Tibetan and Qiang
**East Asia**	China	Sichuan	Panzhihua	China	Sichuan	Panzhihua
**East Asia**	China	Xinjiang	NA	China	Xinjiang	NA
**East Asia**	China	Xinjiang	Khotan	China	Xinjiang	Khotan
**East Asia**	China	Xizang	Chamdo	China	Xizang	Chamdo
**East Asia**	China	Xizang	Ngari	China	Xizang	Ngari
**East Asia**	China	Yunnan	NA	China	Yunnan	NA
**East Asia**	China	Yunnan	Chuxiong Yi	China	Yunnan	Baoshan
**East Asia**	China	Yunnan	Honghe Hani and Yi	China	Yunnan	Dali Bai
**East Asia**	China	Yunnan	Kunming	China	Yunnan	Dehong Dai and Jingpo
**East Asia**	China	Yunnan	Xishuangbanna Dai	China	Yunnan	Honghe Hani and Yi
**East Asia**	China	Zhejiang	Lishui	China	Yunnan	Kunming
**East Asia**	China	Zhejiang	Quzhou	China	Yunnan	Lijiang
**East Asia**	China	Zhejiang	Wenzhou	China	Yunnan	Lincang
**East Asia**	China	Zhejiang	Zhoushan	China	Yunnan	Nujiang Lisu
**NA**	NA	NA	NA	China	Yunnan	Pu'er
**NA**	NA	NA	NA	China	Yunnan	Xishuangbanna Dai
**NA**	NA	NA	NA	China	Zhejiang	Lishui
**NA**	NA	NA	NA	China	Zhejiang	Quzhou
**NA**	NA	NA	NA	China	Zhejiang	Wenzhou

**NA**	NA	NA	NA	China	Zhejiang	Zhoushan
**Eastern sub-Saharan Africa**	Burundi	Bujumbura Rural	Mugongomanga	Burundi	Bujumbura Rural	Mugongomanga
**Eastern sub-Saharan Africa**	Burundi	Bujumbura Rural	Mukike	Burundi	Bujumbura Rural	Mukike
**Eastern sub-Saharan Africa**	Burundi	Bururi	Bururi	Burundi	Bururi	Matana
**Eastern sub-Saharan Africa**	Burundi	Bururi	Matana	Burundi	Bururi	Mugamba
**Eastern sub-Saharan Africa**	Burundi	Bururi	Mugamba	Burundi	Bururi	Songa
**Eastern sub-Saharan Africa**	Burundi	Bururi	Rutovu	Burundi	Mwaro	Bisoro
**Eastern sub-Saharan Africa**	Burundi	Bururi	Songa	Burundi	Mwaro	Gisozi
**Eastern sub-Saharan Africa**	Burundi	Gitega	Ryansoro	Burundi	Mwaro	Rusaka
**Eastern sub-Saharan Africa**	Burundi	Muramvya	Muramvya	Ethiopia	Amhara	NA
**Eastern sub-Saharan Africa**	Burundi	Mwaro	NA	Ethiopia	Amhara	Debub Gondar
**Eastern sub-Saharan Africa**	Burundi	Mwaro	Bisoro	Ethiopia	Amhara	Mirab Gojjam
**Eastern sub-Saharan Africa**	Burundi	Mwaro	Gisozi	Ethiopia	Amhara	Misraq Gojjam
**Eastern sub-Saharan Africa**	Burundi	Mwaro	Kayokwe	Ethiopia	Gambela Peoples	Majang
**Eastern sub-Saharan Africa**	Burundi	Mwaro	Rusaka	Ethiopia	Oromia	North Shewa
**Eastern sub-Saharan Africa**	Ethiopia	Gambela Peoples	Majang	Ethiopia	Southern Nations,	Bench Maji

					Nationalities and Peoples	
**Eastern sub-Saharan Africa**	Ethiopia	Oromia	North Shewa	Ethiopia	Southern Nations, Nationalities and Peoples	Dawro
**Eastern sub-Saharan Africa**	Ethiopia	Southern Nations, Nationalities and Peoples	Keffa	Ethiopia	Southern Nations, Nationalities and Peoples	Keffa
**Eastern sub-Saharan Africa**	Kenya	Bungoma	Kimilili	Ethiopia	Southern Nations, Nationalities and Peoples	Konta
**Eastern sub-Saharan Africa**	Kenya	Bungoma	Mt. Elgon	Ethiopia	Southern Nations, Nationalities and Peoples	Sheka
**Eastern sub-Saharan Africa**	Kenya	Bungoma	Tongaren	Ethiopia	Tigray	Mehakelegnaw
**Eastern sub-Saharan Africa**	Kenya	Kericho	Bureti	Kenya	Bungoma	NA
**Eastern sub-Saharan Africa**	Kenya	Kericho	Kipkelion East	Kenya	Bungoma	Kabuchai
**Eastern sub-Saharan Africa**	Kenya	Nakuru	Kuresoi North	Kenya	Bungoma	Kimilili
**Eastern sub-Saharan Africa**	Kenya	Nakuru	Kuresoi South	Kenya	Bungoma	Lugari
**Eastern sub-Saharan Africa**	Kenya	Nakuru	Molo	Kenya	Bungoma	Mt. Elgon

**Eastern sub-Saharan Africa**	Kenya	Nyandarua	Ol Jorok	Kenya	Bungoma	Sirisia
**Eastern sub-Saharan Africa**	Kenya	Trans Nzoia	NA	Kenya	Bungoma	Tongaren
**Eastern sub-Saharan Africa**	Kenya	Trans Nzoia	Endebess	Kenya	Busia	Teso North
**Eastern sub-Saharan Africa**	Kenya	Trans Nzoia	Kiminini	Kenya	Trans Nzoia	NA
**Eastern sub-Saharan Africa**	Kenya	Trans Nzoia	Saboti	Kenya	Trans Nzoia	Endebess
**Eastern sub-Saharan Africa**	Kenya	Trans Nzoia	unknown 4	Kenya	Trans Nzoia	Kwanza
**Eastern sub-Saharan Africa**	Kenya	West Pokot	Pokot South	Kenya	Trans Nzoia	Saboti
**Eastern sub-Saharan Africa**	Rwanda	Amajyaruguru	NA	Kenya	Trans Nzoia	unknown 4
**Eastern sub-Saharan Africa**	Rwanda	Amajyaruguru	Burera	Kenya	West Pokot	Kapenguria
**Eastern sub-Saharan Africa**	Rwanda	Amajyaruguru	Gakenke	Kenya	West Pokot	Pokot South
**Eastern sub-Saharan Africa**	Rwanda	Amajyaruguru	Gicumbi	Rwanda	Amajyaruguru	NA
**Eastern sub-Saharan Africa**	Rwanda	Amajyaruguru	Musanze	Rwanda	Amajyaruguru	Burera
**Eastern sub-Saharan Africa**	Rwanda	Amajyaruguru	Rulindo	Rwanda	Amajyaruguru	Gakenke
**Eastern sub-Saharan Africa**	Rwanda	Amajyepfo	Muhanga	Rwanda	Amajyaruguru	Gicumbi
**Eastern sub-Saharan Africa**	Rwanda	Iburengerazuba	NA	Rwanda	Amajyaruguru	Musanze

**Eastern sub-Saharan Africa**	Rwanda	Iburengerazuba	Ngororero	Rwanda	Amajyaruguru	Rulindo
**Eastern sub-Saharan Africa**	Rwanda	Iburengerazuba	Nyabihu	Rwanda	Amajyepfo	Kamonyi
**Eastern sub-Saharan Africa**	Rwanda	Iburengerazuba	Rubavu	Rwanda	Amajyepfo	Muhanga
**Eastern sub-Saharan Africa**	Rwanda	Iburengerazuba	Rutsiro	Rwanda	Iburengerazuba	NA
**Eastern sub-Saharan Africa**	Uganda	Kabale	NA	Rwanda	Iburengerazuba	Karongi
**Eastern sub-Saharan Africa**	Uganda	Kabale	Kabale Municipality	Rwanda	Iburengerazuba	Ngororero
**Eastern sub-Saharan Africa**	Uganda	Kabale	Ndorwa	Rwanda	Iburengerazuba	Nyabihu
**Eastern sub-Saharan Africa**	Uganda	Kisoro	NA	Rwanda	Iburengerazuba	Rubavu
**Eastern sub-Saharan Africa**	Uganda	Kisoro	Bufumbira	Rwanda	Iburengerazuba	Rutsiro
**Eastern sub-Saharan Africa**	Uganda	Kisoro	Kisoro Municipality	Rwanda	Umujyi wa Kigali	NA
**Eastern sub-Saharan Africa**	Uganda	Rubanda	NA	Rwanda	Umujyi wa Kigali	Gasabo
**Eastern sub-Saharan Africa**	Uganda	Rubanda	Rubanda	Tanzania	Dodoma	Chamwino
**Eastern sub-Saharan Africa**	Uganda	Rukiga	NA	Tanzania	Mbeya	Mbeya Rural
**Eastern sub-Saharan Africa**	Uganda	Rukiga	Rukiga	Tanzania	Singida	Singida Rural
**NA**	NA	NA	NA	Uganda	Kabale	NA
**NA**	NA	NA	NA	Uganda	Kabale	Kabale Municipality

**NA**	NA	NA	NA	Uganda	Kabale	Ndorwa
**NA**	NA	NA	NA	Uganda	Kisoro	NA
**NA**	NA	NA	NA	Uganda	Kisoro	Bufumbira
**NA**	NA	NA	NA	Uganda	Kisoro	Kisoro Municipality
**NA**	NA	NA	NA	Uganda	Mbarara	NA
**NA**	NA	NA	NA	Uganda	Mbarara	Rwampara
**NA**	NA	NA	NA	Uganda	Ntungamo	NA
**NA**	NA	NA	NA	Uganda	Ntungamo	Kajara
**NA**	NA	NA	NA	Uganda	Ntungamo	Ruhaama
**NA**	NA	NA	NA	Uganda	Ntungamo	Rushenyi
**NA**	NA	NA	NA	Uganda	Rubanda	NA
**NA**	NA	NA	NA	Uganda	Rubanda	Rubanda
**NA**	NA	NA	NA	Uganda	Rukiga	NA
**NA**	NA	NA	NA	Uganda	Rukiga	Rukiga
**NA**	NA	NA	NA	Uganda	Rukungiri	NA
**NA**	NA	NA	NA	Uganda	Rukungiri	Rubabo
**Middle East**	Iran	NA	NA	Iran	NA	NA
**Middle East**	Iran	Alborz	NA	Iran	Alborz	NA
**Middle East**	Iran	Alborz	Karaj	Iran	Alborz	Karaj
**Middle East**	Iran	Alborz	Nazarabad	Iran	Alborz	Nazarabad
**Middle East**	Iran	Alborz	Savojbalagh	Iran	Alborz	Savojbalagh
**Middle East**	Iran	Alborz	Taleghan	Iran	Alborz	Taleghan
**Middle East**	Iran	Ardebil	NA	Iran	Ardebil	NA
**Middle East**	Iran	Ardebil	Ardebil	Iran	Ardebil	Ardebil
**Middle East**	Iran	Ardebil	Bilehsavar	Iran	Ardebil	Meshkinshah
**Middle East**	Iran	Ardebil	Garmi	Iran	Ardebil	Namin
**Middle East**	Iran	Ardebil	Khalkhal	Iran	Ardebil	Nayer
**Middle East**	Iran	Ardebil	Kowsar	Iran	Ardebil	Sareyn

**Middle East**	Iran	Ardebil	Meshkinshah	Iran	Bushehr	NA
**Middle East**	Iran	Ardebil	Namin	Iran	Bushehr	Bushehr
**Middle East**	Iran	Ardebil	Nayer	Iran	Bushehr	Dashtestan
**Middle East**	Iran	Ardebil	Sareyn	Iran	Bushehr	Dayyer
**Middle East**	Iran	Bushehr	NA	Iran	Bushehr	Deylam
**Middle East**	Iran	Bushehr	Bushehr	Iran	Bushehr	Genaveh
**Middle East**	Iran	Bushehr	Dashtestan	Iran	Bushehr	Tangestan
**Middle East**	Iran	Bushehr	Dashti	Iran	Chaharmahal & Bakhtiyari	NA
**Middle East**	Iran	Bushehr	Dayyer	Iran	Chaharmahal & Bakhtiyari	Ardal
**Middle East**	Iran	Bushehr	Deylam	Iran	Chaharmahal & Bakhtiyari	Borujen
**Middle East**	Iran	Bushehr	Genaveh	Iran	Chaharmahal & Bakhtiyari	Lordegan
**Middle East**	Iran	Bushehr	Tangestan	Iran	Chaharmahal & Bakhtiyari	Shahr-e-Kord
**Middle East**	Iran	Chaharmahal & Bakhtiyari	NA	Iran	East Azarbayejan	NA
**Middle East**	Iran	Chaharmahal & Bakhtiyari	Ardal	Iran	East Azarbayejan	Ahar
**Middle East**	Iran	Chaharmahal & Bakhtiyari	Borujen	Iran	East Azarbayejan	Ajabshir
**Middle East**	Iran	Chaharmahal & Bakhtiyari	Farsan	Iran	East Azarbayejan	Azarshahr
**Middle East**	Iran	Chaharmahal & Bakhtiyari	Keyar	Iran	East Azarbayejan	Bonab
**Middle East**	Iran	Chaharmahal & Bakhtiyari	Kuhrang	Iran	East Azarbayejan	Bostanabad
**Middle East**	Iran	Chaharmahal & Bakhtiyari	Lordegan	Iran	East Azarbayejan	Charoimaq

**Middle East**	Iran	Chaharmahal & Bakhtiyari	Shahr-e-Kord	Iran	East Azarbayejan	Haris
**Middle East**	Iran	East Azarbayejan	NA	Iran	East Azarbayejan	Hashtrud
**Middle East**	Iran	East Azarbayejan	Ahar	Iran	East Azarbayejan	Jolfa
**Middle East**	Iran	East Azarbayejan	Ajabshir	Iran	East Azarbayejan	Kaleibar
**Middle East**	Iran	East Azarbayejan	Azarshahr	Iran	East Azarbayejan	Malekan
**Middle East**	Iran	East Azarbayejan	Bonab	Iran	East Azarbayejan	Maragheh
**Middle East**	Iran	East Azarbayejan	Bostanabad	Iran	East Azarbayejan	Marand
**Middle East**	Iran	East Azarbayejan	Charoimaq	Iran	East Azarbayejan	Miyaneh
**Middle East**	Iran	East Azarbayejan	Haris	Iran	East Azarbayejan	Osku
**Middle East**	Iran	East Azarbayejan	Hashtrud	Iran	East Azarbayejan	Sarab
**Middle East**	Iran	East Azarbayejan	Jolfa	Iran	East Azarbayejan	Shabestar
**Middle East**	Iran	East Azarbayejan	Kaleibar	Iran	East Azarbayejan	Tabriz
**Middle East**	Iran	East Azarbayejan	Malekan	Iran	Esfahan	NA
**Middle East**	Iran	East Azarbayejan	Maragheh	Iran	Esfahan	Dehaghan
**Middle East**	Iran	East Azarbayejan	Marand	Iran	Esfahan	Falavarjan

**Middle East**	Iran	East Azarbayejan	Miyaneh	Iran	Esfahan	Isfahan
**Middle East**	Iran	East Azarbayejan	Osku	Iran	Esfahan	Kashan
**Middle East**	Iran	East Azarbayejan	Sarab	Iran	Esfahan	Mobarakeh
**Middle East**	Iran	East Azarbayejan	Shabestar	Iran	Esfahan	Shahreza
**Middle East**	Iran	East Azarbayejan	Tabriz	Iran	Fars	Farashband
**Middle East**	Iran	Esfahan	NA	Iran	Fars	Gerash
**Middle East**	Iran	Esfahan	Aran & Bidgol	Iran	Fars	Kazerun
**Middle East**	Iran	Esfahan	Dehaghan	Iran	Fars	Khonj
**Middle East**	Iran	Esfahan	Falavarjan	Iran	Fars	Mamasani
**Middle East**	Iran	Esfahan	Golpayegan	Iran	Fars	Marvdasht
**Middle East**	Iran	Esfahan	Isfahan	Iran	Fars	Mohr
**Middle East**	Iran	Esfahan	Kashan	Iran	Fars	Rostam
**Middle East**	Iran	Esfahan	Khour& Biyabanak	Iran	Gilan	NA
**Middle East**	Iran	Esfahan	Lanjan	Iran	Gilan	Amlash
**Middle East**	Iran	Esfahan	Mobarakeh	Iran	Gilan	Astaneh-ye-Ashrafiyeh
**Middle East**	Iran	Esfahan	Najafabad	Iran	Gilan	Bandar-e-Anzali
**Middle East**	Iran	Esfahan	Shahreza	Iran	Gilan	Fuman
**Middle East**	Iran	Fars	Abadeh	Iran	Gilan	Masal
**Middle East**	Iran	Fars	Eqlid	Iran	Gilan	Rasht
**Middle East**	Iran	Fars	Farashband	Iran	Gilan	Rezvanshahr
**Middle East**	Iran	Fars	Gerash	Iran	Gilan	Rudbar
**Middle East**	Iran	Fars	Kazerun	Iran	Gilan	Rudsar
**Middle East**	Iran	Fars	Khonj	Iran	Gilan	Shaft
**Middle East**	Iran	Fars	Lamard	Iran	Gilan	Siahkal

**Middle East**	Iran	Fars	Mamasani	Iran	Gilan	Sume'eh Sara
**Middle East**	Iran	Fars	Marvdasht	Iran	Gilan	Tavalesh
**Middle East**	Iran	Fars	Mohr	Iran	Golestan	Aliabad
**Middle East**	Iran	Fars	Rostam	Iran	Golestan	Azadshahr
**Middle East**	Iran	Fars	Sepidan	Iran	Golestan	Gorgan
**Middle East**	Iran	Gilan	NA	Iran	Golestan	Ramyan
**Middle East**	Iran	Gilan	Amlash	Iran	Hamedan	NA
**Middle East**	Iran	Gilan	Astaneh-ye-Ashrafiyeh	Iran	Hamedan	Bahar
**Middle East**	Iran	Gilan	Astara	Iran	Hamedan	Famenin
**Middle East**	Iran	Gilan	Bandar-e-Anzali	Iran	Hamedan	Hamedan
**Middle East**	Iran	Gilan	Fuman	Iran	Hamedan	Kabudarahang
**Middle East**	Iran	Gilan	Lahijan	Iran	Hamedan	Nahavand
**Middle East**	Iran	Gilan	Langrud	Iran	Hamedan	Tuyserkan
**Middle East**	Iran	Gilan	Masal	Iran	Hormozgan	NA
**Middle East**	Iran	Gilan	Rasht	Iran	Hormozgan	Bandar Abbas
**Middle East**	Iran	Gilan	Rezvanshahr	Iran	Hormozgan	Bandar Lengeh
**Middle East**	Iran	Gilan	Rudbar	Iran	Hormozgan	Bastak
**Middle East**	Iran	Gilan	Rudsar	Iran	Hormozgan	Khamir
**Middle East**	Iran	Gilan	Shaft	Iran	Hormozgan	Qeshm
**Middle East**	Iran	Gilan	Siahkal	Iran	Hormozgan	Rudan
**Middle East**	Iran	Gilan	Sume'eh Sara	Iran	Ilam	NA
**Middle East**	Iran	Gilan	Tavalesh	Iran	Ilam	Abdanan
**Middle East**	Iran	Golestan	NA	Iran	Ilam	Chardavel
**Middle East**	Iran	Golestan	Aliabad	Iran	Ilam	Dehloran
**Middle East**	Iran	Golestan	Azadshahr	Iran	Ilam	Ilam
**Middle East**	Iran	Golestan	Galikesh	Iran	Ilam	Ivan
**Middle East**	Iran	Golestan	Gorgan	Iran	Ilam	Malekshahi
**Middle East**	Iran	Golestan	Kalaleh	Iran	Kerman	Bam
**Middle East**	Iran	Golestan	Kordkuy	Iran	Kerman	Ghaleye-Ganj

**Middle East**	Iran	Golestan	Minudasht	Iran	Kerman	Kahnuj
**Middle East**	Iran	Golestan	Ramyan	Iran	Kerman	Manujan
**Middle East**	Iran	Golestan	Torkaman	Iran	Kermanshah	NA
**Middle East**	Iran	Hamedan	NA	Iran	Kermanshah	Javanrud
**Middle East**	Iran	Hamedan	Asadabad	Iran	Kermanshah	Kangavar
**Middle East**	Iran	Hamedan	Bahar	Iran	Kermanshah	Kermanshah
**Middle East**	Iran	Hamedan	Famenin	Iran	Kermanshah	Paveh
**Middle East**	Iran	Hamedan	Hamedan	Iran	Kermanshah	Ravansar
**Middle East**	Iran	Hamedan	Kabudarahang	Iran	Kermanshah	Salas-e-Babajani
**Middle East**	Iran	Hamedan	Malayer	Iran	Kermanshah	Sonqor
**Middle East**	Iran	Hamedan	Nahavand	Iran	Khorasan-e-Razavi	NA
**Middle East**	Iran	Hamedan	Razan	Iran	Khorasan-e-Razavi	Bajestan
**Middle East**	Iran	Hamedan	Tuyserkan	Iran	Khorasan-e-Razavi	Bakhraz
**Middle East**	Iran	Hormozgan	NA	Iran	Khorasan-e-Razavi	Bardeskan
**Middle East**	Iran	Hormozgan	Bandar Abbas	Iran	Khorasan-e-Razavi	Chenaran
**Middle East**	Iran	Hormozgan	Bastak	Iran	Khorasan-e-Razavi	Firooze
**Middle East**	Iran	Hormozgan	Khamir	Iran	Khorasan-e-Razavi	Gonabad
**Middle East**	Iran	Hormozgan	Qeshm	Iran	Khorasan-e-Razavi	Kashmar
**Middle East**	Iran	Hormozgan	Rudan	Iran	Khorasan-e-Razavi	Khaf
**Middle East**	Iran	Ilam	NA	Iran	Khorasan-e-Razavi	Khalil Abad

**Middle East**	Iran	Ilam	Abdanan	Iran	Khorasan-e-Razavi	Mahvelat
**Middle East**	Iran	Ilam	Chardavel	Iran	Khorasan-e-Razavi	Mashhad
**Middle East**	Iran	Ilam	Darrehshahr	Iran	Khorasan-e-Razavi	Neyshabur
**Middle East**	Iran	Ilam	Dehloran	Iran	Khorasan-e-Razavi	Rashtkhar
**Middle East**	Iran	Ilam	Ilam	Iran	Khorasan-e-Razavi	Taybad
**Middle East**	Iran	Ilam	Ivan	Iran	Khorasan-e-Razavi	Torbat-e-Jam
**Middle East**	Iran	Ilam	Malekshahi	Iran	Khuzestan	NA
**Middle East**	Iran	Ilam	Mehran	Iran	Khuzestan	Abadan
**Middle East**	Iran	Kerman	NA	Iran	Khuzestan	Ahvaz
**Middle East**	Iran	Kerman	Anbarabad	Iran	Khuzestan	Baghemalek
**Middle East**	Iran	Kerman	Bam	Iran	Khuzestan	Bandar-e-Mahshahr
**Middle East**	Iran	Kerman	Ghaleye-Ganj	Iran	Khuzestan	Behbahan
**Middle East**	Iran	Kerman	Jiroft	Iran	Khuzestan	Haftgol
**Middle East**	Iran	Kerman	Kahnuj	Iran	Khuzestan	Hendijan
**Middle East**	Iran	Kerman	Kerman	Iran	Khuzestan	Hoveizeh
**Middle East**	Iran	Kerman	Kohbonan	Iran	Khuzestan	Masjed Soleyman
**Middle East**	Iran	Kerman	Manujan	Iran	Khuzestan	Omidiyeh
**Middle East**	Iran	Kerman	Ravar	Iran	Khuzestan	Ramhormoz
**Middle East**	Iran	Kermanshah	NA	Iran	Khuzestan	Ramshir
**Middle East**	Iran	Kermanshah	Javanrud	Iran	Khuzestan	Shadegan
**Middle East**	Iran	Kermanshah	Kangavar	Iran	Kohgiluyeh & Boyerahmad	NA

**Middle East**	Iran	Kermanshah	Kermanshah	Iran	Kohgiluyeh & Boyerahmad	Bahmaee
**Middle East**	Iran	Kermanshah	Paveh	Iran	Kohgiluyeh & Boyerahmad	Cheram
**Middle East**	Iran	Kermanshah	Ravansar	Iran	Kohgiluyeh & Boyerahmad	Gachsaran
**Middle East**	Iran	Kermanshah	Sonqor	Iran	Kohgiluyeh & Boyerahmad	Kohgiluyeh
**Middle East**	Iran	Khorasan-e-Razavi	NA	Iran	Kordestan	NA
**Middle East**	Iran	Khorasan-e-Razavi	Bajestan	Iran	Kordestan	Bijar
**Middle East**	Iran	Khorasan-e-Razavi	Bardeskan	Iran	Kordestan	Dehgalan
**Middle East**	Iran	Khorasan-e-Razavi	Binalood	Iran	Kordestan	Divandarreh
**Middle East**	Iran	Khorasan-e-Razavi	Chenaran	Iran	Kordestan	Kamyaran
**Middle East**	Iran	Khorasan-e-Razavi	Darrehgaz	Iran	Kordestan	Marivan
**Middle East**	Iran	Khorasan-e-Razavi	Firooze	Iran	Kordestan	Qorveh
**Middle East**	Iran	Khorasan-e-Razavi	Gonabad	Iran	Kordestan	Sanandaj
**Middle East**	Iran	Khorasan-e-Razavi	Joghatay	Iran	Kordestan	Saqqez
**Middle East**	Iran	Khorasan-e-Razavi	Kashmar	Iran	Kordestan	Sarvabad
**Middle East**	Iran	Khorasan-e-Razavi	Khaf	Iran	Lorestan	NA

**Middle East**	Iran	Khorasan-e-Razavi	Khalil Abad	Iran	Lorestan	Aligudarz
**Middle East**	Iran	Khorasan-e-Razavi	Mahvelat	Iran	Lorestan	Azna
**Middle East**	Iran	Khorasan-e-Razavi	Mashhad	Iran	Lorestan	Borujerd
**Middle East**	Iran	Khorasan-e-Razavi	Neyshabur	Iran	Lorestan	Delfan
**Middle East**	Iran	Khorasan-e-Razavi	Quchan	Iran	Lorestan	Doreh
**Middle East**	Iran	Khorasan-e-Razavi	Rashtkhar	Iran	Lorestan	Dorud
**Middle East**	Iran	Khorasan-e-Razavi	Sabzevar	Iran	Lorestan	Khorramabad
**Middle East**	Iran	Khorasan-e-Razavi	Taybad	Iran	Lorestan	Poldokhtar
**Middle East**	Iran	Khorasan-e-Razavi	Torbat-e-Jam	Iran	Lorestan	Selseleh
**Middle East**	Iran	Khuzestan	NA	Iran	Markazi	NA
**Middle East**	Iran	Khuzestan	Abadan	Iran	Markazi	Delijan
**Middle East**	Iran	Khuzestan	Ahvaz	Iran	Markazi	Khondab
**Middle East**	Iran	Khuzestan	Baghemalek	Iran	Markazi	Komeijan
**Middle East**	Iran	Khuzestan	Bandar-e-Mahshahr	Iran	Markazi	Mahalat
**Middle East**	Iran	Khuzestan	Behbahan	Iran	Markazi	Saveh
**Middle East**	Iran	Khuzestan	Haftgol	Iran	Markazi	Shazand
**Middle East**	Iran	Khuzestan	Hendijan	Iran	Markazi	Tafresh
**Middle East**	Iran	Khuzestan	Hoveizeh	Iran	Markazi	Zarandiyeh
**Middle East**	Iran	Khuzestan	Khorramshahr	Iran	Mazandaran	NA
**Middle East**	Iran	Khuzestan	Masjed Soleyman	Iran	Mazandaran	Amol
**Middle East**	Iran	Khuzestan	Omidiyeh	Iran	Mazandaran	Babol

**Middle East**	Iran	Khuzestan	Ramhormoz	Iran	Mazandaran	Behshahr
**Middle East**	Iran	Khuzestan	Ramshir	Iran	Mazandaran	Chalus
**Middle East**	Iran	Khuzestan	Shadegan	Iran	Mazandaran	Galugah
**Middle East**	Iran	Kohgiluyeh & Boyerahmad	NA	Iran	Mazandaran	Neka
**Middle East**	Iran	Kohgiluyeh & Boyerahmad	Bahmaee	Iran	Mazandaran	Noshahr
**Middle East**	Iran	Kohgiluyeh & Boyerahmad	Cheram	Iran	Mazandaran	Nur
**Middle East**	Iran	Kohgiluyeh & Boyerahmad	Dena	Iran	Mazandaran	Qaemshahr
**Middle East**	Iran	Kohgiluyeh & Boyerahmad	Gachsaran	Iran	Mazandaran	Ramsar
**Middle East**	Iran	Kohgiluyeh & Boyerahmad	Kohgiluyeh	Iran	Mazandaran	Sari
**Middle East**	Iran	Kordestan	NA	Iran	Mazandaran	Tonekabon
**Middle East**	Iran	Kordestan	Baneh	Iran	North Khorasan	Garmeh
**Middle East**	Iran	Kordestan	Bijar	Iran	North Khorasan	Jajarm
**Middle East**	Iran	Kordestan	Dehgalan	Iran	Qazvin	NA
**Middle East**	Iran	Kordestan	Divandarreh	Iran	Qazvin	Abyek
**Middle East**	Iran	Kordestan	Kamyaran	Iran	Qazvin	Alborz
**Middle East**	Iran	Kordestan	Marivan	Iran	Qazvin	Boyinzahra
**Middle East**	Iran	Kordestan	Qorveh	Iran	Qazvin	Qazvin
**Middle East**	Iran	Kordestan	Sanandaj	Iran	Qom	NA
**Middle East**	Iran	Kordestan	Saqqez	Iran	Qom	Qom
**Middle East**	Iran	Kordestan	Sarvabad	Iran	Semnan	NA
**Middle East**	Iran	Lorestan	NA	Iran	Semnan	Damghan
**Middle East**	Iran	Lorestan	Aligudarz	Iran	Semnan	Garmsar
**Middle East**	Iran	Lorestan	Azna	Iran	Semnan	Mehdishahr
**Middle East**	Iran	Lorestan	Borujerd	Iran	Semnan	Semnan

**Middle East**	Iran	Lorestan	Delfan	Iran	Semnan	Shahrud
**Middle East**	Iran	Lorestan	Doreh	Iran	Sistan & Baluchestan	Chabahar
**Middle East**	Iran	Lorestan	Dorud	Iran	Sistan & Baluchestan	Konarak
**Middle East**	Iran	Lorestan	Khorramabad	Iran	South Khorasan	NA
**Middle East**	Iran	Lorestan	Kuhdasht	Iran	South Khorasan	Birjand
**Middle East**	Iran	Lorestan	Poldokhtar	Iran	South Khorasan	Darmiyan
**Middle East**	Iran	Lorestan	Selseleh	Iran	South Khorasan	Qayenat
**Middle East**	Iran	Markazi	NA	Iran	South Khorasan	Sarbisheh
**Middle East**	Iran	Markazi	Arak	Iran	Tehran	NA
**Middle East**	Iran	Markazi	Ashtiyan	Iran	Tehran	Eslamshahr
**Middle East**	Iran	Markazi	Delijan	Iran	Tehran	Malard
**Middle East**	Iran	Markazi	Farahan	Iran	Tehran	Pakdasht
**Middle East**	Iran	Markazi	Khomeyn	Iran	Tehran	Robatkarim
**Middle East**	Iran	Markazi	Khondab	Iran	Tehran	Shahriar
**Middle East**	Iran	Markazi	Komeijan	Iran	Tehran	Shemiranat
**Middle East**	Iran	Markazi	Mahalat	Iran	Tehran	Tehran
**Middle East**	Iran	Markazi	Saveh	Iran	West Azarbayejan	NA
**Middle East**	Iran	Markazi	Shazand	Iran	West Azarbayejan	Bukan
**Middle East**	Iran	Markazi	Tafresh	Iran	West Azarbayejan	Chaipareh
**Middle East**	Iran	Markazi	Zarandiyeh	Iran	West Azarbayejan	Chaldoran
**Middle East**	Iran	Mazandaran	NA	Iran	West Azarbayejan	Khoy
**Middle East**	Iran	Mazandaran	Abbas abad	Iran	West Azarbayejan	Mahabad

**Middle East**	Iran	Mazandaran	Amol	Iran	West Azarbayejan	Maku
**Middle East**	Iran	Mazandaran	Babol	Iran	West Azarbayejan	Miyandoab
**Middle East**	Iran	Mazandaran	Babolsar	Iran	West Azarbayejan	Naqadeh
**Middle East**	Iran	Mazandaran	Behshahr	Iran	West Azarbayejan	Orumiyeh
**Middle East**	Iran	Mazandaran	Chalus	Iran	West Azarbayejan	Piranshahr
**Middle East**	Iran	Mazandaran	Fereydunkenar	Iran	West Azarbayejan	Poldasht
**Middle East**	Iran	Mazandaran	Galugah	Iran	West Azarbayejan	Salmas
**Middle East**	Iran	Mazandaran	Juybar	Iran	West Azarbayejan	Shahindezh
**Middle East**	Iran	Mazandaran	Mahmoudabad	Iran	West Azarbayejan	Showt
**Middle East**	Iran	Mazandaran	Neka	Iran	Yazd	NA
**Middle East**	Iran	Mazandaran	Noshahr	Iran	Yazd	Ardakan
**Middle East**	Iran	Mazandaran	Nur	Iran	Yazd	Ashkzar
**Middle East**	Iran	Mazandaran	Qaemshahr	Iran	Yazd	Behabad
**Middle East**	Iran	Mazandaran	Ramsar	Iran	Yazd	Meybod
**Middle East**	Iran	Mazandaran	Sari	Iran	Yazd	Taft
**Middle East**	Iran	Mazandaran	Savadkuh	Iran	Yazd	Yazd
**Middle East**	Iran	Mazandaran	Tonekabon	Iran	Zanjan	NA
**Middle East**	Iran	North Khorasan	Faruj	Iran	Zanjan	Abhar
**Middle East**	Iran	Qazvin	NA	Iran	Zanjan	Ijerud
**Middle East**	Iran	Qazvin	Abyek	Iran	Zanjan	Khodabandeh
**Middle East**	Iran	Qazvin	Alborz	Iran	Zanjan	Khorramdarreh

**Middle East**	Iran	Qazvin	Boyinzahra	Iran	Zanjan	Mahneshan
**Middle East**	Iran	Qazvin	Qazvin	Iran	Zanjan	Tarom
**Middle East**	Iran	Qazvin	Takestan	Iran	Zanjan	Zanjan
**Middle East**	Iran	Qom	NA	NA	NA	NA
**Middle East**	Iran	Qom	Qom	NA	NA	NA
**Middle East**	Iran	Semnan	NA	NA	NA	NA
**Middle East**	Iran	Semnan	Damghan	NA	NA	NA
**Middle East**	Iran	Semnan	Garmsar	NA	NA	NA
**Middle East**	Iran	Semnan	Mehdishahr	NA	NA	NA
**Middle East**	Iran	Semnan	Semnan	NA	NA	NA
**Middle East**	Iran	Semnan	Shahrud	NA	NA	NA
**Middle East**	Iran	Sistan & Baluchestan	Chabahar	NA	NA	NA
**Middle East**	Iran	Sistan & Baluchestan	Zabol	NA	NA	NA
**Middle East**	Iran	South Khorasan	NA	NA	NA	NA
**Middle East**	Iran	South Khorasan	Birjand	NA	NA	NA
**Middle East**	Iran	South Khorasan	Darmiyan	NA	NA	NA
**Middle East**	Iran	South Khorasan	Nahbandan	NA	NA	NA
**Middle East**	Iran	South Khorasan	Qayenat	NA	NA	NA
**Middle East**	Iran	South Khorasan	Sarbisheh	NA	NA	NA
**Middle East**	Iran	Tehran	NA	NA	NA	NA
**Middle East**	Iran	Tehran	Damavand	NA	NA	NA
**Middle East**	Iran	Tehran	Eslamshahr	NA	NA	NA
**Middle East**	Iran	Tehran	Firuzkuh	NA	NA	NA
**Middle East**	Iran	Tehran	Malard	NA	NA	NA
**Middle East**	Iran	Tehran	Pakdasht	NA	NA	NA
**Middle East**	Iran	Tehran	Qods	NA	NA	NA
**Middle East**	Iran	Tehran	Robatkarim	NA	NA	NA

**Middle East**	Iran	Tehran	Shahriar	NA	NA	NA
**Middle East**	Iran	Tehran	Shemiranat	NA	NA	NA
**Middle East**	Iran	Tehran	Tehran	NA	NA	NA
**Middle East**	Iran	Tehran	Varamin	NA	NA	NA
**Middle East**	Iran	West Azarbayejan	NA	NA	NA	NA
**Middle East**	Iran	West Azarbayejan	Bukan	NA	NA	NA
**Middle East**	Iran	West Azarbayejan	Chaipareh	NA	NA	NA
**Middle East**	Iran	West Azarbayejan	Chaldoran	NA	NA	NA
**Middle East**	Iran	West Azarbayejan	Khoy	NA	NA	NA
**Middle East**	Iran	West Azarbayejan	Mahabad	NA	NA	NA
**Middle East**	Iran	West Azarbayejan	Maku	NA	NA	NA
**Middle East**	Iran	West Azarbayejan	Miyandoab	NA	NA	NA
**Middle East**	Iran	West Azarbayejan	Naqadeh	NA	NA	NA
**Middle East**	Iran	West Azarbayejan	Orumiyeh	NA	NA	NA
**Middle East**	Iran	West Azarbayejan	Oshnaviyeh	NA	NA	NA
**Middle East**	Iran	West Azarbayejan	Piranshahr	NA	NA	NA
**Middle East**	Iran	West Azarbayejan	Poldasht	NA	NA	NA

**Middle East**	Iran	West Azarbayejan	Salmas	NA	NA	NA
**Middle East**	Iran	West Azarbayejan	Sardasht	NA	NA	NA
**Middle East**	Iran	West Azarbayejan	Shahindezh	NA	NA	NA
**Middle East**	Iran	West Azarbayejan	Showt	NA	NA	NA
**Middle East**	Iran	West Azarbayejan	Takab	NA	NA	NA
**Middle East**	Iran	Yazd	NA	NA	NA	NA
**Middle East**	Iran	Yazd	Abarkuh	NA	NA	NA
**Middle East**	Iran	Yazd	Ardakan	NA	NA	NA
**Middle East**	Iran	Yazd	Ashkzar	NA	NA	NA
**Middle East**	Iran	Yazd	Behabad	NA	NA	NA
**Middle East**	Iran	Yazd	Mehriz	NA	NA	NA
**Middle East**	Iran	Yazd	Meybod	NA	NA	NA
**Middle East**	Iran	Yazd	Taft	NA	NA	NA
**Middle East**	Iran	Yazd	Yazd	NA	NA	NA
**Middle East**	Iran	Zanjan	NA	NA	NA	NA
**Middle East**	Iran	Zanjan	Abhar	NA	NA	NA
**Middle East**	Iran	Zanjan	Ijerud	NA	NA	NA
**Middle East**	Iran	Zanjan	Khodabandeh	NA	NA	NA
**Middle East**	Iran	Zanjan	Khorramdarreh	NA	NA	NA
**Middle East**	Iran	Zanjan	Mahneshan	NA	NA	NA
**Middle East**	Iran	Zanjan	Tarom	NA	NA	NA
**Middle East**	Iran	Zanjan	Zanjan	NA	NA	NA
**Oceania**	Timor-Leste	Ainaro	Hato Builico	Indonesia	Nusa Tenggara Timur	Alor

**Oceania**	Timor-Leste	Ainaro	Maubisse	Indonesia	Nusa Tenggara Timur	Belu
**Oceania**	Timor-Leste	Manufahi	Fato Berliu	Timor-Leste	Ainaro	Hato Builico
**Oceania**	Timor-Leste	Manufahi	Turiscai	Timor-Leste	Ainaro	Maubisse
**NA**	NA	NA	NA	Timor-Leste	Lautém	Tutuala
**NA**	NA	NA	NA	Timor-Leste	Manatuto	Laclubar
**NA**	NA	NA	NA	Timor-Leste	Manatuto	Laleia
**NA**	NA	NA	NA	Timor-Leste	Manatuto	Soibada
**NA**	NA	NA	NA	Timor-Leste	Manufahi	NA
**NA**	NA	NA	NA	Timor-Leste	Manufahi	Alas
**NA**	NA	NA	NA	Timor-Leste	Manufahi	Fato Berliu
**NA**	NA	NA	NA	Timor-Leste	Manufahi	Same
**NA**	NA	NA	NA	Timor-Leste	Manufahi	Turiscai
**NA**	NA	NA	NA	Timor-Leste	Viqueque	Lacluta
**NA**	NA	NA	NA	Timor-Leste	Viqueque	Uato Carbau
**NA**	NA	NA	NA	Timor-Leste	Viqueque	Viqueque
**NA**	NA	NA	NA	India	Mizoram	Champhai
**Southeast Asia**	Thailand	Bangkok Metropolis	NA	Thailand	NA	NA
**Southeast Asia**	Thailand	Bangkok Metropolis	Bang Kapi	Thailand	Bangkok Metropolis	NA
**Southeast Asia**	Thailand	Bangkok Metropolis	Bang Khae	Thailand	Bangkok Metropolis	Bang Khae
**Southeast Asia**	Thailand	Bangkok Metropolis	Bang Na	Thailand	Bangkok Metropolis	Bang Na
**Southeast Asia**	Thailand	Bangkok Metropolis	Chatuchak	Thailand	Bangkok Metropolis	Chatuchak
**Southeast Asia**	Thailand	Bangkok Metropolis	Din Dang	Thailand	Bangkok Metropolis	Huai Kwang

**Southeast Asia**	Thailand	Bangkok Metropolis	Huai Kwang	Thailand	Bangkok Metropolis	Phaya Thai
**Southeast Asia**	Thailand	Bangkok Metropolis	Khlong Toey	Thailand	Bangkok Metropolis	Wattana
**Southeast Asia**	Thailand	Bangkok Metropolis	Nongkheam	Thailand	Buri Ram	Non Din Daeng
**Southeast Asia**	Thailand	Bangkok Metropolis	Phaya Thai	Thailand	Chachoengsao	NA
**Southeast Asia**	Thailand	Bangkok Metropolis	Suan Luang	Thailand	Chachoengsao	Ban Pho
**Southeast Asia**	Thailand	Bangkok Metropolis	Wang Thonglang	Thailand	Chachoengsao	Muang Chachoengsao
**Southeast Asia**	Thailand	Bangkok Metropolis	Wattana	Thailand	Chachoengsao	Sanam Chaikhet
**Southeast Asia**	Thailand	Chanthaburi	NA	Thailand	Chachoengsao	Tha Ta Kieb
**Southeast Asia**	Thailand	Chanthaburi	K. Kao Kichakut	Thailand	Chanthaburi	NA
**Southeast Asia**	Thailand	Chanthaburi	K. Na Yai Am	Thailand	Chanthaburi	K. Kao Kichakut
**Southeast Asia**	Thailand	Chanthaburi	Kang Hang Maeo	Thailand	Chanthaburi	K. Na Yai Am
**Southeast Asia**	Thailand	Chanthaburi	Khlung	Thailand	Chanthaburi	Kang Hang Maeo
**Southeast Asia**	Thailand	Chanthaburi	Makham	Thailand	Chanthaburi	Khlung
**Southeast Asia**	Thailand	Chanthaburi	Muang Chanthaburi	Thailand	Chanthaburi	Makham
**Southeast Asia**	Thailand	Chanthaburi	Pong Nam Ron	Thailand	Chanthaburi	Muang Chanthaburi
**Southeast Asia**	Thailand	Chanthaburi	Tha Mai	Thailand	Chanthaburi	Pong Nam Ron
**Southeast Asia**	Thailand	Chiang Mai	NA	Thailand	Chanthaburi	Tha Mai
**Southeast Asia**	Thailand	Chiang Mai	Chiang Dao	Thailand	Chiang Mai	NA
**Southeast Asia**	Thailand	Chiang Mai	Chom Thong	Thailand	Chiang Mai	Chiang Dao
**Southeast Asia**	Thailand	Chiang Mai	Doi Saket	Thailand	Chiang Mai	Chom Thong
**Southeast Asia**	Thailand	Chiang Mai	Doi Tao	Thailand	Chiang Mai	Doi Saket
**Southeast Asia**	Thailand	Chiang Mai	Fang	Thailand	Chiang Mai	Doi Tao

**Southeast Asia**	Thailand	Chiang Mai	Hang Dong	Thailand	Chiang Mai	Fang
**Southeast Asia**	Thailand	Chiang Mai	Hot	Thailand	Chiang Mai	Hang Dong
**Southeast Asia**	Thailand	Chiang Mai	K. Doi Lo	Thailand	Chiang Mai	Hot
**Southeast Asia**	Thailand	Chiang Mai	K. Mae On	Thailand	Chiang Mai	K. Doi Lo
**Southeast Asia**	Thailand	Chiang Mai	Mae Ai	Thailand	Chiang Mai	K. Mae On
**Southeast Asia**	Thailand	Chiang Mai	Mae Chaem	Thailand	Chiang Mai	Mae Ai
**Southeast Asia**	Thailand	Chiang Mai	Mae Rim	Thailand	Chiang Mai	Mae Chaem
**Southeast Asia**	Thailand	Chiang Mai	Mae Taeng	Thailand	Chiang Mai	Mae Rim
**Southeast Asia**	Thailand	Chiang Mai	Mae Wang	Thailand	Chiang Mai	Mae Taeng
**Southeast Asia**	Thailand	Chiang Mai	Muang Chiang Mai	Thailand	Chiang Mai	Mae Wang
**Southeast Asia**	Thailand	Chiang Mai	Omkoi	Thailand	Chiang Mai	Muang Chiang Mai
**Southeast Asia**	Thailand	Chiang Mai	Phrao	Thailand	Chiang Mai	Omkoi
**Southeast Asia**	Thailand	Chiang Mai	Samoeng	Thailand	Chiang Mai	Phrao
**Southeast Asia**	Thailand	Chiang Mai	San Kamphaeng	Thailand	Chiang Mai	Samoeng
**Southeast Asia**	Thailand	Chiang Mai	San Pa Tong	Thailand	Chiang Mai	San Kamphaeng
**Southeast Asia**	Thailand	Chiang Mai	San Sai	Thailand	Chiang Mai	San Pa Tong
**Southeast Asia**	Thailand	Chiang Mai	Saraphi	Thailand	Chiang Mai	San Sai
**Southeast Asia**	Thailand	Chiang Mai	Wiang Haeng	Thailand	Chiang Mai	Saraphi
**Southeast Asia**	Thailand	Chiang Rai	Khun Tan	Thailand	Chiang Mai	Wiang Haeng
**Southeast Asia**	Thailand	Chiang Rai	Mae Suai	Thailand	Chiang Rai	Khun Tan
**Southeast Asia**	Thailand	Chiang Rai	Pa Daet	Thailand	Chiang Rai	Mae Suai
**Southeast Asia**	Thailand	Chiang Rai	Phan	Thailand	Chiang Rai	Phan
**Southeast Asia**	Thailand	Chiang Rai	Thoeng	Thailand	Chiang Rai	Thoeng
**Southeast Asia**	Thailand	Chiang Rai	Wiang Pa Pao	Thailand	Chiang Rai	Wiang Pa Pao
**Southeast Asia**	Thailand	Chon Buri	Ban Bung	Thailand	Chon Buri	NA
**Southeast Asia**	Thailand	Chon Buri	Bo Thong	Thailand	Chon Buri	Ban Bung
**Southeast Asia**	Thailand	Chon Buri	Nong Yai	Thailand	Chon Buri	Bo Thong
**Southeast Asia**	Thailand	Chon Buri	Phanat Nikhom	Thailand	Chon Buri	K. Ko Chan

**Southeast Asia**	Thailand	Lampang	NA	Thailand	Chon Buri	Muang Chon Buri
**Southeast Asia**	Thailand	Lampang	Chae Hom	Thailand	Chon Buri	Nong Yai
**Southeast Asia**	Thailand	Lampang	Hang Chat	Thailand	Chon Buri	Phan Thong
**Southeast Asia**	Thailand	Lampang	Ko Kha	Thailand	Chon Buri	Phanat Nikhom
**Southeast Asia**	Thailand	Lampang	Mae Mo	Thailand	Lampang	NA
**Southeast Asia**	Thailand	Lampang	Mae Phrik	Thailand	Lampang	Chae Hom
**Southeast Asia**	Thailand	Lampang	Mae Tha	Thailand	Lampang	Hang Chat
**Southeast Asia**	Thailand	Lampang	Muang Lampang	Thailand	Lampang	Ko Kha
**Southeast Asia**	Thailand	Lampang	Mueang Pan	Thailand	Lampang	Mae Mo
**Southeast Asia**	Thailand	Lampang	Ngao	Thailand	Lampang	Mae Phrik
**Southeast Asia**	Thailand	Lampang	Soem Ngam	Thailand	Lampang	Mae Tha
**Southeast Asia**	Thailand	Lampang	Sop Prap	Thailand	Lampang	Muang Lampang
**Southeast Asia**	Thailand	Lampang	Wang Nua	Thailand	Lampang	Mueang Pan
**Southeast Asia**	Thailand	Lamphun	NA	Thailand	Lampang	Ngao
**Southeast Asia**	Thailand	Lamphun	Ban Hong	Thailand	Lampang	Soem Ngam
**Southeast Asia**	Thailand	Lamphun	K. Wiang Nong Long	Thailand	Lampang	Sop Prap
**Southeast Asia**	Thailand	Lamphun	Li	Thailand	Lampang	Thoen
**Southeast Asia**	Thailand	Lamphun	Mae Tha	Thailand	Lampang	Wang Nua
**Southeast Asia**	Thailand	Lamphun	Pa Sang	Thailand	Lamphun	NA
**Southeast Asia**	Thailand	Lamphun	Thung Hua Chang	Thailand	Lamphun	Ban Hong
**Southeast Asia**	Thailand	Loei	Dan Sai	Thailand	Lamphun	Ban Thi
**Southeast Asia**	Thailand	Loei	Na Haeo	Thailand	Lamphun	K. Wiang Nong Long
**Southeast Asia**	Thailand	Loei	Phu Rua	Thailand	Lamphun	Li
**Southeast Asia**	Thailand	Loei	Tha Li	Thailand	Lamphun	Mae Tha
**Southeast Asia**	Thailand	Mae Hong Son	NA	Thailand	Lamphun	Muang Lamphun
**Southeast Asia**	Thailand	Mae Hong Son	Khun Yuam	Thailand	Lamphun	Pa Sang
**Southeast Asia**	Thailand	Mae Hong Son	Mae La Noi	Thailand	Lamphun	Thung Hua Chang
**Southeast Asia**	Thailand	Mae Hong Son	Mae Sariang	Thailand	Loei	NA

**Southeast Asia**	Thailand	Mae Hong Son	Muang Mae Hong Son	Thailand	Loei	Dan Sai
**Southeast Asia**	Thailand	Mae Hong Son	Pai	Thailand	Loei	Muang Loei
**Southeast Asia**	Thailand	Mae Hong Son	Pang Ma Pha	Thailand	Loei	Na Haeo
**Southeast Asia**	Thailand	Mae Hong Son	Sop Moei	Thailand	Loei	Phu Rua
**Southeast Asia**	Thailand	Nakhon Pathom	Sam Phran	Thailand	Loei	Tha Li
**Southeast Asia**	Thailand	Nan	NA	Thailand	Mae Hong Son	NA
**Southeast Asia**	Thailand	Nan	Ban Luang	Thailand	Mae Hong Son	Khun Yuam
**Southeast Asia**	Thailand	Nan	Bo Klue	Thailand	Mae Hong Son	Mae La Noi
**Southeast Asia**	Thailand	Nan	Chalermphrakiet	Thailand	Mae Hong Son	Mae Sariang
**Southeast Asia**	Thailand	Nan	Chiang Klang	Thailand	Mae Hong Son	Muang Mae Hong Son
**Southeast Asia**	Thailand	Nan	K. Phu Pieng	Thailand	Mae Hong Son	Pai
**Southeast Asia**	Thailand	Nan	Mae Charim	Thailand	Mae Hong Son	Pang Ma Pha
**Southeast Asia**	Thailand	Nan	Muang Nan	Thailand	Mae Hong Son	Sop Moei
**Southeast Asia**	Thailand	Nan	Na Mun	Thailand	Nakhon Pathom	Sam Phran
**Southeast Asia**	Thailand	Nan	Na Noi	Thailand	Nan	NA
**Southeast Asia**	Thailand	Nan	Pua	Thailand	Nan	Ban Luang
**Southeast Asia**	Thailand	Nan	Santi Suk	Thailand	Nan	Bo Klue
**Southeast Asia**	Thailand	Nan	Song Kwae	Thailand	Nan	Chalermphrakiet
**Southeast Asia**	Thailand	Nan	Tha Wang Pha	Thailand	Nan	Chiang Klang
**Southeast Asia**	Thailand	Nan	Thung Chang	Thailand	Nan	K. Phu Pieng
**Southeast Asia**	Thailand	Nan	Wiang Sa	Thailand	Nan	Mae Charim
**Southeast Asia**	Thailand	Phayao	NA	Thailand	Nan	Muang Nan
**Southeast Asia**	Thailand	Phayao	Chiang Kham	Thailand	Nan	Na Mun
**Southeast Asia**	Thailand	Phayao	Chiang Muan	Thailand	Nan	Na Noi
**Southeast Asia**	Thailand	Phayao	Chun	Thailand	Nan	Pua
**Southeast Asia**	Thailand	Phayao	Dok Kham Tai	Thailand	Nan	Santi Suk
**Southeast Asia**	Thailand	Phayao	K. Phu Kam Yao	Thailand	Nan	Song Kwae

**Southeast Asia**	Thailand	Phayao	K. Phu Sang	Thailand	Nan	Tha Wang Pha
**Southeast Asia**	Thailand	Phayao	Mae Chai	Thailand	Nan	Thung Chang
**Southeast Asia**	Thailand	Phayao	Muang Phayao	Thailand	Nan	Wiang Sa
**Southeast Asia**	Thailand	Phayao	Pong	Thailand	Phayao	NA
**Southeast Asia**	Thailand	Phetchabun	Lom Kao	Thailand	Phayao	Chiang Kham
**Southeast Asia**	Thailand	Phitsanulok	NA	Thailand	Phayao	Chiang Muan
**Southeast Asia**	Thailand	Phitsanulok	Chat Trakan	Thailand	Phayao	Chun
**Southeast Asia**	Thailand	Phitsanulok	Muang Phitsanulok	Thailand	Phayao	Dok Kham Tai
**Southeast Asia**	Thailand	Phitsanulok	Nakhon Thai	Thailand	Phayao	K. Phu Kam Yao
**Southeast Asia**	Thailand	Phitsanulok	Wang Thong	Thailand	Phayao	K. Phu Sang
**Southeast Asia**	Thailand	Phra Nakhon Si Ayutthaya	Phra Nakhon Si Ayutthaya	Thailand	Phayao	Mae Chai
**Southeast Asia**	Thailand	Phra Nakhon Si Ayutthaya	Uthai	Thailand	Phayao	Muang Phayao
**Southeast Asia**	Thailand	Phra Nakhon Si Ayutthaya	Wang Noi	Thailand	Phayao	Pong
**Southeast Asia**	Thailand	Phrae	NA	Thailand	Phetchabun	Lom Kao
**Southeast Asia**	Thailand	Phrae	Den Chai	Thailand	Phetchabun	Wang Pong
**Southeast Asia**	Thailand	Phrae	Long	Thailand	Phichit	K. Sak Lek
**Southeast Asia**	Thailand	Phrae	Muang Phrae	Thailand	Phichit	Wachira Barami
**Southeast Asia**	Thailand	Phrae	Nong Muang Kai	Thailand	Phichit	Wang Sai Phun
**Southeast Asia**	Thailand	Phrae	Rong Kwang	Thailand	Phitsanulok	NA
**Southeast Asia**	Thailand	Phrae	Song	Thailand	Phitsanulok	Bang Krathum
**Southeast Asia**	Thailand	Phrae	Sung Men	Thailand	Phitsanulok	Chat Trakan
**Southeast Asia**	Thailand	Rayong	NA	Thailand	Phitsanulok	Muang Phitsanulok
**Southeast Asia**	Thailand	Rayong	Ban Khai	Thailand	Phitsanulok	Nakhon Thai
**Southeast Asia**	Thailand	Rayong	K. Khao Chamao	Thailand	Phitsanulok	Noen Maprang
**Southeast Asia**	Thailand	Rayong	K. Nikhom Pattan	Thailand	Phitsanulok	Phrom Phiram

**Southeast Asia**	Thailand	Rayong	Klaeng	Thailand	Phitsanulok	Wang Thong
**Southeast Asia**	Thailand	Rayong	Muang Rayong	Thailand	Phitsanulok	Wat Bot
**Southeast Asia**	Thailand	Rayong	Pluak Daeng	Thailand	Phrae	NA
**Southeast Asia**	Thailand	Rayong	Wang Chan	Thailand	Phrae	Den Chai
**Southeast Asia**	Thailand	Sa Kaeo	NA	Thailand	Phrae	Long
**Southeast Asia**	Thailand	Sa Kaeo	K. Kok Sung	Thailand	Phrae	Muang Phrae
**Southeast Asia**	Thailand	Sa Kaeo	K. Wang Sombun	Thailand	Phrae	Nong Muang Kai
**Southeast Asia**	Thailand	Sa Kaeo	Wang Nam Yen	Thailand	Phrae	Rong Kwang
**Southeast Asia**	Thailand	Sa Kaeo	Watthana Nakhon	Thailand	Phrae	Song
**Southeast Asia**	Thailand	Sing Buri	Muang Sing Buri	Thailand	Phrae	Sung Men
**Southeast Asia**	Thailand	Trat	NA	Thailand	Prachin Buri	Kabin Buri
**Southeast Asia**	Thailand	Trat	Bo Rai	Thailand	Rayong	NA
**Southeast Asia**	Thailand	Trat	Laem Ngop	Thailand	Rayong	Ban Chang
**Southeast Asia**	Thailand	Uttaradit	NA	Thailand	Rayong	Ban Khai
**Southeast Asia**	Thailand	Uttaradit	Ban Khok	Thailand	Rayong	K. Khao Chamao
**Southeast Asia**	Thailand	Uttaradit	Fak Tha	Thailand	Rayong	K. Nikhom Pattan
**Southeast Asia**	Thailand	Uttaradit	Muang Uttaradit	Thailand	Rayong	Klaeng
**Southeast Asia**	Thailand	Uttaradit	Nam Pat	Thailand	Rayong	Muang Rayong
**Southeast Asia**	Thailand	Uttaradit	Tha Pla	Thailand	Rayong	Wang Chan
**Southeast Asia**	Thailand	Uttaradit	Thong Saen Khan	Thailand	Sa Kaeo	NA
**Southeast Asia**	Vietnam	An Giang	NA	Thailand	Sa Kaeo	Aranyaprathet
**Southeast Asia**	Vietnam	An Giang	Châu Phú	Thailand	Sa Kaeo	K. Kok Sung
**Southeast Asia**	Vietnam	An Giang	Châu Thành	Thailand	Sa Kaeo	K. Wang Sombun
**Southeast Asia**	Vietnam	An Giang	Chợ Mới	Thailand	Sa Kaeo	Kao Cha Kan
**Southeast Asia**	Vietnam	An Giang	Long Xuyên	Thailand	Sa Kaeo	Khlong Hat
**Southeast Asia**	Vietnam	An Giang	Phú Tân	Thailand	Sa Kaeo	Muang Sa Kaeo
**Southeast Asia**	Vietnam	An Giang	Thoại Sơn	Thailand	Sa Kaeo	Ta Phraya
**Southeast Asia**	Vietnam	An Giang	Tân Châu	Thailand	Sa Kaeo	Wang Nam Yen

**Southeast Asia**	Vietnam	Bắc Giang	Hiệp Hòa	Thailand	Sa Kaeo	Watthana Nakhon
**Southeast Asia**	Vietnam	Bắc Giang	Tân Yên	Thailand	Samut Sakhon	Krathum Baen
**Southeast Asia**	Vietnam	Bắc Giang	Việt Yên	Thailand	Tak	Mae Ramat
**Southeast Asia**	Vietnam	Bắc Giang	Yên Dũng	Thailand	Tak	Sam Ngao
**Southeast Asia**	Vietnam	Bắc Giang	Yên Thế	Thailand	Tak	Tha Song Yang
**Southeast Asia**	Vietnam	Bắc Ninh	NA	Thailand	Trat	NA
**Southeast Asia**	Vietnam	Bắc Ninh	Bắc Ninh	Thailand	Trat	Bo Rai
**Southeast Asia**	Vietnam	Bắc Ninh	Gia Bình	Thailand	Trat	Khao Saming
**Southeast Asia**	Vietnam	Bắc Ninh	Lương Tài	Thailand	Trat	Laem Ngop
**Southeast Asia**	Vietnam	Bắc Ninh	Quế Võ	Thailand	Uttaradit	NA
**Southeast Asia**	Vietnam	Bắc Ninh	Thuận Thành	Thailand	Uttaradit	Ban Khok
**Southeast Asia**	Vietnam	Bắc Ninh	Tiên Du	Thailand	Uttaradit	Fak Tha
**Southeast Asia**	Vietnam	Bắc Ninh	Từ Sơn	Thailand	Uttaradit	Muang Uttaradit
**Southeast Asia**	Vietnam	Bắc Ninh	Yên Phong	Thailand	Uttaradit	Nam Pat
**Southeast Asia**	Vietnam	Bến Tre	Ba Tri	Thailand	Uttaradit	Tha Pla
**Southeast Asia**	Vietnam	Bến Tre	Giồng Trôm	Thailand	Uttaradit	Thong Saen Khan
**Southeast Asia**	Vietnam	Bến Tre	Mỏ Cày Bắc	Vietnam	An Giang	NA
**Southeast Asia**	Vietnam	Cần Thơ	NA	Vietnam	An Giang	Châu Phú
**Southeast Asia**	Vietnam	Cần Thơ	Bình Thuỷ	Vietnam	An Giang	Châu Thành
**Southeast Asia**	Vietnam	Cần Thơ	Cái Răng	Vietnam	An Giang	Châu Đốc
**Southeast Asia**	Vietnam	Cần Thơ	Cờ Đỏ	Vietnam	An Giang	Chợ Mới
**Southeast Asia**	Vietnam	Cần Thơ	Ninh Kiều	Vietnam	An Giang	Long Xuyên
**Southeast Asia**	Vietnam	Cần Thơ	Phong Điền	Vietnam	An Giang	Phú Tân
**Southeast Asia**	Vietnam	Cần Thơ	Thốt Nốt	Vietnam	An Giang	Thoại Sơn
**Southeast Asia**	Vietnam	Cần Thơ	Thới Lai	Vietnam	An Giang	Tân Châu
**Southeast Asia**	Vietnam	Cần Thơ	Vĩnh Thạnh	Vietnam	An Giang	Tịnh Biên
**Southeast Asia**	Vietnam	Cần Thơ	Ô Môn	Vietnam	Bắc Giang	Hiệp Hòa
**Southeast Asia**	Vietnam	Hoà Bình	Lạc Thủy	Vietnam	Bắc Giang	Lạng Giang

**Southeast Asia**	Vietnam	Hà Nam	NA	Vietnam	Bắc Giang	Tân Yên
**Southeast Asia**	Vietnam	Hà Nam	Bình Lục	Vietnam	Bắc Giang	Việt Yên
**Southeast Asia**	Vietnam	Hà Nam	Duy Tiên	Vietnam	Bắc Giang	Yên Dũng
**Southeast Asia**	Vietnam	Hà Nam	Kim Bảng	Vietnam	Bắc Giang	Yên Thế
**Southeast Asia**	Vietnam	Hà Nam	Lý Nhân	Vietnam	Bắc Kạn	Chợ Mới
**Southeast Asia**	Vietnam	Hà Nam	Phủ Lý	Vietnam	Bắc Ninh	NA
**Southeast Asia**	Vietnam	Hà Nam	Thanh Liêm	Vietnam	Bắc Ninh	Bắc Ninh
**Southeast Asia**	Vietnam	Hà Nội	NA	Vietnam	Bắc Ninh	Gia Bình
**Southeast Asia**	Vietnam	Hà Nội	Bắc Từ Liêm	Vietnam	Bắc Ninh	Lương Tài
**Southeast Asia**	Vietnam	Hà Nội	Gia Lâm	Vietnam	Bắc Ninh	Quế Võ
**Southeast Asia**	Vietnam	Hà Nội	Hai Bà Trưng	Vietnam	Bắc Ninh	Thuận Thành
**Southeast Asia**	Vietnam	Hà Nội	Hoài Đức	Vietnam	Bắc Ninh	Tiên Du
**Southeast Asia**	Vietnam	Hà Nội	Hoàng Mai	Vietnam	Bắc Ninh	Từ Sơn
**Southeast Asia**	Vietnam	Hà Nội	Hà Đông	Vietnam	Bắc Ninh	Yên Phong
**Southeast Asia**	Vietnam	Hà Nội	Long Biên	Vietnam	Cần Thơ	NA
**Southeast Asia**	Vietnam	Hà Nội	Mê Linh	Vietnam	Cần Thơ	Cờ Đỏ
**Southeast Asia**	Vietnam	Hà Nội	Mỹ Đức	Vietnam	Cần Thơ	Thốt Nốt
**Southeast Asia**	Vietnam	Hà Nội	Nam Từ Liêm	Vietnam	Cần Thơ	Thới Lai
**Southeast Asia**	Vietnam	Hà Nội	Phú Xuyên	Vietnam	Cần Thơ	Vĩnh Thạnh
**Southeast Asia**	Vietnam	Hà Nội	Sóc Sơn	Vietnam	Cần Thơ	Ô Môn
**Southeast Asia**	Vietnam	Hà Nội	Thanh Oai	Vietnam	Hà Nam	NA
**Southeast Asia**	Vietnam	Hà Nội	Thanh Trì	Vietnam	Hà Nam	Bình Lục
**Southeast Asia**	Vietnam	Hà Nội	Thanh Xuân	Vietnam	Hà Nam	Duy Tiên
**Southeast Asia**	Vietnam	Hà Nội	Thường Tín	Vietnam	Hà Nam	Kim Bảng
**Southeast Asia**	Vietnam	Hà Nội	Đan Phượng	Vietnam	Hà Nam	Lý Nhân
**Southeast Asia**	Vietnam	Hà Nội	Đông Anh	Vietnam	Hà Nam	Phủ Lý
**Southeast Asia**	Vietnam	Hà Nội	Ứng Hòa	Vietnam	Hà Nam	Thanh Liêm
**Southeast Asia**	Vietnam	Hưng Yên	NA	Vietnam	Hà Nội	NA
**Southeast Asia**	Vietnam	Hưng Yên	Hưng Yên	Vietnam	Hà Nội	Bắc Từ Liêm

**Southeast Asia**	Vietnam	Hưng Yên	Khoái Châu	Vietnam	Hà Nội	Gia Lâm
**Southeast Asia**	Vietnam	Hưng Yên	Kim Động	Vietnam	Hà Nội	Hoài Đức
**Southeast Asia**	Vietnam	Hưng Yên	Mỹ Hào	Vietnam	Hà Nội	Hà Đông
**Southeast Asia**	Vietnam	Hưng Yên	Phù Cừ	Vietnam	Hà Nội	Long Biên
**Southeast Asia**	Vietnam	Hưng Yên	Tiên Lữ	Vietnam	Hà Nội	Mê Linh
**Southeast Asia**	Vietnam	Hưng Yên	Văn Giang	Vietnam	Hà Nội	Mỹ Đức
**Southeast Asia**	Vietnam	Hưng Yên	Văn Lâm	Vietnam	Hà Nội	Phú Xuyên
**Southeast Asia**	Vietnam	Hưng Yên	Yên Mỹ	Vietnam	Hà Nội	Sóc Sơn
**Southeast Asia**	Vietnam	Hưng Yên	Ân Thi	Vietnam	Hà Nội	Thanh Trì
**Southeast Asia**	Vietnam	Hải Dương	Bình Giang	Vietnam	Hà Nội	Thường Tín
**Southeast Asia**	Vietnam	Hải Dương	Thanh Miện	Vietnam	Hà Nội	Đan Phượng
**Southeast Asia**	Vietnam	Hải Phòng	NA	Vietnam	Hà Nội	Đông Anh
**Southeast Asia**	Vietnam	Hải Phòng	An Dương	Vietnam	Hưng Yên	NA
**Southeast Asia**	Vietnam	Hải Phòng	An Lão	Vietnam	Hưng Yên	Khoái Châu
**Southeast Asia**	Vietnam	Hải Phòng	Dương Kinh	Vietnam	Hưng Yên	Kim Động
**Southeast Asia**	Vietnam	Hải Phòng	Hồng Bàng	Vietnam	Hưng Yên	Mỹ Hào
**Southeast Asia**	Vietnam	Hải Phòng	Kiến An	Vietnam	Hưng Yên	Phù Cừ
**Southeast Asia**	Vietnam	Hải Phòng	Kiến Thuỵ	Vietnam	Hưng Yên	Tiên Lữ
**Southeast Asia**	Vietnam	Hải Phòng	Tiên Lãng	Vietnam	Hưng Yên	Văn Giang
**Southeast Asia**	Vietnam	Hải Phòng	Vĩnh Bảo	Vietnam	Hưng Yên	Văn Lâm
**Southeast Asia**	Vietnam	Hậu Giang	NA	Vietnam	Hưng Yên	Yên Mỹ
**Southeast Asia**	Vietnam	Hậu Giang	Châu Thành	Vietnam	Hưng Yên	Ân Thi
**Southeast Asia**	Vietnam	Hậu Giang	Châu Thành A	Vietnam	Hải Phòng	Kiến Thuỵ
**Southeast Asia**	Vietnam	Hậu Giang	Ngã Bảy	Vietnam	Hậu Giang	Châu Thành
**Southeast Asia**	Vietnam	Hậu Giang	Phụng Hiệp	Vietnam	Hậu Giang	Châu Thành A
**Southeast Asia**	Vietnam	Kiên Giang	Giồng Riềng	Vietnam	Kiên Giang	Tân Hiệp
**Southeast Asia**	Vietnam	Kiên Giang	Tân Hiệp	Vietnam	Long An	NA
**Southeast Asia**	Vietnam	Long An	Bến Lức	Vietnam	Long An	Kiến Tường
**Southeast Asia**	Vietnam	Long An	Tân Hưng	Vietnam	Long An	Tân Hưng

**Southeast Asia**	Vietnam	Nam Định	Mỹ Lộc	Vietnam	Long An	Tân Thạnh
**Southeast Asia**	Vietnam	Nam Định	Ý Yên	Vietnam	Long An	Vĩnh Hưng
**Southeast Asia**	Vietnam	Ninh Bình	NA	Vietnam	Ninh Bình	Gia Viễn
**Southeast Asia**	Vietnam	Ninh Bình	Gia Viễn	Vietnam	Ninh Bình	Hoa Lư
**Southeast Asia**	Vietnam	Ninh Bình	Hoa Lư	Vietnam	Ninh Bình	Nho Quan
**Southeast Asia**	Vietnam	Ninh Bình	Nho Quan	Vietnam	Sóc Trăng	NA
**Southeast Asia**	Vietnam	Ninh Bình	Ninh Bình	Vietnam	Sóc Trăng	Châu Thành
**Southeast Asia**	Vietnam	Ninh Bình	Tam Điệp	Vietnam	Sóc Trăng	Kế Sách
**Southeast Asia**	Vietnam	Ninh Bình	Yên Mô	Vietnam	Sóc Trăng	Long Phú
**Southeast Asia**	Vietnam	Sóc Trăng	NA	Vietnam	Sóc Trăng	Sóc Trăng
**Southeast Asia**	Vietnam	Sóc Trăng	Châu Thành	Vietnam	Sóc Trăng	Trần Đề
**Southeast Asia**	Vietnam	Sóc Trăng	Cù Lao Dung	Vietnam	Thái Bình	Hưng Hà
**Southeast Asia**	Vietnam	Sóc Trăng	Kế Sách	Vietnam	Thái Nguyên	NA
**Southeast Asia**	Vietnam	Sóc Trăng	Long Phú	Vietnam	Thái Nguyên	Phú Bình
**Southeast Asia**	Vietnam	Sóc Trăng	Mỹ Tú	Vietnam	Thái Nguyên	Phú Lương
**Southeast Asia**	Vietnam	Sóc Trăng	Mỹ Xuyên	Vietnam	Thái Nguyên	Phổ Yên
**Southeast Asia**	Vietnam	Sóc Trăng	Sóc Trăng	Vietnam	Thái Nguyên	Sông Công
**Southeast Asia**	Vietnam	Sóc Trăng	Trần Đề	Vietnam	Thái Nguyên	Thái Nguyên
**Southeast Asia**	Vietnam	Thái Bình	NA	Vietnam	Thái Nguyên	Võ Nhai
**Southeast Asia**	Vietnam	Thái Bình	Hưng Hà	Vietnam	Thái Nguyên	Đại Từ
**Southeast Asia**	Vietnam	Thái Bình	Kiến Xương	Vietnam	Thái Nguyên	Định Hóa
**Southeast Asia**	Vietnam	Thái Bình	Quỳnh Phụ	Vietnam	Thái Nguyên	Đồng Hỷ
**Southeast Asia**	Vietnam	Thái Bình	Thái Bình	Vietnam	Trà Vinh	Càng Long
**Southeast Asia**	Vietnam	Thái Bình	Thái Thụy	Vietnam	Trà Vinh	Cầu Kè
**Southeast Asia**	Vietnam	Thái Bình	Tiền Hải	Vietnam	Trà Vinh	Tiểu Cần
**Southeast Asia**	Vietnam	Thái Bình	Đông Hưng	Vietnam	Vĩnh Long	NA
**Southeast Asia**	Vietnam	Thái Nguyên	NA	Vietnam	Vĩnh Long	Bình Tân
**Southeast Asia**	Vietnam	Thái Nguyên	Phú Bình	Vietnam	Vĩnh Long	Long Hồ
**Southeast Asia**	Vietnam	Thái Nguyên	Phú Lương	Vietnam	Vĩnh Long	Mang Thít

**Southeast Asia**	Vietnam	Thái Nguyên	Phổ Yên	Vietnam	Vĩnh Long	Tam Bình
**Southeast Asia**	Vietnam	Thái Nguyên	Sông Công	Vietnam	Vĩnh Long	Trà Ôn
**Southeast Asia**	Vietnam	Thái Nguyên	Thái Nguyên	Vietnam	Vĩnh Long	Vũng Liêm
**Southeast Asia**	Vietnam	Thái Nguyên	Võ Nhai	Vietnam	Vĩnh Phúc	NA
**Southeast Asia**	Vietnam	Thái Nguyên	Đại Từ	Vietnam	Vĩnh Phúc	Bình Xuyên
**Southeast Asia**	Vietnam	Thái Nguyên	Định Hóa	Vietnam	Vĩnh Phúc	Phúc Yên
**Southeast Asia**	Vietnam	Thái Nguyên	Đồng Hỷ	Vietnam	Vĩnh Phúc	Tam Dương
**Southeast Asia**	Vietnam	Trà Vinh	NA	Vietnam	Vĩnh Phúc	Tam Đảo
**Southeast Asia**	Vietnam	Trà Vinh	Châu Thành	Vietnam	Vĩnh Phúc	Vĩnh Yên
**Southeast Asia**	Vietnam	Trà Vinh	Càng Long	Vietnam	Đồng Tháp	NA
**Southeast Asia**	Vietnam	Trà Vinh	Cầu Kè	Vietnam	Đồng Tháp	Cao Lãnh
**Southeast Asia**	Vietnam	Trà Vinh	Cầu Ngang	Vietnam	Đồng Tháp	Cao Lãnh (Thành phố)
**Southeast Asia**	Vietnam	Trà Vinh	Tiểu Cần	Vietnam	Đồng Tháp	Châu Thành
**Southeast Asia**	Vietnam	Trà Vinh	Trà Cú	Vietnam	Đồng Tháp	Hồng Ngự
**Southeast Asia**	Vietnam	Trà Vinh	Trà Vinh	Vietnam	Đồng Tháp	Hồng Ngự (Thị xã)
**Southeast Asia**	Vietnam	Vĩnh Long	NA	Vietnam	Đồng Tháp	Lai Vung
**Southeast Asia**	Vietnam	Vĩnh Long	Bình Minh	Vietnam	Đồng Tháp	Lấp Vò
**Southeast Asia**	Vietnam	Vĩnh Long	Bình Tân	Vietnam	Đồng Tháp	Sa Đéc
**Southeast Asia**	Vietnam	Vĩnh Long	Long Hồ	Vietnam	Đồng Tháp	Tam Nông
**Southeast Asia**	Vietnam	Vĩnh Long	Mang Thít	Vietnam	Đồng Tháp	Thanh Bình
**Southeast Asia**	Vietnam	Vĩnh Long	Tam Bình	Vietnam	Đồng Tháp	Tháp Mười
**Southeast Asia**	Vietnam	Vĩnh Long	Trà Ôn	Vietnam	Đồng Tháp	Tân Hồng
**Southeast Asia**	Vietnam	Vĩnh Long	Vĩnh Long	NA	NA	NA
**Southeast Asia**	Vietnam	Vĩnh Long	Vũng Liêm	NA	NA	NA
**Southeast Asia**	Vietnam	Vĩnh Phúc	Bình Xuyên	NA	NA	NA
**Southeast Asia**	Vietnam	Vĩnh Phúc	Phúc Yên	NA	NA	NA
**Southeast Asia**	Vietnam	Vĩnh Phúc	Vĩnh Yên	NA	NA	NA
**Southeast Asia**	Vietnam	Đồng Tháp	NA	NA	NA	NA

**Southeast Asia**	Vietnam	Đồng Tháp	Cao Lãnh	NA	NA	NA
**Southeast Asia**	Vietnam	Đồng Tháp	Cao Lãnh (Thành phố)	NA	NA	NA
**Southeast Asia**	Vietnam	Đồng Tháp	Châu Thành	NA	NA	NA
**Southeast Asia**	Vietnam	Đồng Tháp	Hồng Ngự	NA	NA	NA
**Southeast Asia**	Vietnam	Đồng Tháp	Hồng Ngự (Thị xã)	NA	NA	NA
**Southeast Asia**	Vietnam	Đồng Tháp	Lai Vung	NA	NA	NA
**Southeast Asia**	Vietnam	Đồng Tháp	Lấp Vò	NA	NA	NA
**Southeast Asia**	Vietnam	Đồng Tháp	Sa Đéc	NA	NA	NA
**Southeast Asia**	Vietnam	Đồng Tháp	Tam Nông	NA	NA	NA
**Southeast Asia**	Vietnam	Đồng Tháp	Thanh Bình	NA	NA	NA
**Southeast Asia**	Vietnam	Đồng Tháp	Tháp Mười	NA	NA	NA
**Southeast Asia**	Vietnam	Đồng Tháp	Tân Hồng	NA	NA	NA
**Tropical South America**	Brazil	Piauí	NA	Brazil	Piauí	NA
**Tropical South America**	Brazil	Piauí	Acauã	Brazil	Piauí	Acauã
**Tropical South America**	Brazil	Piauí	Agricolândia	Brazil	Piauí	Agricolândia
**Tropical South America**	Brazil	Piauí	Alagoinha do Piauí	Brazil	Piauí	Alagoinha do Piauí
**Tropical South America**	Brazil	Piauí	Alegrete do Piauí	Brazil	Piauí	Alegrete do Piauí
**Tropical South America**	Brazil	Piauí	Alto Longá	Brazil	Piauí	Alto Longá
**Tropical South America**	Brazil	Piauí	Altos	Brazil	Piauí	Altos
**Tropical South America**	Brazil	Piauí	Alvorada do Gurguéia	Brazil	Piauí	Alvorada do Gurguéia
**Tropical South America**	Brazil	Piauí	Amarante	Brazil	Piauí	Angical do Piauí

**Tropical South America**	Brazil	Piauí	Angical do Piauí	Brazil	Piauí	Antônio Almeida
**Tropical South America**	Brazil	Piauí	Antônio Almeida	Brazil	Piauí	Anísio de Abreu
**Tropical South America**	Brazil	Piauí	Anísio de Abreu	Brazil	Piauí	Aroazes
**Tropical South America**	Brazil	Piauí	Aroazes	Brazil	Piauí	Arraial
**Tropical South America**	Brazil	Piauí	Arraial	Brazil	Piauí	Avelino Lopes
**Tropical South America**	Brazil	Piauí	Avelino Lopes	Brazil	Piauí	Baixa Grande do Ribeiro
**Tropical South America**	Brazil	Piauí	Baixa Grande do Ribeiro	Brazil	Piauí	Barra d'Alcântara
**Tropical South America**	Brazil	Piauí	Barra d'Alcântara	Brazil	Piauí	Barras
**Tropical South America**	Brazil	Piauí	Barras	Brazil	Piauí	Barreiras do Piauí
**Tropical South America**	Brazil	Piauí	Barreiras do Piauí	Brazil	Piauí	Barro Duro
**Tropical South America**	Brazil	Piauí	Barro Duro	Brazil	Piauí	Batalha
**Tropical South America**	Brazil	Piauí	Batalha	Brazil	Piauí	Belém do Piauí
**Tropical South America**	Brazil	Piauí	Bela Vista do Piauí	Brazil	Piauí	Beneditinos
**Tropical South America**	Brazil	Piauí	Belém do Piauí	Brazil	Piauí	Bertolínia
**Tropical South America**	Brazil	Piauí	Beneditinos	Brazil	Piauí	Boa Hora

**Tropical South America**	Brazil	Piauí	Bertolínia	Brazil	Piauí	Bocaina
**Tropical South America**	Brazil	Piauí	Betânia do Piauí	Brazil	Piauí	Bom Jesus
**Tropical South America**	Brazil	Piauí	Boa Hora	Brazil	Piauí	Bonfim do Piauí
**Tropical South America**	Brazil	Piauí	Bocaina	Brazil	Piauí	Boqueirão do Piauí
**Tropical South America**	Brazil	Piauí	Bom Jesus	Brazil	Piauí	Brazileira
**Tropical South America**	Brazil	Piauí	Bonfim do Piauí	Brazil	Piauí	Buriti dos Montes
**Tropical South America**	Brazil	Piauí	Boqueirão do Piauí	Brazil	Piauí	Cabeceiras do Piauí
**Tropical South America**	Brazil	Piauí	Brazileira	Brazil	Piauí	Caldeirão Grande do Piauí
**Tropical South America**	Brazil	Piauí	Brejo do Piauí	Brazil	Piauí	Campinas do Piauí
**Tropical South America**	Brazil	Piauí	Buriti dos Lopes	Brazil	Piauí	Campo Grande do Piauí
**Tropical South America**	Brazil	Piauí	Buriti dos Montes	Brazil	Piauí	Campo Largo do Piauí
**Tropical South America**	Brazil	Piauí	Cabeceiras do Piauí	Brazil	Piauí	Campo Maior
**Tropical South America**	Brazil	Piauí	Cajazeiras do Piauí	Brazil	Piauí	Canavieira
**Tropical South America**	Brazil	Piauí	Caldeirão Grande do Piauí	Brazil	Piauí	Canto do Buriti
**Tropical South America**	Brazil	Piauí	Campinas do Piauí	Brazil	Piauí	Capitão de Campos

**Tropical South America**	Brazil	Piauí	Campo Alegre do Fidalgo	Brazil	Piauí	Caracol
**Tropical South America**	Brazil	Piauí	Campo Grande do Piauí	Brazil	Piauí	Castelo do Piauí
**Tropical South America**	Brazil	Piauí	Campo Largo do Piauí	Brazil	Piauí	Coivaras
**Tropical South America**	Brazil	Piauí	Campo Maior	Brazil	Piauí	Colônia do Gurguéia
**Tropical South America**	Brazil	Piauí	Canavieira	Brazil	Piauí	Colônia do Piauí
**Tropical South America**	Brazil	Piauí	Canto do Buriti	Brazil	Piauí	Conceição do Canindé
**Tropical South America**	Brazil	Piauí	Capitão Gervásio Oliveira	Brazil	Piauí	Coronel José Dias
**Tropical South America**	Brazil	Piauí	Capitão de Campos	Brazil	Piauí	Corrente
**Tropical South America**	Brazil	Piauí	Caracol	Brazil	Piauí	Cristalândia do Piauí
**Tropical South America**	Brazil	Piauí	Caraúbas do Piauí	Brazil	Piauí	Cristino Castro
**Tropical South America**	Brazil	Piauí	Caridade do Piauí	Brazil	Piauí	Curimatá
**Tropical South America**	Brazil	Piauí	Castelo do Piauí	Brazil	Piauí	Currais
**Tropical South America**	Brazil	Piauí	Cocal	Brazil	Piauí	Demerval Lobão
**Tropical South America**	Brazil	Piauí	Cocal de Telha	Brazil	Piauí	Dirceu Arcoverde
**Tropical South America**	Brazil	Piauí	Cocal dos Alves	Brazil	Piauí	Dom Expedito Lopes

**Tropical South America**	Brazil	Piauí	Coivaras	Brazil	Piauí	Dom Inocêncio
**Tropical South America**	Brazil	Piauí	Colônia do Gurguéia	Brazil	Piauí	Domingos Mourão
**Tropical South America**	Brazil	Piauí	Colônia do Piauí	Brazil	Piauí	Elesbão Veloso
**Tropical South America**	Brazil	Piauí	Conceição do Canindé	Brazil	Piauí	Eliseu Martins
**Tropical South America**	Brazil	Piauí	Coronel José Dias	Brazil	Piauí	Esperantina
**Tropical South America**	Brazil	Piauí	Corrente	Brazil	Piauí	Fartura do Piauí
**Tropical South America**	Brazil	Piauí	Cristalândia do Piauí	Brazil	Piauí	Flores do Piauí
**Tropical South America**	Brazil	Piauí	Cristino Castro	Brazil	Piauí	Floresta do Piauí
**Tropical South America**	Brazil	Piauí	Curimatá	Brazil	Piauí	Floriano
**Tropical South America**	Brazil	Piauí	Currais	Brazil	Piauí	Francinópolis
**Tropical South America**	Brazil	Piauí	Curral Novo do Piauí	Brazil	Piauí	Francisco Ayres
**Tropical South America**	Brazil	Piauí	Curralinhos	Brazil	Piauí	Francisco Santos
**Tropical South America**	Brazil	Piauí	Demerval Lobão	Brazil	Piauí	Fronteiras
**Tropical South America**	Brazil	Piauí	Dirceu Arcoverde	Brazil	Piauí	Geminiano
**Tropical South America**	Brazil	Piauí	Dom Expedito Lopes	Brazil	Piauí	Gilbués

**Tropical South America**	Brazil	Piauí	Dom Inocêncio	Brazil	Piauí	Guadalupe
**Tropical South America**	Brazil	Piauí	Domingos Mourão	Brazil	Piauí	Guaribas
**Tropical South America**	Brazil	Piauí	Elesbão Veloso	Brazil	Piauí	Hugo Napoleão
**Tropical South America**	Brazil	Piauí	Eliseu Martins	Brazil	Piauí	Inhuma
**Tropical South America**	Brazil	Piauí	Esperantina	Brazil	Piauí	Ipiranga do Piauí
**Tropical South America**	Brazil	Piauí	Fartura do Piauí	Brazil	Piauí	Isaías Coelho
**Tropical South America**	Brazil	Piauí	Flores do Piauí	Brazil	Piauí	Itainópolis
**Tropical South America**	Brazil	Piauí	Floresta do Piauí	Brazil	Piauí	Itaueira
**Tropical South America**	Brazil	Piauí	Floriano	Brazil	Piauí	Jacobina do Piauí
**Tropical South America**	Brazil	Piauí	Francinópolis	Brazil	Piauí	Jaicós
**Tropical South America**	Brazil	Piauí	Francisco Ayres	Brazil	Piauí	Jardim do Mulato
**Tropical South America**	Brazil	Piauí	Francisco Macêdo	Brazil	Piauí	Jerumenha
**Tropical South America**	Brazil	Piauí	Francisco Santos	Brazil	Piauí	José de Freitas
**Tropical South America**	Brazil	Piauí	Fronteiras	Brazil	Piauí	Juazeiro do Piauí
**Tropical South America**	Brazil	Piauí	Geminiano	Brazil	Piauí	Jurema

**Tropical South America**	Brazil	Piauí	Gilbués	Brazil	Piauí	Júlio Borges
**Tropical South America**	Brazil	Piauí	Guadalupe	Brazil	Piauí	Lagoa Alegre
**Tropical South America**	Brazil	Piauí	Guaribas	Brazil	Piauí	Lagoa de São Francisco
**Tropical South America**	Brazil	Piauí	Hugo Napoleão	Brazil	Piauí	Lagoa do Barro do Piauí
**Tropical South America**	Brazil	Piauí	Inhuma	Brazil	Piauí	Lagoa do Piauí
**Tropical South America**	Brazil	Piauí	Ipiranga do Piauí	Brazil	Piauí	Lagoa do Sítio
**Tropical South America**	Brazil	Piauí	Isaías Coelho	Brazil	Piauí	Lagoinha do Piauí
**Tropical South America**	Brazil	Piauí	Itainópolis	Brazil	Piauí	Landri Sales
**Tropical South America**	Brazil	Piauí	Itaueira	Brazil	Piauí	Manoel Emídio
**Tropical South America**	Brazil	Piauí	Jacobina do Piauí	Brazil	Piauí	Marcolândia
**Tropical South America**	Brazil	Piauí	Jaicós	Brazil	Piauí	Marcos Parente
**Tropical South America**	Brazil	Piauí	Jardim do Mulato	Brazil	Piauí	Miguel Leão
**Tropical South America**	Brazil	Piauí	Jatobá do Piauí	Brazil	Piauí	Milton Brandão
**Tropical South America**	Brazil	Piauí	Jerumenha	Brazil	Piauí	Monsenhor Gil
**Tropical South America**	Brazil	Piauí	Joaquim Pires	Brazil	Piauí	Monsenhor Hipólito

**Tropical South America**	Brazil	Piauí	Joca Marques	Brazil	Piauí	Monte Alegre do Piauí
**Tropical South America**	Brazil	Piauí	José de Freitas	Brazil	Piauí	Morro Cabeça No Tempo
**Tropical South America**	Brazil	Piauí	Juazeiro do Piauí	Brazil	Piauí	Morro do Chapéu do Piauí
**Tropical South America**	Brazil	Piauí	Jurema	Brazil	Piauí	Nazaré do Piauí
**Tropical South America**	Brazil	Piauí	Júlio Borges	Brazil	Piauí	Novo Oriente do Piauí
**Tropical South America**	Brazil	Piauí	Lagoa Alegre	Brazil	Piauí	Novo Santo Antônio
**Tropical South America**	Brazil	Piauí	Lagoa de São Francisco	Brazil	Piauí	Oeiras
**Tropical South America**	Brazil	Piauí	Lagoa do Barro do Piauí	Brazil	Piauí	Olho d'água do Piauí
**Tropical South America**	Brazil	Piauí	Lagoa do Piauí	Brazil	Piauí	Padre Marcos
**Tropical South America**	Brazil	Piauí	Lagoa do Sítio	Brazil	Piauí	Paes Landim
**Tropical South America**	Brazil	Piauí	Lagoinha do Piauí	Brazil	Piauí	Palmeira do Piauí
**Tropical South America**	Brazil	Piauí	Landri Sales	Brazil	Piauí	Palmeirais
**Tropical South America**	Brazil	Piauí	Luzilândia	Brazil	Piauí	Paquetá
**Tropical South America**	Brazil	Piauí	Madeiro	Brazil	Piauí	Parnaguá
**Tropical South America**	Brazil	Piauí	Manoel Emídio	Brazil	Piauí	Passagem Franca do Piauí

**Tropical South America**	Brazil	Piauí	Marcolândia	Brazil	Piauí	Patos do Piauí
**Tropical South America**	Brazil	Piauí	Marcos Parente	Brazil	Piauí	Paulistana
**Tropical South America**	Brazil	Piauí	Massapê do Piauí	Brazil	Piauí	Pavussu
**Tropical South America**	Brazil	Piauí	Matias Olímpio	Brazil	Piauí	Pedro Li
**Tropical South America**	Brazil	Piauí	Miguel Alves	Brazil	Piauí	Picos
**Tropical South America**	Brazil	Piauí	Miguel Leão	Brazil	Piauí	Pimenteiras
**Tropical South America**	Brazil	Piauí	Milton Brandão	Brazil	Piauí	Pio IX
**Tropical South America**	Brazil	Piauí	Monsenhor Gil	Brazil	Piauí	Piripiri
**Tropical South America**	Brazil	Piauí	Monsenhor Hipólito	Brazil	Piauí	Porto Alegre do Piauí
**Tropical South America**	Brazil	Piauí	Monte Alegre do Piauí	Brazil	Piauí	Prata do Piauí
**Tropical South America**	Brazil	Piauí	Morro Cabeça No Tempo	Brazil	Piauí	Queimada Nova
**Tropical South America**	Brazil	Piauí	Morro do Chapéu do Piauí	Brazil	Piauí	Redenção do Gurguéia
**Tropical South America**	Brazil	Piauí	Nazaré do Piauí	Brazil	Piauí	Regeneração
**Tropical South America**	Brazil	Piauí	Nossa Senhora de Nazaré	Brazil	Piauí	Riacho Frio
**Tropical South America**	Brazil	Piauí	Nossa Senhora dos Remédios	Brazil	Piauí	Ribeiro Gonçalves

**Tropical South America**	Brazil	Piauí	Nova Santa Rita	Brazil	Piauí	Rio Grande do Piauí
**Tropical South America**	Brazil	Piauí	Novo Oriente do Piauí	Brazil	Piauí	Santa Cruz dos Milagres
**Tropical South America**	Brazil	Piauí	Novo Santo Antônio	Brazil	Piauí	Santa Filomena
**Tropical South America**	Brazil	Piauí	Oeiras	Brazil	Piauí	Santa Luz
**Tropical South America**	Brazil	Piauí	Olho d'água do Piauí	Brazil	Piauí	Santa Rosa do Piauí
**Tropical South America**	Brazil	Piauí	Padre Marcos	Brazil	Piauí	Santana do Piauí
**Tropical South America**	Brazil	Piauí	Paes Landim	Brazil	Piauí	Santo Antônio de Lisboa
**Tropical South America**	Brazil	Piauí	Pajeú do Piauí	Brazil	Piauí	Santo Antônio dos Milagres
**Tropical South America**	Brazil	Piauí	Palmeira do Piauí	Brazil	Piauí	Santo Inácio do Piauí
**Tropical South America**	Brazil	Piauí	Palmeirais	Brazil	Piauí	Sebastião Barros
**Tropical South America**	Brazil	Piauí	Paquetá	Brazil	Piauí	Sebastião Leal
**Tropical South America**	Brazil	Piauí	Parnaguá	Brazil	Piauí	Sigefredo Pacheco
**Tropical South America**	Brazil	Piauí	Parnaíba	Brazil	Piauí	Simões
**Tropical South America**	Brazil	Piauí	Passagem Franca do Piauí	Brazil	Piauí	Socorro do Piauí
**Tropical South America**	Brazil	Piauí	Patos do Piauí	Brazil	Piauí	Sussuapara

**Tropical South America**	Brazil	Piauí	Paulistana	Brazil	Piauí	São Braz do Piauí
**Tropical South America**	Brazil	Piauí	Pavussu	Brazil	Piauí	São Francisco do Piauí
**Tropical South America**	Brazil	Piauí	Pedro Laurentino	Brazil	Piauí	São Félix do Piauí
**Tropical South America**	Brazil	Piauí	Pedro Li	Brazil	Piauí	São Gonçalo do Gurguéia
**Tropical South America**	Brazil	Piauí	Picos	Brazil	Piauí	São Gonçalo do Piauí
**Tropical South America**	Brazil	Piauí	Pimenteiras	Brazil	Piauí	São José do Divino
**Tropical South America**	Brazil	Piauí	Pio IX	Brazil	Piauí	São José do Peixe
**Tropical South America**	Brazil	Piauí	Piracuruca	Brazil	Piauí	São José do Piauí
**Tropical South America**	Brazil	Piauí	Piripiri	Brazil	Piauí	São João Piaui
**Tropical South America**	Brazil	Piauí	Porto	Brazil	Piauí	São João da Canabrava
**Tropical South America**	Brazil	Piauí	Porto Alegre do Piauí	Brazil	Piauí	São João da Serra
**Tropical South America**	Brazil	Piauí	Prata do Piauí	Brazil	Piauí	São João da Varjota
**Tropical South America**	Brazil	Piauí	Queimada Nova	Brazil	Piauí	São Juliao
**Tropical South America**	Brazil	Piauí	Redenção do Gurguéia	Brazil	Piauí	São Lourenço do Piauí
**Tropical South America**	Brazil	Piauí	Regeneração	Brazil	Piauí	São Luis do Piauí

**Tropical South America**	Brazil	Piauí	Riacho Frio	Brazil	Piauí	São Miguel Tapuio
**Tropical South America**	Brazil	Piauí	Ribeira do Piauí	Brazil	Piauí	São Miguel da Baixa Grande
**Tropical South America**	Brazil	Piauí	Ribeiro Gonçalves	Brazil	Piauí	São Miguel do Fidalgo
**Tropical South America**	Brazil	Piauí	Rio Grande do Piauí	Brazil	Piauí	São Pedro do Piauí
**Tropical South America**	Brazil	Piauí	Santa Cruz do Piaui	Brazil	Piauí	São Raimundo Nonato
**Tropical South America**	Brazil	Piauí	Santa Cruz dos Milagres	Brazil	Piauí	Tanque do Piauí
**Tropical South America**	Brazil	Piauí	Santa Filomena	Brazil	Piauí	Teresina
**Tropical South America**	Brazil	Piauí	Santa Luz	Brazil	Piauí	União
**Tropical South America**	Brazil	Piauí	Santa Rosa do Piauí	Brazil	Piauí	Uruçuí
**Tropical South America**	Brazil	Piauí	Santana do Piauí	Brazil	Piauí	Valença do Piauí
**Tropical South America**	Brazil	Piauí	Santo Antônio de Lisboa	Brazil	Piauí	Vila Nova do Piauí
**Tropical South America**	Brazil	Piauí	Santo Antônio dos Milagres	Brazil	Piauí	Várzea Grande
**Tropical South America**	Brazil	Piauí	Santo Inácio do Piauí	Brazil	Piauí	Wall Ferraz
**Tropical South America**	Brazil	Piauí	Sebastião Barros	Brazil	Piauí	Água Branca
**Tropical South America**	Brazil	Piauí	Sebastião Leal	NA	NA	NA

**Tropical South America**	Brazil	Piauí	Sigefredo Pacheco	NA	NA	NA
**Tropical South America**	Brazil	Piauí	Simplício Mendes	NA	NA	NA
**Tropical South America**	Brazil	Piauí	Simões	NA	NA	NA
**Tropical South America**	Brazil	Piauí	Socorro do Piauí	NA	NA	NA
**Tropical South America**	Brazil	Piauí	Sussuapara	NA	NA	NA
**Tropical South America**	Brazil	Piauí	São Braz do Piauí	NA	NA	NA
**Tropical South America**	Brazil	Piauí	São Francisco de Assis do Piauí	NA	NA	NA
**Tropical South America**	Brazil	Piauí	São Francisco do Piauí	NA	NA	NA
**Tropical South America**	Brazil	Piauí	São Félix do Piauí	NA	NA	NA
**Tropical South America**	Brazil	Piauí	São Gonçalo do Gurguéia	NA	NA	NA
**Tropical South America**	Brazil	Piauí	São Gonçalo do Piauí	NA	NA	NA
**Tropical South America**	Brazil	Piauí	São José do Divino	NA	NA	NA
**Tropical South America**	Brazil	Piauí	São José do Peixe	NA	NA	NA
**Tropical South America**	Brazil	Piauí	São José do Piauí	NA	NA	NA
**Tropical South America**	Brazil	Piauí	São João Piaui	NA	NA	NA

**Tropical South America**	Brazil	Piauí	São João da Canabrava	NA	NA	NA
**Tropical South America**	Brazil	Piauí	São João da Fronteira	NA	NA	NA
**Tropical South America**	Brazil	Piauí	São João da Serra	NA	NA	NA
**Tropical South America**	Brazil	Piauí	São João da Varjota	NA	NA	NA
**Tropical South America**	Brazil	Piauí	São João do Arraial	NA	NA	NA
**Tropical South America**	Brazil	Piauí	São Juliao	NA	NA	NA
**Tropical South America**	Brazil	Piauí	São Lourenço do Piauí	NA	NA	NA
**Tropical South America**	Brazil	Piauí	São Luis do Piauí	NA	NA	NA
**Tropical South America**	Brazil	Piauí	São Miguel Tapuio	NA	NA	NA
**Tropical South America**	Brazil	Piauí	São Miguel da Baixa Grande	NA	NA	NA
**Tropical South America**	Brazil	Piauí	São Miguel do Fidalgo	NA	NA	NA
**Tropical South America**	Brazil	Piauí	São Pedro do Piauí	NA	NA	NA
**Tropical South America**	Brazil	Piauí	São Raimundo Nonato	NA	NA	NA
**Tropical South America**	Brazil	Piauí	Tamboril do Piauí	NA	NA	NA
**Tropical South America**	Brazil	Piauí	Tanque do Piauí	NA	NA	NA

**Tropical South America**	Brazil	Piauí	Teresina	NA	NA	NA
**Tropical South America**	Brazil	Piauí	União	NA	NA	NA
**Tropical South America**	Brazil	Piauí	Uruçuí	NA	NA	NA
**Tropical South America**	Brazil	Piauí	Valença do Piauí	NA	NA	NA
**Tropical South America**	Brazil	Piauí	Vera Mendes	NA	NA	NA
**Tropical South America**	Brazil	Piauí	Vila Nova do Piauí	NA	NA	NA
**Tropical South America**	Brazil	Piauí	Várzea Grande	NA	NA	NA
**Tropical South America**	Brazil	Piauí	Wall Ferraz	NA	NA	NA
**Tropical South America**	Brazil	Piauí	Água Branca	NA	NA	NA

Supplementary Table 15: Anaemia fitted parameters

Lower, median, and upper quantiles (percentiles 0.025, 0.50, 0.975) are displayed for the main parameters from the models by region. The fixed effects covariates corresponding to the predicted ensemble rasters are shown in the first five columns, while fitted values for the spatiotemporal field hyperparameters and the precisions (inverse variance) for our random effects are shown in the last five columns.

Regions	Percentiles	int	gam	gbm	lasso	Nominal Range	Nominal Variance	AR1 rho	Precision for Nugget	Precision for Country RE
North Africa	0.025	-0.38	-0.12	0.32	0.39	0.04	0.29	0.09	4.25	1213.18
	0.500	-0.08	0.00	0.48	0.52	0.06	0.54	0.38	5.29	12882.99
	0.975	0.22	0.12	0.64	0.64	0.10	1.10	0.65	6.50	66780.16
Southern sub-Saharan Africa	0.025	-0.25	-0.17	0.11	0.52	0.05	0.16	-0.46	12.74	1170.41
	0.500	-0.10	0.01	0.32	0.68	0.08	0.26	-0.20	18.42	12974.33
	0.975	0.04	0.19	0.52	0.83	0.12	0.42	0.18	27.02	67050.49
Eastern sub-Saharan Africa	0.025	-0.08	-0.01	0.08	0.67	0.04	0.10	0.14	4.77	32.95
	0.500	0.03	0.10	0.18	0.72	0.05	0.15	0.47	5.21	690.24
	0.975	0.13	0.21	0.29	0.77	0.07	0.20	0.64	5.68	166402.50
Western sub-Saharan Africa	0.025	-0.25	0.05	0.13	0.50	0.04	0.19	-0.23	7.69	8.14
	0.500	-0.09	0.18	0.26	0.55	0.06	0.26	-0.07	8.51	23.66
	0.975	0.06	0.32	0.40	0.61	0.08	0.34	0.12	9.54	76.70
Oceania	0.025	-0.43	-0.08	0.07	0.76	0.25	0.26	-0.73	840.19	1276.53
	0.500	0.03	0.01	0.17	0.82	0.49	0.60	-0.44	11331.49	13144.44
	0.975	0.48	0.11	0.26	0.87	1.07	1.57	-0.01	63631.38	67062.79
Middle East	0.025	-0.50	-0.10	-0.07	0.70	0.17	0.00	-0.50	5.47	1072.96
	0.500	-0.16	0.06	0.11	0.82	0.69	0.02	0.58	6.43	13365.11
	0.975	0.18	0.23	0.30	0.95	2.90	0.19	0.97	7.54	68517.94
Central America and the Caribbean	0.025	-0.66	-0.07	0.28	0.59	0.04	0.29	0.11	4.46	0.88
	0.500	-0.15	0.00	0.36	0.65	0.06	0.44	0.40	4.99	3.60
	0.975	0.36	0.06	0.43	0.71	0.09	0.68	0.66	5.57	12.90
	0.025	-0.15	-0.19	-0.16	0.55	0.06	0.00	-0.94	2368.28	2090.13

Tropical South America	0.500	-0.04	0.14	0.15	0.71	0.43	0.01	-0.57	17070.20	16318.90
	0.975	0.06	0.47	0.45	0.88	3.88	0.10	0.53	93252.33	87946.98
Andean South America	0.025	-0.32	-0.17	0.12	0.85	0.07	0.37	-0.51	6.37	8.55
	0.500	-0.11	-0.09	0.21	0.88	0.08	0.51	-0.38	7.07	86.75
	0.975	0.11	-0.01	0.29	0.92	0.11	0.74	-0.25	7.94	2459.14
Central sub-Saharan Africa	0.025	-0.18	-0.08	-0.04	0.65	0.06	0.08	-0.64	11.15	720.76
	0.500	-0.06	0.10	0.13	0.77	0.09	0.18	-0.23	16.84	10871.93
	0.975	0.06	0.28	0.30	0.89	0.15	0.38	0.23	24.57	63062.24
South Asia	0.025	-0.37	-0.14	0.26	0.35	0.03	0.30	0.36	8.68	1153.71
	0.500	-0.21	0.00	0.46	0.54	0.04	0.44	0.75	19.38	13811.58
	0.975	-0.05	0.15	0.66	0.72	0.05	0.62	0.94	42.84	69083.44
Central Asia	0.025	-0.14	-0.06	0.14	0.72	0.08	0.34	0.05	4.86	8127.91
	0.500	0.06	0.02	0.22	0.76	0.10	0.52	0.10	4.92	15317.64
	0.975	0.26	0.10	0.30	0.80	0.16	0.96	0.18	4.95	23031.86
Southeast Asia	0.025	-0.23	-0.06	0.00	0.47	0.05	0.18	-0.76	1.50	1238.35
	0.500	0.09	0.17	0.23	0.60	0.10	0.39	-0.26	1.69	14269.91
	0.975	0.40	0.39	0.47	0.73	0.19	0.89	0.48	1.85	70166.50
East Asia	0.025	-0.88	-0.02	0.12	0.45	0.02	0.07	-0.37	7.89	0.57
	0.500	-0.41	0.14	0.29	0.57	0.04	0.11	0.02	9.43	3.52
	0.975	0.07	0.30	0.46	0.70	0.06	0.18	0.36	11.77	19.02

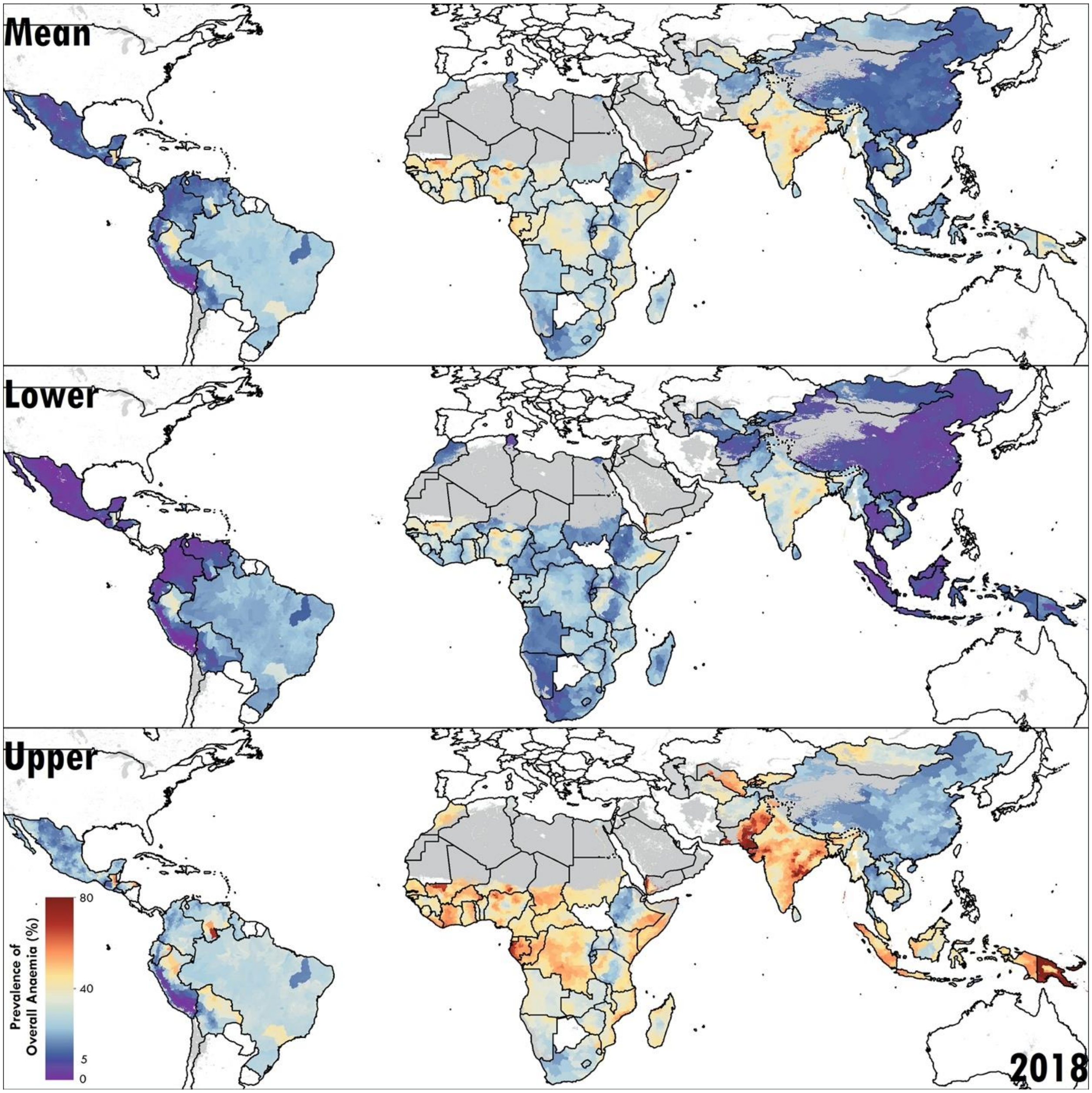

Supplementary Figure 10: Anaemia posterior means and upper and lower 95% uncertainty intervals

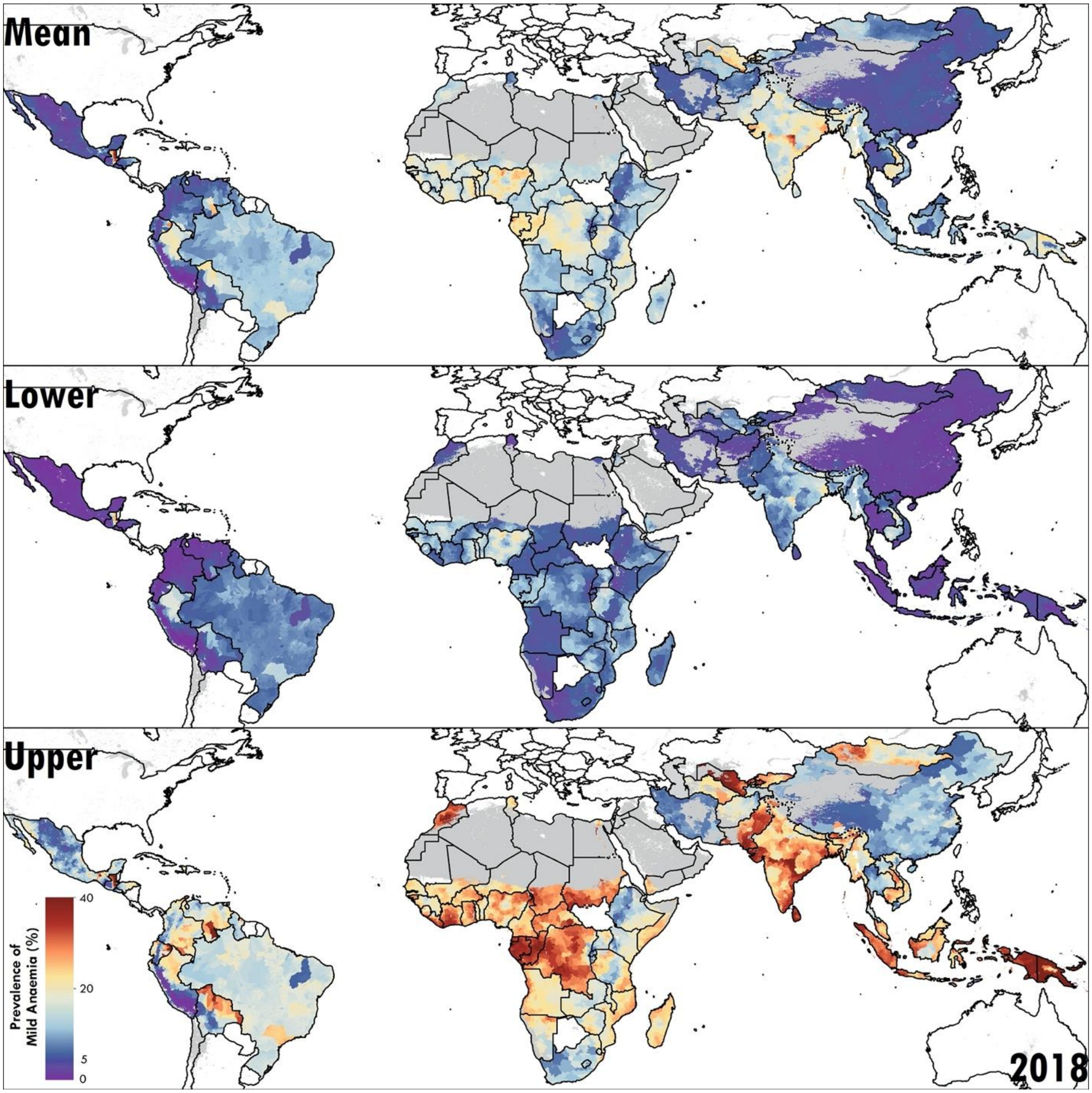

Supplementary Figure 11: Mild anaemia posterior means and upper and lower 95% uncertainty intervals

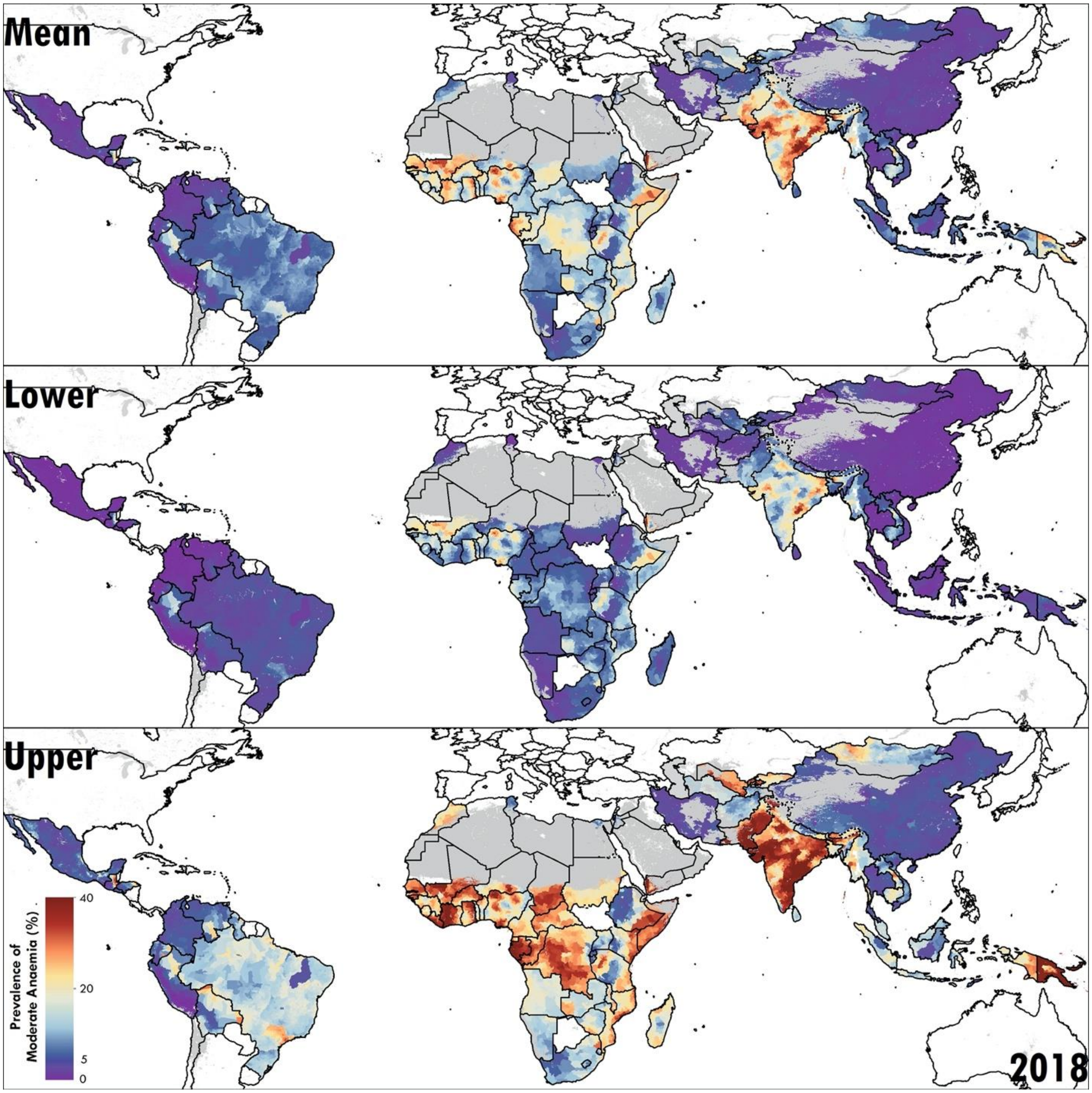

Supplementary Figure 12: Moderate anaemia posterior means and upper and lower 95% uncertainty intervals

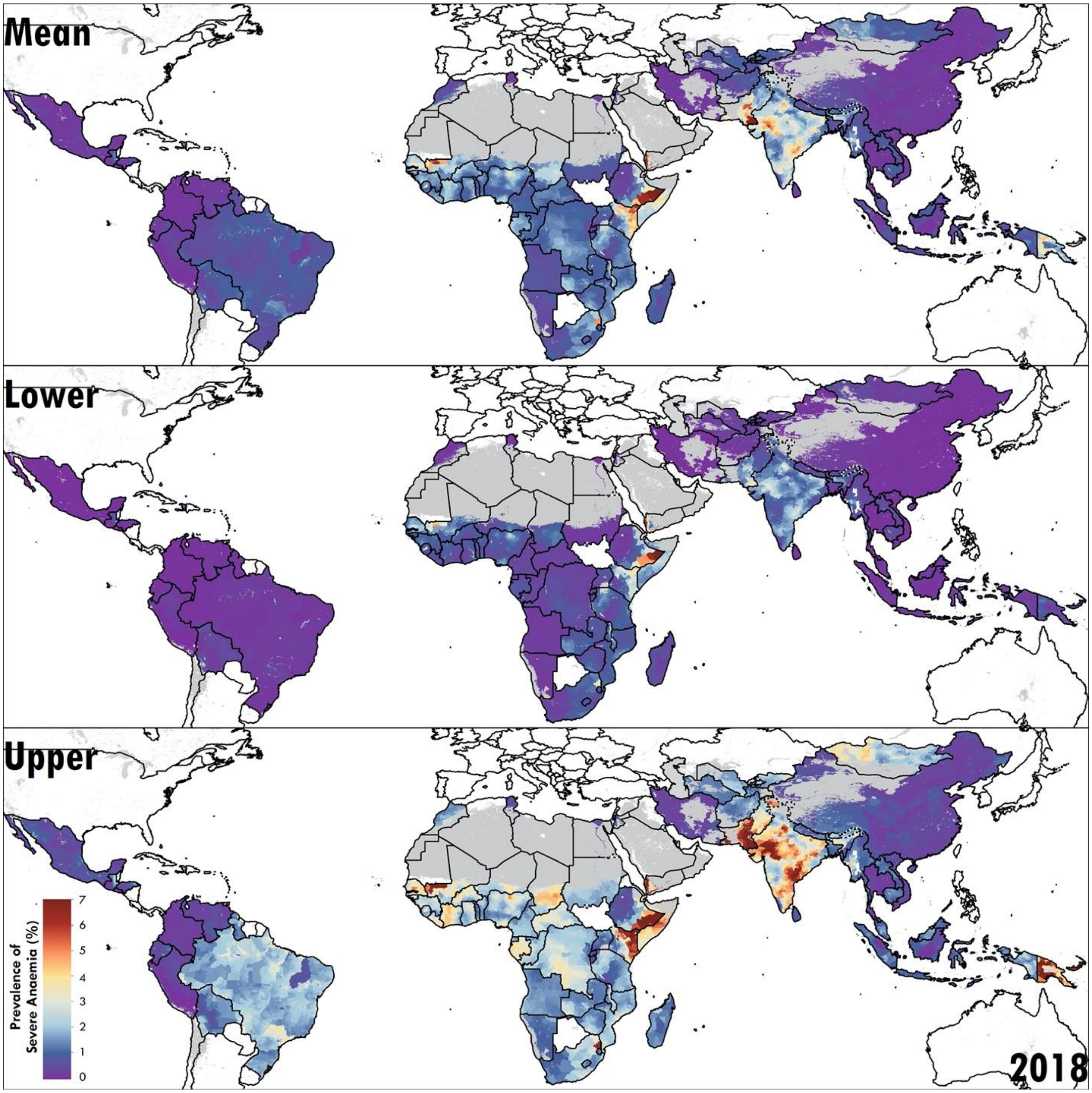

Supplementary Figure 13: Severe anaemia posterior means and upper and lower 95% uncertainty intervals

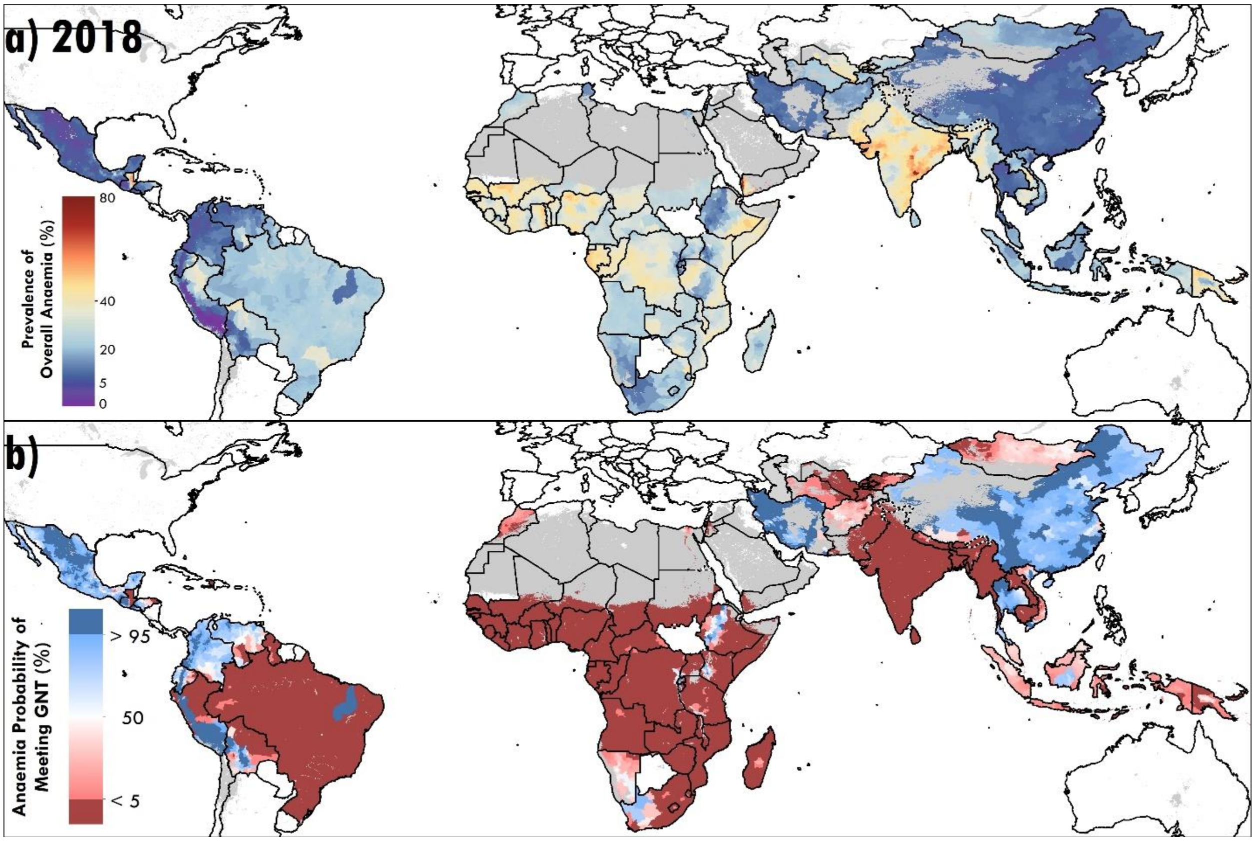

Supplementary Figure 14: Prevalence for overall anaemia among WRA in 2018, and probability of achieving the WHO GNT for overall anaemia by 2018

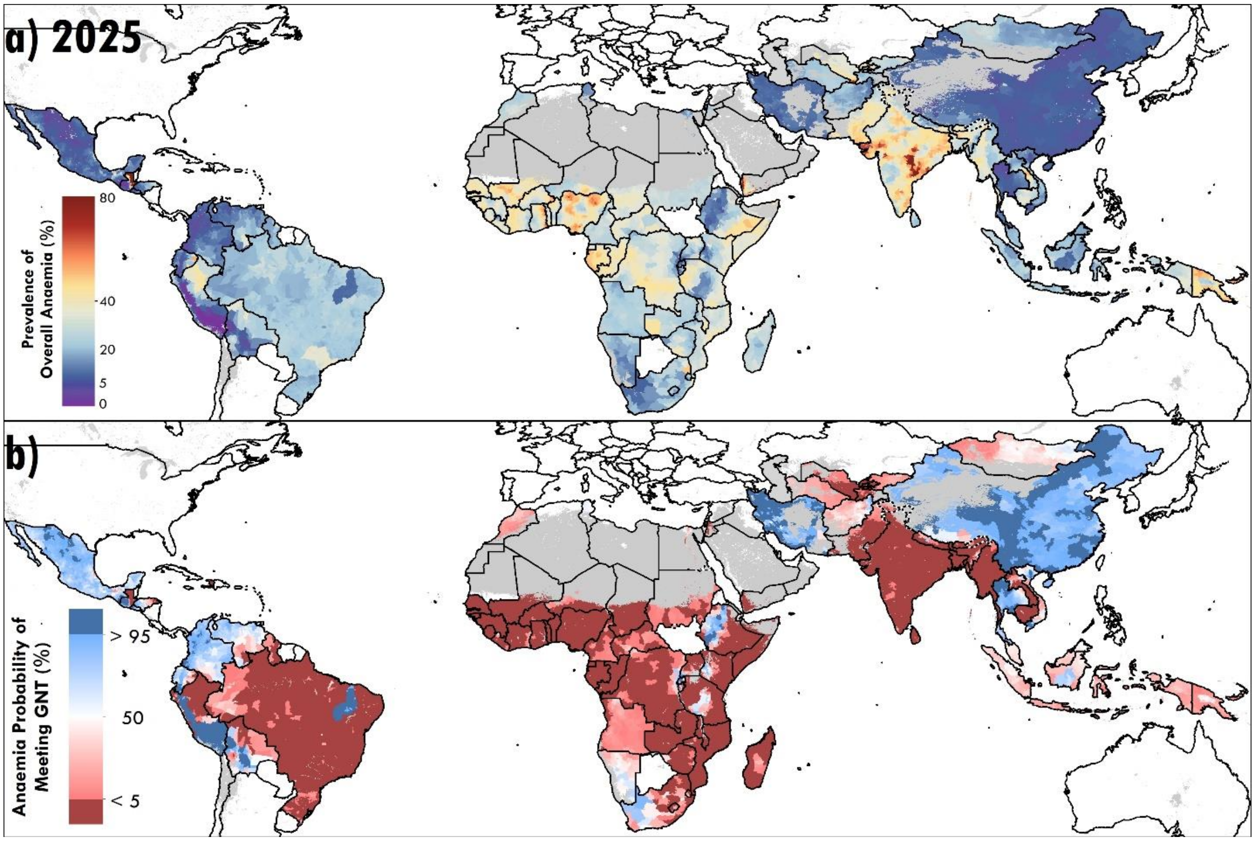

Supplementary Figure 15: Prevalence for overall anaemia among WRA in 2025, and probability of achieving the WHO GNT for overall anaemia by 2025

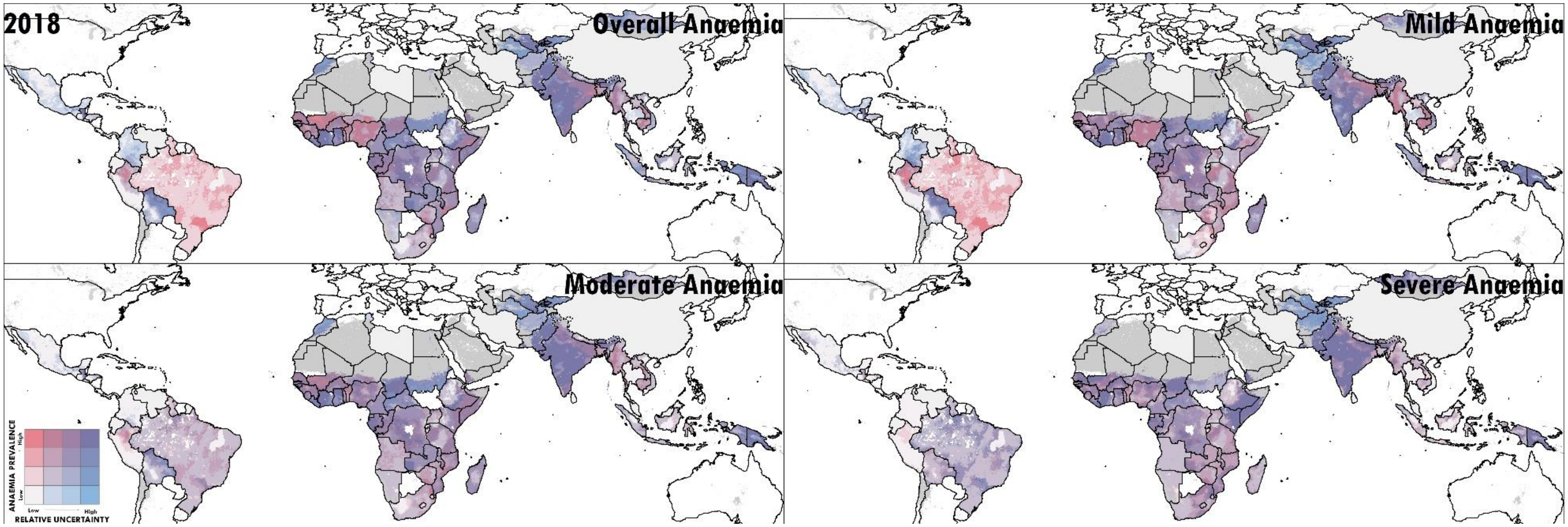

Supplementary Figure 16: Overlapping population-weighted quartiles of overall anaemia and relative 95% uncertainty in 2018

6.0 Model validation

6.1 Anaemia validation metrics

Supplementary Table 16: Predictive metrics for anaemia aggregated to admin 0

The out-of-sample (OOS) column indicates whether the metric was calculated using in-sample or out-of-sample predictions. Mean error, root-mean-squared-error (RMSE), correlation (Corr) and coefficient of variation (Cov) are in proportion.

Year	OOS	Median SS	Mean err.	RMSE	Corr.	95% Cov.
**2000**	FALSE	5364.000	-0.008	0.014	0.998	0.946
**2001**	FALSE	958.500	0.001	0.035	0.989	0.992
**2002**	FALSE	1683.000	-0.006	0.023	0.981	0.960
**2003**	FALSE	5370.000	-0.006	0.008	0.998	0.983
**2004**	FALSE	2730.000	-0.005	0.008	0.998	0.995
**2005**	FALSE	4133.500	0.001	0.011	0.998	0.989
**2006**	FALSE	4522.500	-0.009	0.046	0.977	0.904
**2007**	FALSE	1750.000	0.007	0.017	0.993	0.921
**2008**	FALSE	4768.000	-0.008	0.041	0.981	0.994
**2009**	FALSE	3933.000	-0.005	0.015	0.991	0.979
**2010**	FALSE	7237.500	-0.002	0.010	0.998	0.989
**2011**	FALSE	7024.500	0.001	0.012	0.996	0.987
**2012**	FALSE	5182.500	0.001	0.008	0.999	0.992
**2013**	FALSE	6195.500	-0.001	0.006	1.000	0.988
**2014**	FALSE	3426.000	0.006	0.024	0.987	0.986
**2015**	FALSE	12935.000	-0.001	0.005	0.999	0.981
**2016**	FALSE	5231.080	-0.009	0.018	0.997	0.994
**2017**	FALSE	9407.500	0.000	0.006	0.997	0.990
**2018**	FALSE	14502.000	0.003	0.006	1.000	0.996

Supplementary Table 17: Predictive metrics for anaemia aggregated to admin 1

Year	OOS	Median SS	Mean err.	RMSE	Corr.	95% Cov.
**2000**	FALSE	109.000	-0.008	0.032	0.988	0.946
**2001**	FALSE	236.000	0.001	0.040	0.986	0.995
**2002**	FALSE	597.484	-0.006	0.033	0.988	0.959
**2003**	FALSE	571.000	-0.006	0.025	0.991	0.982
**2004**	FALSE	328.000	-0.005	0.023	0.988	0.995
**2005**	FALSE	417.500	0.001	0.023	0.992	0.989
**2006**	FALSE	37.000	-0.009	0.057	0.959	0.904
**2007**	FALSE	312.000	0.007	0.025	0.986	0.914
**2008**	FALSE	406.000	-0.008	0.048	0.976	0.994
**2009**	FALSE	363.000	-0.005	0.039	0.969	0.980
**2010**	FALSE	350.500	-0.002	0.028	0.986	0.989
**2011**	FALSE	298.500	0.001	0.022	0.991	0.987
**2012**	FALSE	683.500	0.001	0.021	0.995	0.992
**2013**	FALSE	385.000	-0.001	0.019	0.995	0.989
**2014**	FALSE	507.000	0.006	0.028	0.987	0.985
**2015**	FALSE	1196.000	-0.001	0.008	0.998	0.981
**2016**	FALSE	64.000	-0.009	0.031	0.989	0.994
**2017**	FALSE	716.500	0.000	0.023	0.978	0.989
**2018**	FALSE	609.500	0.003	0.012	0.998	0.997

Supplementary Table 18: Predictive metrics for anaemia aggregated to admin 2

Year	OOS	Median SS	Mean err.	RMSE	Corr.	95% Cov.
**2000**	FALSE	27.648	-0.008	0.059	0.960	0.921
**2001**	FALSE	48.675	0.001	0.056	0.972	0.994
**2002**	FALSE	271.000	-0.006	0.044	0.984	0.959
**2003**	FALSE	38.000	-0.006	0.048	0.968	0.984
**2004**	FALSE	1.841	-0.005	0.039	0.968	0.996
**2005**	FALSE	81.969	0.001	0.041	0.976	0.989
**2006**	FALSE	1.766	-0.009	0.070	0.937	0.905
**2007**	FALSE	38.995	0.007	0.044	0.958	0.927
**2008**	FALSE	33.292	-0.008	0.062	0.962	0.992
**2009**	FALSE	61.000	-0.005	0.052	0.952	0.981
**2010**	FALSE	38.217	-0.002	0.045	0.967	0.988
**2011**	FALSE	32.000	0.001	0.049	0.962	0.986
**2012**	FALSE	68.643	0.001	0.035	0.987	0.993
**2013**	FALSE	32.000	-0.001	0.055	0.961	0.988
**2014**	FALSE	40.000	0.006	0.060	0.953	0.987
**2015**	FALSE	622.000	-0.001	0.025	0.986	0.981
**2016**	FALSE	25.639	-0.009	0.044	0.980	0.993
**2017**	FALSE	110.000	0.000	0.041	0.940	0.989
**2018**	FALSE	31.000	0.003	0.042	0.978	0.996

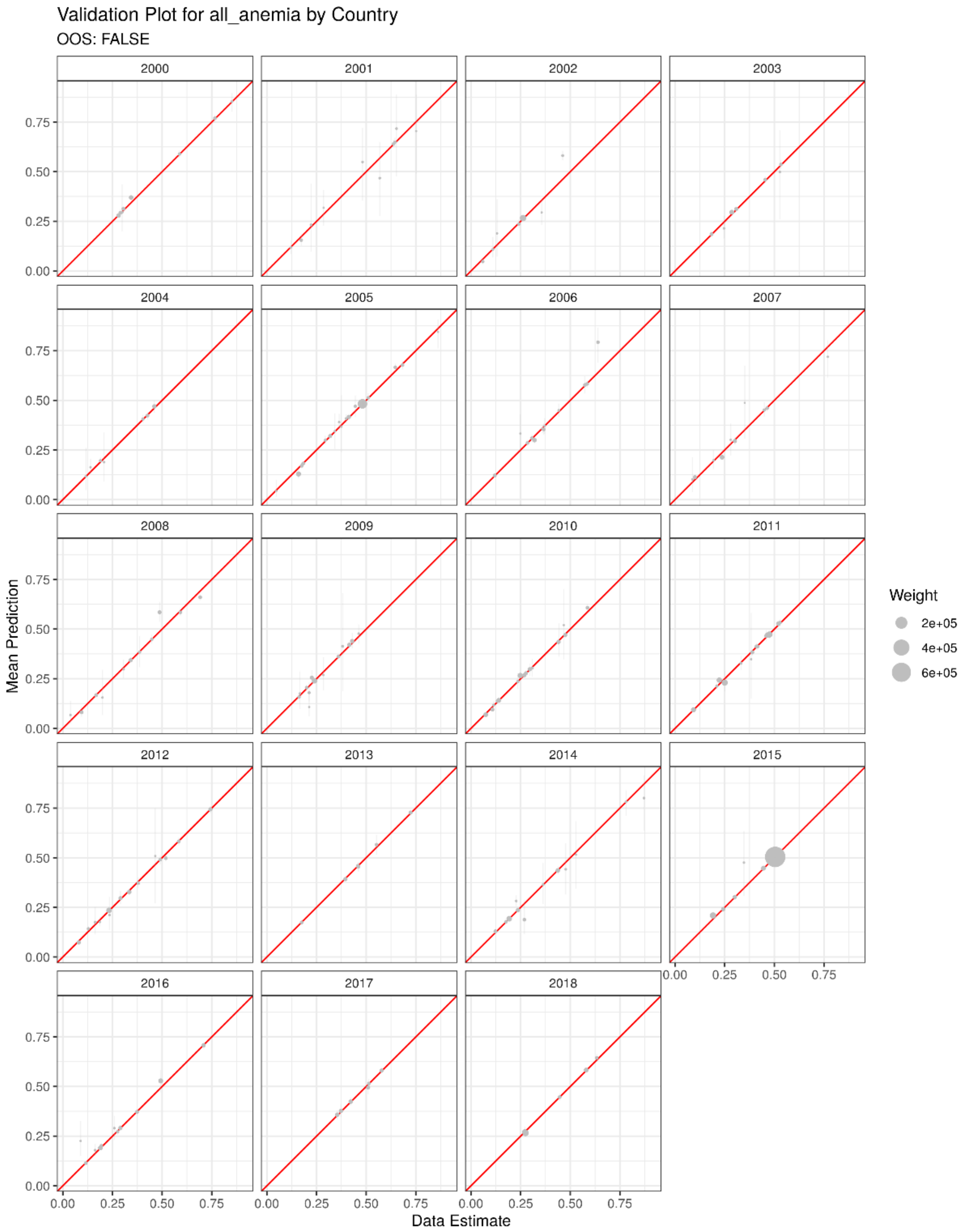

Supplementary Figure 17: Anaemia admin 0 aggregation in-sample

Comparison of in-sample anaemia predictions aggregated to admin 0 with 95% uncertainty intervals plotted against admin 0 aggregated data observations.

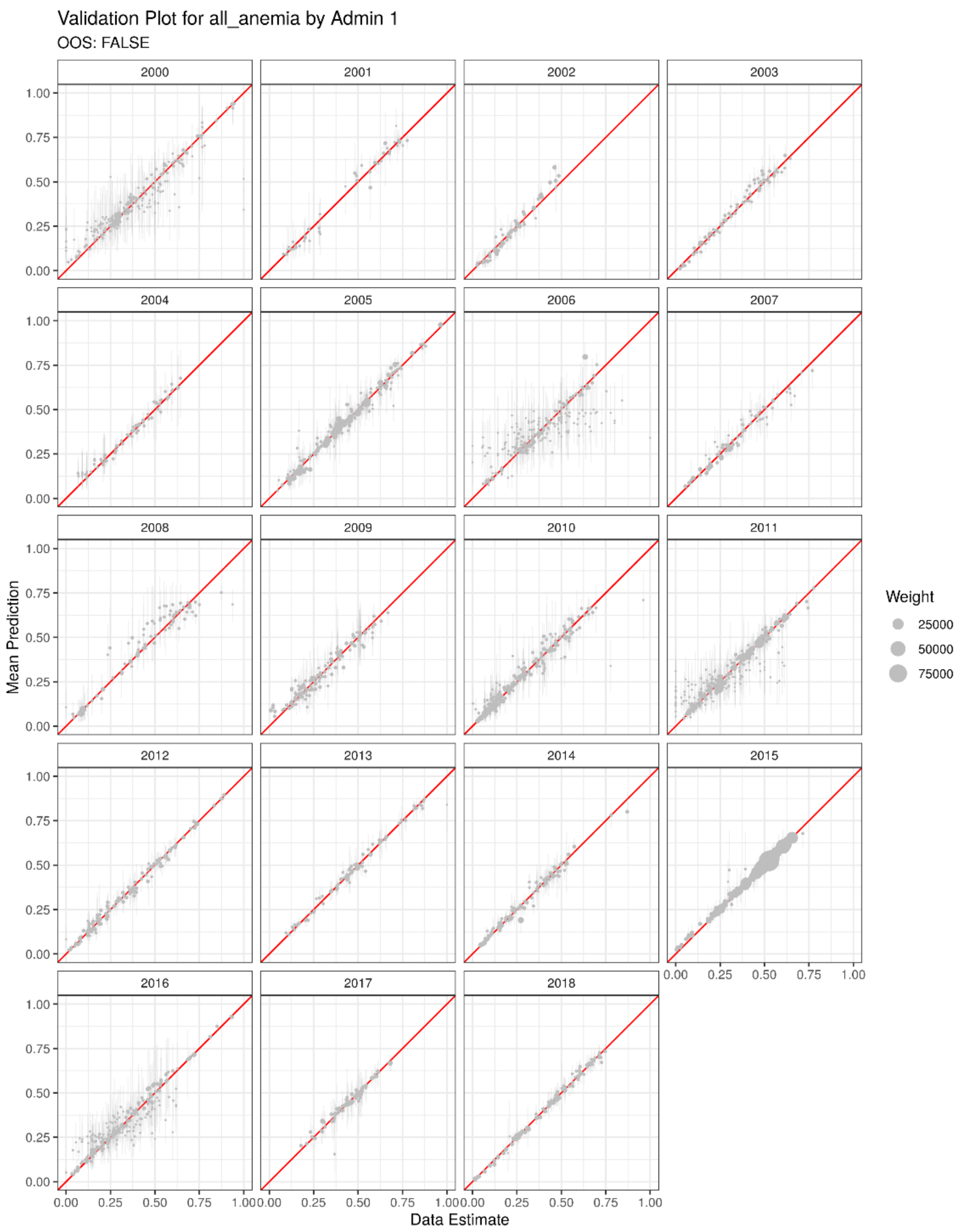

Supplementary Figure 18: Anaemia admin 1 aggregation in-sample

Comparison of in-sample anaemia predictions aggregated to admin 1 with 95% uncertainty intervals plotted against admin 1 aggregated data observations.

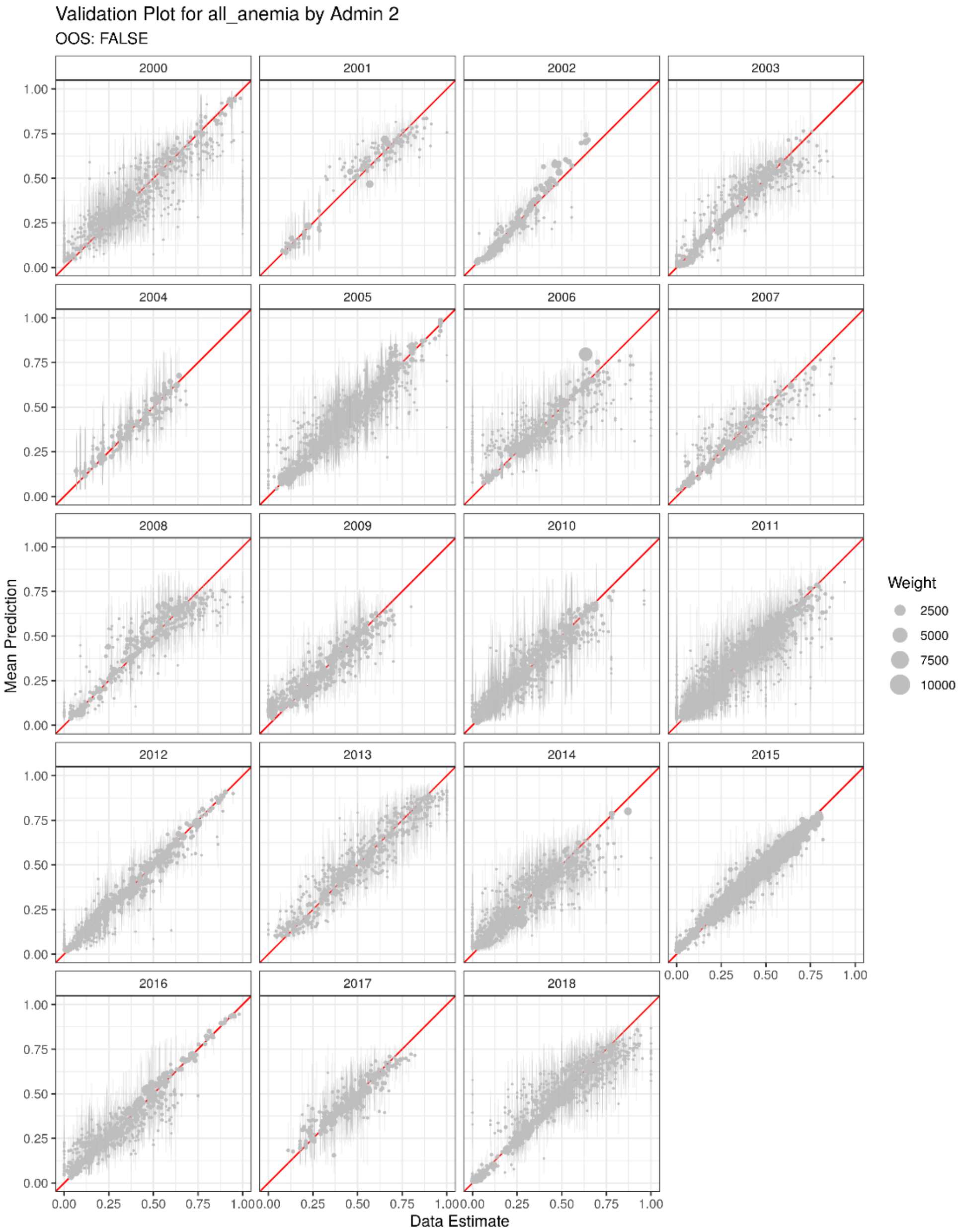

Supplementary Figure 19: Anaemia admin 2 aggregation in-sample

Comparison of in-sample anaemia predictions aggregated to admin 2 with 95% uncertainty intervals plotted against admin 2 aggregated data observations.

6.2 Anaemia sensitivity analysis

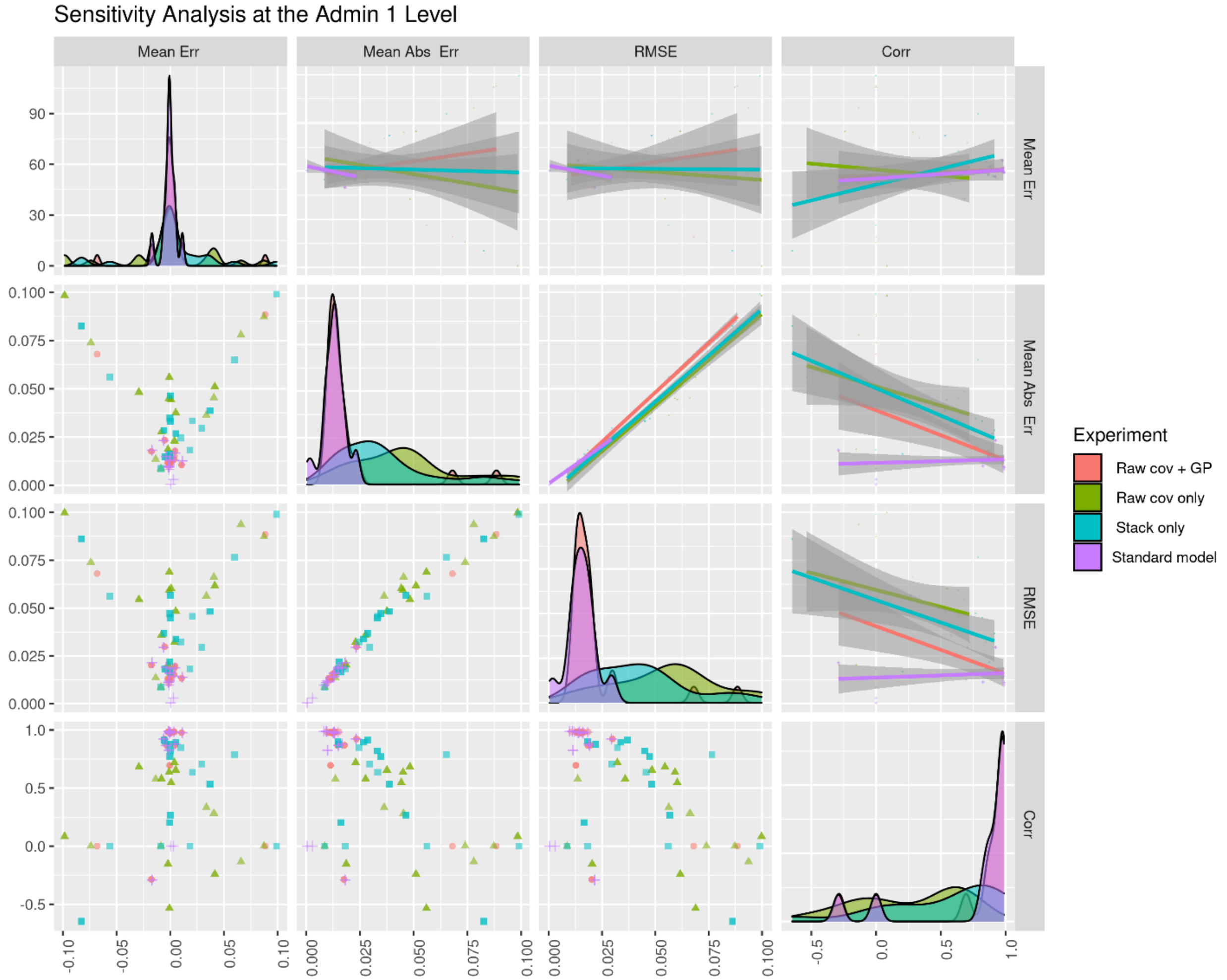

Supplementary Figure 20: Sensitivity analysis results admin 1 level

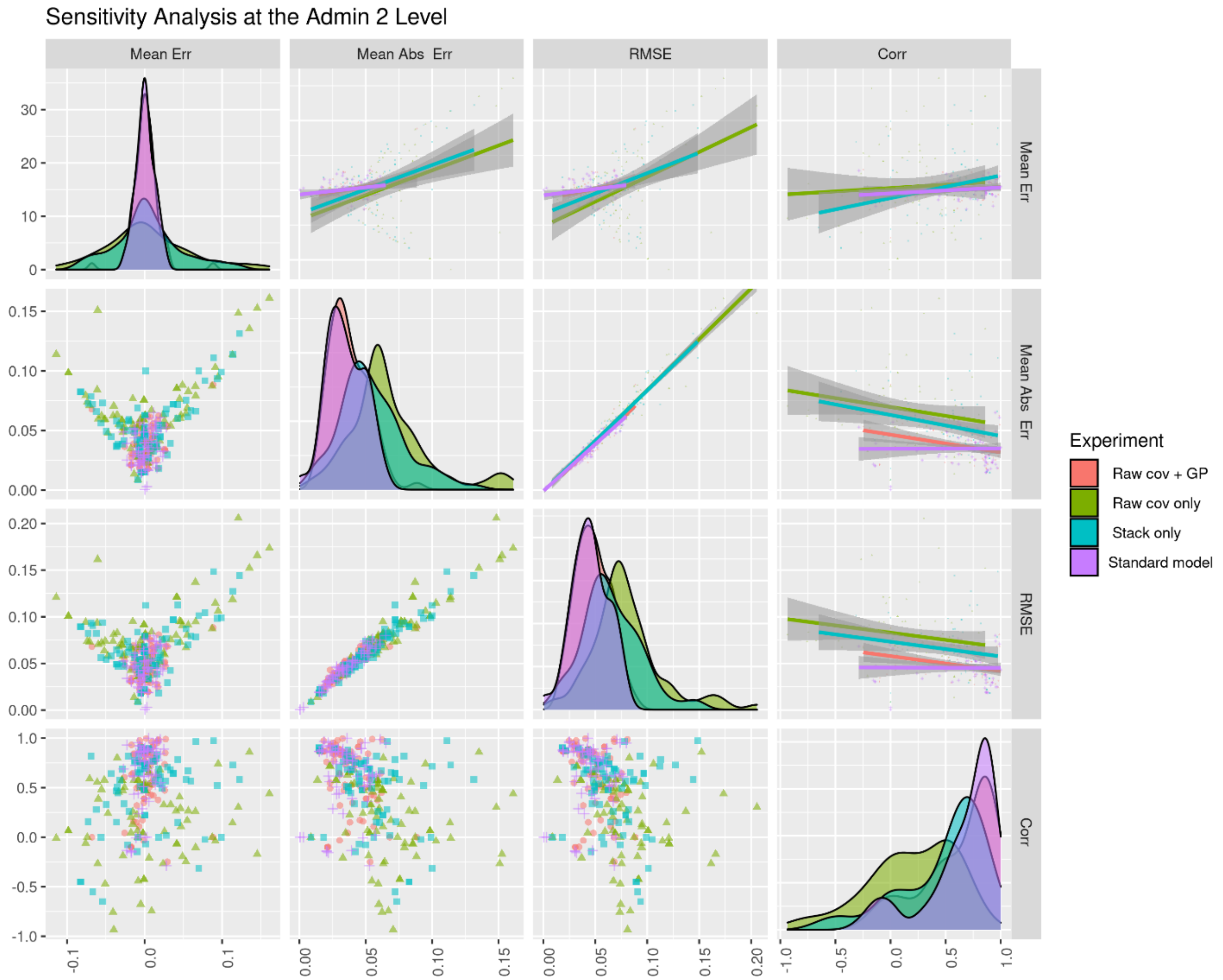

Supplementary Figure 21: Sensitivity analysis results admin 2 level

7.0 Supplementary references

1. Dicker, D. *et al.* Global, regional, and national age-sex-specific mortality and life expectancy, 1950–2017: a systematic analysis for the Global Burden of Disease Study 2017. *Lancet* **392**, 1684–1735 (2018).
2. Roberts, D. R. *et al.* Cross-validation strategies for data with temporal, spatial, hierarchical, or phylogenetic structure. *Ecography* n/a-n/a (2017) doi:10.1111/ecog.02881.
3. Global Administrative Areas (GADM). GADM database of global administrative areas. http://www.gadm.org (2018).
